# Polymorphisms in *Pf*kelch13 domains before and after the introduction of artemisinin-based combination therapy in Southwest Nigeria

**DOI:** 10.1371/journal.pone.0316479

**Published:** 2025-03-31

**Authors:** Abiodun Amusan, Olugbenga Akinola, Kazeem Akano, María Hernández-Castañeda, Jenna K. Dick, Akintunde Sowunmi, Geoffrey Hart, Grace Gbotosho

**Affiliations:** 1 Department of Pharmacology and Toxicology, Faculty of Pharmacy, University of Ibadan, Ibadan, Nigeria; 2 Malaria Research Laboratories, Institute for Medical Research and Training, College of Medicine, University of Ibadan, Ibadan, Nigeria; 3 Department of Pharmacology and Therapeutics, College of Medicine, University of Ibadan, Ibadan, Nigeria; 4 Department of Biological Sciences and African Centre of Excellence for Genomics of Infectious Diseases, Redeemer’s University, Ede, Osun State, Nigeria; 5 Department of Medicine, Center for Immunology, University of Minnesota, Minneapolis, Minnesota, United States of America.; University of Health and Allied Sciences, GHANA

## Abstract

Emergence of artemisinin resistance in East Africa has greatly necessitated surveillance of artemisinin resistance in other African countries with great malaria burden. Specific mutations within the β-propeller and BTB/Poz domains of *Pf*kelch13 gene are validated markers of artemisinin resistance. Sparse information exists on mutations outside these domains that may also contribute to artemisinin resistance. This study evaluated the occurrence and frequency of mutations in all domains of *Pf*kelch13 gene, and their impact on treatment outcome in Ibadan pre- and post-adoption of Artemisinin-based Combination Therapies (ACTs) in Nigeria. Dry blood spots prepared from blood samples obtained from *P. falciparum*-infected patients during retrospective (2000–2005) and prospective (2021) studies were analysed. Treatment outcomes with dihydroartemisinin-piperaquine were evaluated in a cohort of patients from the prospective study during a 42-day follow-up. Nested amplifications of *Pf*kelch13 gene fragments were done and subjected to Sanger dideoxy sequencing. Mutations were identified by sequence alignment and correlated with treatment outcome parameters including parasite clearance time (PCT) and day 2 parasite reduction ratio (PRRD_2_) among others. Mean PCT was 2.1 ± 0.6 days (95%CI: 1.97–2.24) while PRRD_2_ was 4815 with 100% adequate clinical and parasitological response. Altogether, 64 (11 retrospective/53 prospective) samples were successfully sequenced. None of the β-propeller domain mutations validated as artemisinin resistance markers were found within the analysed samples. However, four distinct mutations, K189T (64.2%), K189N (1.9%), R255K (3.8%), and N217H (1.9%) were identified within the N-terminal domain of the prospective samples while the K189T mutation was identified in a retrospective sample obtained in 2003. The K189T and R255K mutations correlated significantly with longer parasite clearance time in treated patients (P < 0.002). There was no evidence of validated molecular marker of artemisinin resistance within the ß-propeller domain of *Pf*kelch13. However, frequency of K189T mutation and its significant correlation with longer PCT may suggest possibilities of geographical variations in genetic drivers of artemisinin resistance.

## Introduction

Malaria is an age-long global health threat and its control has necessitated deployment of several chemotherapeutic approaches [1]. The burden of this disease is highest in tropical countries within sub-Saharan Africa [2]. For instance, about 94% of the total global burden of malaria is domiciled in this region and Nigeria accounted for 31% of global malaria-related deaths in 2022 [3]. The higher prevalence of malaria mortality in this region is attributable to the dominance of *Plasmodium falciparum*, the most severe form of malaria infection in the population [4]. Moreso, the warm temperature in tropical climates ( > 20°C) is essentially favourable for complete asexual life cycle development of *P. falciparum* within the mosquito vector [5]. Unfortunately, *P. falciparum* has developed resistance to almost all known antimalarial drugs [6] and its resistance to artemisinins, is seriously undermining global malaria control efforts [7].

The global adoption of artemisinin-based combination therapies (ACTs) as the first-line option for malaria treatment and its deployment in Nigeria in 2005 revived the hope for malaria control and elimination. This followed widespread incidences of treatment failures to earlier antimalarials such as chloroquine and sulphadoxine-pyrimethamine (SP) which resulted in a skyrocketed increase in malaria-related morbidity and mortality [8]. The adoption of ACTs for the treatment of acute uncomplicated malaria was accompanied by significant success in malaria control in most malaria-endemic countries and globally [9,10]. Unfortunately, emergence of artemisinin-resistant parasites which emanated from southeast Asia and its continuous spread is posing serious threats to the efficacy of ACTs [11].

Historically, the Greater Mekong Subregion (GMS) in southeast Asia is the geographical origin of resistance to most antimalarial drugs [12–14]. The known major drivers of artemisinin resistance identified in this region are single nucleotide polymorphisms (SNPs) within the BTB-POZ and β-propeller domains of the gene encoding kelch13 protein in *P. falciparum* [12,15]. Over 250 *Pf*kelch13 mutations have been identified through genomic surveillance of field isolates in the region [16]. Of these, 13 have been validated by WHO as associated with artemisinin resistance through significant correlations with both *in-vivo* delayed parasite clearance and *in-vitro* reduced susceptibility to artemisinin [15,16]. These validated SNPs are C580Y, Y493H, R539T, P553L, I543T, F446I, C469Y, R561H, N458Y, M476I, P574L, R6221 and A675V [16].

The spread of artemisinin resistance is assuming a similar pattern to earlier antimalarial drugs: this involved spread within the GMS, South America, and subsequently to sub-Saharan Africa [8]. Recently, indigenous emergence of some validated artemisinin resistance markers was reported in some East African countries with significant *in-vivo* correlation with prolonged parasite clearance half-lives [8,17,18]. Specifically, de novo emergence of the mutant R561H was reported in Tanzania and Rwanda [8,17,19] while A675V and C469Y mutations were reported in Uganda. These were established through observed positive correlation of mutations with delayed parasite clearance in patients following treatment with an ACT, and increased ring-stage survival of parasites in the presence of artemisinin in *in-vitro* studies. This has currently heightened the fear of a total spread of these resistant parasites to other parts of sub-Sahara Africa where the greatest global burden of the disease is domiciled [3]. Considering the lack of approved alternatives to ACTs for malaria treatment, the consequences of widespread artemisinin-resistant parasites within sub-Saharan African countries may be detrimental [8,20]. Of more concern are reported cases of delayed parasite clearance following complete doses of artemisinin-based combination therapies in sub-Saharan African countries [21,22]. Although no clinical evidence of artemisinin resistance has been reported in Nigeria, there have been cases of reduced efficacy of some of the currently used ACTs following complete treatment protocols [23–25].

Molecular surveillance has also been conducted in Nigeria to evaluate the presence of validated artemisinin resistance markers in the population [26–29]. However, none of the studies has reported any of these validated *Pf*kelch13 mutations in Nigeria. *Pf*kech13 gene comprises 3 domains: a parasite-specific N-terminal also called the coiled coil-containing domain, a highly conserved BTB-POZ domain, and a six-bladed β-propeller domain [7,30]. Most of the artemisinin resistance-associated *Pf*kelch13 mutations are domiciled within the β-propeller domain of the gene [30]. To date, there is sparse information on clinical correlations of mutations outside the propeller region of the *Pf*kelch13 gene.

The possibilities of pre-existence of markers of artemisinin resistance before the actual clinical emergence of artemisinin resistance in 2008 in Cambodia have been reported [15]. Moreso, a study revealed reduced susceptibility to artemisinin *in-vitro* long before the adoption of ACTs [31]. This was a predictor of parasites’ inherent genetic potential to develop resistance to artemisinins. However, the genomic profile of circulating parasites relative to artemisinin resistance before the introduction of ACTs is unclear. To date, no study has evaluated the pre-existence of molecular markers of artemisinin resistance before the adoption of artemisinin-based combination therapy in Nigeria.

Therefore, this study was conducted to evaluate mutations present in the three domains of *Pf*kelch13 gene in patient isolates of *P. falciparum* in southwest Nigeria before and after the introduction of ACTs in Nigeria and to determine the clinical relevance of such mutations in artemisinin resistance.

## Materials and methods

### Study design, and site

The study involved analysis of samples from a retrospective and a prospective study. The retrospective studies were antimalarial therapeutic efficacy studies conducted between 2000 and 2005 at the Malaria Clinic of the Malaria Research Laboratories, Institute for Advanced Medical Research and Training (IAMRAT), College of Medicine, University of Ibadan, before the adoption of ACTs in Nigeria. The information of patients enrolled in the retrospective studies such as the baseline age, weight, temperature, and parasitemia was accessed on 6th April 2021. Participants were deidentified and only their unique identification codes were available to the researchers. The prospective study was part of an ongoing drug therapeutic efficacy study of dihydroartemisinin-piperaquine conducted at the Malaria Clinic of the Malaria Research Laboratories, IAMRAT, College of Medicine, University of Ibadan. The study was an open-label single-arm study conducted between the period of 28th June 2021 to 19th November 2021. Ibadan is a malaria endemic area in southwest Nigeria, West Africa where malaria transmission occurs all year round and is more intense between April and October. Ibadan is located on latitude 7°22.6536′ N and longitude 3°54.3546′ E. The molecular analysis was conducted at the Hart Laboratory, Medical School, University of Minnesota, U.S.A.

### Ethics approval and consent to participate

Ethical approval for this study was obtained from both the Joint University of Ibadan/University College Hospital Ethics Committee (#UI/EC/20/0171) and the Oyo State Ministry of Health Research Ethics Committee (AD 13/479/4219). Registration of this study was also done, and approval was obtained from the Pan Africa Clinical Trial Registry (PACTR) with identification number PACTR202108584742856. Written informed consent was obtained from study participants and the parents/guardians of children. Assent was also obtained directly from children ≥10 years.

### Patients and sample collection

**Fig 1:**
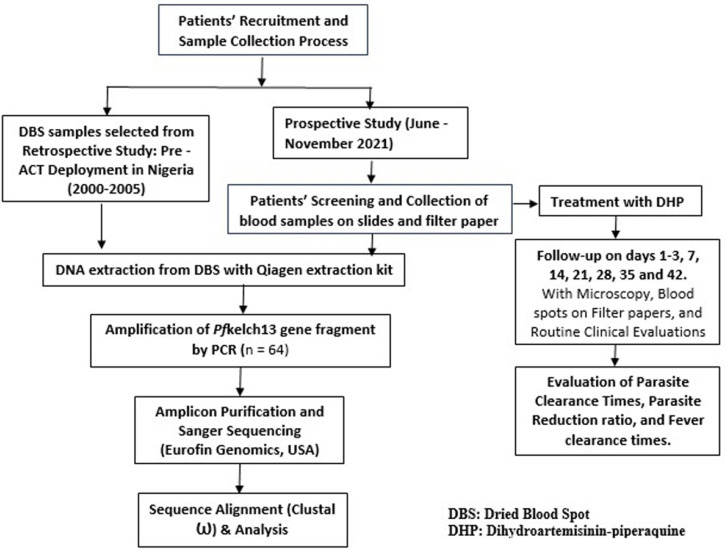
Flow chart showing patients’ recruitment and molecular analysis of *Pf*kech13 gene. This figure depicts the study design showcasing the organogram of patients’ enrolment, evaluation of endpoint parameters following treatment of uncomplicated malaria patients with dihydroartemisinin-piperaquine, and molecular analysis.

The study population were patients aged 0.9–35 years who presented to the malaria clinic for treatment during the study periods. Dry blood spots (DBS) collected before drug treatment during retrospective studies were selected by random purposive sampling. The selected DBS were identified by the participants’ unique identification code on the filter paper label. Patients were enrolled into the prospective study based on the following criteria: microscopically confirmed *Plasmodium* infection, a body (axillary) temperature ≥ 37.5°C, or history of fever within one to two days preceding presentation, absence of other concomitant illnesses, without any history of antimalarial drug use within 2 weeks before presentation, and obtainment of written informed consent from parents or guardians of participants. Patients were excluded from the study if they had severe malaria, serious underlying diseases (renal, cardiac, or hepatic), or severe malnutrition. The baseline demographic and parasitological parameters of all enrolled participants are described in Table 1. In the prospective study, patients were treated with a fixed-dose combination of dihydroartemisinin-piperaquine tablets (40 mg/320 mg) for 3 days according to their body weights. A cohort of the treated patients was followed up on days 1–3, 7, 14, 21, 28, 35, and 42 with microscopy, collection of blood spots on filter papers, and routine clinical evaluations. Treatment outcomes such as parasite clearance times (PCT), fever clearance times (FCT), and parasite reduction ratio were documented after the follow-up period. Finger-pricked blood samples were collected on 3MM Whatman^®^ filter paper before treatment initiation (day 0) and were allowed to dry at room temperature. The dried blood spots were stored in sealed plastic bags at room temperature with silica gel until molecular analysis. The study flowchart showing the clinical study, sample collection process, and molecular analysis is illustrated in Fig 1.

**Table 1:** Baseline demographic, clinical, and parasitological parameters of enrolled patients.

Parameters	Values
**Gender**	
Male/female	39/25
**Age (years)**	
Mean ± S.D	9.4 ± 5.3
Range	0.9–35
**Weight (kg)**	
Mean ± S.D	24.3 ± 9.6
Range	10–45
**Temperature (°C)**	
Mean ± S.D	37.5 ± 1.2
Range	35.5–40.2
**Hematocrit (%)**	
Mean ± S.D	34.4 ± 5.4
Range	23–49
**GMPD (µL**^**−1**^)	35402
**Range**	1261–384000

GMPD, geometric mean parasite density; S.D., standard deviation

### Extraction of parasite DNA

Genomic DNA was extracted from all the DBS using QIAamp DNA Mini blood kit (Qiagen, Germantown, MD, USA) according to the manufacturer’s protocol. Briefly, cells were lysed with 180 μL of buffer ATL from three 3mm punches of DBS, treated with 20 μL Proteinase K solution, and 200 μL each of buffer AL and ethanol (96–100%). Solutions were transferred into QIAamp Mini spin columns and washed with 500 μL each of AW1 and AW2 buffer. Pure DNA was eluted into a new 1.5 mL tube with 100 μL buffer AE. The extracted genomic DNA concentration was quantified on a nanodrop spectrophotometer and stored at 4°C.

### Amplification of *P. falciparum* kelch13 and amplicon purification

A total of 64 genomic DNA (11 retrospective and 53 prospective) samples were analysed for *Pf*kelch13 mutations. Two fragments containing the N-terminal/BTB-POZ domain (1312 bp) and the β-propeller domain (849 bp) of the *Pf*kelch13 gene were amplified in a nested polymerase chain reaction. The oligonucleotide primer pairs used have been previously reported [32,33] and were synthesized by Integrated DNA Technologies (IDT, Coralville, USA). The following primers were used for the primary amplification: Fw, 5′-TATAACAAGGCGTAAATATTCGTG-3′ and Rv, 5′-TGTGCATGAAAATAAATATTAAAGAAG-3′. For nested amplifications, Fw, 5′-GCGGAAGTAGTAGCGAGAAT-3′ and Rv, 5′-CGCCAGCATTGTTGACTAAT-3′ were used for the N-terminal/BTB-POZ domain while Fw, 5′-GCCTTGTTGAAAGAAGCAGA-3′ and Rv, 5′-GCCAAGCTGCCATTCATTTG-3′ were used for the β-propeller domain. The thermocycler was programmed with the following conditions: 94°C for 5 min, followed by 40 cycles of: 94°C for 30 s, 55°C for 90 s, and 68°C for 90 s, then a final extension at 68°C for 10 min.

Briefly, 12.5 μL reaction volume containing 6.25 μL iProof HF 2X master mix (Bio-Rad, Irvine, USA), 0.5 μL (10 μM) of each primer, 2.75 μL of nuclease-free water and 2.5 μL of genomic DNA template was used for the primary amplification. For the nested amplifications, 12.5 μL reaction volume containing 6.25 μL iProof HF 2X master mix (Bio-Rad, Irvine, USA), 0.5 μL (10 μM) of each primer, 4.25 μL of nuclease-free water, and 1 μL of genomic DNA template was used for each nested amplification. The template for the nested reaction was a 1:100 dilution of primary reaction amplicons. Each PCR contained a *P. falciparum* 3D7 genomic DNA template as positive control while a reaction containing *P. falciparum*-negative DNA and a no-template reaction served as negative controls. Amplicons were resolved on 1% agarose gel stained with SYBR green dye (Invitrogen, Carlsbad, USA) using a GeneRuler 1 kb DNA Ladder (ThermoFisher, Waltham, USA). Agarose gel images of the amplified fragments were viewed in Gel Doc EZ Imager (Bio-Rad, Irvine, USA) documentation system.

Amplicons were purified by enzymatic clean-up with a mixture of exonuclease 1 (ThermoFisher, Waltham, USA) and Shrimp Alkaline Phosphatase (SAP) (ThermoFisher, Waltham, USA) (ExoSAP). This was done to remove residual PCR components such as excess nucleotides and primers that may interfere with sequencing.

### Sequence data analysis

Purified amplicons were subjected to bidirectional Sanger dideoxy sequencing at Eurofins Genomics commercial sequencing centre (Eurofins, USA). The sequences were viewed and base called with the Bioedit software version 7.2.6.1. Manual base calling was also carried out for some of the sequences where necessary. The *Pf*kelch13 sequences were aligned with *Pf*3D7 reference sequence (Gene ID: *Pf*3D7_1343700) obtained from the National Centre for Biotechnology Information database using Clustal omega alignment program. All identified changes in nucleotide sequence were reported. Sequence alignment was done for both the forward and reverse sequences of each sample to validate all the identified single nucleotide polymorphisms.

### Statistical analysis

Data were analysed using the Statistical Package for Social Sciences (SPSS) version 25 and GraphPad Prism version 9.01. Genotypes were expressed as frequencies and percentages. Parasitological parameters such as parasite densities and parasite clearance times (PCT) evaluated in the prospective study were compared between the groups of patients carrying the *Pf*kelch13 SNPs and the wildtypes. Comparison was done using student’s t-test. P-values of ˂0.05 were considered statistically significant.

## Result

### Treatment responses of patients followed up in the prospective study

The mean parasite clearance time of the followed-up patients was 2.1 ± 0.6 days (95% CI: 1.97–2.24) while the mean fever clearance time was 1.3 ± 0.7 days (95% CI: 1.1–1.6). The patients’ geometric mean parasite reduction ratio on day 2 (PRRD2) was 4815 (95% CI: 942–8687). There was no recrudescence/reinfection till day 42 in the observed study participants. The overall PCR corrected day 42 adequate clinical and parasitological response to treatment (ACPR) was 100%.

### Single nucleotide polymorphisms within the domains of *Pf*kelch13 gene

**Fig 2:**
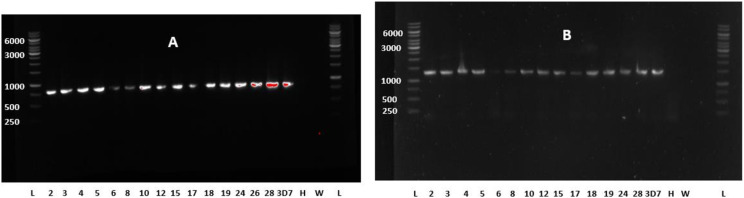
Agarose Gel Images of the Amplified Domains of *Pf*kelch13 Gene. 3D7: Positive control H: Human malaria negative DNA, W: Water. **(A)** Agarose gel electrophoretic resolution of downstream *Pf*kelch 13 propeller domains with an expected amplicon size of 849 bp highlighting the positive amplification of the β-Propeller domain of *Pf*kelch13 gene from selected representative samples. **(B)** Gel resolution of upstream *Pf*kelch13 gene capturing the BTB-POZ and the coiled-coil containing (CCC) domain with an amplicon size of 1312 bp. The human malaria-negative DNA (H), and nuclease-free water (W) show no amplification band, while the ladder (L) indicates the reference sizes.

The agarose gel images of the amplified fragments of *Pf*kelch13 gene are presented in Fig 2. All the 64 samples analysed for *Pf*kelch13 mutations were successfully sequenced with interpretable results. Following alignment with a reference sequence, none of the WHO-validated SNPs that are associated with artemisinin resistance domiciled within the β-propeller domain was found. No other synonymous or non-synonymous mutation was found in this domain. However, within the N-terminal domain, four distinct single nucleotide polymorphisms were identified while no nucleotide changes were found within the BTB/POZ domain. Within the N-terminal domain, there were changes from amino acid lysine (K) to threonine (T) (K189T) or asparagine (K189N) at position 189 with frequencies of 64.2% and 1.9% respectively in the prospective samples. In addition, there were changes from asparagine to histidine (H) at position 217 (N217H) and from arginine (R) to lysine (R255K) at position 255 with frequencies of 1.9% and 3.8% respectively. Furthermore, K189T mutation was identified in one of the retrospective samples obtained in year 2003 while none of the other 3 mutations was found among the retrospective samples. Details of the identified SNPs and their frequencies are shown in Table 2.

**Table 2:** Positions and frequencies of identified SNPS within the amplified fragments of *Pf*kelch13.

Amino acid change	SNP	Frequency [percentage (%)]		Overall frequency percentage (%)
		Retrospective	Prospective	
K to T	K189T	1/11(9.1)	34/53 (64.2)	35/64 (54.7)
K to N	K189N	0/11 (0)	1/53 (1.9)	1/64 (1.6)
R to K	R255K	0/11 (0)	2/53 (3.8)	2/64 (3.2)
N to H	N217H	0/11 (0)	1/53 (1.9)	1/64 (1.6)

SNP, single nucleotide polymorphism

### Association between identified SNPs within the N-terminal domain of *Pf*kelch13 and parasitological outcomes

**Fig 3:**
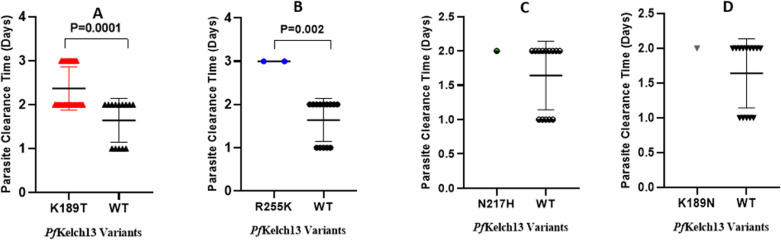
Association between *Pf*kelch13 SNPs and parasite clearance time (PCT) in patients enrolled in the prospective clinical study. Panels (**A**) and (**B**) depict a significant association between the single nucleotide polymorphisms, K189T and R255K respectively, and longer mean parasite clearance times. There was a significant delay in parasite clearance following drug treatment in patients harbouring parasite strains with the above mutations compared to patients carrying the wildtype. Conversely, there was no association between the mean parasite clearance times in patients carrying parasites with the N217H (**C**) or K189N (**D**) mutations compared to the wildtype (P = 0.5).

Mean parasite clearance time was longer in patients who had parasites with K189T mutations (P = 0.0001) and R255K mutations (P = 0.002) compared to the wild-type variants. The mean parasite clearance times in patients carrying parasites with K189N and N217H mutations were similar to the wild type (P = 0.5). The mean PCTs in patients carrying parasites with the *Pf*kelch13 variants are shown in Fig 3.

**Fig 4:**
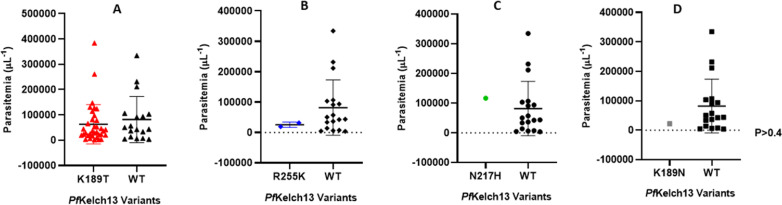
Association between *Pf*kelch13 SNPs and baseline parasitemia in patients enrolled in the prospective clinical study. There was no significant difference between the mean baseline parasite density differentials and identified polymorphisms in parasite DNA in all enrolled participants. Patients carrying parasites with SNPs within the BTB-POZ and coiled coil-containing (CCC) domain of the *Pf*kelch13 did not present with significantly different parasitemia when compared to patients carrying *Pf*kelch13 wildtype variant.

However, the mean baseline parasitemia of patients carrying parasites with the identified mutations was similar to the wildtype variants (P > 0.4) as shown in Fig 4.

## Discussion

Efforts in malarial control are currently being threatened by resistance to artemisinins and further threats are envisaged by the increasing spread of these resistant parasites beyond the Southeast Asian domain [17,19,34]. With its emergence in East Africa, the postulation of a total spread of artemisinin-resistant parasites to sub-Sahara Africa by 2040 portends great danger to this malaria-endemic region and the world at large [7]. The endemicity of malaria in the sub-Saharan African region and the exclusive reliance on ACTs for acute uncomplicated malaria emphasizes the need to curtail the emergence of artemisinin resistance in this region. The necessity for continuous and extensive monitoring and surveillance of the presence of artemisinin-resistant parasites in this region is growing stronger day by day. Peculiar attention is therefore required in Nigeria with a significantly high burden of malaria [3]. This study involved molecular profiling of *Plasmodium falciparum* kelch13 gene in Ibadan, southwest Nigeria to evaluate the presence of the validated molecular markers of artemisinin resistance and other identified mutations.

Identification of any of the validated markers of artemisinin resistance in a population is a reliable approach for monitoring malaria parasites’ susceptibility to this drug. In our present study, none of the artemisinin resistance-associated mutations were seen in the population. This implied that no genetic-based evidence of de novo emergence or spread of artemisinin-resistant parasites exists in the study population. This observation supports the 100% adequate clinical and parasitological responses observed in the patients whose samples were analysed in this study. This current report is in tandem with similar studies conducted earlier in other parts of southwest Nigeria [26,35,36] or northern Nigeria [27]. In these earlier studies, none of the WHO-validated *Pf*kelch13 mutations that were reported in Southeast Asian countries were reported as well. Furthermore, similar studies conducted in Nigeria’s neighbouring countries such as Cameroun and Niger Republic also reported absence of these artemisinin resistance validated markers [37,38].

Currently, malaria treatment in Nigeria and other malaria-endemic countries in sub-Sahara Africa relies absolutely on Artemisinin-based combination therapies as the first- and second-line treatment options [9]. The current finding provides some assurance on the continuous efficacy of dihydroartemisinin-piperaquine in this region, therefore strengthening the prospect of malaria control in Nigeria. However, this also awakens the need for more strict measures to continuously preserve its efficacy.

To date, no mutation within the N-terminal (coiled coil-containing) domain of *Pf*kelch 13 has been validated as associated with artemisinin resistance [7]. Interestingly, four distinct mutations were identified within this domain in this study (K189T, K189N, R255K, N217H). Of notable interest is the significantly high frequency of K189T mutation (64.2%) within the population. This is in tandem with the reported prevalence in a similar study conducted in Lagos, the most populous metropolitan city in Nigeria and West Africa [39]. Other studies in Africa had reported a similarly high prevalence of the K189T mutations. For instance, prevalence of 53.12% and 62.8% were reported in Equatorial Guinea and Uganda respectively [40,41]. Thus, making K189T mutation the most prevalent mutation ever reported in Africa outside the β-propeller region of *Pf*kelch13 [42]. In contrast, this mutation is rarely reported in Southeast Asia [43,44]. In the absence of validated molecular evidence of artemisinin resistance in this study region despite earlier reports of declining responsiveness of patients to treatment with some ACTs [23–25], the actual drivers of these reported delayed parasite clearance in the region remain yet to be unraveled. Therefore, it is logical to suggest that delayed parasite clearance following artemisinin use in this region may be driven by other genetic markers different from the already established ones in the Southeast Asian region.

Most reports have described all mutations within the N-terminal domain including K189T mutation as having no *in-vitro* or clinical relevance to artemisinin resistance [40,41,45]. However, beyond the high prevalence of K189T mutation in this study, it also contributed significantly to slower parasite clearance in patients carrying parasites with such genotypes compared to the wild type. Therefore, the potential implications of the high prevalence of the K189T mutation in *Pf*kelch13 gene in the African population and its significant correlation with longer parasite clearance time in this study cannot be overlooked. It is therefore hypothesized that *Pf*kelch13 K189T mutation may play an unidentified but significant role that could influence artemisinin resistance. Furthermore, considering the occurrence of another change at the same position 189 (K189N), we postulate that there are possibilities that activities around position 189 in *Pf*kelch13 gene may have either a direct influence or modulate downstream events on sensitive positions within the propeller domain along the gene script that contributes to artemisinin resistance. However, further studies are still required to better understand the actual role or significance of position 189 of *Pf*kelch13 gene on artemisinin resistance.

Apart from the K189T mutation in *Pf*kelch13, a change from arginine to lysine at position 255 of the *Pf*kelch13 gene (R255K) also correlated significantly with longer parasite clearance times in patients carrying parasites with such genotype (P = 0.002) in this study, although with a very low frequency. This mutation has also been reported earlier with low prevalence in Cameroun and Liberia (∼1%), French Guiana (0.4%), and in the Southeast Asian region without any association with delayed parasite clearance [42,46,47].

Although none of the mutations within the N-terminal of *Pf*kelch13 gene has been found relevant to artemisinin resistance to date *in vitro*, more single nucleotide polymorphisms within this domain are increasingly being identified with prominent roles in artemisinin resistance. For instance, E252Q change within this domain was reported to confer artemisinin resistance in the Thai Western Border as well as the Northwest Thailand-Myanmar border [7,44]. This was inferred from its strong correlation with reduced parasites’ susceptibility to artemisinin and its increasing prevalence in the region over the years. This evidence from the previous studies and the current one partially justifies the claim that mutations within the N-terminal domain of *Pf*kelch13 may have potential relevance and contributions to artemisinin resistance. In addition, possibilities of geographical variations in genetic drivers of artemisinin resistance cannot be overruled as well as suspicion of an imminent emergence of artemisinin resistance in Nigeria.

There are sparse reports on the K189T mutation and other mutations within the N-terminal domain of *Pf*kelch13 in Nigeria because most studies on *Pf*kelch13 analysis in this region focused exclusively on the β-propeller domain, neglecting other domains [26–29]. Therefore, continuous extensive molecular surveillance is needed in Nigeria to provide consolidated and robust data on mutations in the domains of *Pf*kelch13 in the population.

Furthermore, an observation of a K189T *Pf*kelch13 mutation in a retrospective sample is notable. *Pf*kelch13 mutations were first reported as drivers of artemisinin resistance in 2014 six years after the first clinical report of artemisinin resistance [12]. In this study, concrete assertions cannot be made on the parasite’s genomic status relative to artemisinin resistance during the pre-ACT era since the identified mutation has not been qualified as a candidate or validated marker of artemisinin resistance. However, considering earlier thoughts on the potential relevance of K189T mutation in artemisinin resistance and previous findings of Oduola *et al* in 1992, this finding further corroborates the postulation that parasites may have innate potential for resistance to artemisinin before the actual introduction of ACTs [31]. This information on the pre-existence of resistance markers of drugs before their adoption provides an understanding of the pace of genetic evolution of molecular markers of resistance.

## Conclusion

There is currently no validated molecular evidence of artemisinin resistance in the study population. However, the high frequency of K189T mutation in the study population and its significant correlation with delayed parasite clearance time creates suspicion of an imminent emergence of artemisinin resistance in the region. The study also revealed an inherent potential of parasites to develop artemisinin resistance before the introduction of ACTs in Nigeria.

## Supporting information

Supporting information 1 S1 Raw Images. These are the raw images of the electrophoretic gel resolution after amplifications of fragments of *Pf*kelch13 propeller domain and the coiled-coil containing (CCC)/BTB-POZ domains. (PDF)

Supporting information 2 Pfkelch13 sequences. These are files of raw sequence data of both forward and reverse sequences of *Pf*kelch13 fragments. The files named KN1FW and KNIR represent sequences for forward and reverse reactions of the propeller domain respectively while those named KN2FW and KN2R represent sequences for forward and reverse reactions of the coiled-coil containing (CCC)/BTB-POZ domains respectively. (ZIP)
