# Supplementary material for: Polymorphisms in Pfkelch13 domains before and after the introduction of artemisinin-based combination therapy in Southwest Nigeria
Source: PLoS One. 2025 Mar 31;20(3):e0316479. doi: 10.1371/journal.pone.0316479 (PMC11957316; doi:10.1371/journal.pone.0316479)

Raw uncropped agarose gel image of Figure 2A obtained from gel documentation system

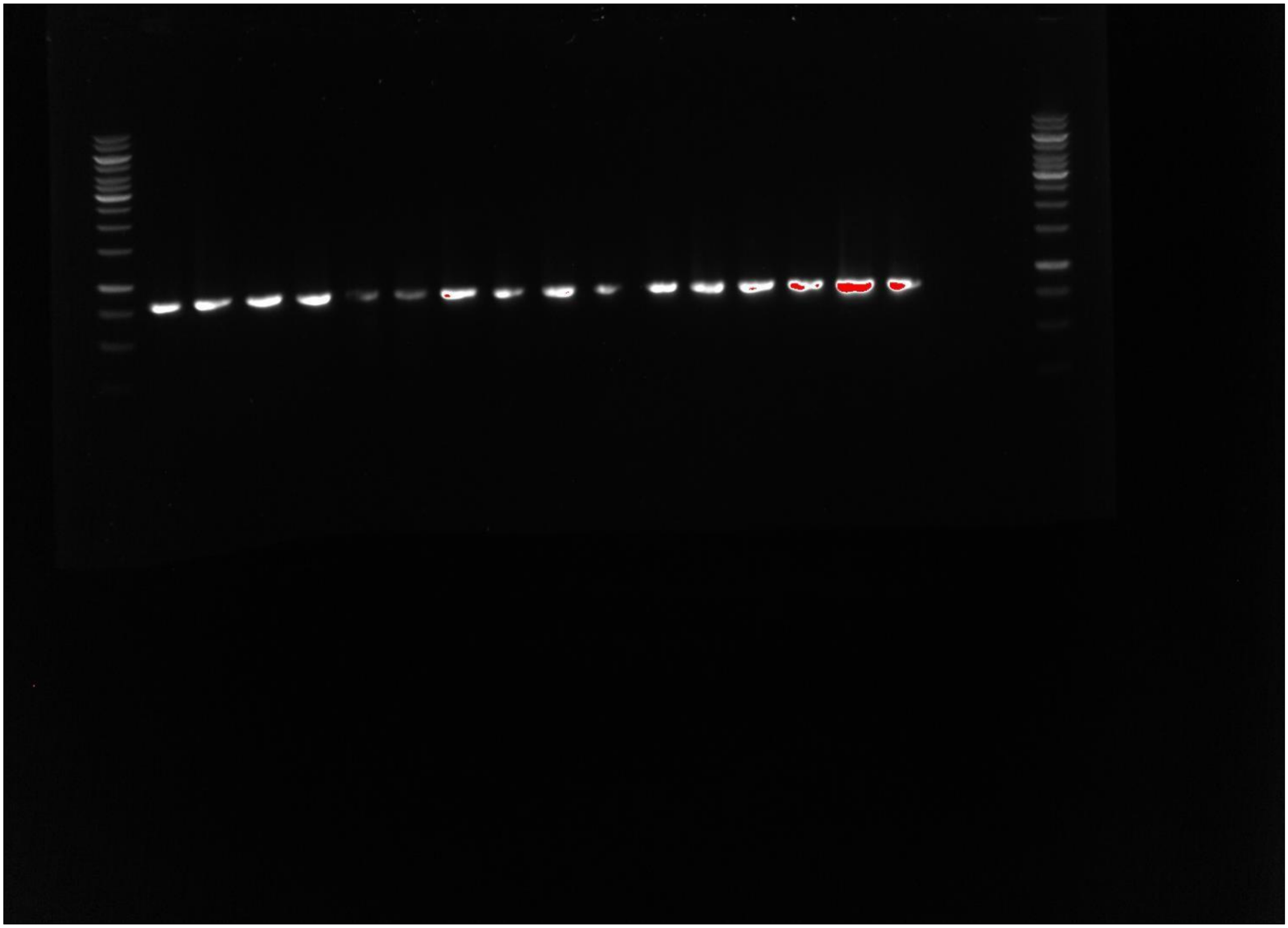

Raw uncropped agarose gel image of Figure 2B obtained from gel documentation system

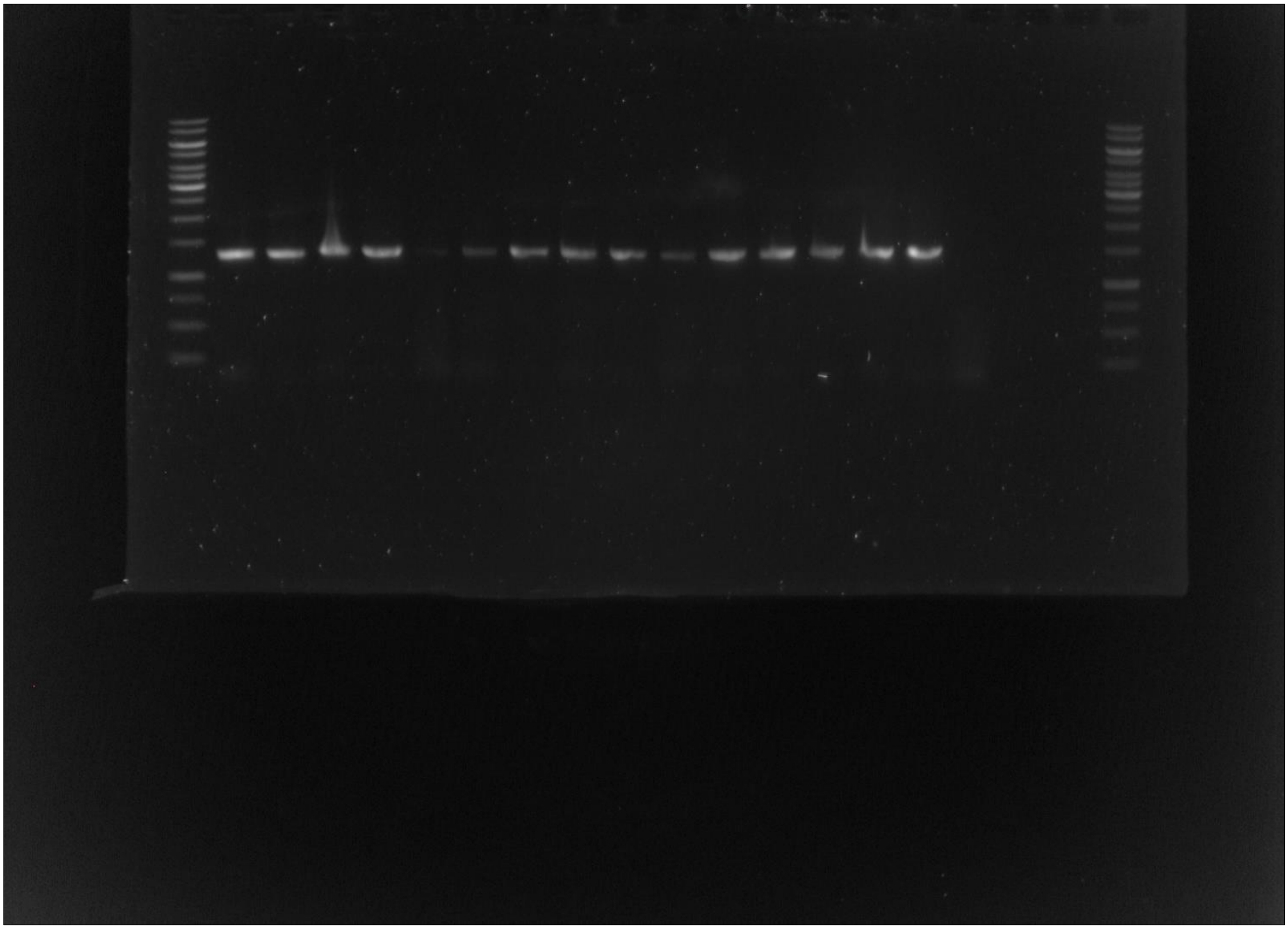

Supplement: Supporting information 1 — (PDF) [file pone.0316479.s001.pdf]
