# Supplementary material for: Polymorphisms in Pfkelch13 domains before and after the introduction of artemisinin-based combination therapy in Southwest Nigeria
Source: PLoS One. 2025 Mar 31;20(3):e0316479. doi: 10.1371/journal.pone.0316479 (PMC11957316; doi:10.1371/journal.pone.0316479)
Supplement: Supporting information 2 — (ZIP) [file pone.0316479.s002.zip › 002KN2F_PREMIX_Plate_KELCH1_F09.pdf]

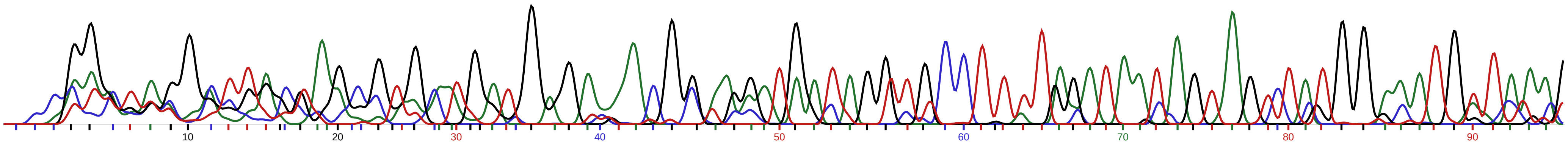

C C C G G C T A G G C T T T G C G A A G C C G G T G C A T G G A T C G A G A C T A C G G A G A A T G A T A G G T G C C T G T T T A G A T A A T A G T A G T C T A T G G A A A T G T T A A A A

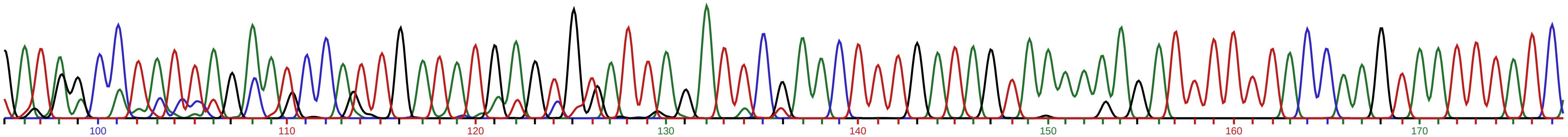

G A T A G C C T A T T A G A A T C C A T T G A T A T G A G T G T A T T A G A T T C G A A C T T T G A T A G T A A A A A G A T T T T T A C C A A G T A A T T T A T C

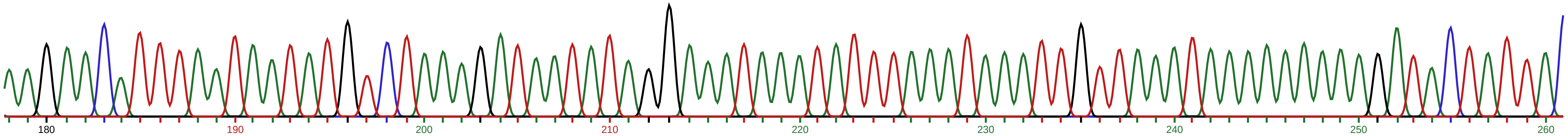

A A G A A C A T T T A A T A A T A T G T C T A A A G A T A A T A T A G G A A A T A A A T A T T T A A A T A A A T T G T T A A A T A A A A A A A A G A T A C T A T T A

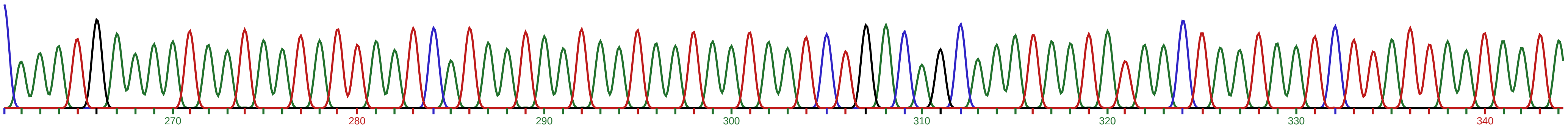

C A A A T G A A A A T A A T A A T A T T A A T C A T A A T A A T A A T A A T A A T A A T A A T C T G A C A G C A A A T A A T A T A A C T A A T A A T C T T A T T A A T A A T A

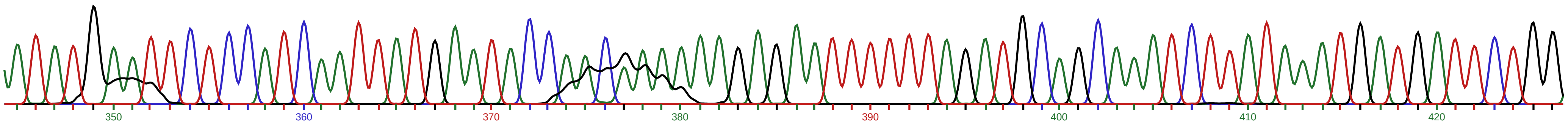

A T A T G A A T T C T C C A T C A A T T A T G A A T A C C A A C G A A A A A G A G A A T T T T T T A G A T G C A G C A A A T C T T A T A A A T G A T G A T T C T G G

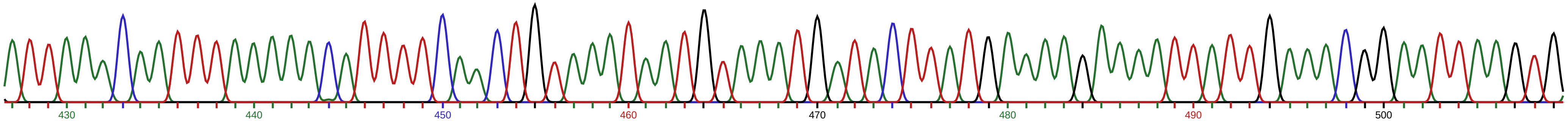

A T T A A A C A A T T T A A A A A C A T T T T C A A C T G T A A A T A A T G T A A A T G A T A C T T A T G A A A A G A A A A T T A T T G A A A C G G A A T T A A G T G

Samples: 13530
Bases: 1156
Average spacing: 12

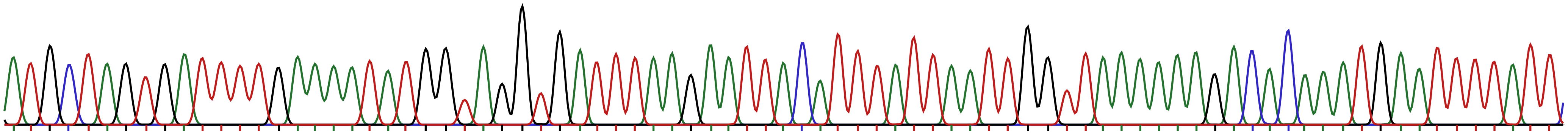

510 520 530 540 550 560 570 580 590

A T G C T A G T G A T T T T G A A A A T A T G G T A G G T G A T T T A A G A A T T A C A T T T A T T A A T T G G T T A A A A A A G A C A C A A A T G A A T T T T A T T

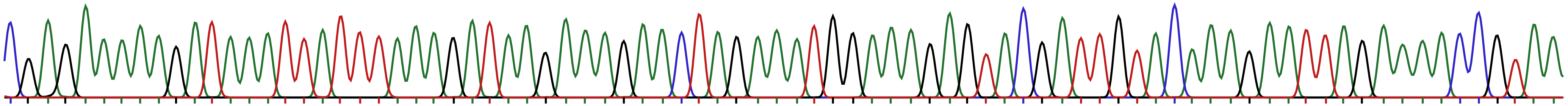

600 610 620 630 640 650 660 670

C G A G A A A A A G A T A A A T T A T T T A A A G A T A A G A A A G A A C T A G A A A T G G A A A G A G T A C G A T T G T A C A A A G A A T T A G A A A A C C G T A A

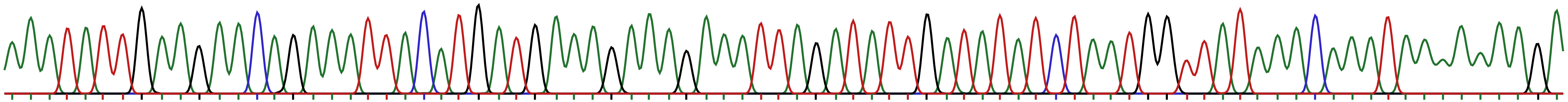

680 690 700 710 720 730 740 750

A A A T A T T G A A G A A C A G A A A T T A C A T G A T G A A A G A A A G A A A T T A G A T A T T G A T A T A T C T A A T G G T T A T A A A C A A A T A A A A A A A G A

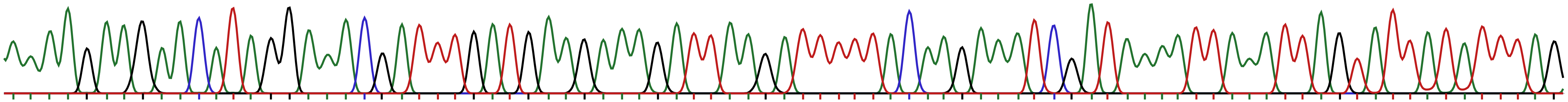

760 770 780 790 800 810 820 830 840

A A A A G A A G A A C A T A G G A A A C G A T T T G A T G A A G A A A G A T T A A G A T T T T T A C A A G A A A T C G A T A A A A T T A A A T T A G T A T T A T A T T T A G

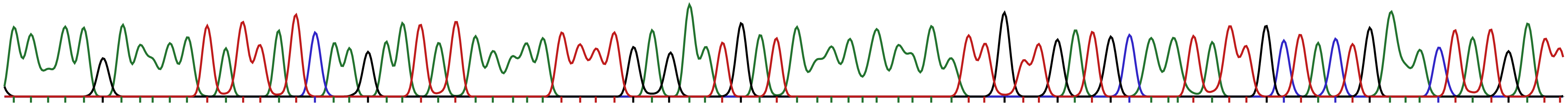

850 860 870 880 890 900 910 920 930

A A A A A G A AA A A T A T T A T C A A G A A T A T A A AAA T T T T G A G A A T G A T A AAAA AAA A T T G T T G A T G C A AA T A T T G C T A C T G A A A C T A T G A T T

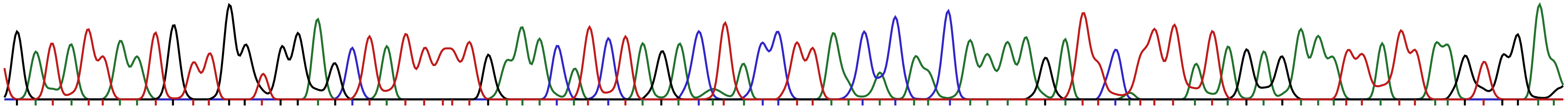

940 950 960 970 980 990 1000 1010 1020

G A T A T T A A T G T T G G T G G A G C T A T T TT T G A A A C A T C T A G A CAT A C C T T A A C A C A A C A A A A G A T T CATT T A T A G A G A A A T T A T T A A G T G G A A

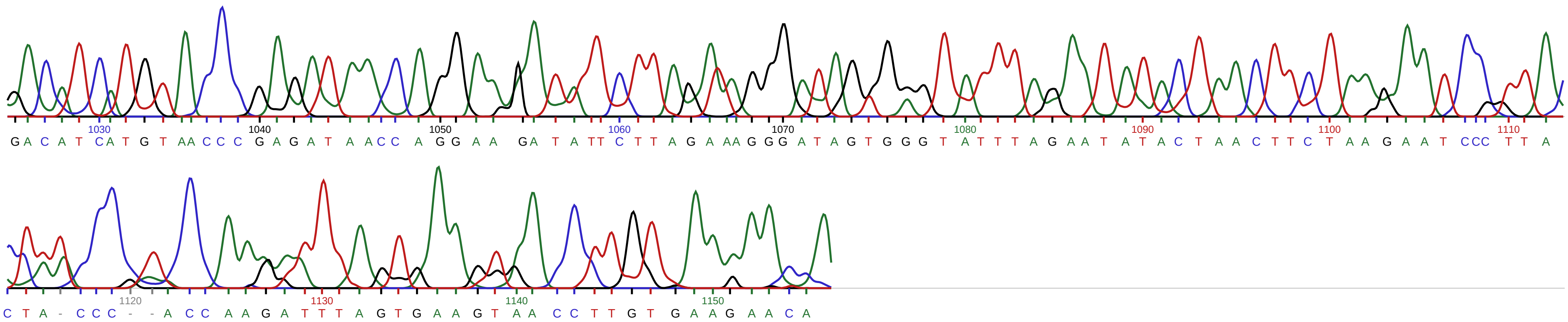
1030 1040 1050 1060 1070 1080 1090 1100 1110
G A C A T C A T G T A A C C C G A G A T A A C C A G G A A G A T A T T C T T A G A A A G G G A T A G T G G G T A T T T A G A A T A T A C T A A C T T C T A A G A A T C C C T T A
1120 1130 1140 1150
C T A - C C C - - A C C A A G A T T T A G T G A A G T A A C C T T G T G A A G A A C A
