# Supplementary material for: Polymorphisms in Pfkelch13 domains before and after the introduction of artemisinin-based combination therapy in Southwest Nigeria
Source: PLoS One. 2025 Mar 31;20(3):e0316479. doi: 10.1371/journal.pone.0316479 (PMC11957316; doi:10.1371/journal.pone.0316479)
Supplement: Supporting information 2 — (ZIP) [file pone.0316479.s002.zip › 008KN1R_PREMIX_Plate_KELCH1_B02.pdf]

Samples: 13815
Bases: 815
Average spacing: 17

10 20 30 40 50 60 70

- - - - - - - - - - - - - - - T - - A - T T - - C A T T T T C T C C T C C T G A A T T A T A - A A G A A T G T G A C A A T G - G G C A C - - - - - -

80 90 100 110 120 130 140 150

- - - - - - - T T T T T C T C T G G T A C A C C A T T T A G A A A T T - - - - - T T - - - T T A A A T G G - T G A T A T T G T T C A A C G G A A T C T A A T A T G T T

160 170 180 190 200 210 220 230 240

A T G T T C A T T A T C A A T A C C T C C A A C A A C A T A T A T T T G A T T A A G G T A A T T A A A A G C T G C T C C T G A A C T T C T A G C T T C T A A T A A G G

250 260 270 280 290 300 310 320

C A T A T G G A A A T T G T T C C C A T T T A T T C A T T T T T T C T T C A T A T A C T T C A A T A G A A T T T A A T C T C T C A C C A T G A G T T C C A C C A A T G A

330 340 350 360 370 380 390 400

C A T A A A T T T T A T T A T C A A A A G C A A C A C A C A T A G C T G A T G A T C T A G G G G T A T T C A A A G G T G C C A C C T C T A C C C A T G C T T T C A T A

410 420 430 440 450 460 470 480 490

C G A T G A T C A T A T G T T T T T A C A T T C G G T A T A A T A G A A G A G C C A T C A T A T C C C C A A T A C A A T A A A T T C T A C C A T T T G A C G T A A C

Samples: 13815
Bases: 815
Average spacing: 17

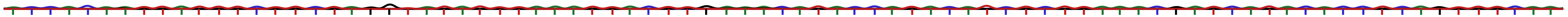

500 510 520 530 540 550 560 570

A C C A C A A T T A T T T C T T C T A G G T A T A T T T A A A T T A C T T G A A A C A T A C C A T A C A T C T C T T A A A C G A T C A T A C A C C T C A G T T T C A A

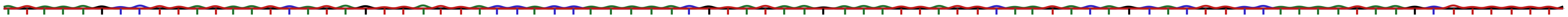

580 590 600 610 620 630 640 650

A T A A A G C C T T A T A A T C A T A G T T A T T A C C A C C A A A A A C G T A T A A G A A A T T A T T C A A T A C A G C A C T T C C A A A A T A A G C T T T T T T

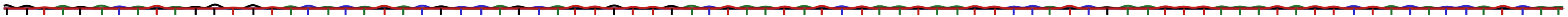

660 670 680 690 700 710 720 730

G G T A G A C A T A G G T G T A C A C A T A C G C C A G C A T T G T T G A C T A A T A T C T A A T A A T T C C A T C G A A T T T A A A T A T T C T A C A C C A T C A A

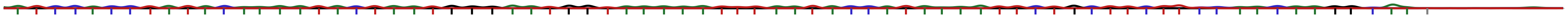

740 750 760 770 780 790 800 810

A T C C A C C T A T A C A A A A T A C T A A T G G G A A T G G T A A A A A T T T A A T A C C A T A A A A T T C T G C T T C T T C C A A C A A G G C A A -

Samples:	13815
Bases:	815
Average spacing:	17
