# Supplementary material for: Polymorphisms in Pfkelch13 domains before and after the introduction of artemisinin-based combination therapy in Southwest Nigeria
Source: PLoS One. 2025 Mar 31;20(3):e0316479. doi: 10.1371/journal.pone.0316479 (PMC11957316; doi:10.1371/journal.pone.0316479)
Supplement: Supporting information 2 — (ZIP) [file pone.0316479.s002.zip › 017KN2F_PREMIX_Plate_KELCH1_G06.pdf]

Samples: 14221
Bases: 871
Average spacing: 17

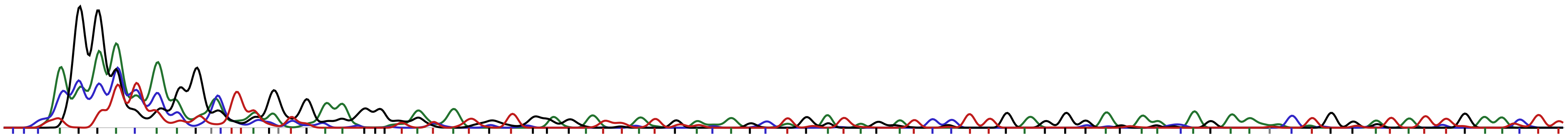

10 20 30 40 50 60 70 80

C C C A G G A C A A G - C T T A G - T G A A G G G T A T A T G T G G G A T T A T G A C A G C T G A T A G A T C C T T G A G G G A G A A C A G A A - C T G G A T A T T G A A C T T

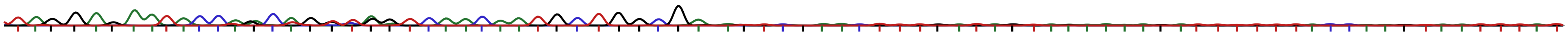

90 100 110 120 130 140 150 160 170

T A G G A G A A T A C C A G C A G G T G G T C A A C A A T G C T G G C G A A G T C G A A C T T T G A T A G T A A A A A A G A T T T T T T A C C A A G T A A T T T A T

180 190 200 210 220 230 240 250

C A A G A A C A T T T A A T A A T A T G T C T A A A G A T A A T A T A G G A A A T A A A T A T T T A A A T A A A T T G T T A A A T A A A A A A A A A G A T A C T A T T

260 270 280 290 300 310 320 330

A C A A A T G A A A A T A A T A A T A T T A A T C A T A A T A A T A A T A A T A A T A A T C T G A C A G C A A A T A A T A T A A C T A A T A A T C T T A T T A A T A A

340 350 360 370 380 390 400 410

T A A T A T G A A T T C T C C A T C A A T T A T G A A T A C C A A C A A A A A A G A G A A T T T T T T A G A T G C A G C A A A T C T T A T A A A T G A T G A T T C T G

420 430 440 450 460 470 480 490 500

G A T T A A A C A A T T T A A A A A C A T T T T C A A C T G T A A A T A A T G T A A A T G A T A C T T A T G A A A A G A A A A T T A T T G A A A C G G A A T T A A G T

Samples: 14221
Bases: 871
Average spacing: 17

510 520 530 540 550 560 570 580

G A T G C T A G T G A T T T T G A A A A T A T G G T A G G T G A T T T A A G A A T T A C A T T T A T T A A T T G G T T A A A A A A C A C A C A A A T G A A T T T T A

590 600 610 620 630 640 650 660

T T C C A G A A A A A G A T A A A T T A T T T A A A G A T A A G A A A G A A C T A G A A A T G G A A A G A G T A C C A T T G T A C A A A G A A T T A T A A A A C C G T A

670 680 690 700 710 720 730 740 750

A A A A T A T T G A A G A A C A G A A A T T A C A T G A T G A A A G A A A G A A A T T A T A T A T T G A T A T A T C T A A T G G T T A T A A A C A A A T A A A C A T

760 770 780 790 800 810 820 830

A T - A C A C A G - A - A A - C - T A G G A A A C G A T T T G A T G A A C A A A A A T T A C T A T C T T T C C - - C - A T C - A T A A A A T T A A A T T A - T A T T A

840 850 860 870

T A T T T - - - - A A - - A - - A T A T - - T C G G C A A A T A A A A - -

Samples: 14221
Bases: 871
Average spacing: 17
