# Supplementary material for: Polymorphisms in Pfkelch13 domains before and after the introduction of artemisinin-based combination therapy in Southwest Nigeria
Source: PLoS One. 2025 Mar 31;20(3):e0316479. doi: 10.1371/journal.pone.0316479 (PMC11957316; doi:10.1371/journal.pone.0316479)

Samples: 13582
Bases: 823
Average spacing: 17

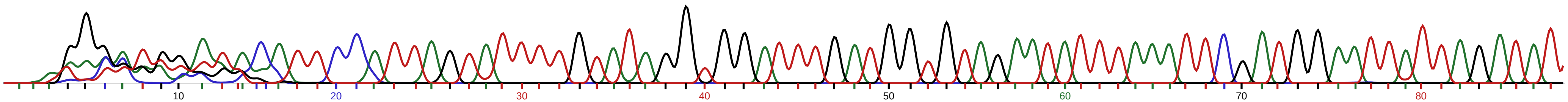

10 20 30 40 50 60 70 80

A A A G G C A T G G A T T A C C A T T C C A T T A G T A T T T T G T A T A G G T G G A T T G A T G G T G T A G A A T A T T T A A A T T C G A T G G A A T T A T T A G A T A T

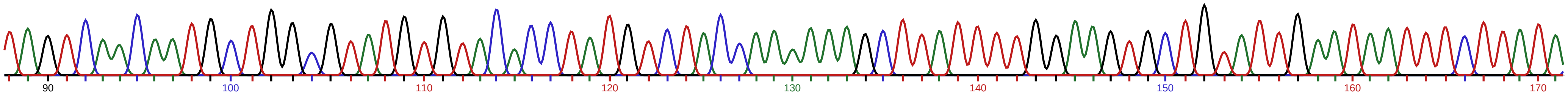

90 100 110 120 130 140 150 160 170

T A G T C A A C A A T G C T G G C G T A T G T G T A C A C C T A T G T C T A C C A A A A A A G C T T A T T T T G G A A G T G C T G T A T T G A A T A A T T T C T T A T A

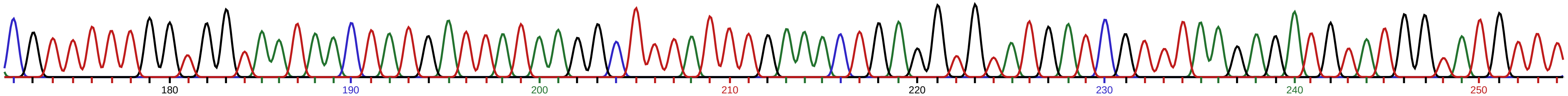

180 190 200 210 220 230 240 250

C G T T T T T G G T G G T A A T A A C T A T G A T T A T A A G G C T T T A T T T G A A A C T G A G G T G T A T G A T C G T T T A A G A G A T G T A T G G T A T G T T T

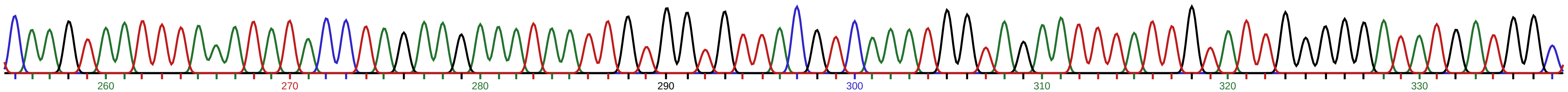

260 270 280 290 300 310 320 330

C A A G T A A T T T A A A T A T A C C T A G A A G A A A T A A T T G T G G T G T T A C G T C A A A T G G T A G A A T T T A T T G T A T T G G G G G A T A T G A T G G C

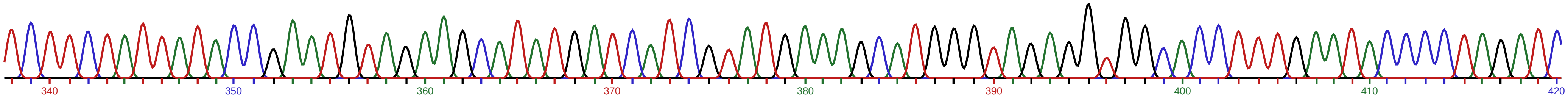

340 350 360 370 380 390 400 410 420

T C T T C T A T T A T A C C G A A T G T A G A A G C A T A T G A T C A T C G T A T G A A A G C A T G G G T A G A G G T G G C A C C T T T G A A T A C C C C T A G A T C

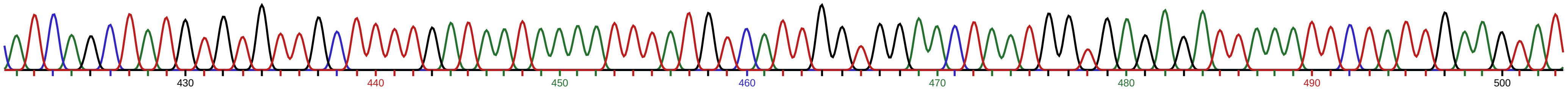

430 440 450 460 470 480 490 500

A T C A G C T A T G T G T G T T G C T T T T G A T A A T A A A A T T T A T G T C A T T G G T G G A A C T A A T G G T G A G A G A T T A A A T T C T A T T G A A G T A T

Samples: 13582
Bases: 823
Average spacing: 17



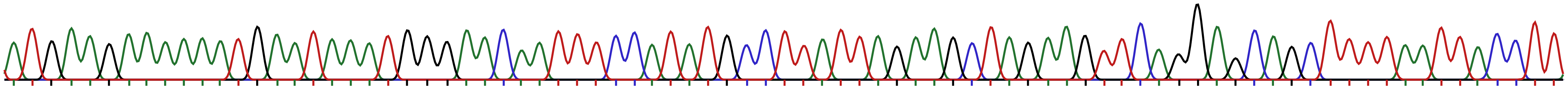

510 520 530 540 550 560 570 580

A T G A A G A A A A A A T G A A T A A A T G G G A A C A A T T T C C A T A T G C C T T A T T A G A A G C T A G A A G T T C A G G A G C A G C T T T T A A T T A C C T T

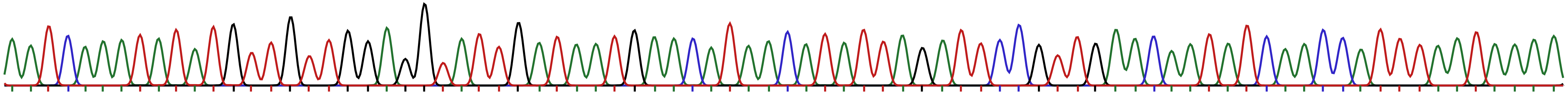

590 600 610 620 630 640 650 660

A A T C A A A T A T A T G T T G T T G G A G G T A T T G A T A A T G A A C A T A A C A T A T T A G A T T C C G T T G A A C A A T A T C A A C C A T T T A A T A A A A

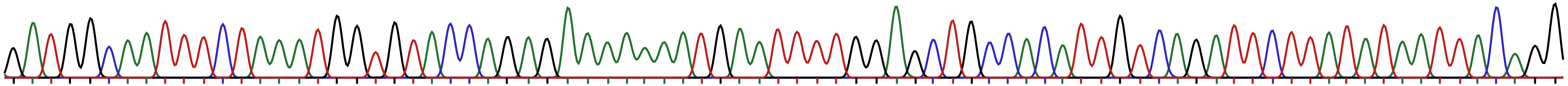

670 680 690 700 710 720 730 740 750

G A T G G C A A T T T C T A A A T G G T G T A C C A G A G A A A A A A T G A A T T T T G G A G C T G C C A C A T T G T C A G A T T C T T T A T A T A A T T A C A G G

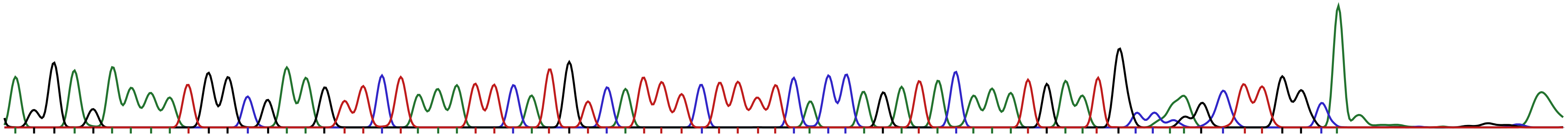

760 770 780 790 800 810 820

A G G A G A A A A T G G C G A A G T T C T A A A T T C A T G T C A T T T C T T T T C A C C A G A T A C A A A T G A A T G C C A A G C T T G G C A

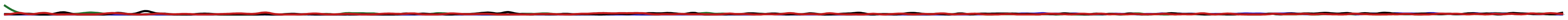

Samples: 13582
Bases: 823
Average spacing: 17

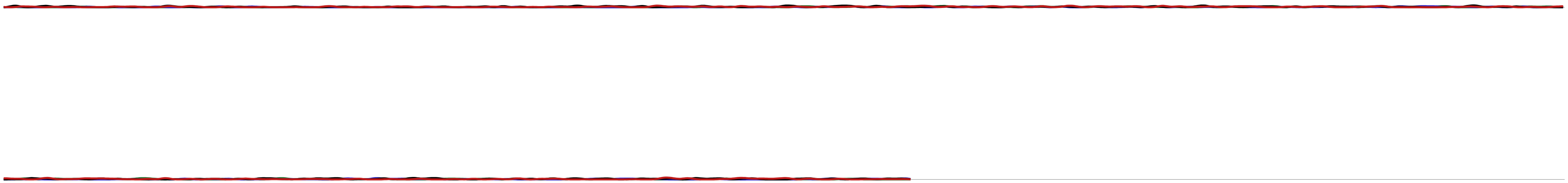

Supplement: Supporting information 2 — (ZIP) [file pone.0316479.s002.zip › 017KNIFW_PREMIX_Plate_CORKELCH_E04.pdf]
