# Supplementary material for: Polymorphisms in Pfkelch13 domains before and after the introduction of artemisinin-based combination therapy in Southwest Nigeria
Source: PLoS One. 2025 Mar 31;20(3):e0316479. doi: 10.1371/journal.pone.0316479 (PMC11957316; doi:10.1371/journal.pone.0316479)
Supplement: Supporting information 2 — (ZIP) [file pone.0316479.s002.zip › 019KN2F_PREMIX_Plate_KELCH1_G08.pdf]

Samples: 13908
Bases: 1173
Average spacing: 12

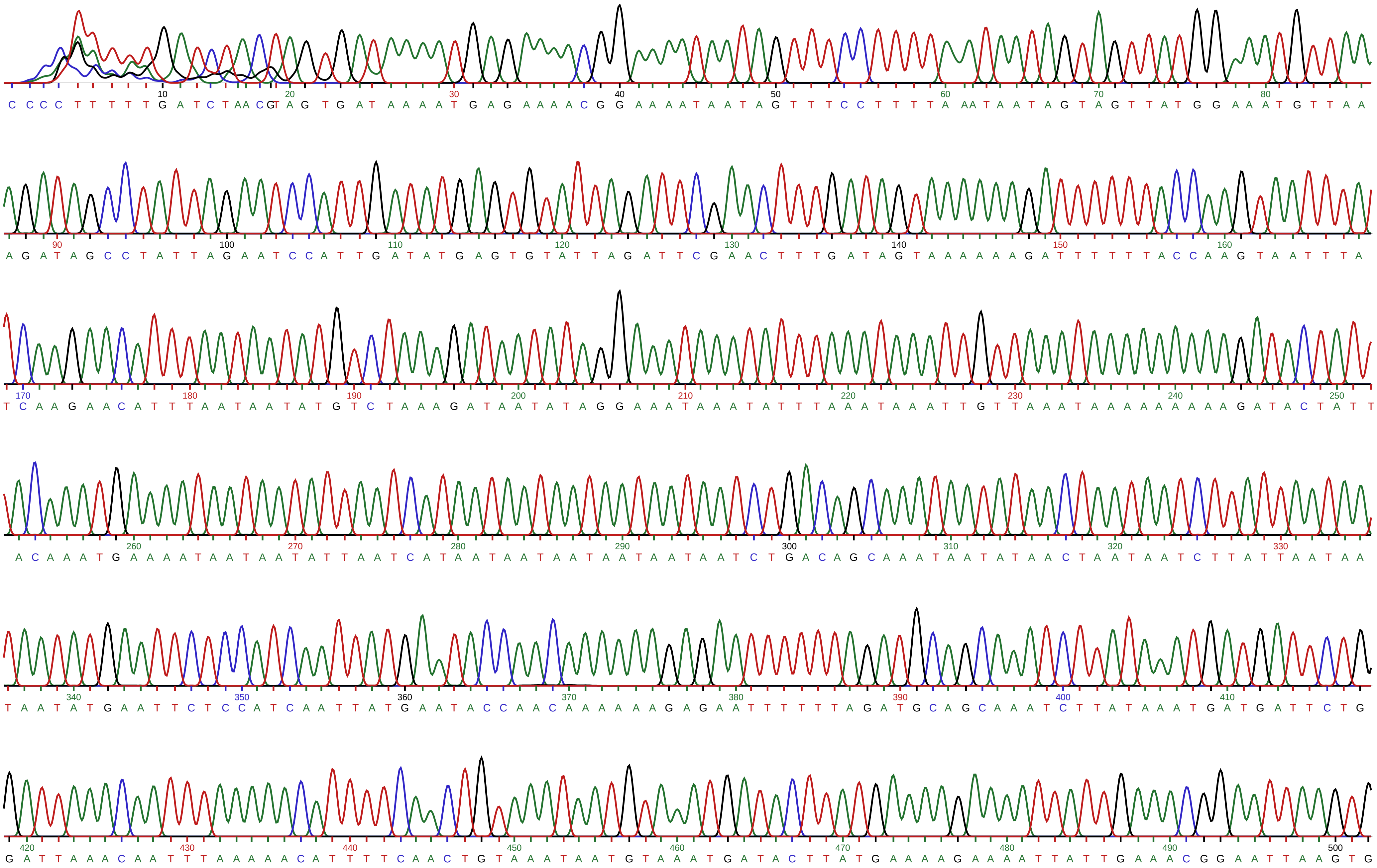

Samples: 13908
Bases: 1173
Average spacing: 12

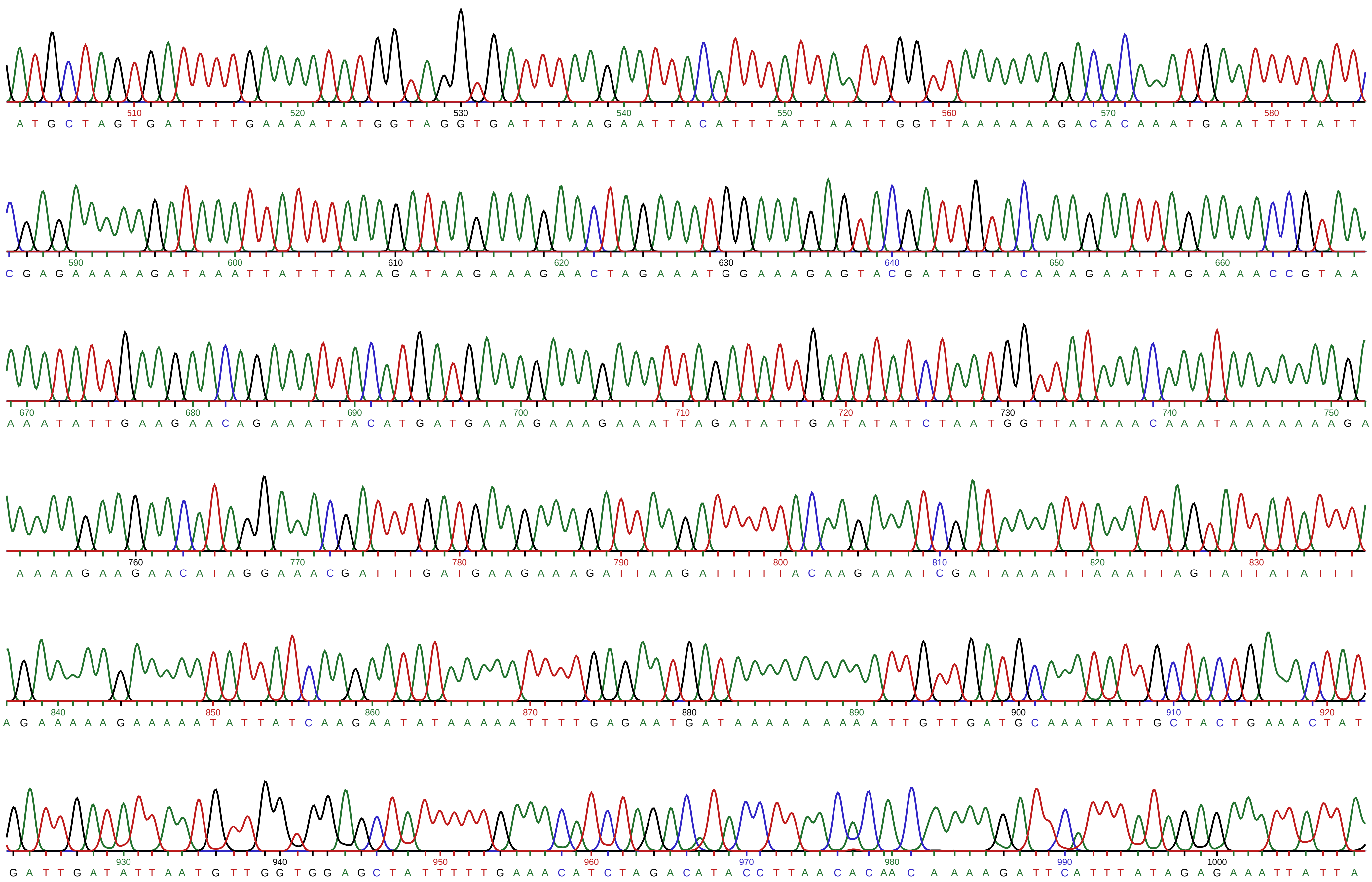

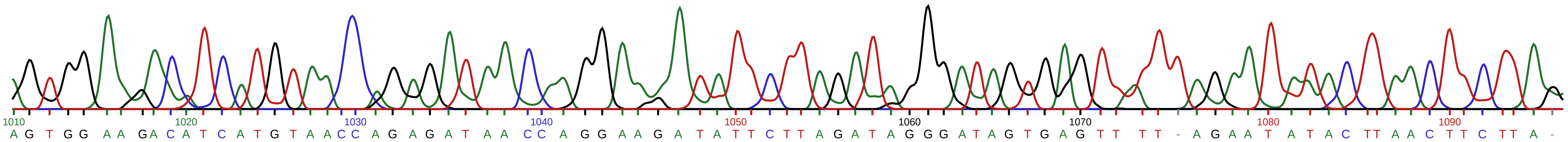
1010 1020 1030 1040 1050 1060 1070 1080 1090
A G T G G A A G A C A T C A T G T A A C C A G A G A T A A C C A G G A A G A T A T T C T T A G A T A G G G A T A G T G A G T T T T - A G A A T A T A C T T A A C T T C T T A -

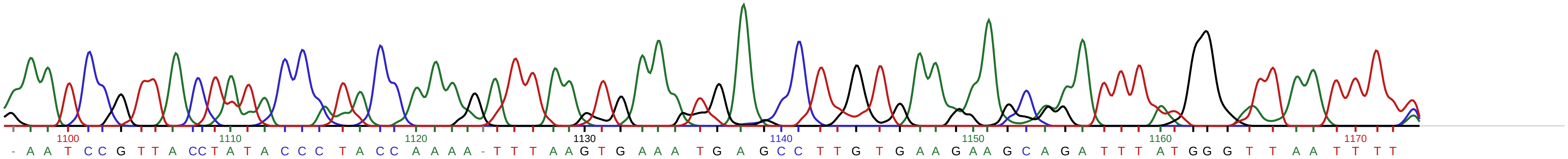
1100 1110 1120 1130 1140 1150 1160 1170
- A A T C C G T T A C C T A T A C C C T A C C A A A A - T T T A A G T G A A A T G A G C C T T G T G A A G A A G C A G A T T T A T G G G T T A A T T T T
