# Supplementary material for: Polymorphisms in Pfkelch13 domains before and after the introduction of artemisinin-based combination therapy in Southwest Nigeria
Source: PLoS One. 2025 Mar 31;20(3):e0316479. doi: 10.1371/journal.pone.0316479 (PMC11957316; doi:10.1371/journal.pone.0316479)

Samples: 14826
Bases: 642
Average spacing: 24

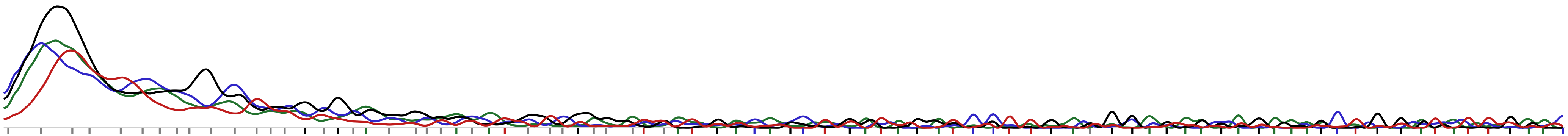

10 20 30 40 50 60 70 80

- - - - - - - - - - - - - - - - G - G - A - - - - A - A T - - - G - - - T - A T G A C A G C T G A T A G A T C C T T G A - G G A G A A G A G A A G C T G G A T A T T G A A C

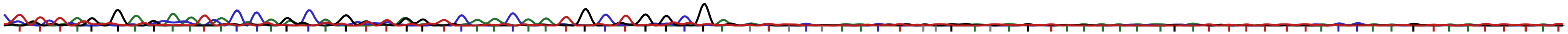

90 100 110 120 130 140 150 160

T T T A G G A G A A T A C C A G C A G T T G G T C A A C A A T G C T G G C G A - T - C - A A C T - - G A - A G T A A A A A A G A T T T T T T A C C A A G T A A T T T A T

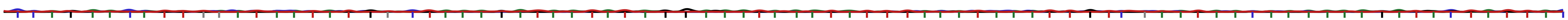

170 180 190 200 210 220 230 240 250

C C A G A A C A T T - - A T A A T A T G - C T A A A G A T A A T A T A G G A A A T A A A T A T T T A A A T A A A T T G T C - A A T A A C A A A A A A G A T A C T A T T A C

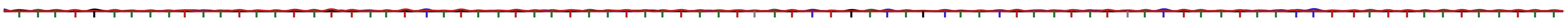

260 270 280 290 300 310 320 330

A A A T G A A A A T A A T A A T A T T A A T C A T A A T A A T A A T A A T A A T - A T C T G A C A G C A A C T A A T A T - A C T A A T A A C C T T A T T A A T A A T A A

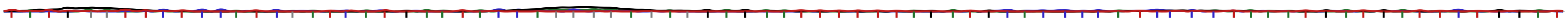

340 350 360 370 380 390 400 410 420

T A T G - - T T C T C C A T C - A T C A T G A A T A C C A - - - - - A - A - G A G A A T T T T T T A G A T G C A C C C C A T C C C C T A A A T G A T G A T T C T G G A T

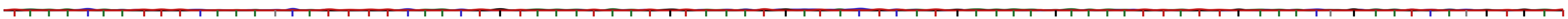

430 440 450 460 470 480 490 500

T A A A C A A T T T C A A A - C A T T T T C A A C T G T A A A T A A T G T A A A T G A T A C T C A T G A A A A G A A A A T T A T T G A A A C - G A A T C A - G T G A T

Samples: 14826
Bases: 642
Average spacing: 24

510 520 530 540 550 560 570 580

G C T A T T G A T T T T G A A A A T A T G G T A G G C G A T T T A A C A A T T A C A T T T A T T A A T T G G T T A A A - - - C A C A C - A A T G A A T T T T A T T

590 600 610 620 630 640

C T A - A A A A A A - A T A A - - - - - - - - A A - A T A A - A A A A A A C T A T A A A T G G A - A C A - T A C

Samples:	14826
Bases:	642
Average spacing:	24

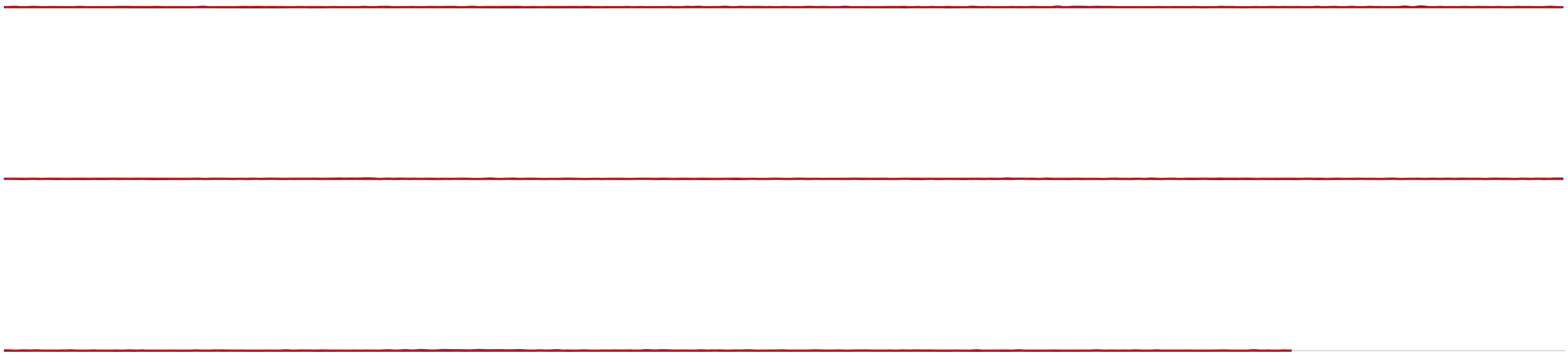

Supplement: Supporting information 2 — (ZIP) [file pone.0316479.s002.zip › 022KN2F_PREMIX_Plate_KELCH1_G09.pdf]
