# Supplementary material for: Polymorphisms in Pfkelch13 domains before and after the introduction of artemisinin-based combination therapy in Southwest Nigeria
Source: PLoS One. 2025 Mar 31;20(3):e0316479. doi: 10.1371/journal.pone.0316479 (PMC11957316; doi:10.1371/journal.pone.0316479)

Samples: 13173
Bases: 822
Average spacing: 17

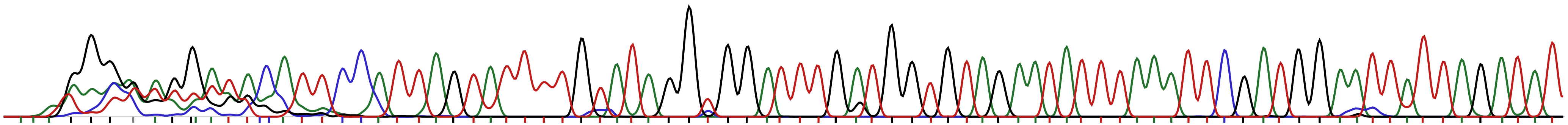

10 20 30 40 50 60 70 80

AAAGGG-AGGAATTCCATTCCATTAGTATTTTGTATAGGTGGATTTGATGGTGTAGAATATTTAAATTCGATGGAATTATTAGATAT

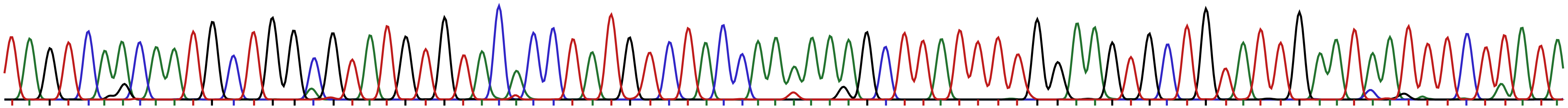

90 100 110 120 130 140 150 160 170

TAGTCAACAATGCTGGCGTATGTGTACACCTATGTCTACCAAAAAAGCTTATTTTGGAAGTGCTGTATTGAATAATTTCTTATA

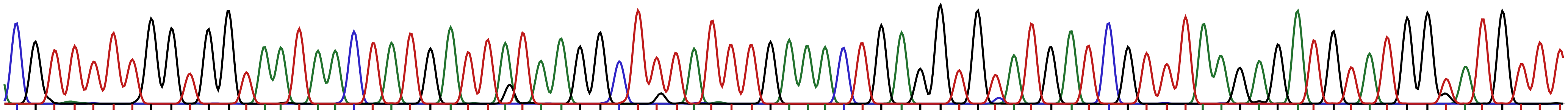

180 190 200 210 220 230 240 250

CGTTTTTGGTGGTAATAACTATGATTATAAGGCTTTATTTGAAACTGAGGTGTATGATCGTTTAAGAGATGTATGGTATGTTT

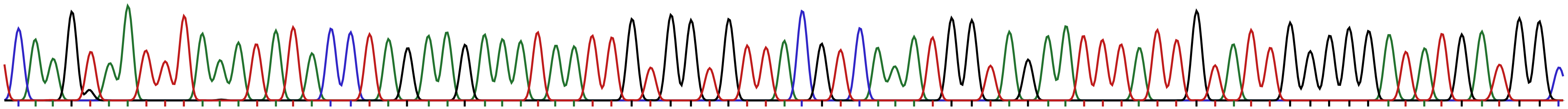

260 270 280 290 300 310 320 330

CAAGTAATTTAAATATACCTAGAAGAAATAATTGTGGTGTTACGTCAAATGGTAGAATTTATTGTATTGGGGGATATGATGGC

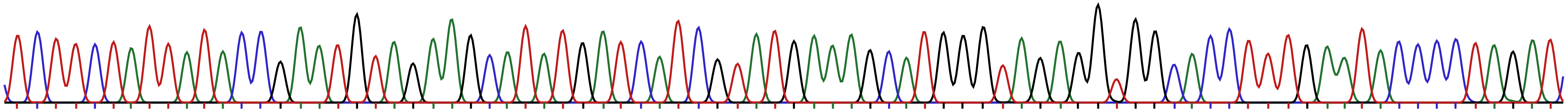

340 350 360 370 380 390 400 410

TCTTCTATTATACCGAATGTAGAAGCATATGATCATCGTATGAAAGCATGGGTAGAGGTGGCACCTTTGAATACCCCTAGAT

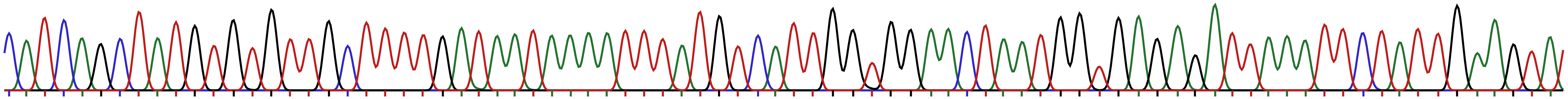

420 430 440 450 460 470 480 490 500

CATCAGCTATGTGTGTTGCTTTTGATAATAAAATTTATGTCATTGGTGGAACTAATGGTGAGAGATTAAATTCTATTGAAGTA

Samples: 13173
Bases: 822
Average spacing: 17

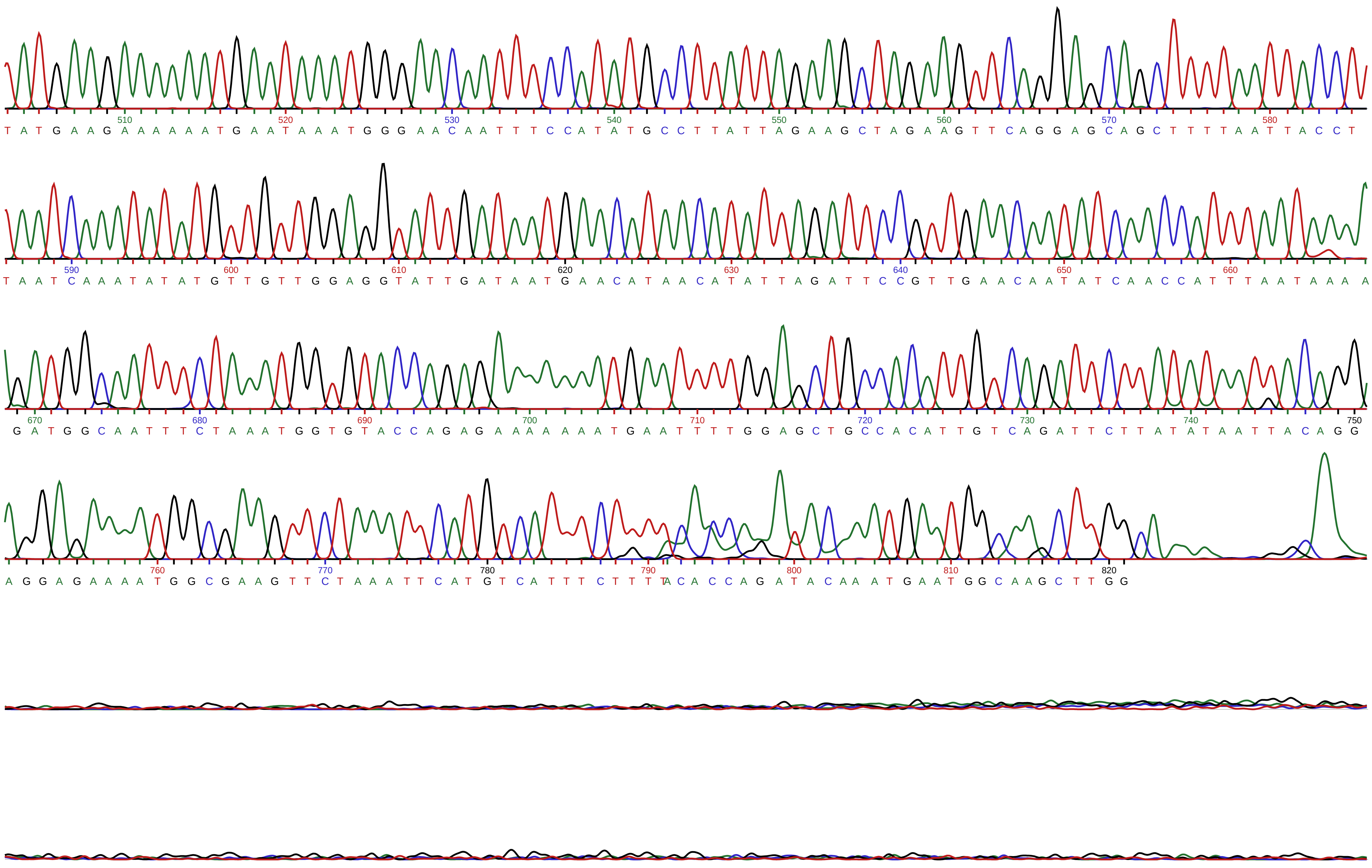

Samples: 13173
Bases: 822
Average spacing: 17

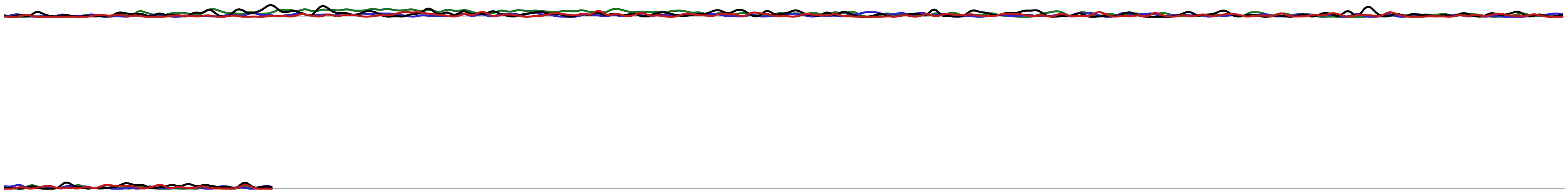

Supplement: Supporting information 2 — (ZIP) [file pone.0316479.s002.zip › 022KNIFW_PREMIX_Plate_CORKELCH_E07.pdf]
