# Supplementary material for: Polymorphisms in Pfkelch13 domains before and after the introduction of artemisinin-based combination therapy in Southwest Nigeria
Source: PLoS One. 2025 Mar 31;20(3):e0316479. doi: 10.1371/journal.pone.0316479 (PMC11957316; doi:10.1371/journal.pone.0316479)

Samples: 13490
Bases: 589
Average spacing: 23

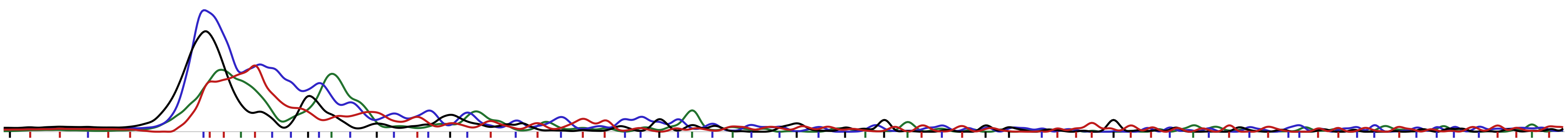

10 20 30 40 50 60 70

G T T C T T CT T A T C C GCA C G C TC G C T C T C T T CC G C A C A C C G C G A G A T C T G G C T T T G T T C A C T C T CC T T C C T C C C C T T A T

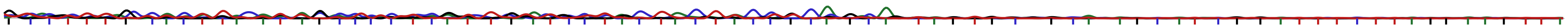

80 90 100 110 120 130 140 150

G C C T T T G C A C T T A A T A G C C C C T C A T T G A T C C T A C T A C T A C C G C A G C A T A G T G C G C A A G C A T C G G A T T T C T T A G G A A G T T A

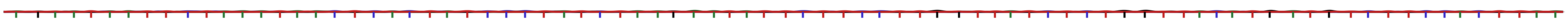

160 170 180 190 200 210 220 230 240

A G A A T A A T T C T A A A T A A C T C A C T A T C C C T A T C T A A G A A T A T T C T T C C T T G G T T A T C T C T G G T T A C A T G A T G T C T T C C A C T T A A

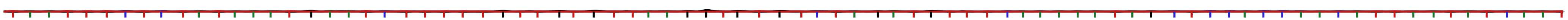

250 260 270 280 290 300 310 320

T A A T T T C T C T A T A A A T G A A T C T T T T T G T T G T G T T A A G G T G T G T C T A G A T G T T T C A A A A A T A G C T C C A C C A A C A T T A A T A T C A A T

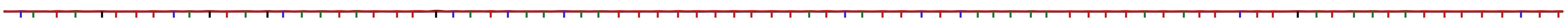

330 340 350 360 370 380 390 400

C A T A G T T T C A G T A G C A A T A T T T G C A T C A A C A A T T T T T T T T T T A T C A T T C T C A A A A A T T T T A T A T T C T T G A T A A T A T T T T T C T T

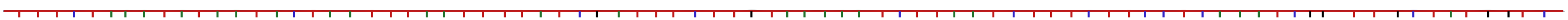

410 420 430 440 450 460 470 480

T T T C T A A A T A T A A T A C T A A T T T A A T T T T A T C G A T T T C T T G T A A A A A T C T T A A T C T T T C T T C C T C A A A T C G G T T G C T A T G G T C

Samples: 13490
Bases: 589
Average spacing: 23

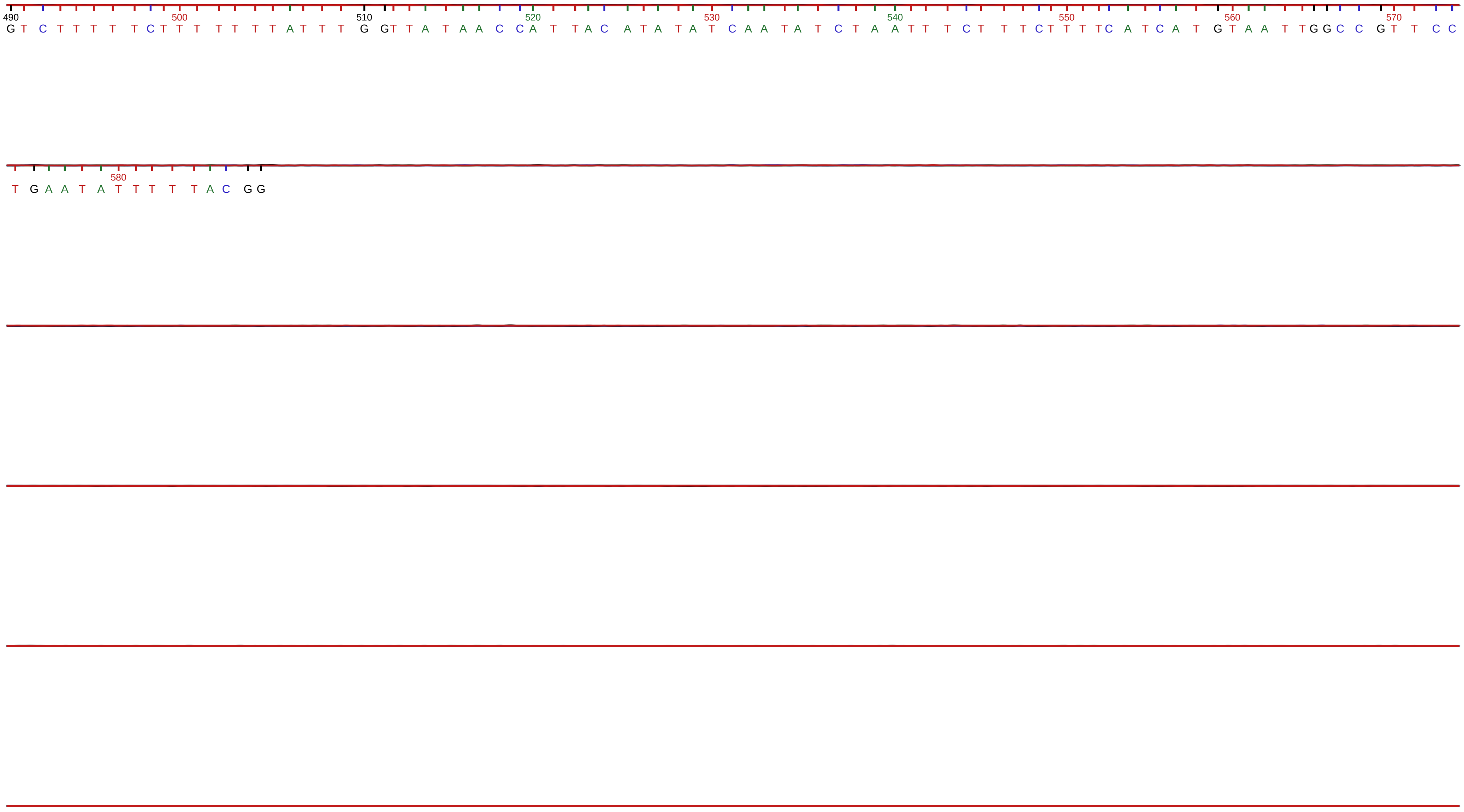

Samples:	13490
Bases:	589
Average spacing:	23

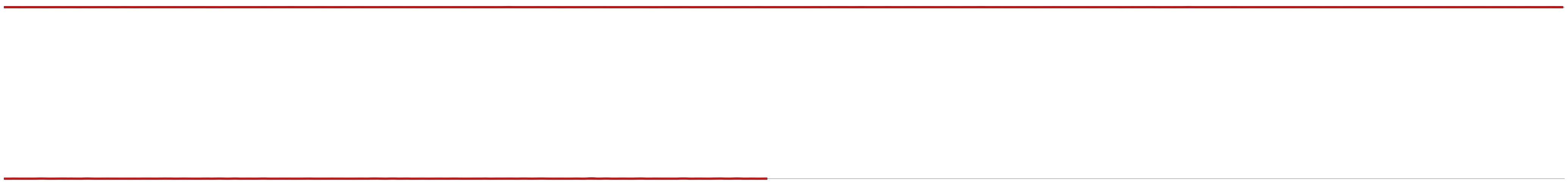

Supplement: Supporting information 2 — (ZIP) [file pone.0316479.s002.zip › 026KN2R_PREMIX_Plate_KELCH2_E01.pdf]
