# Supplementary material for: Polymorphisms in Pfkelch13 domains before and after the introduction of artemisinin-based combination therapy in Southwest Nigeria
Source: PLoS One. 2025 Mar 31;20(3):e0316479. doi: 10.1371/journal.pone.0316479 (PMC11957316; doi:10.1371/journal.pone.0316479)

Samples: 13143
Bases: 1106
Average spacing: 12

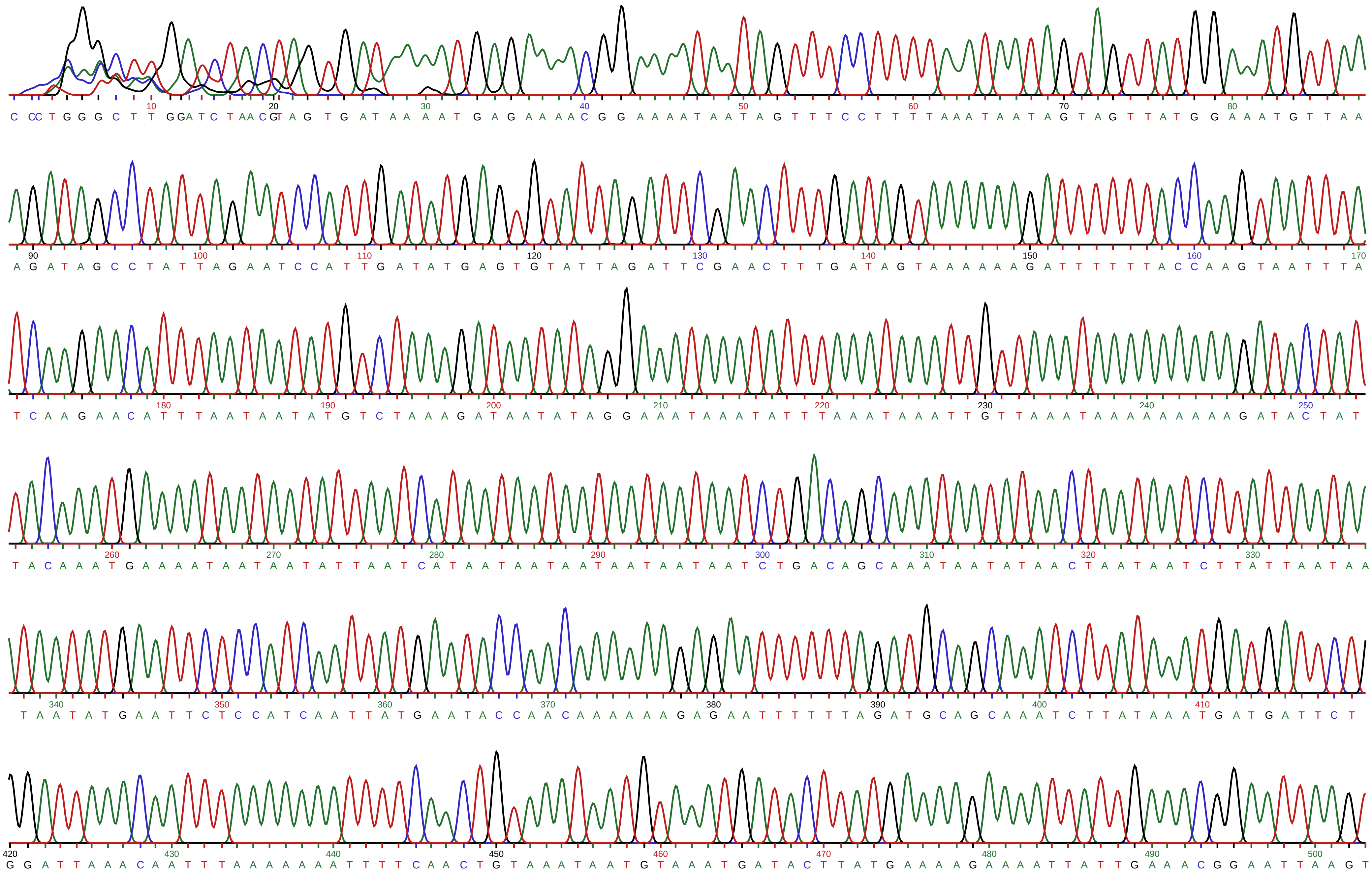

Samples: 13143
Bases: 1106
Average spacing: 12

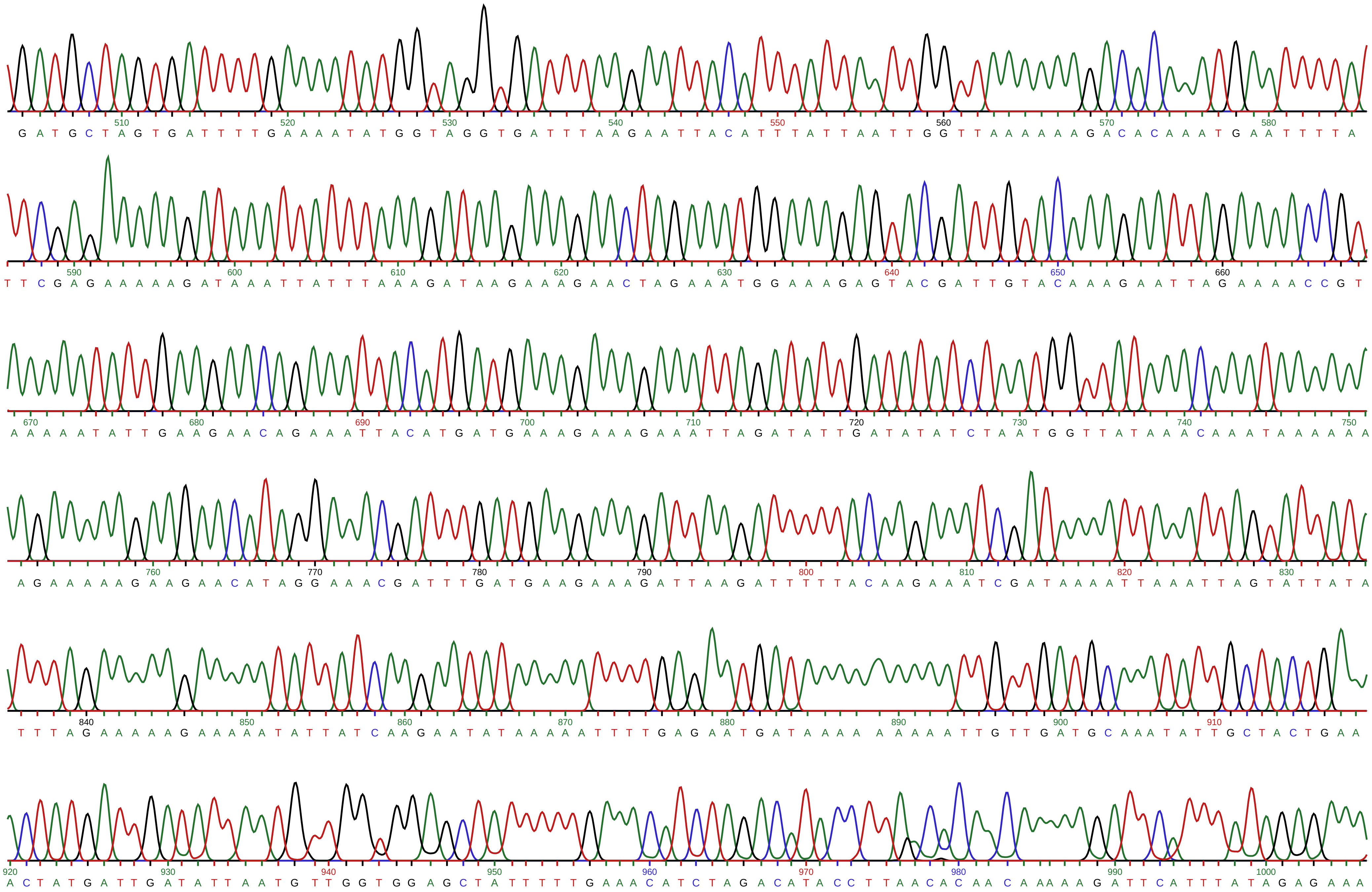

Samples: 13143
Bases: 1106
Average spacing: 12

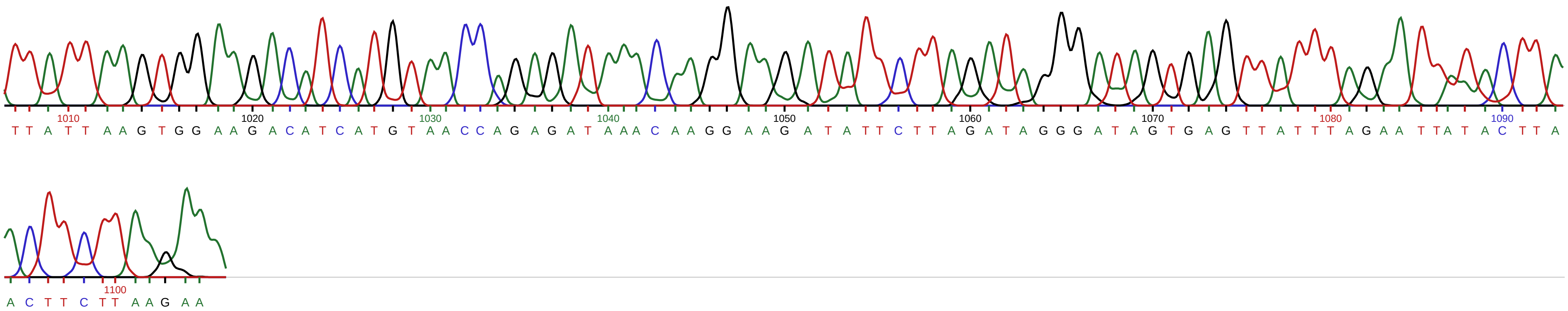

Supplement: Supporting information 2 — (ZIP) [file pone.0316479.s002.zip › 028KN2F_PREMIX_Plate_KELCH1_G12.pdf]
