# Supplementary figures and images for: Polymorphisms in Pfkelch13 domains before and after the introduction of artemisinin-based combination therapy in Southwest Nigeria
Source: PLoS One. 2025 Mar 31;20(3):e0316479. doi: 10.1371/journal.pone.0316479 (PMC11957316; doi:10.1371/journal.pone.0316479)

Samples: 12240  
Bases: 1025  
Average spacing: 12

Page: 1 / 3  
8/17/2022

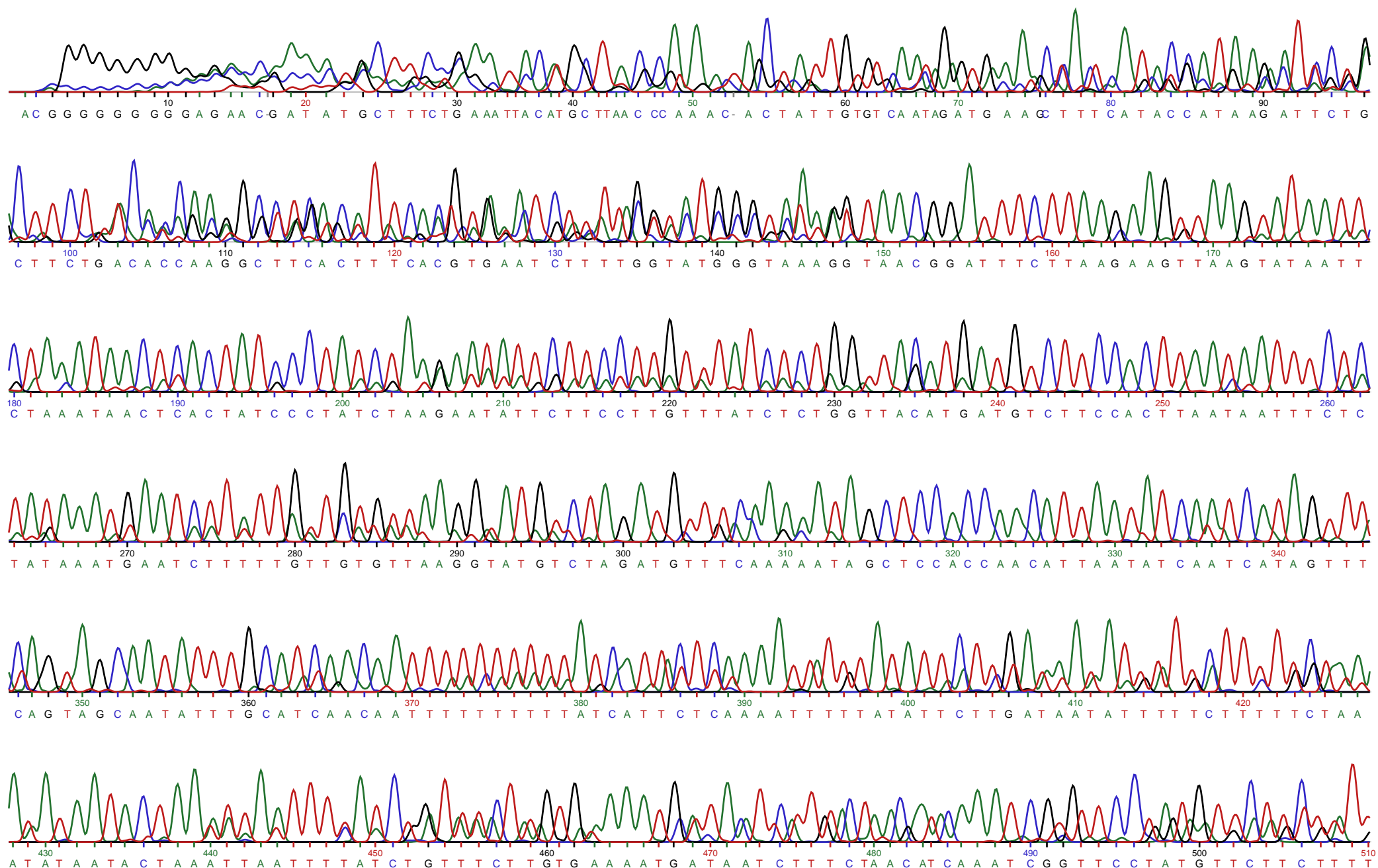

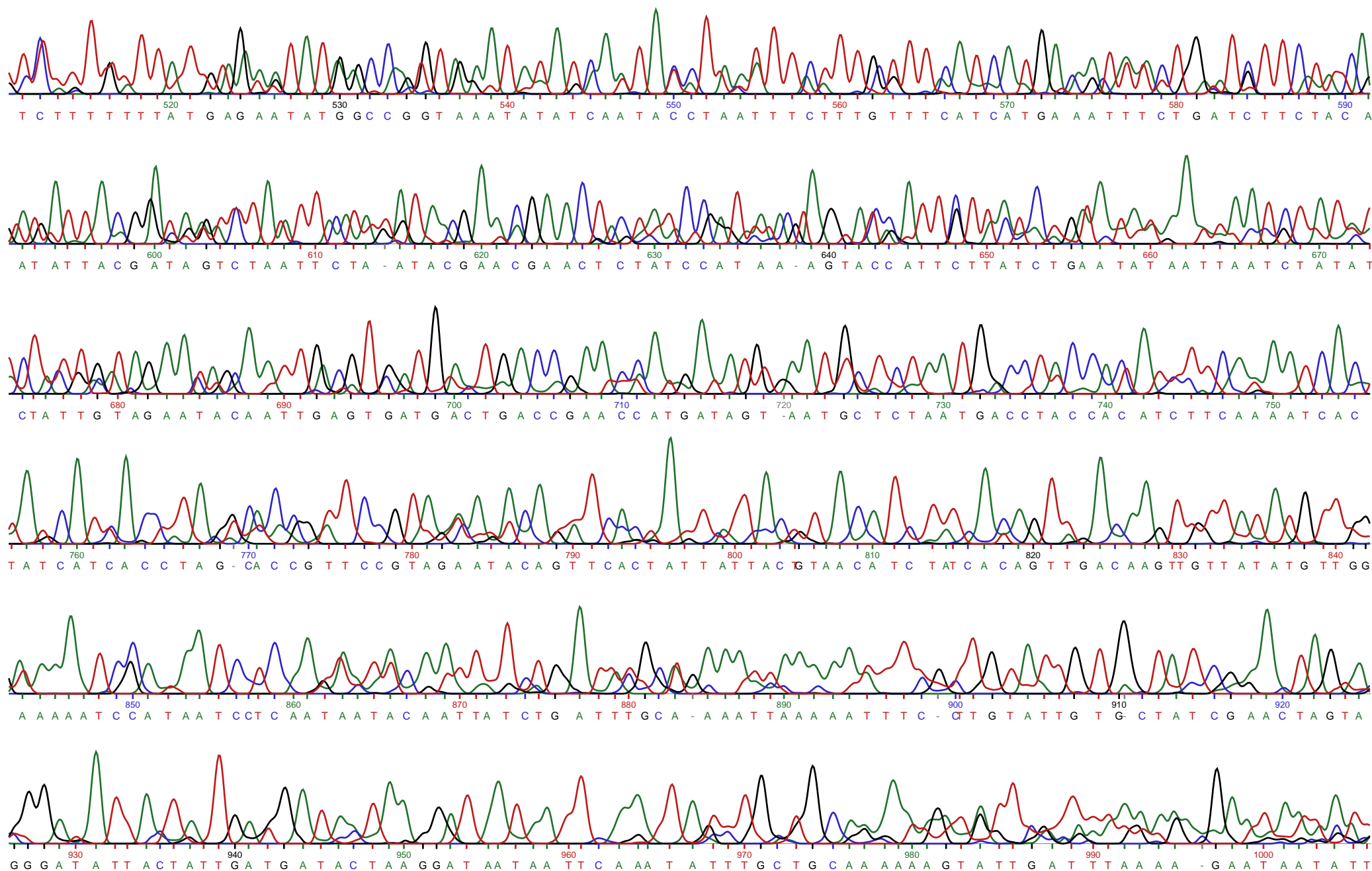

Samples: 12240  
Bases: 1025  
Average spacing: 12

Page: 3 / 3  
8/17/2022

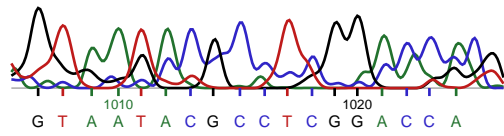

Supplement: Supporting information 2 — (ZIP) [file pone.0316479.s002.zip › 002KN2R_PREMIX_Plate_KELCH2_C11.pdf]

Samples: 13422  
Bases: 826  
Average spacing: 17

Page: 1 / 3  
8/17/2022

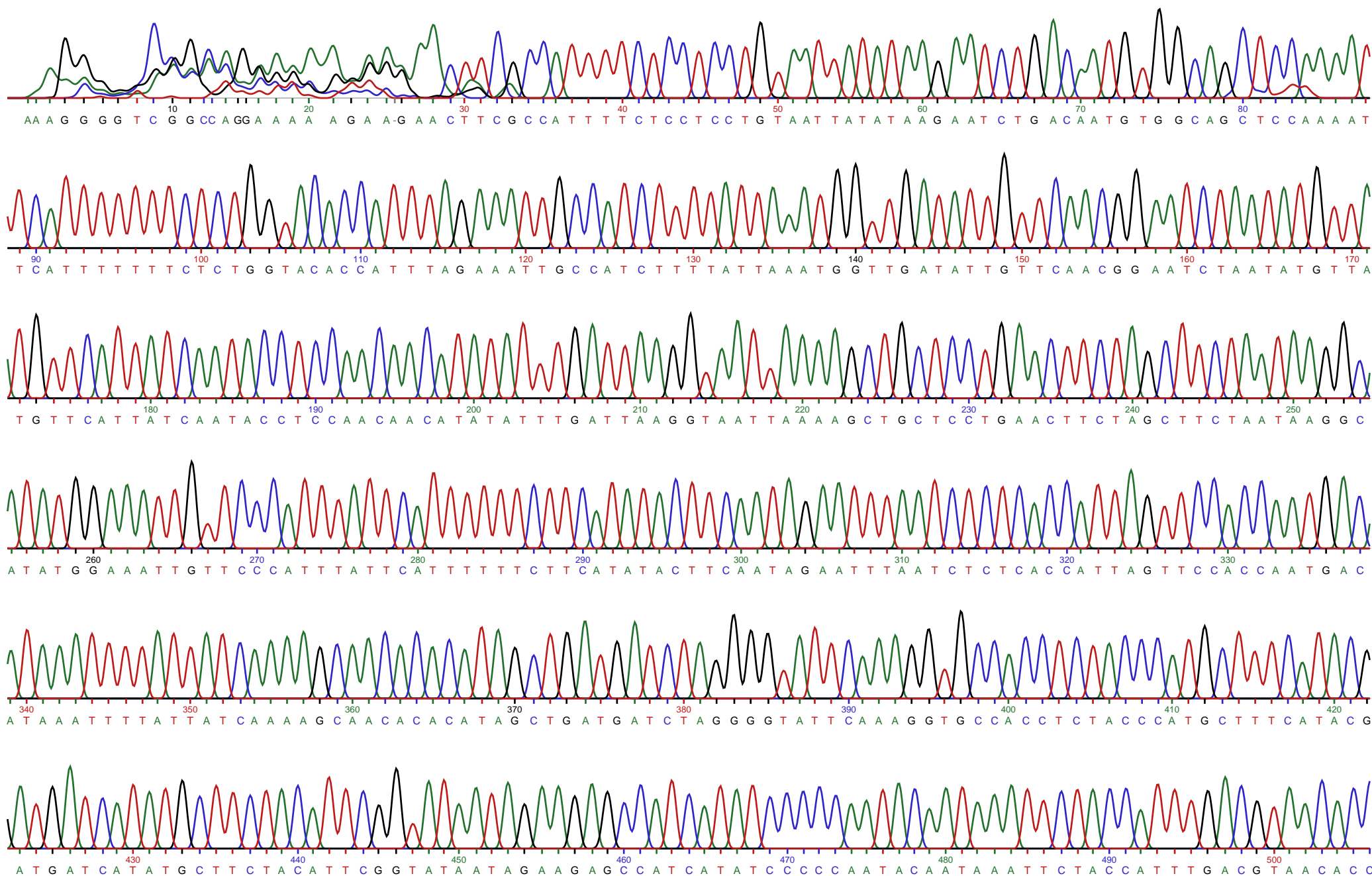

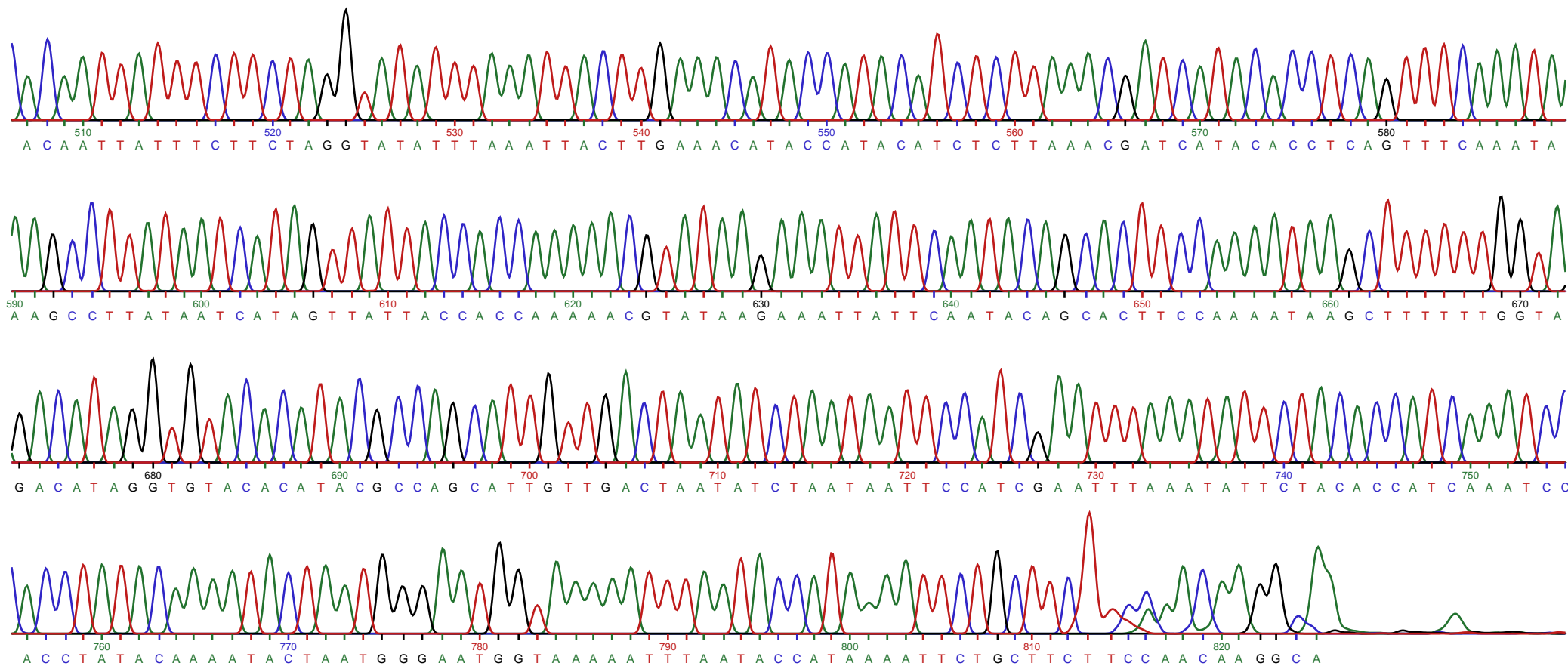

Samples: 13422  
Bases: 826  
Average spacing: 17

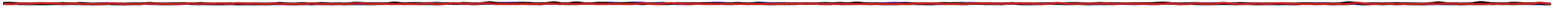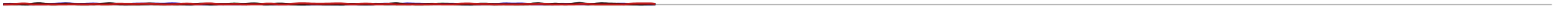

Supplement: Supporting information 2 — (ZIP) [file pone.0316479.s002.zip › 003KN1R_PREMIX_Plate_KELCH1_A10.pdf]

Page: 1 / 3  
8/17/2022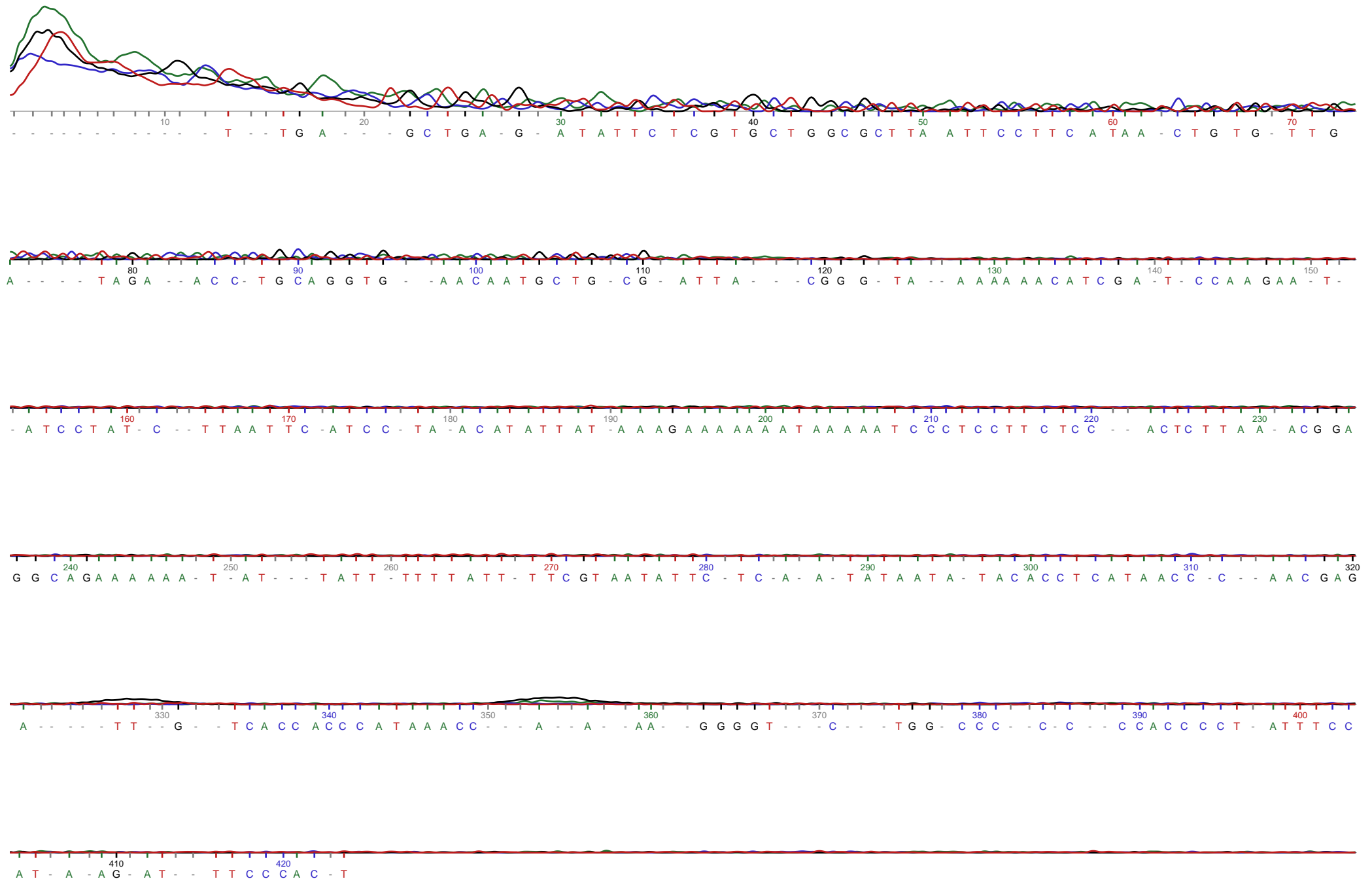

Samples: 14824  
Bases: 425  
Average spacing: 35

Page: 2 / 3  
8/17/2022

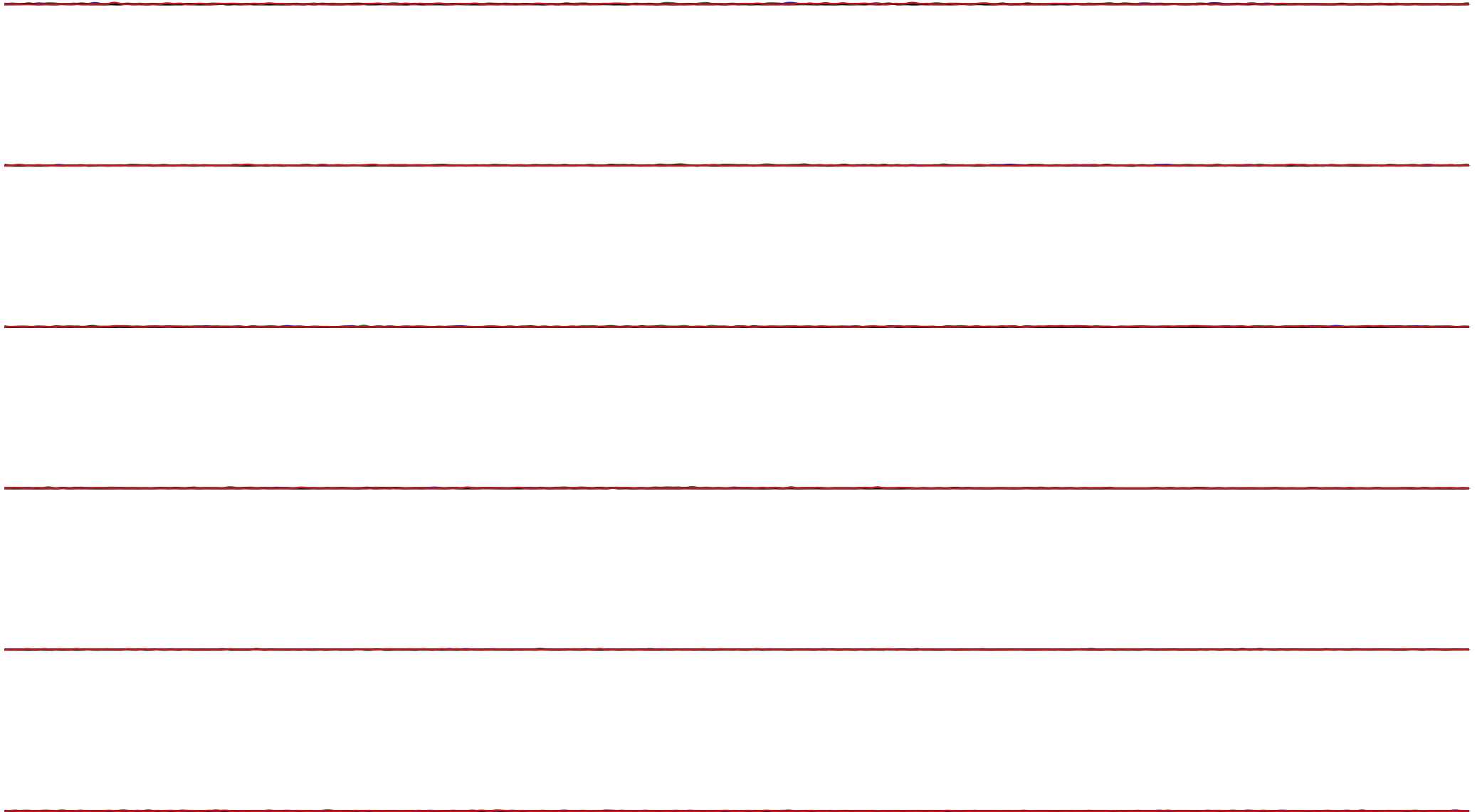

Supplement: Supporting information 2 — (ZIP) [file pone.0316479.s002.zip › 003KN2F_PREMIX_Plate_KELCH1_F10.pdf]

Samples: 11847  
Bases: 985  
Average spacing: 13

Page: 1 / 2  
8/17/2022

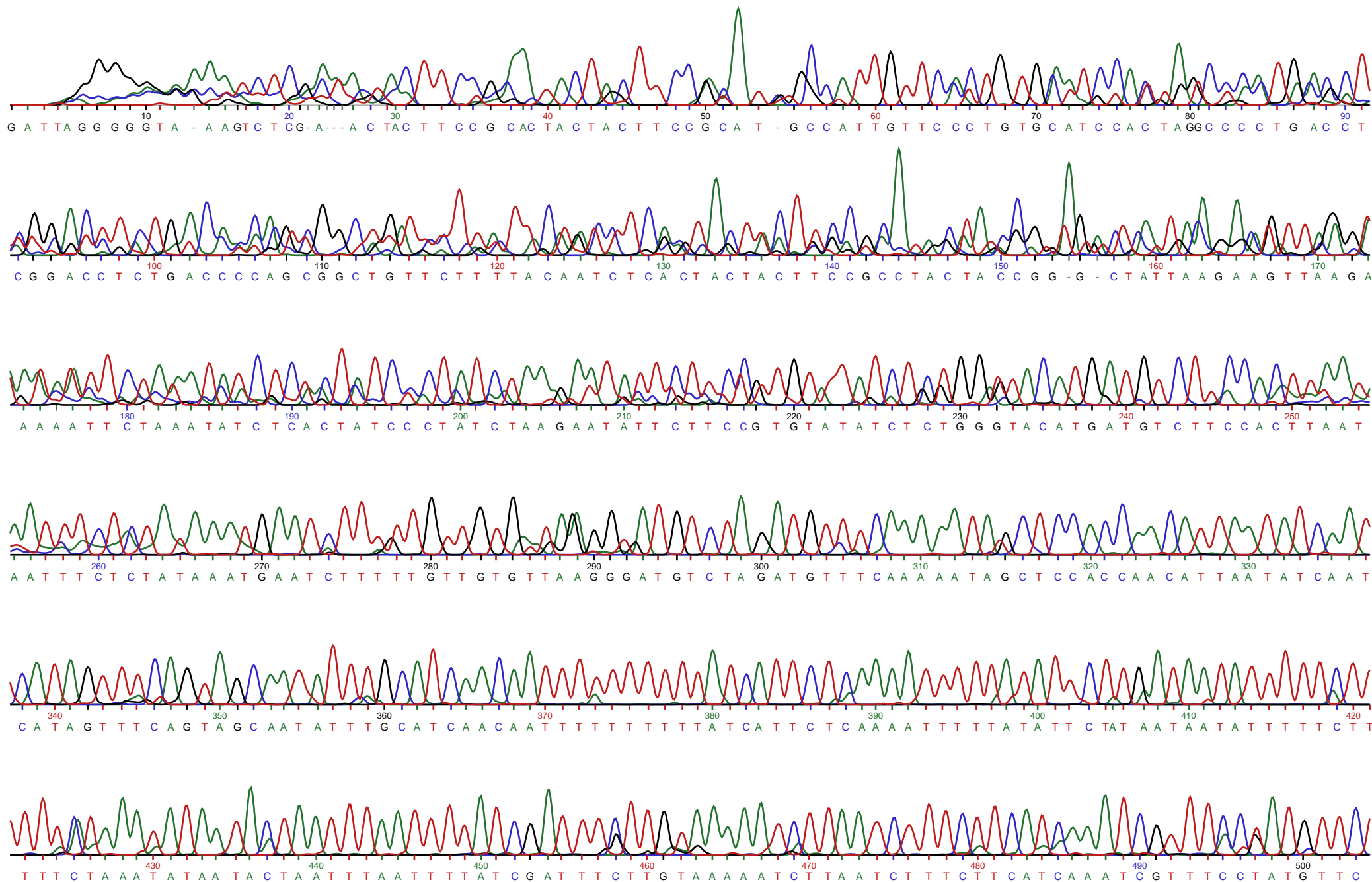

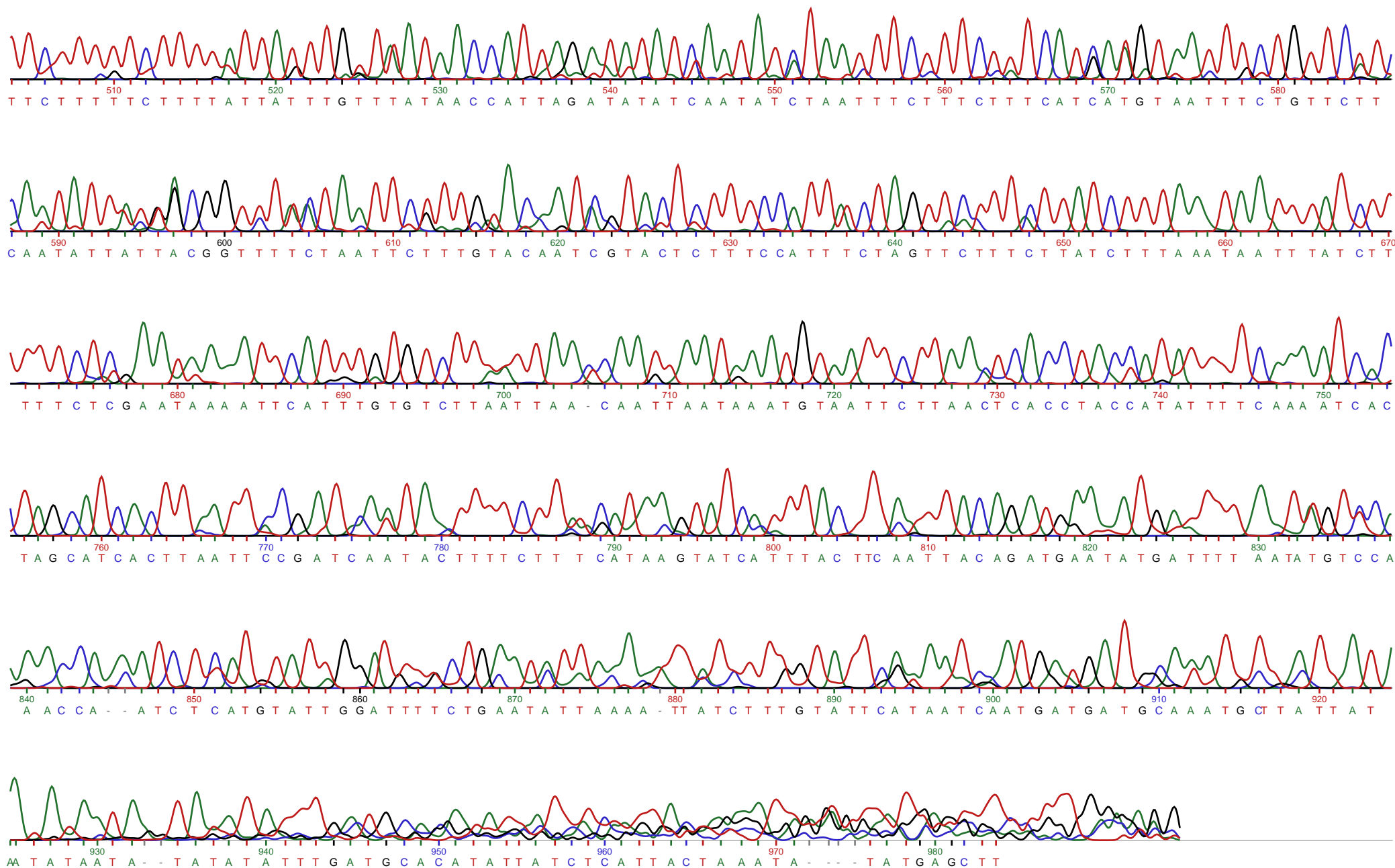

Supplement: Supporting information 2 — (ZIP) [file pone.0316479.s002.zip › 003KN2R_PREMIX_Plate_KELCH2_C12.pdf]

Samples: 13260  
Bases: 831  
Average spacing: 16

Page: 1 / 3  
8/17/2022

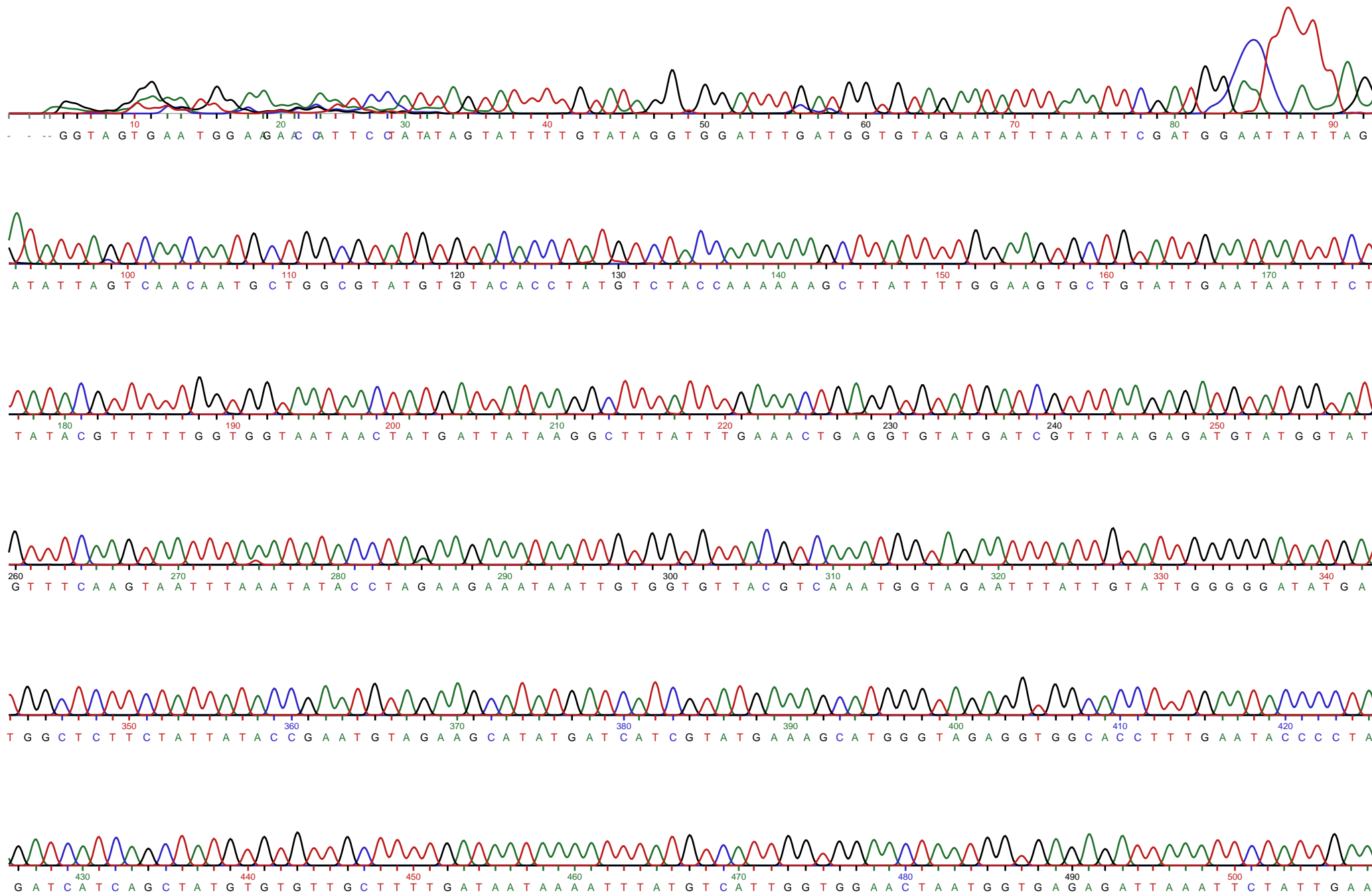

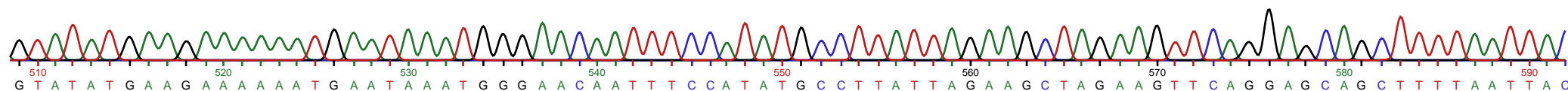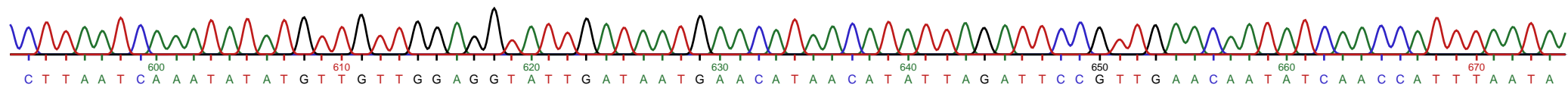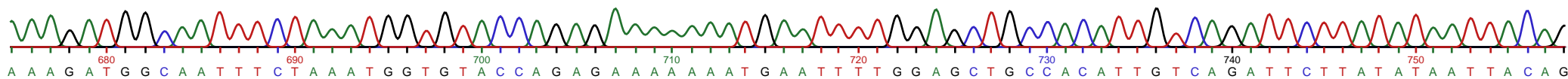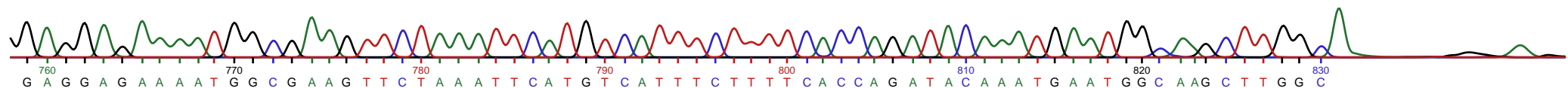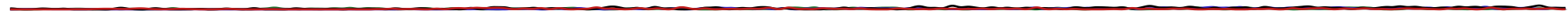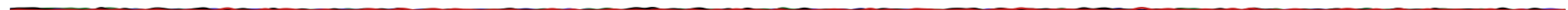

Samples: 13260  
Bases: 831  
Average spacing: 16

Page: 3 / 3  
8/17/2022

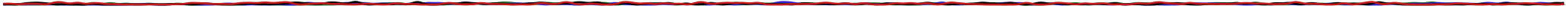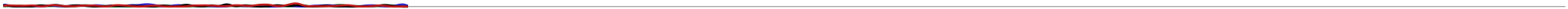

Supplement: Supporting information 2 — (ZIP) [file pone.0316479.s002.zip › 003KNIFW_PREMIX_Plate_CORKELCH_D08.pdf]

Page: 1 / 3  
8/17/2022

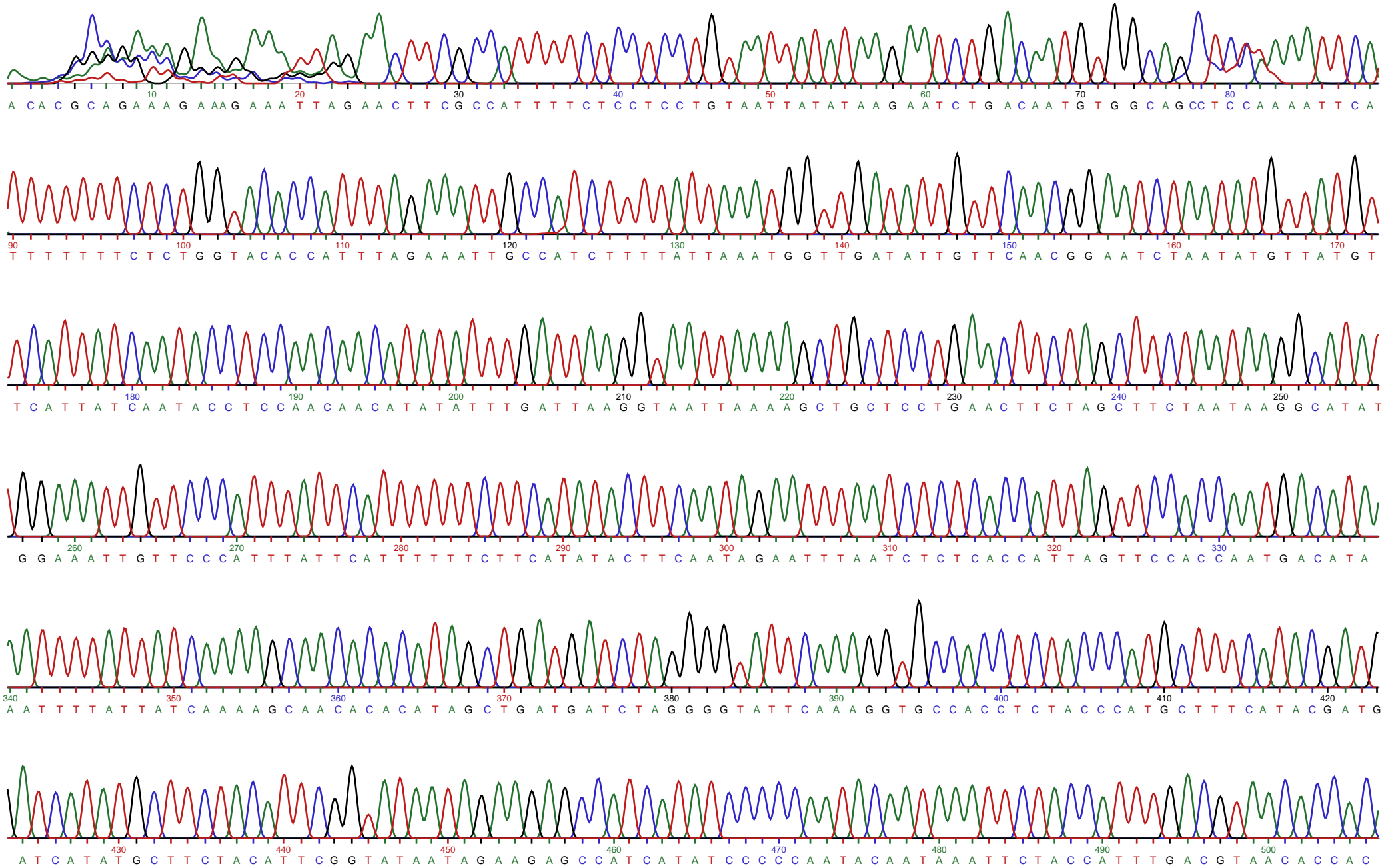

Samples: 13114  
Bases: 824  
Average spacing: 16

Page: 2 / 3  
8/17/2022

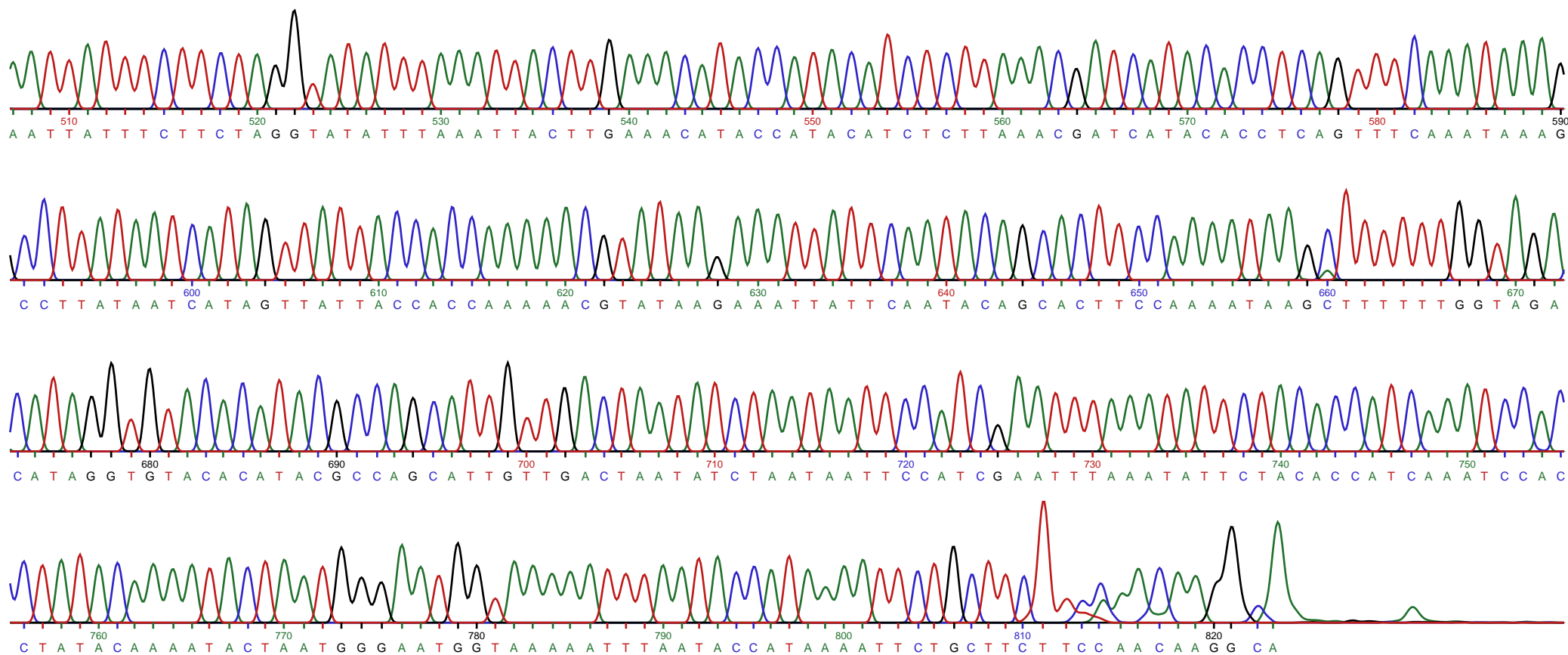

Samples: 13114  
Bases: 824  
Average spacing: 16

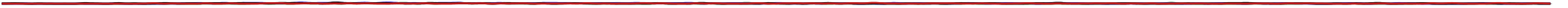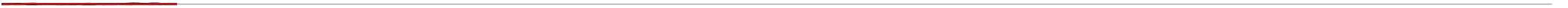

Supplement: Supporting information 2 — (ZIP) [file pone.0316479.s002.zip › 004KN1R_PREMIX_Plate_KELCH1_A11.pdf]

Page: 1 / 3  
8/17/2022

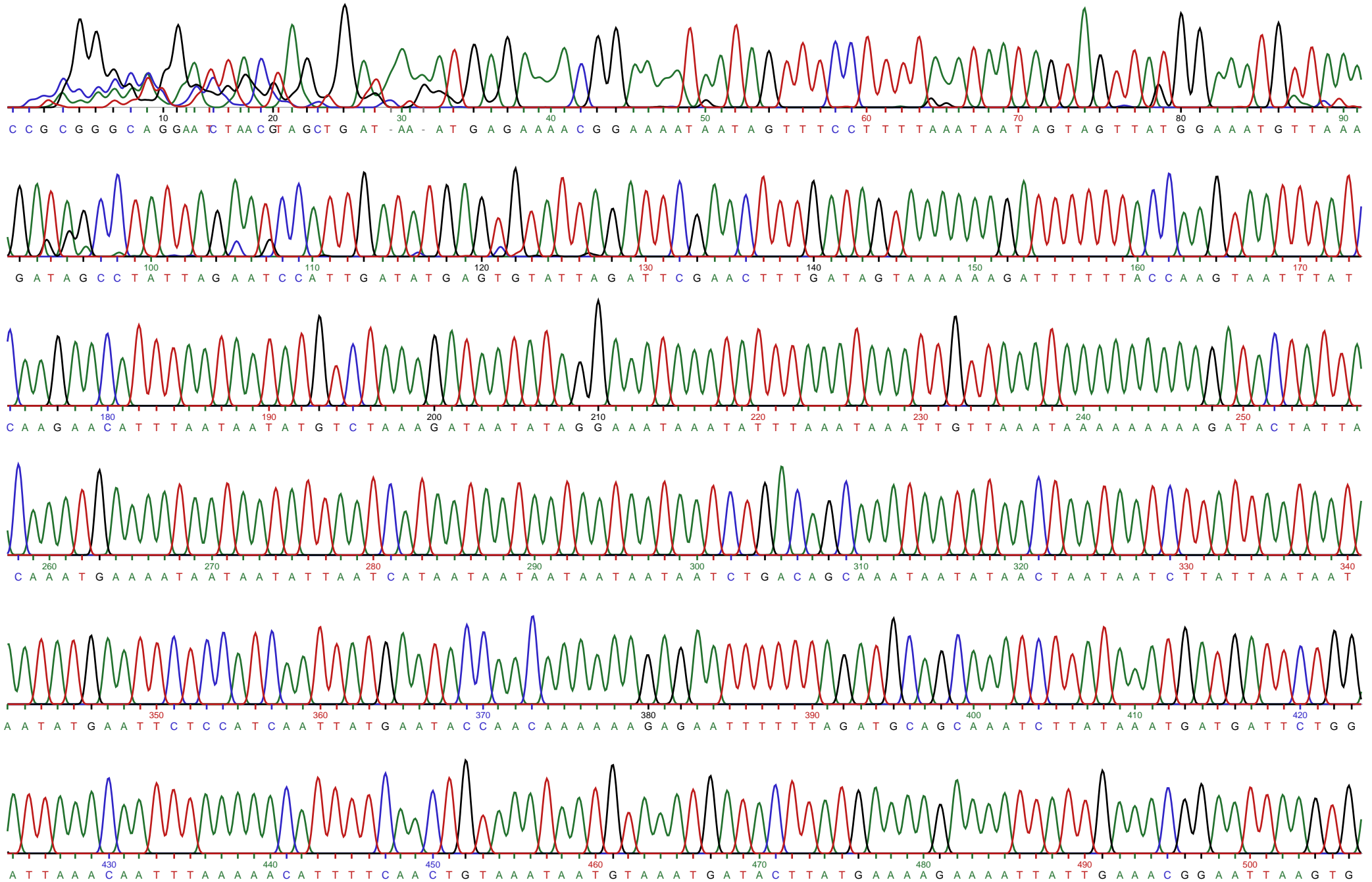

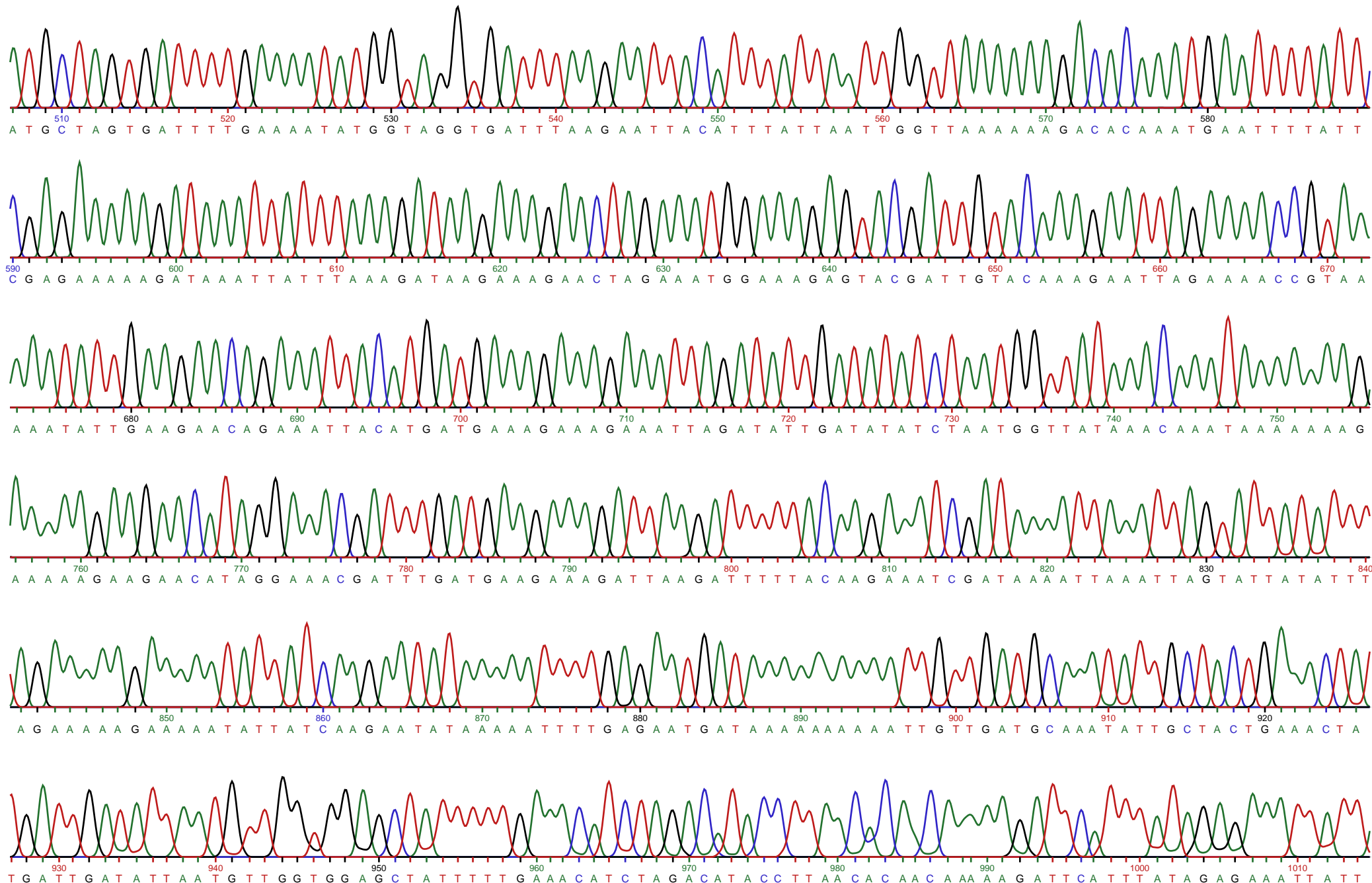

Samples: 13161  
Bases: 1116  
Average spacing: 12

Page: 3 / 3  
8/17/2022

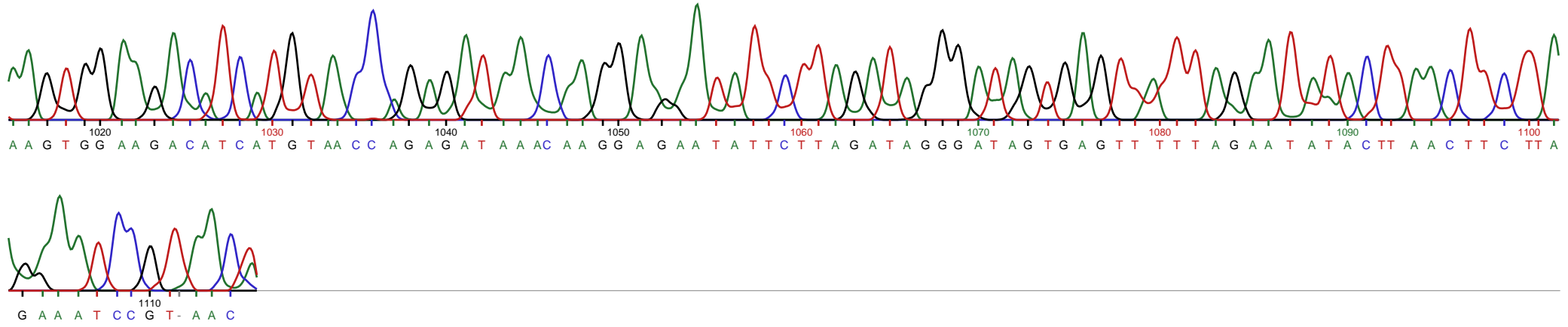

Supplement: Supporting information 2 — (ZIP) [file pone.0316479.s002.zip › 004KN2F_PREMIX_Plate_KELCH1_F11.pdf]

Page: 1 / 3  
8/17/2022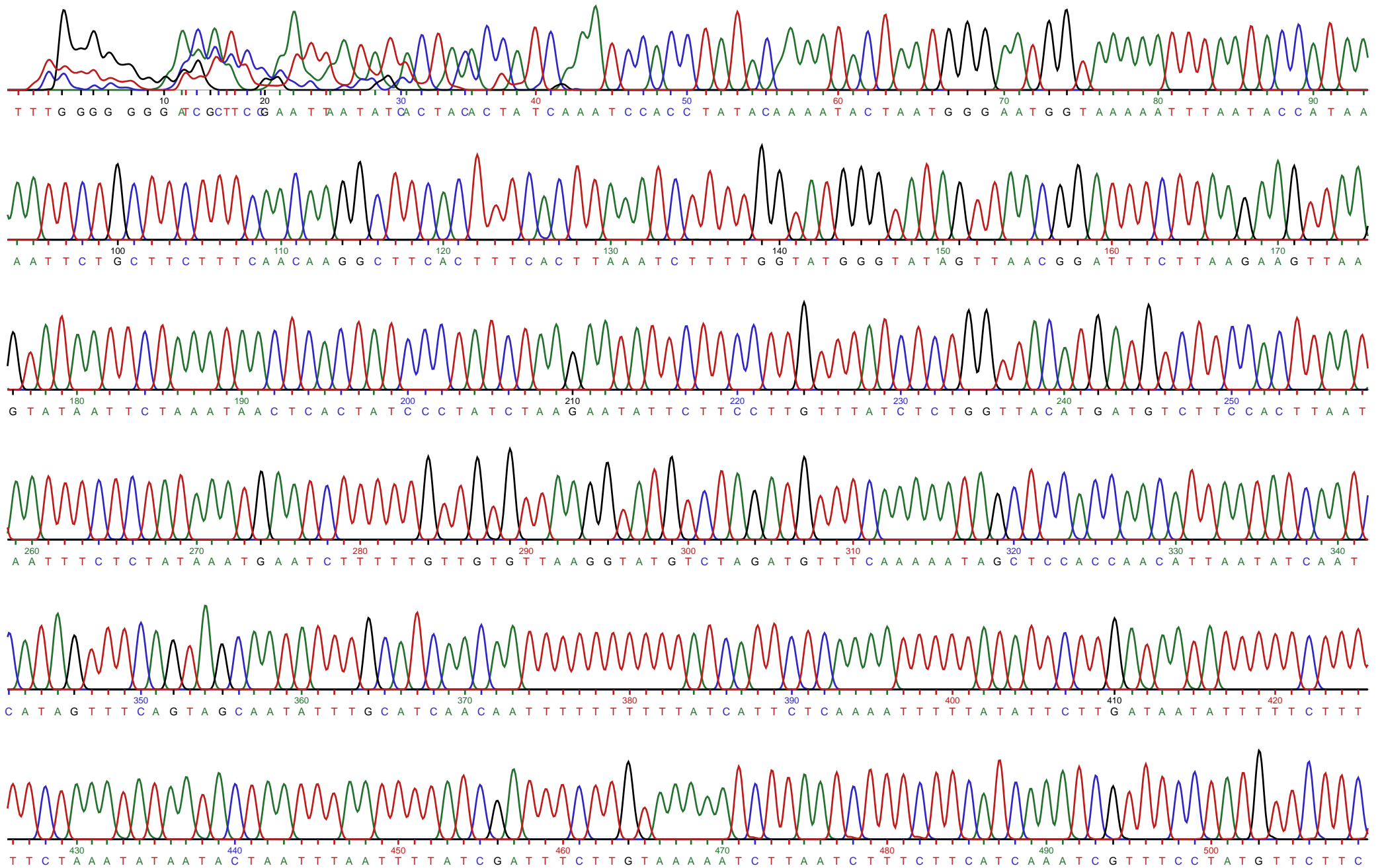

Page: 2 / 3  
8/17/2022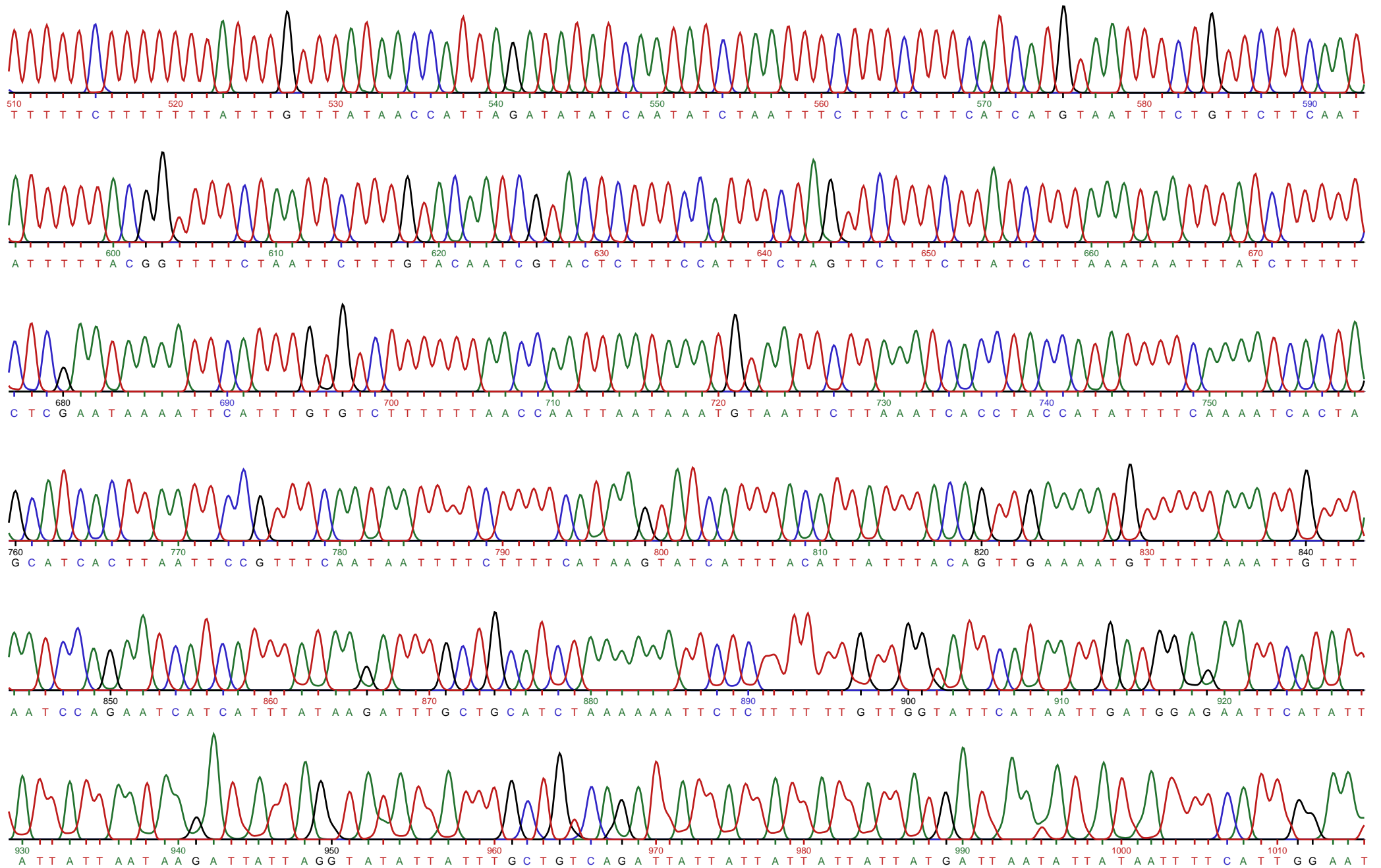

Samples: 12594  
Bases: 1065  
Average spacing: 12

Page: 3 / 3  
8/17/2022

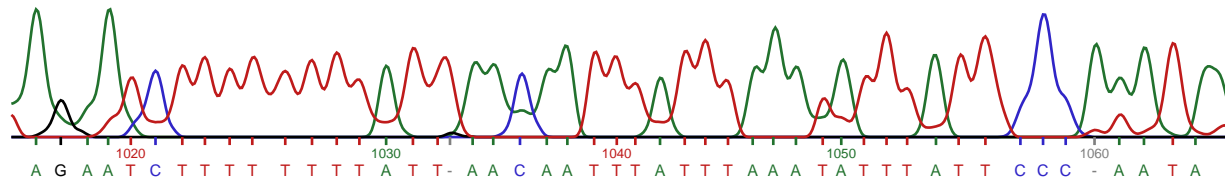

Supplement: Supporting information 2 — (ZIP) [file pone.0316479.s002.zip › 004KN2R_PREMIX_Plate_KELCH2_D01.pdf]

Samples: 13099  
Bases: 843  
Average spacing: 16

Page: 1 / 3  
8/17/2022

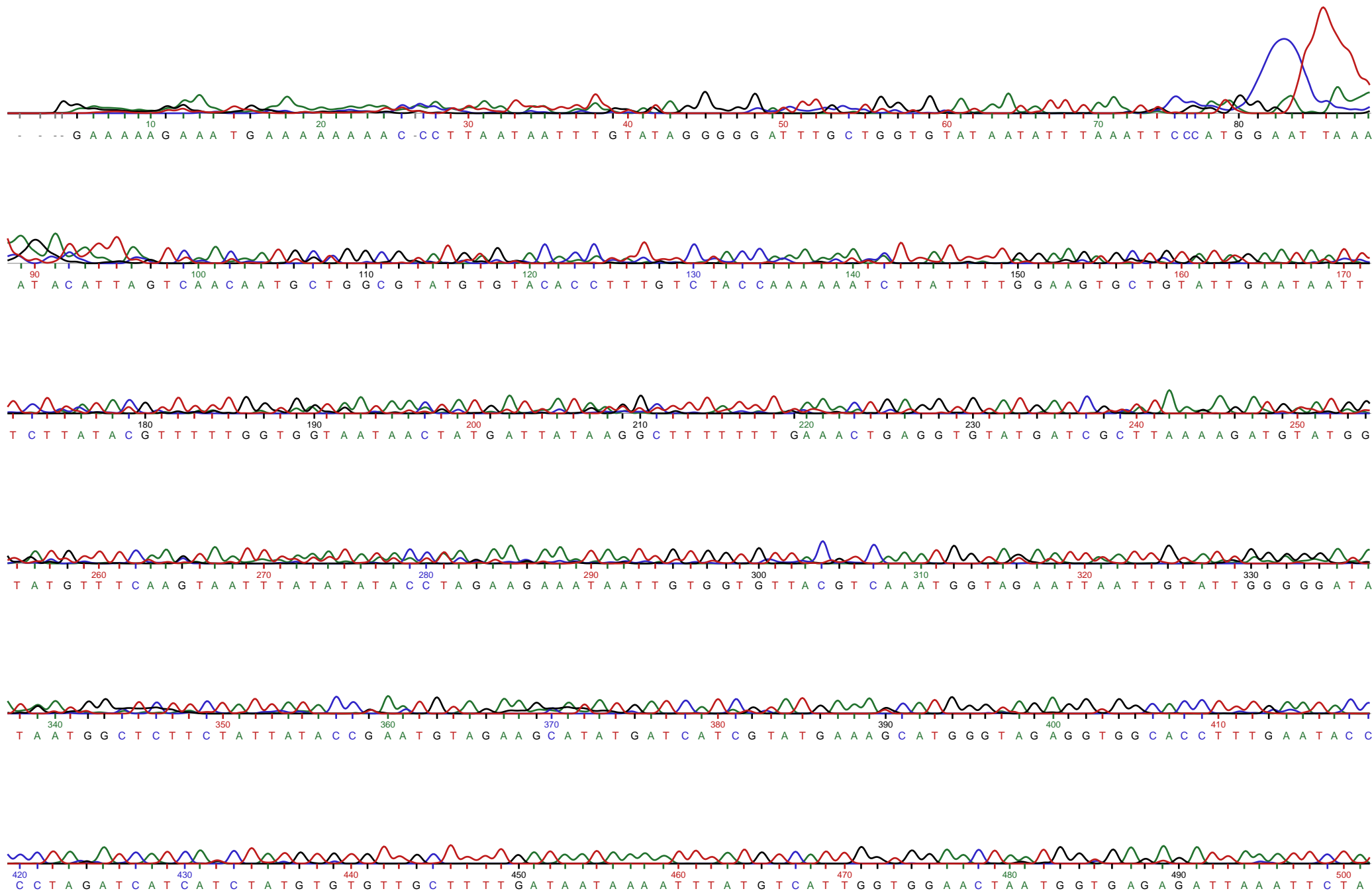

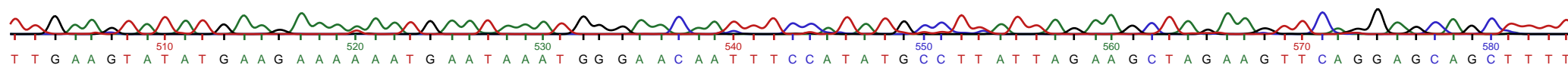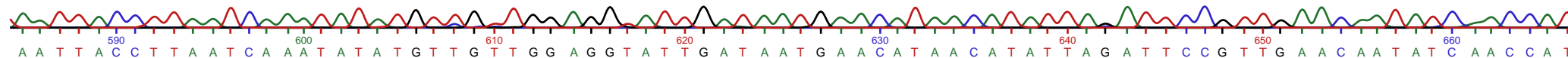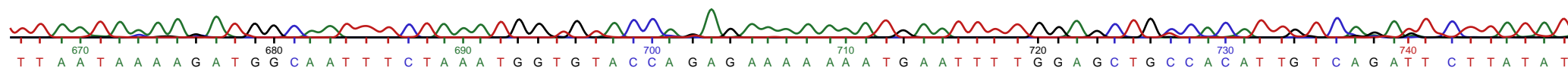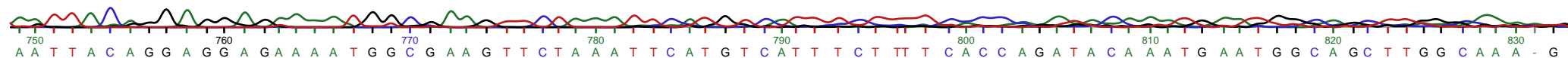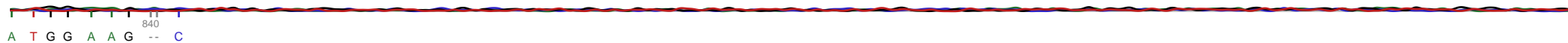

Samples: 13099  
Bases: 843  
Average spacing: 16

Page: 3 / 3  
8/17/2022

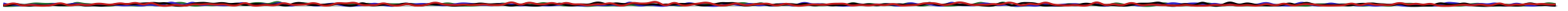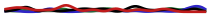

Supplement: Supporting information 2 — (ZIP) [file pone.0316479.s002.zip › 004KNIFW_PREMIX_Plate_CORKELCH_D09.pdf]

Samples: 12569  
Bases: 529  
Average spacing: 24

Page: 1 / 3  
8/17/2022

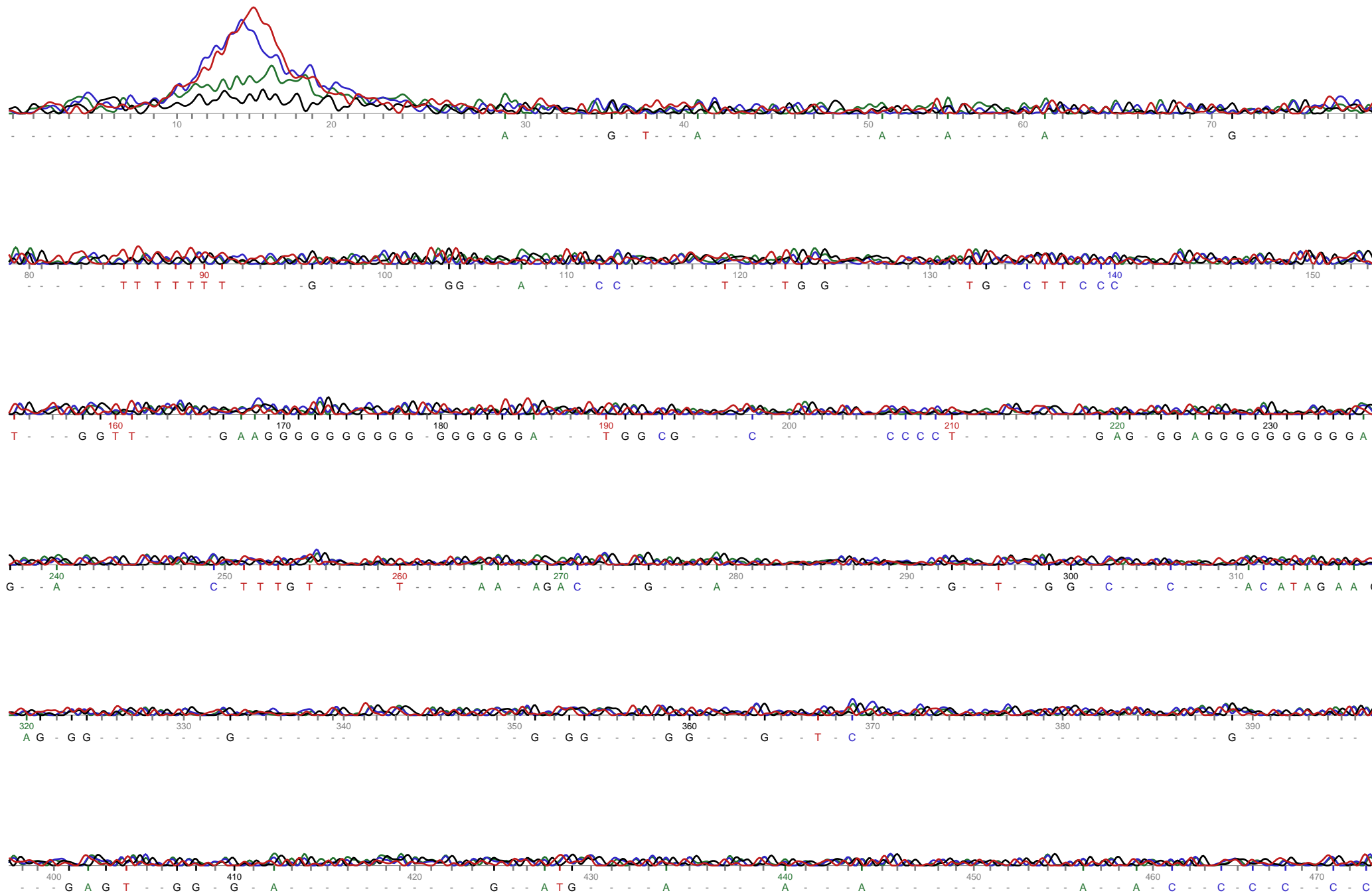

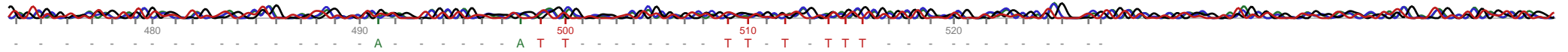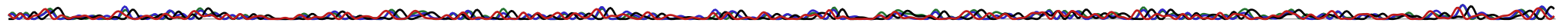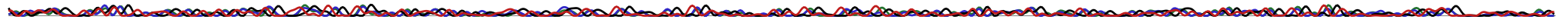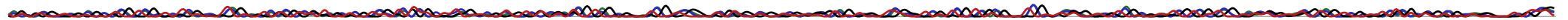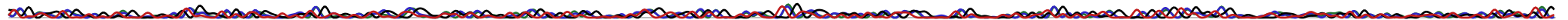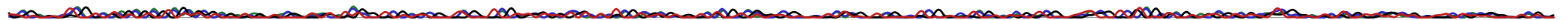

Samples: 12569  
Bases: 529  
Average spacing: 24

Page: 3 / 3  
8/17/2022

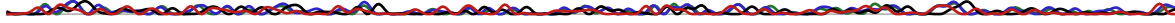

Supplement: Supporting information 2 — (ZIP) [file pone.0316479.s002.zip › 005KN1R_PREMIX_Plate_KELCH1_A12.pdf]

Page: 1 / 3  
8/17/2022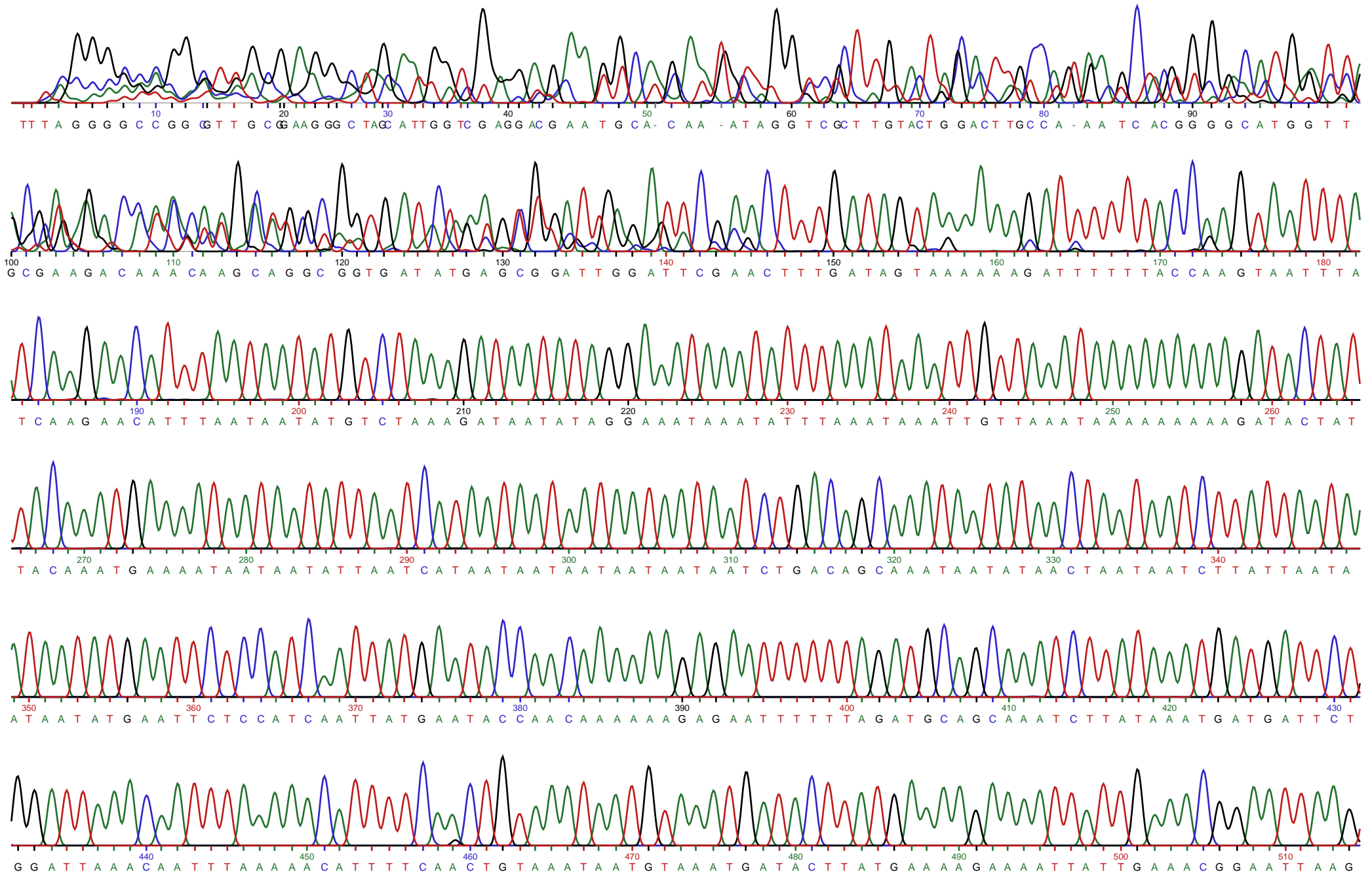

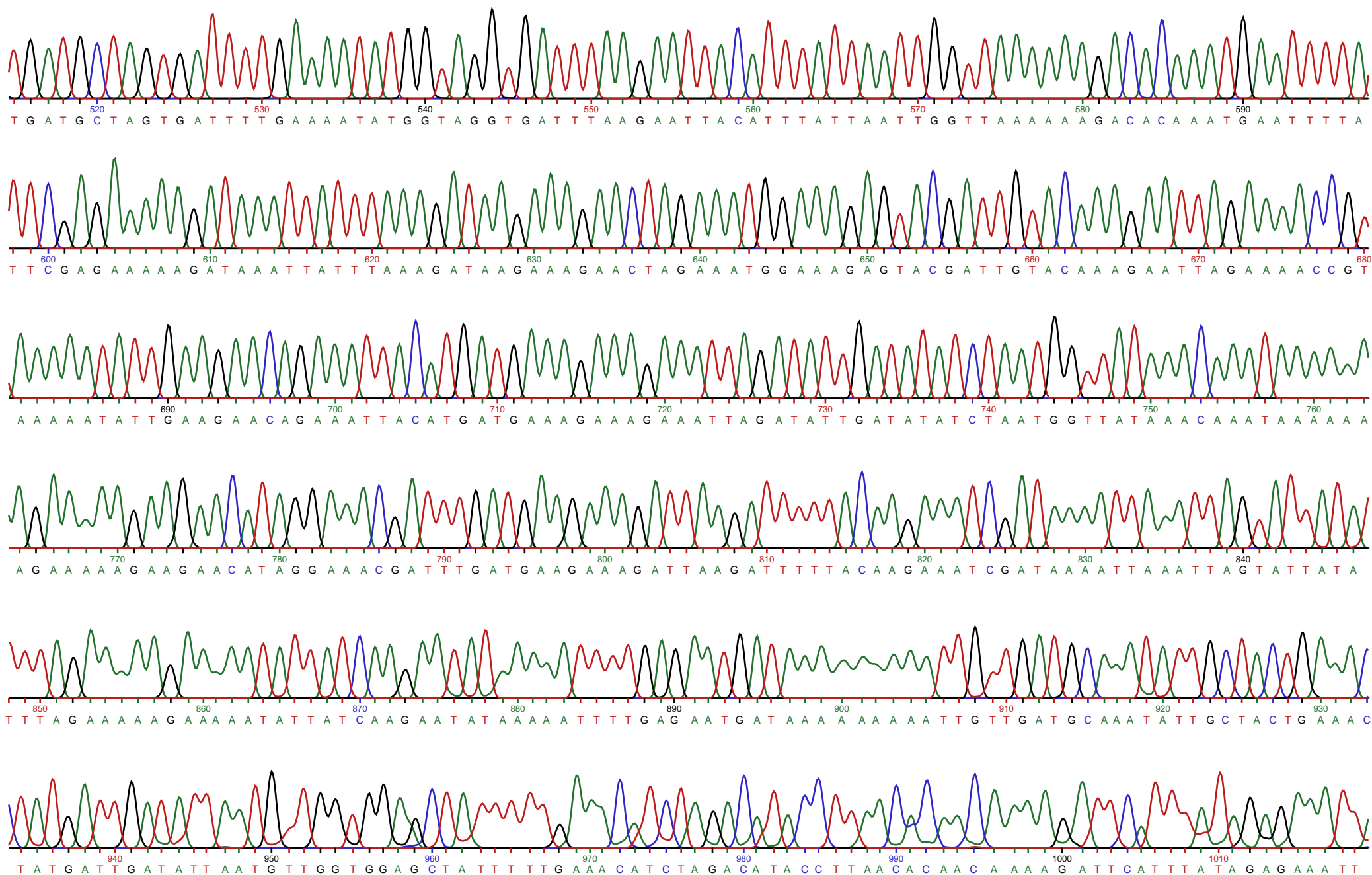

Samples: 12989  
Bases: 1104  
Average spacing: 12

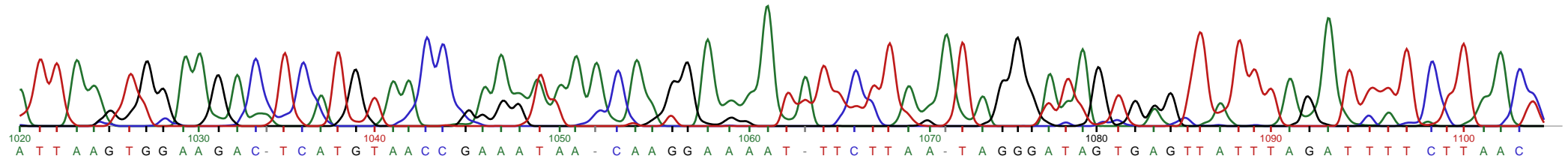

Supplement: Supporting information 2 — (ZIP) [file pone.0316479.s002.zip › 005KN2F_PREMIX_Plate_KELCH1_F12.pdf]

Samples: 13278  
Bases: 1122  
Average spacing: 12

Page: 1 / 3  
8/17/2022

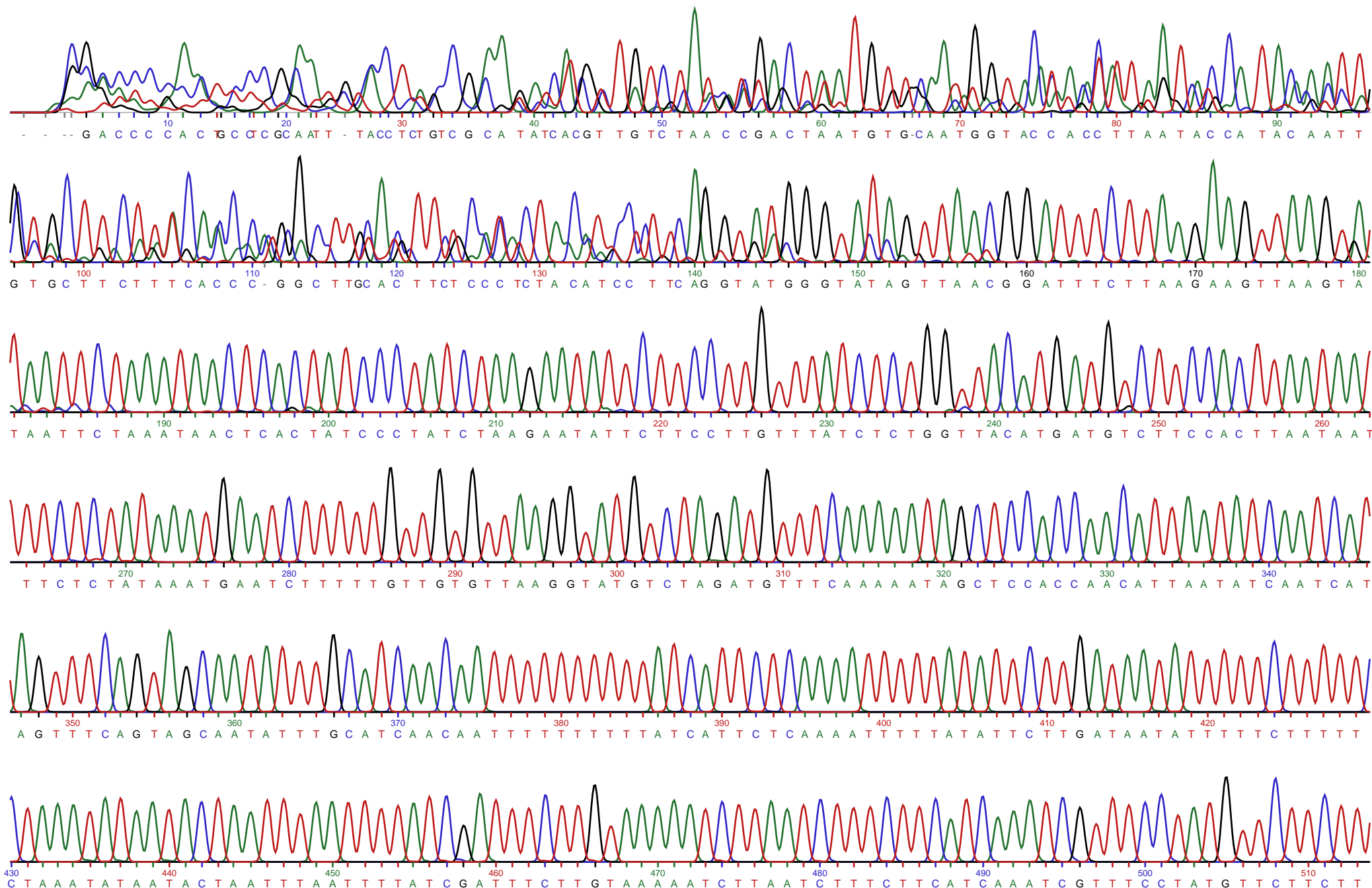

Page: 2 / 3  
8/17/2022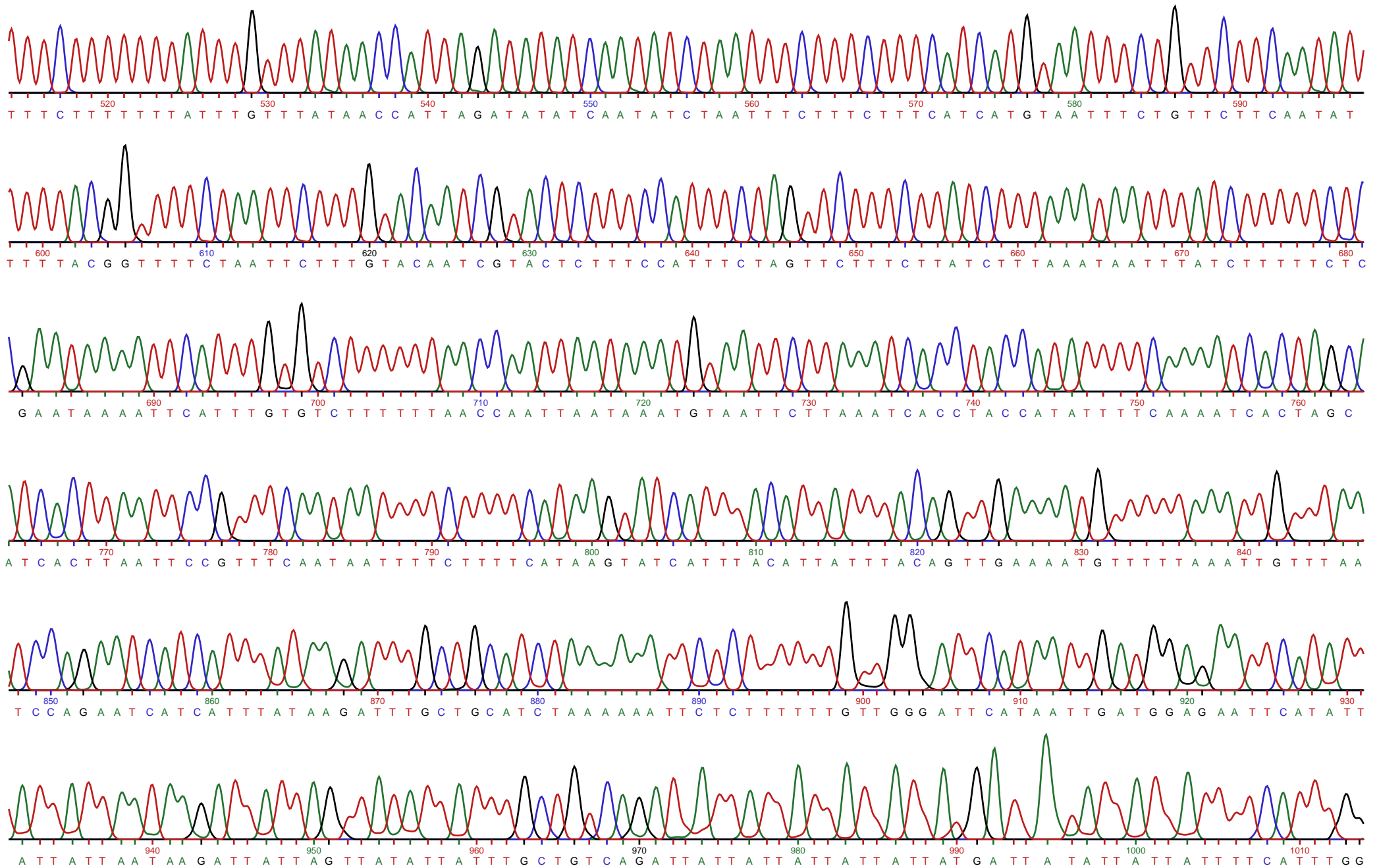

Samples: 13278  
Bases: 1122  
Average spacing: 12

Page: 3 / 3  
8/17/2022

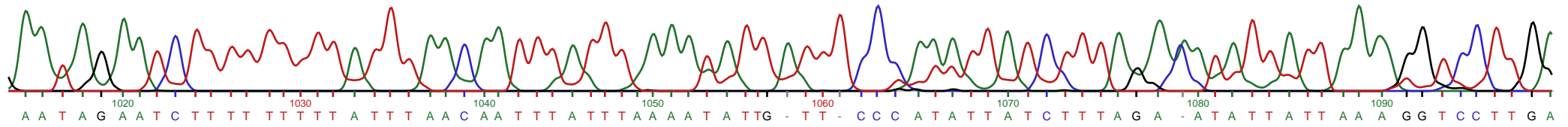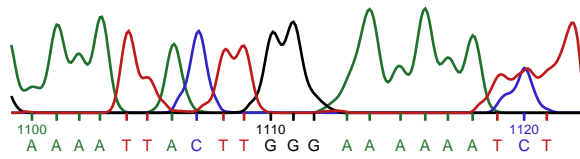

Supplement: Supporting information 2 — (ZIP) [file pone.0316479.s002.zip › 005KN2R_PREMIX_Plate_KELCH2_D02.pdf]

Samples: 12933  
Bases: 829  
Average spacing: 16

Page: 1 / 3  
8/17/2022

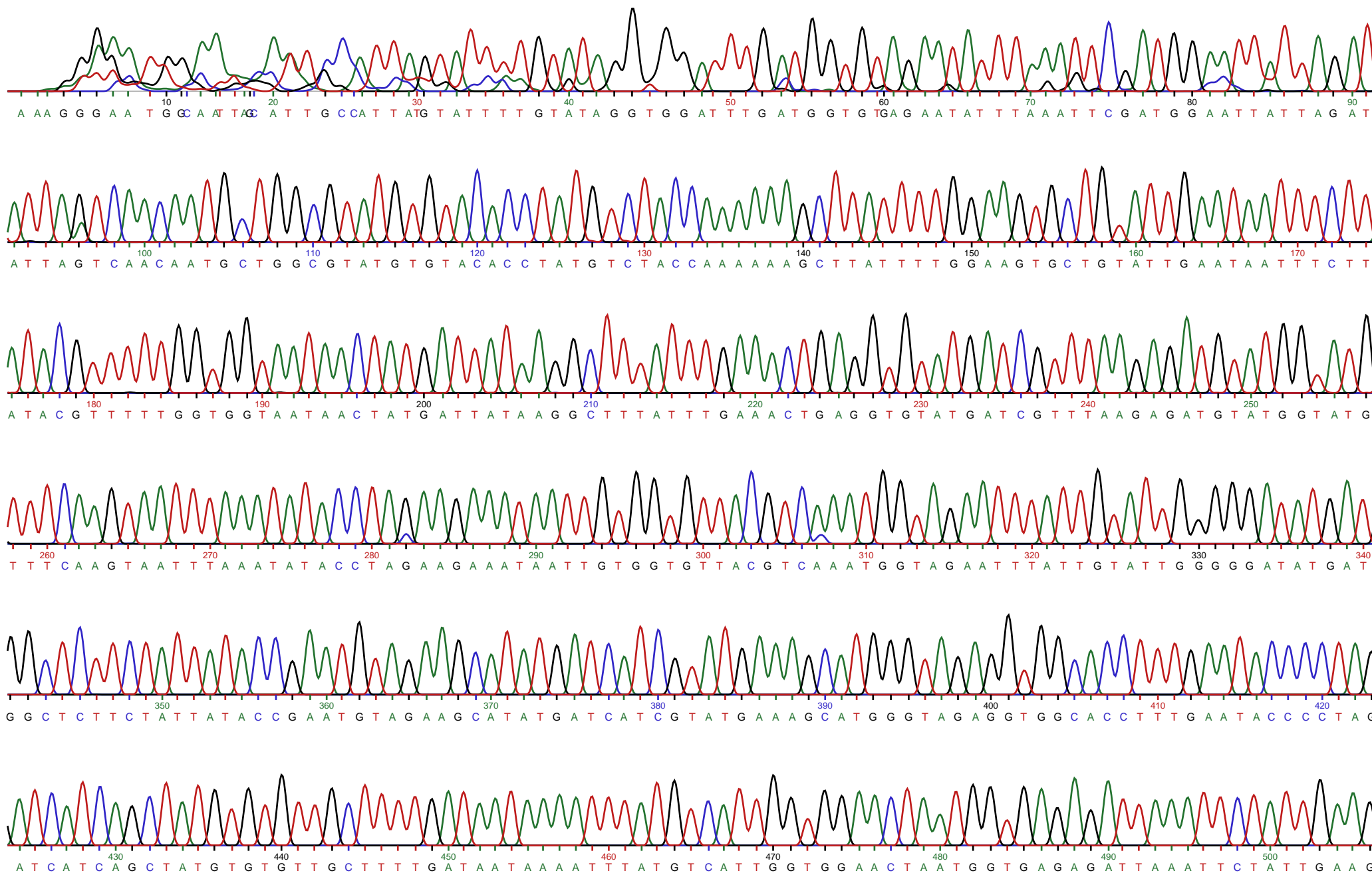

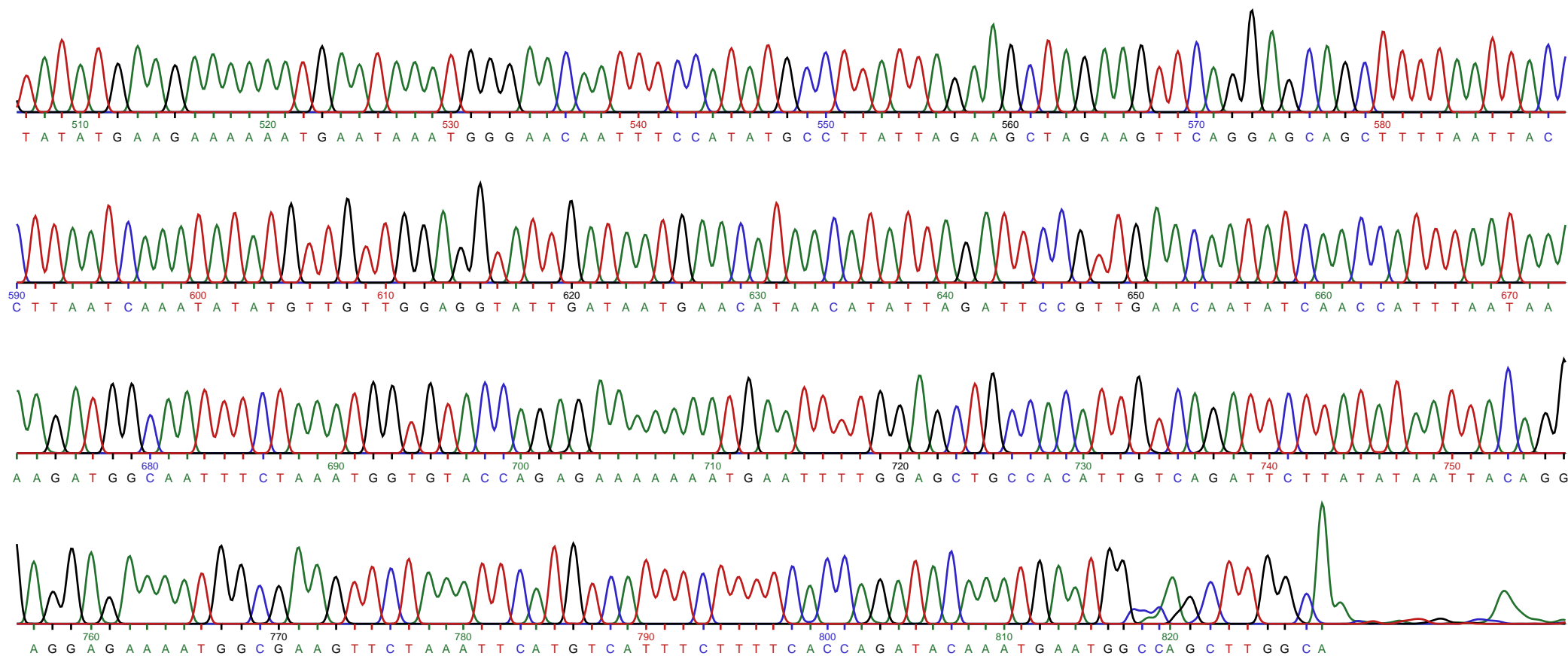

Samples: 12933  
Bases: 829  
Average spacing: 16

Page: 3 / 3  
8/17/2022

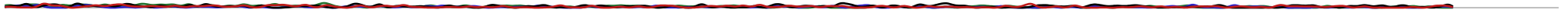

Supplement: Supporting information 2 — (ZIP) [file pone.0316479.s002.zip › 005KNIFW_PREMIX_Plate_CORKELCH_D10.pdf]

Samples: 13795  
Bases: 827  
Average spacing: 17

Page: 1 / 3  
8/17/2022

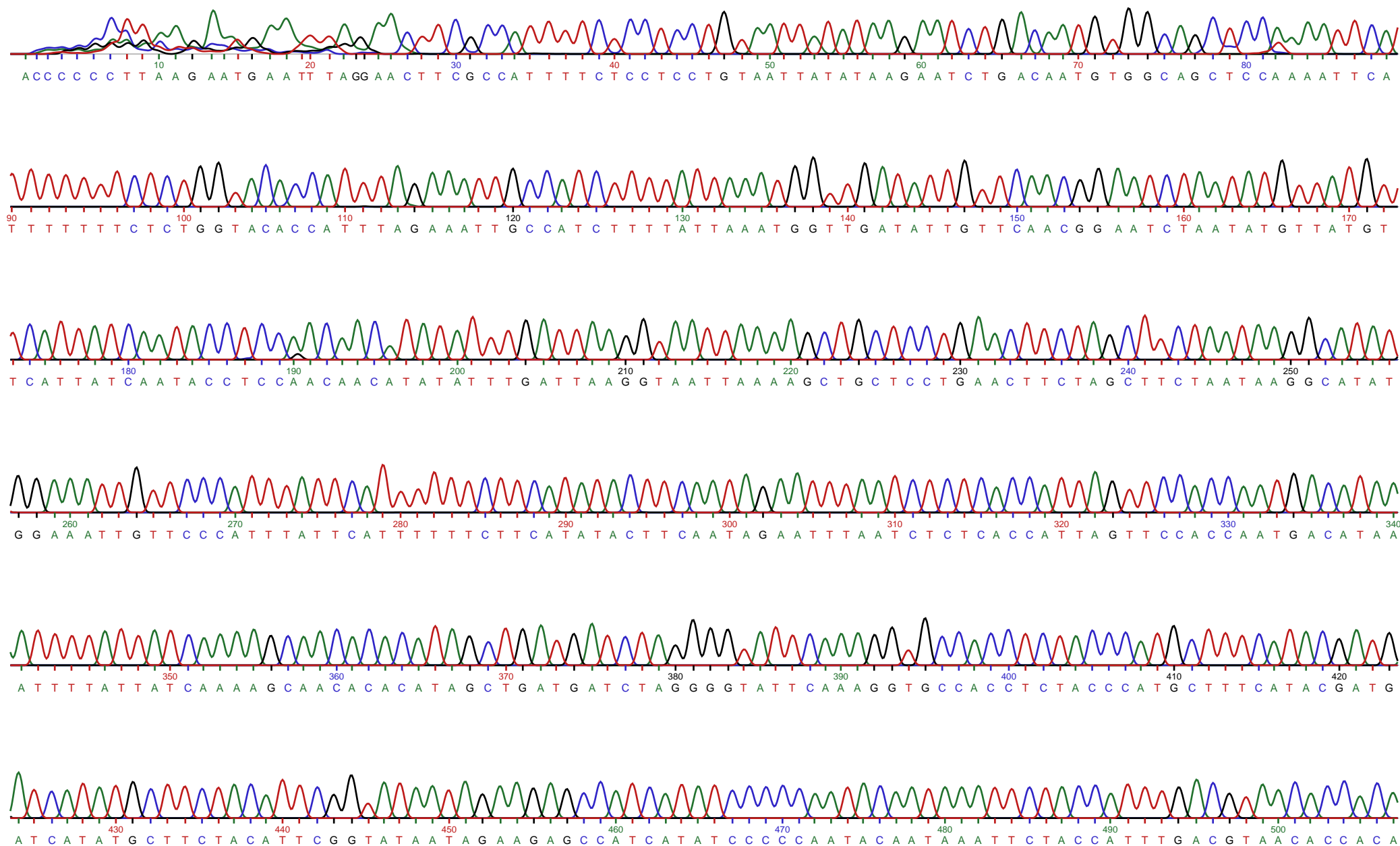

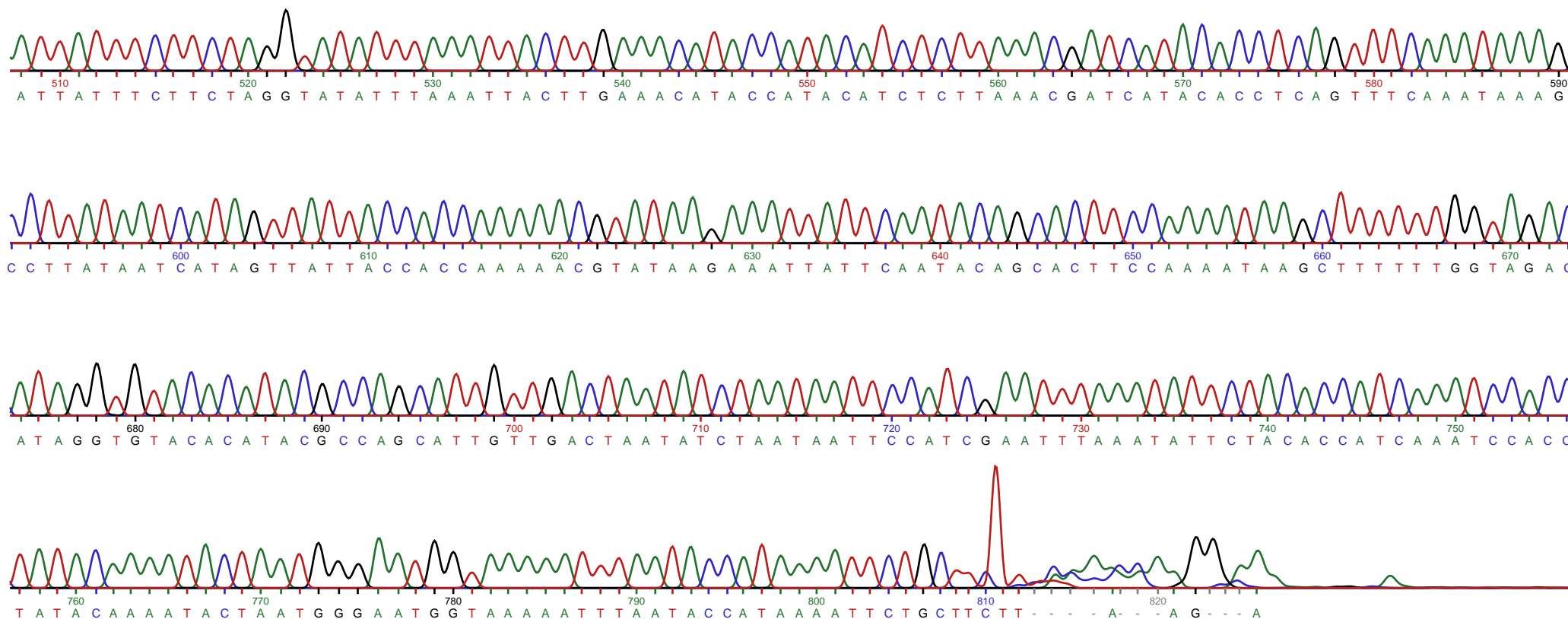

---

---

Supplement: Supporting information 2 — (ZIP) [file pone.0316479.s002.zip › 006KN1R_PREMIX_Plate_KELCH1_B01.pdf]

Page: 1 / 3  
8/17/2022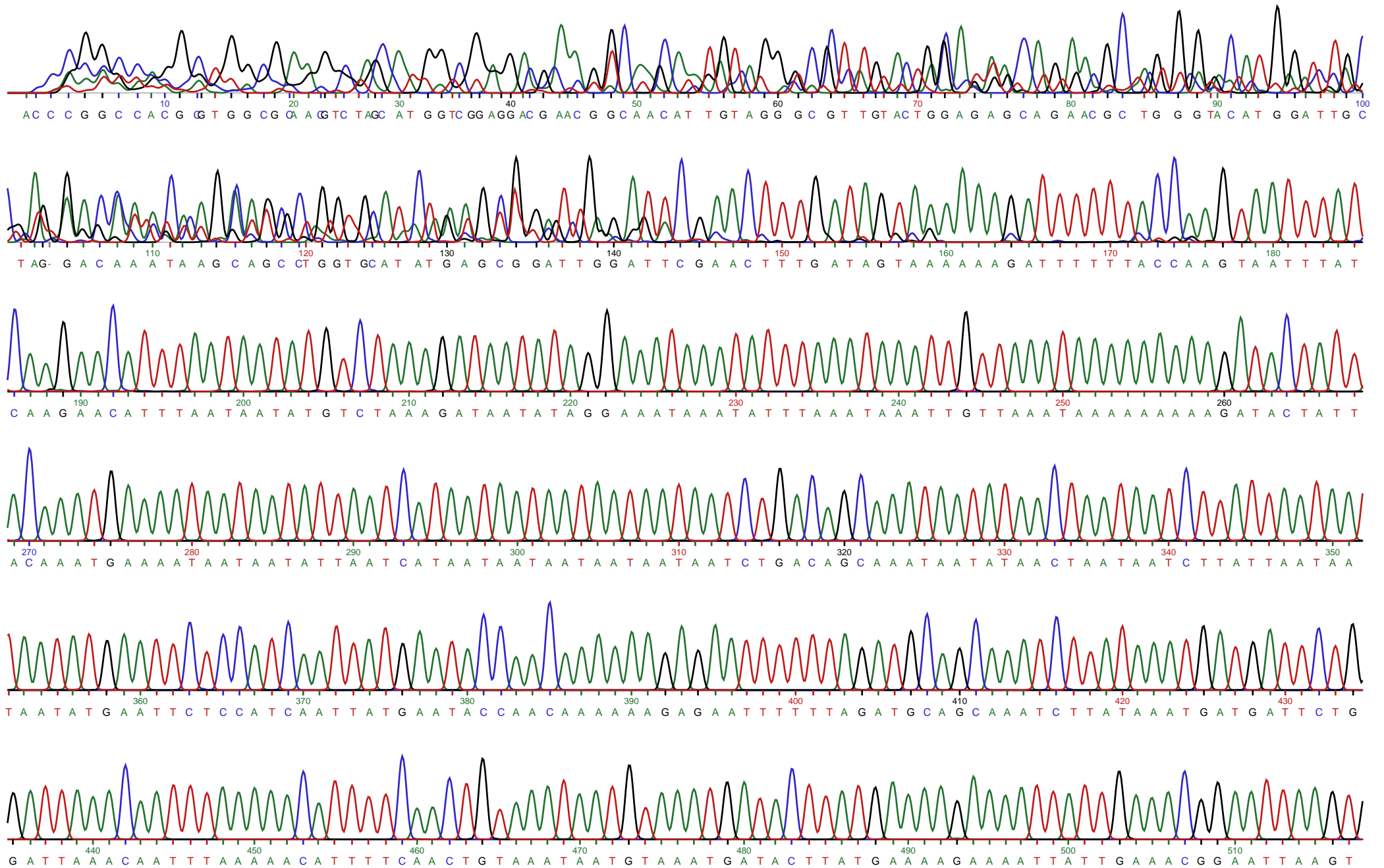

Samples: 13605  
Bases: 1161  
Average spacing: 12

Page: 2 / 3  
8/17/2022

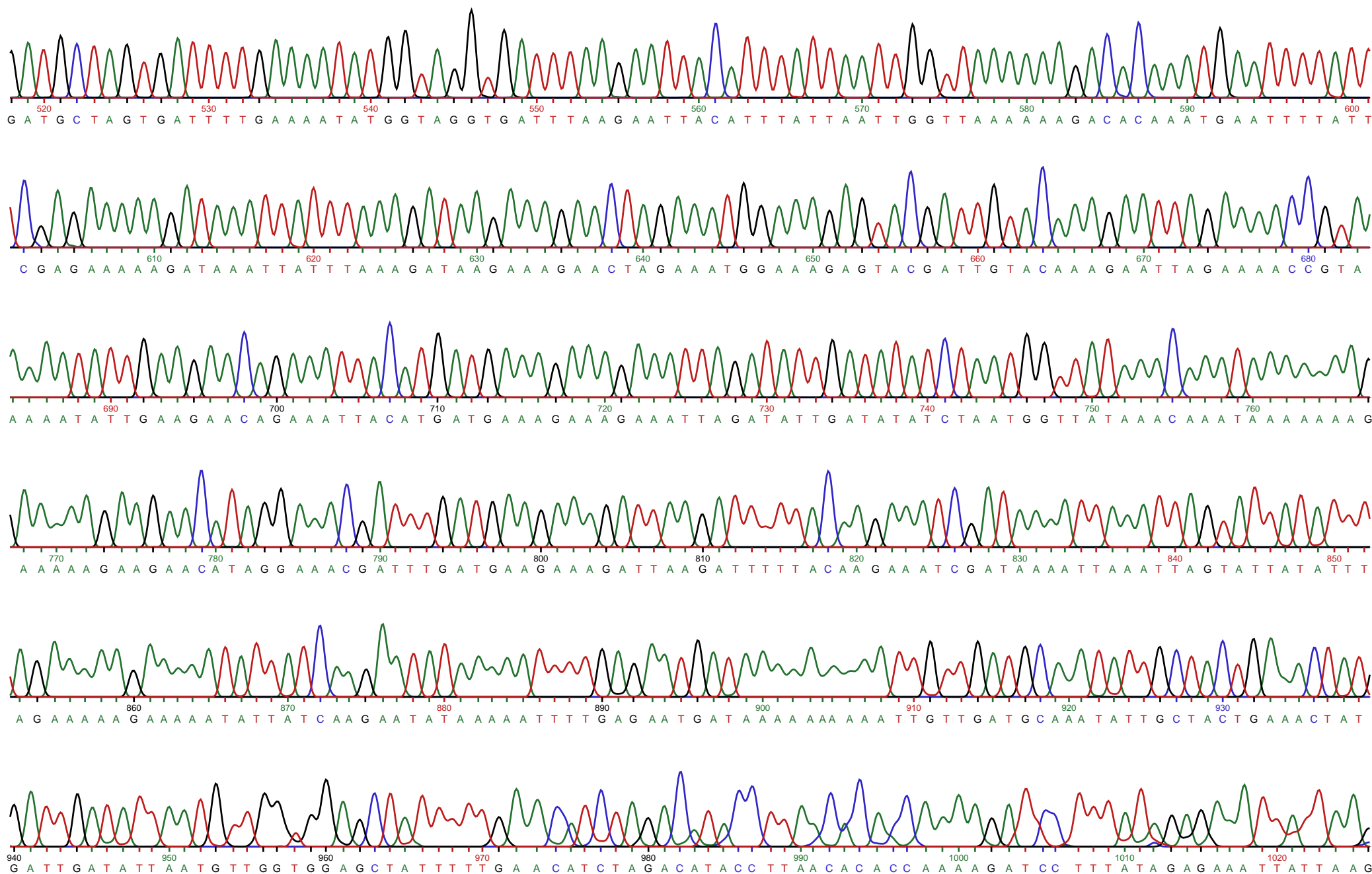

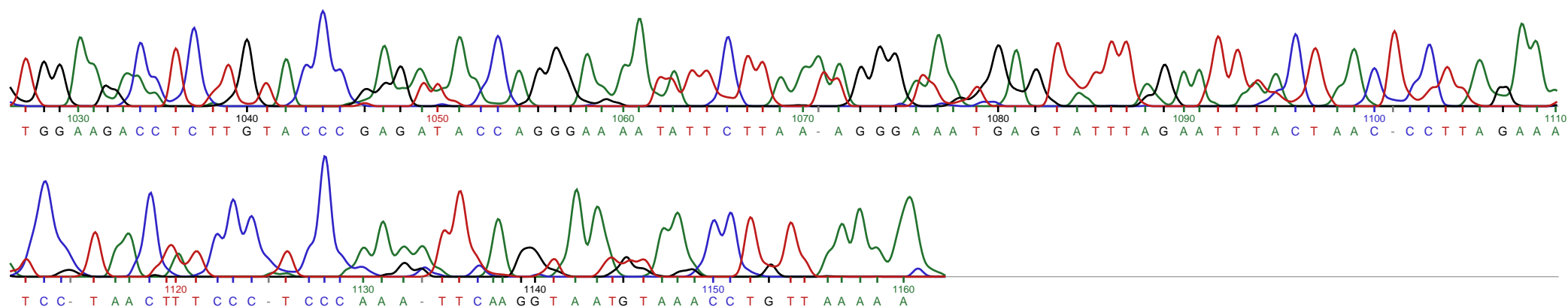

Supplement: Supporting information 2 — (ZIP) [file pone.0316479.s002.zip › 006KN2F_PREMIX_Plate_KELCH1_G01.pdf]

Page: 1 / 3  
8/17/2022

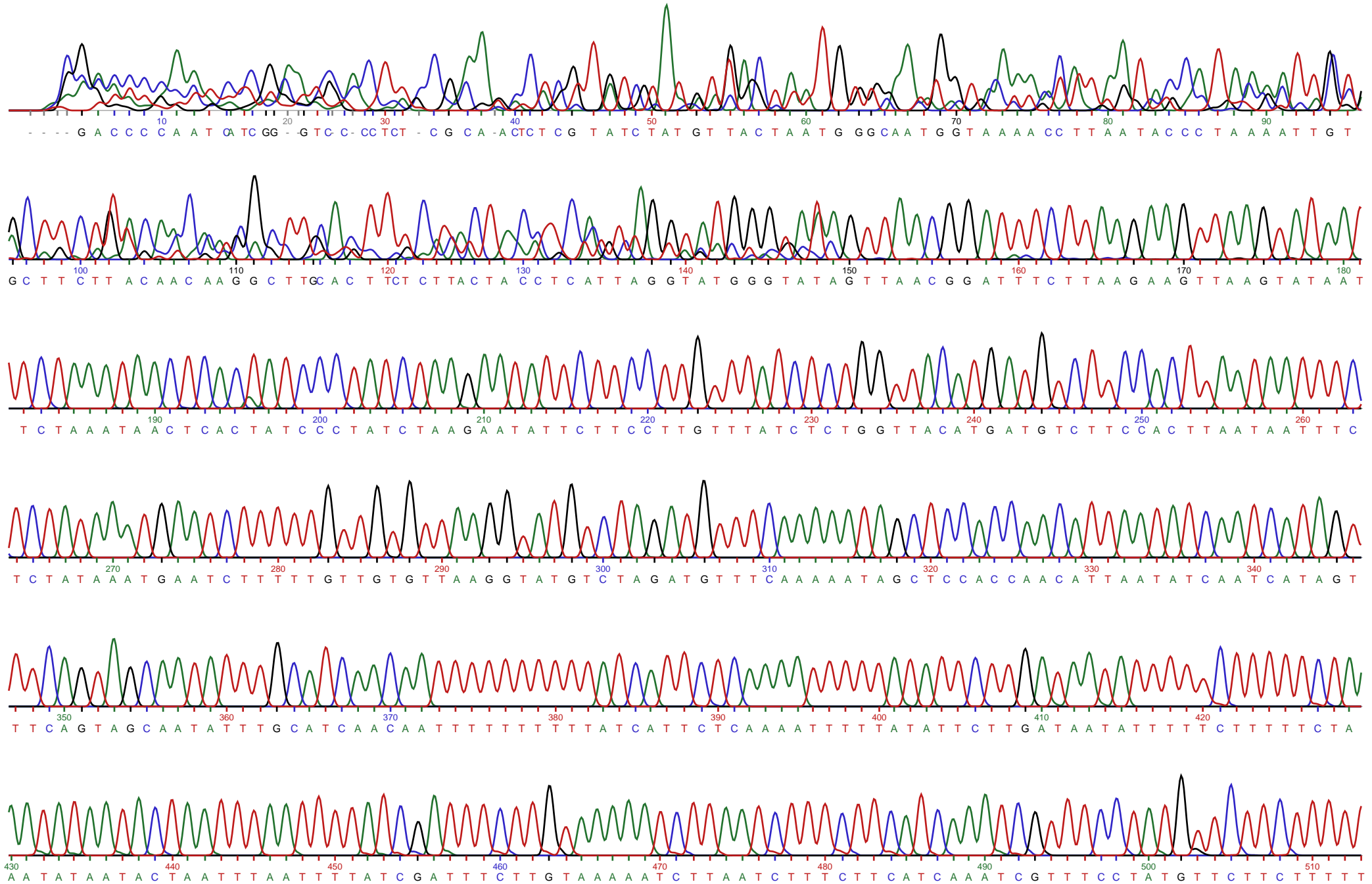

Samples: 13189  
Bases: 1120  
Average spacing: 12

Page: 2 / 3  
8/17/2022

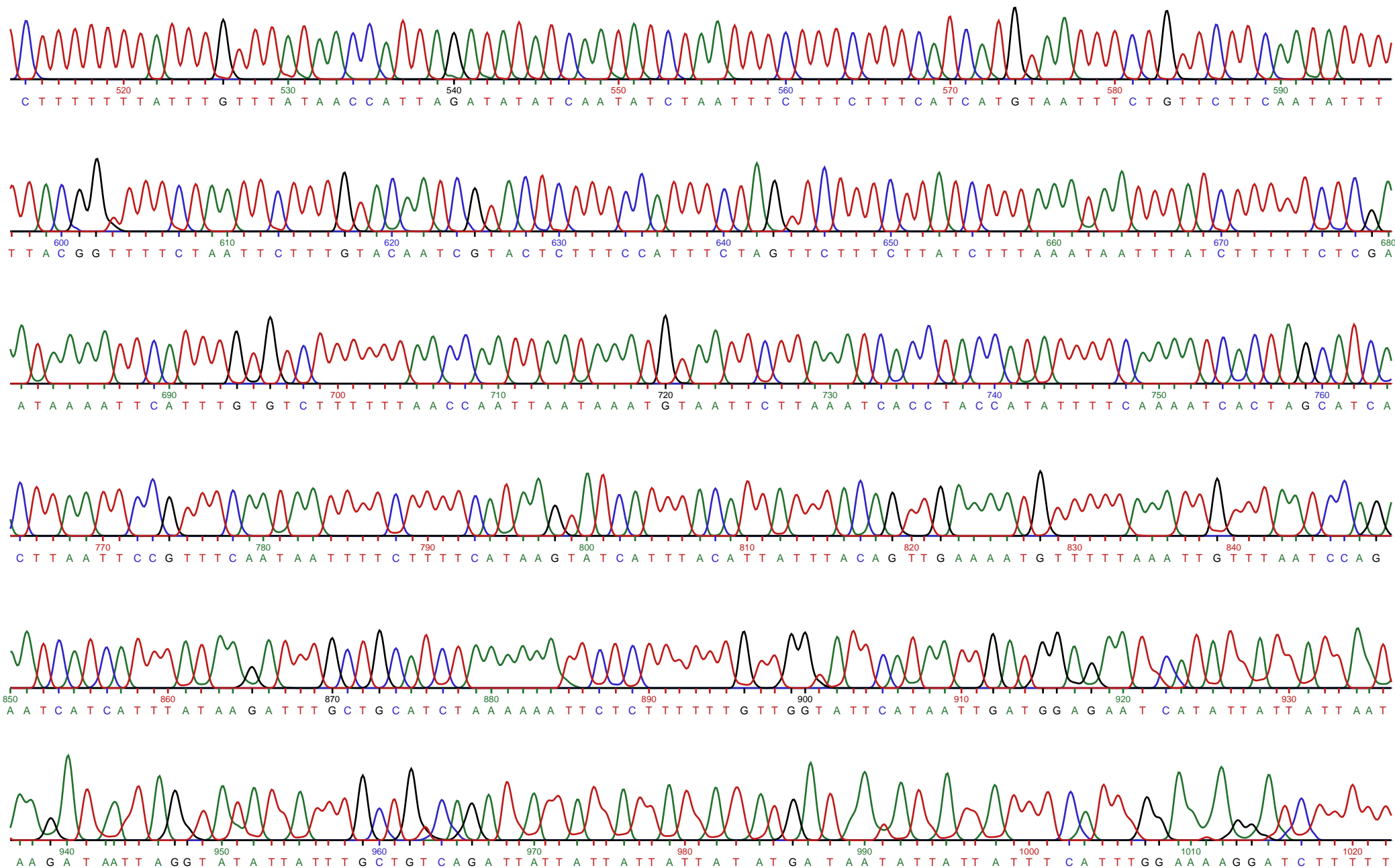

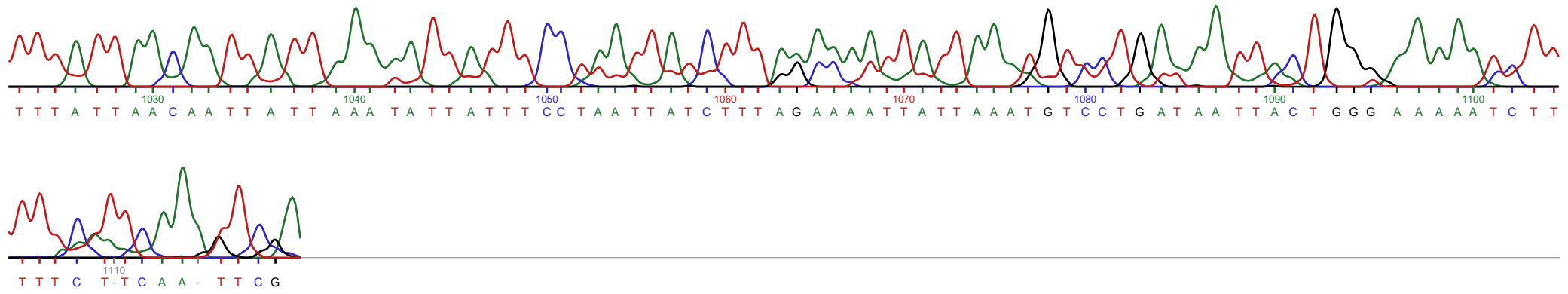

Supplement: Supporting information 2 — (ZIP) [file pone.0316479.s002.zip › 006KN2R_PREMIX_Plate_KELCH2_D03.pdf]

Page: 1 / 3  
8/17/2022

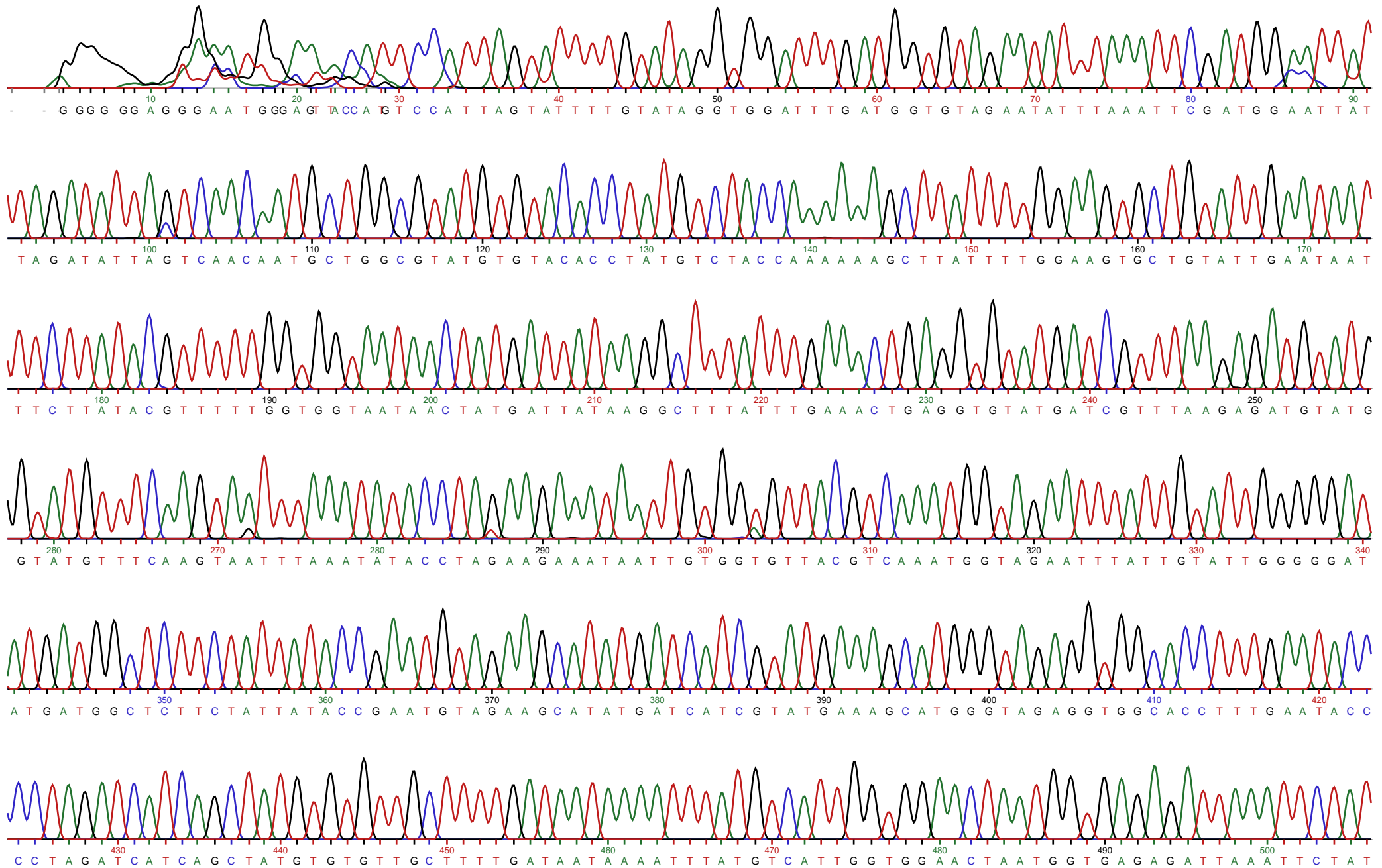

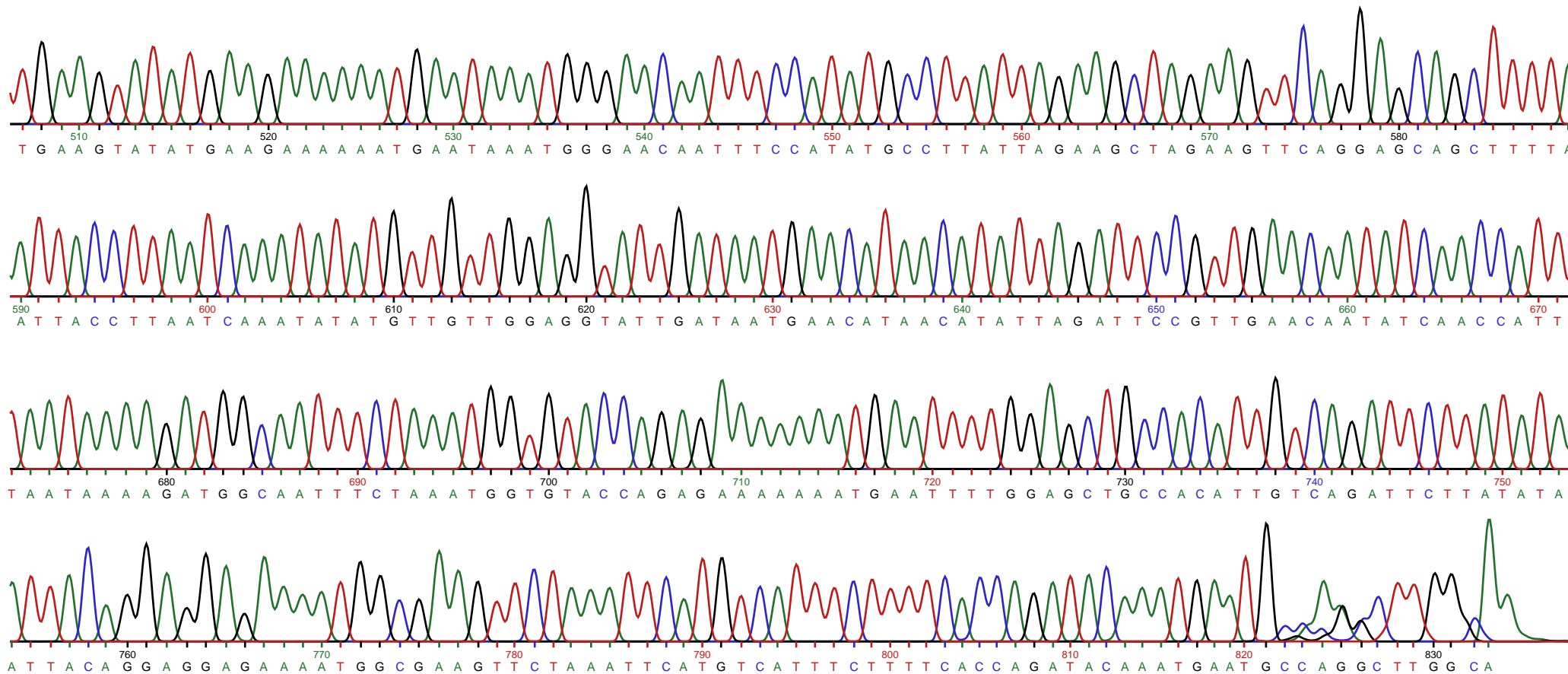

Samples: 12565  
Bases: 834  
Average spacing: 16

Page: 3 / 3  
8/17/2022

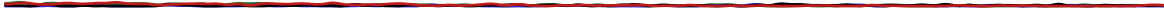

Supplement: Supporting information 2 — (ZIP) [file pone.0316479.s002.zip › 006KNIFW_PREMIX_Plate_CORKELCH_D11.pdf]

Page: 1 / 3  
8/17/2022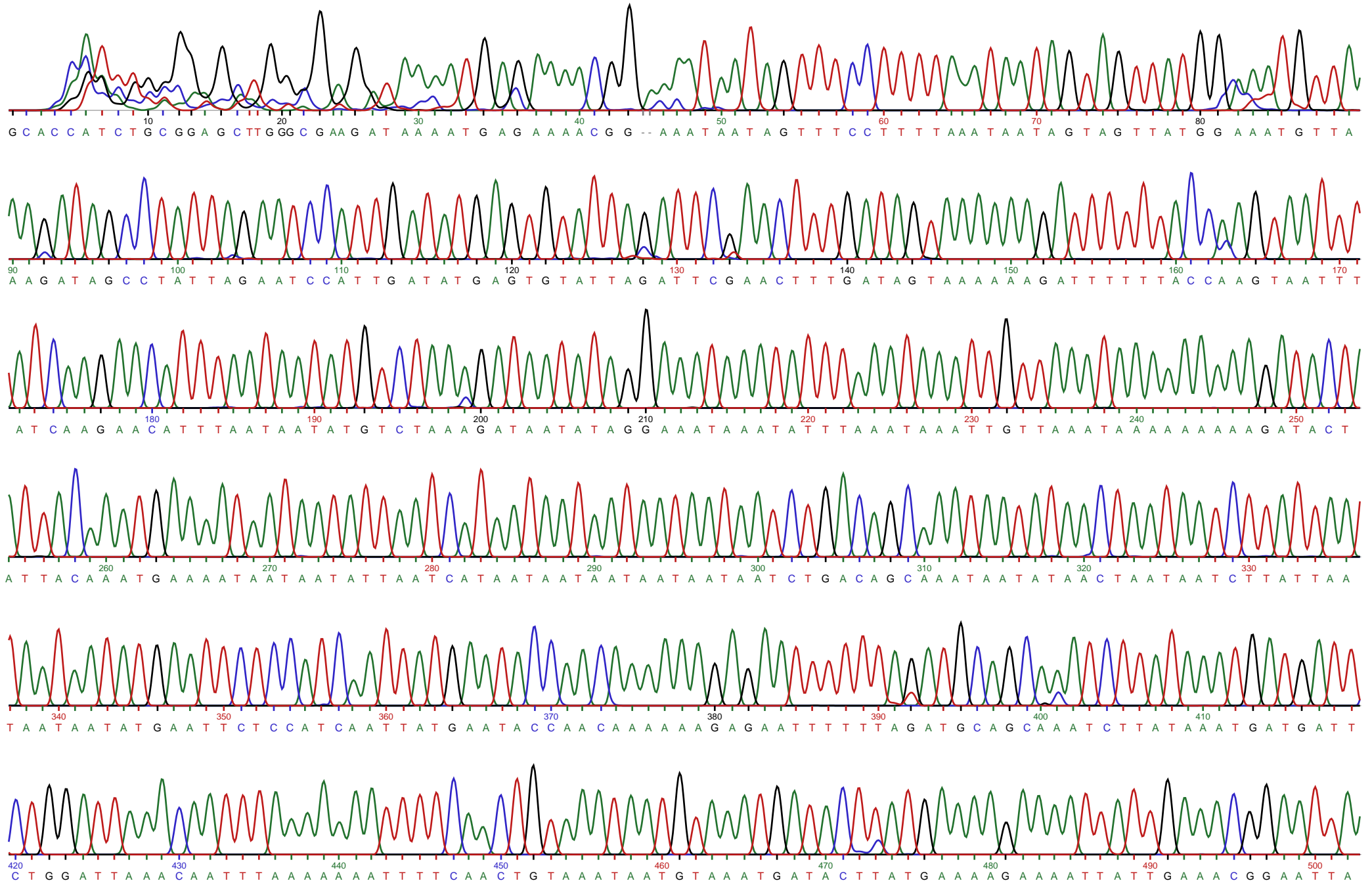

Samples: 13988  
Bases: 1174  
Average spacing: 12

Page: 2 / 3  
8/17/2022

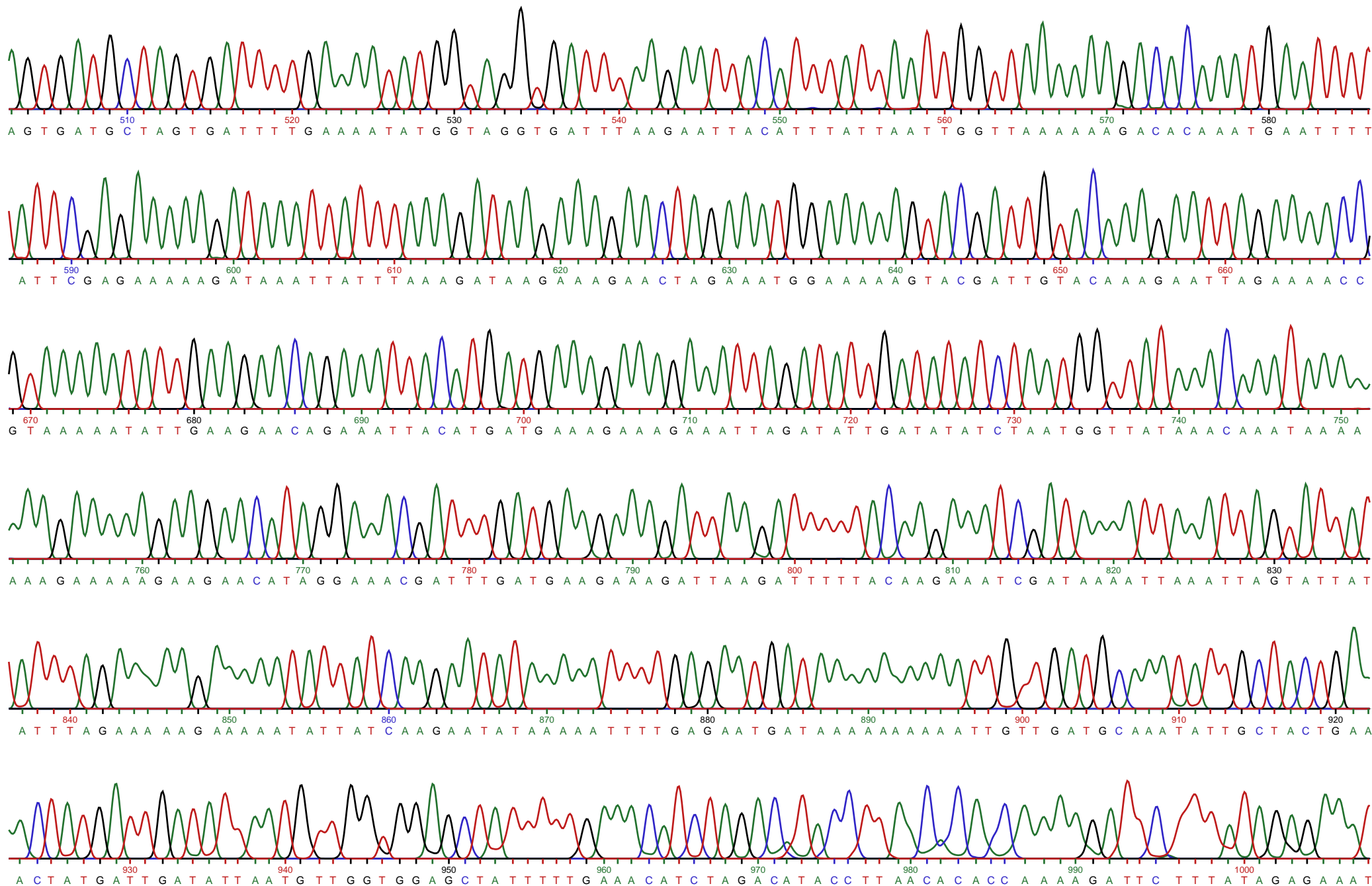

Samples: 13988  
Bases: 1174  
Average spacing: 12

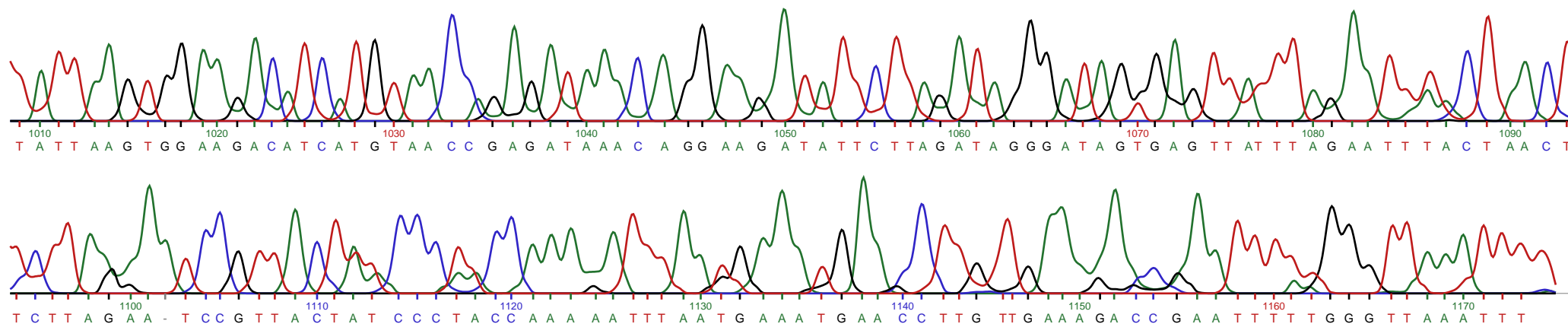

Supplement: Supporting information 2 — (ZIP) [file pone.0316479.s002.zip › 008KN2F_PREMIX_Plate_KELCH1_G02.pdf]

Samples: 13685  
Bases: 1147  
Average spacing: 12

Page: 1 / 3  
8/17/2022

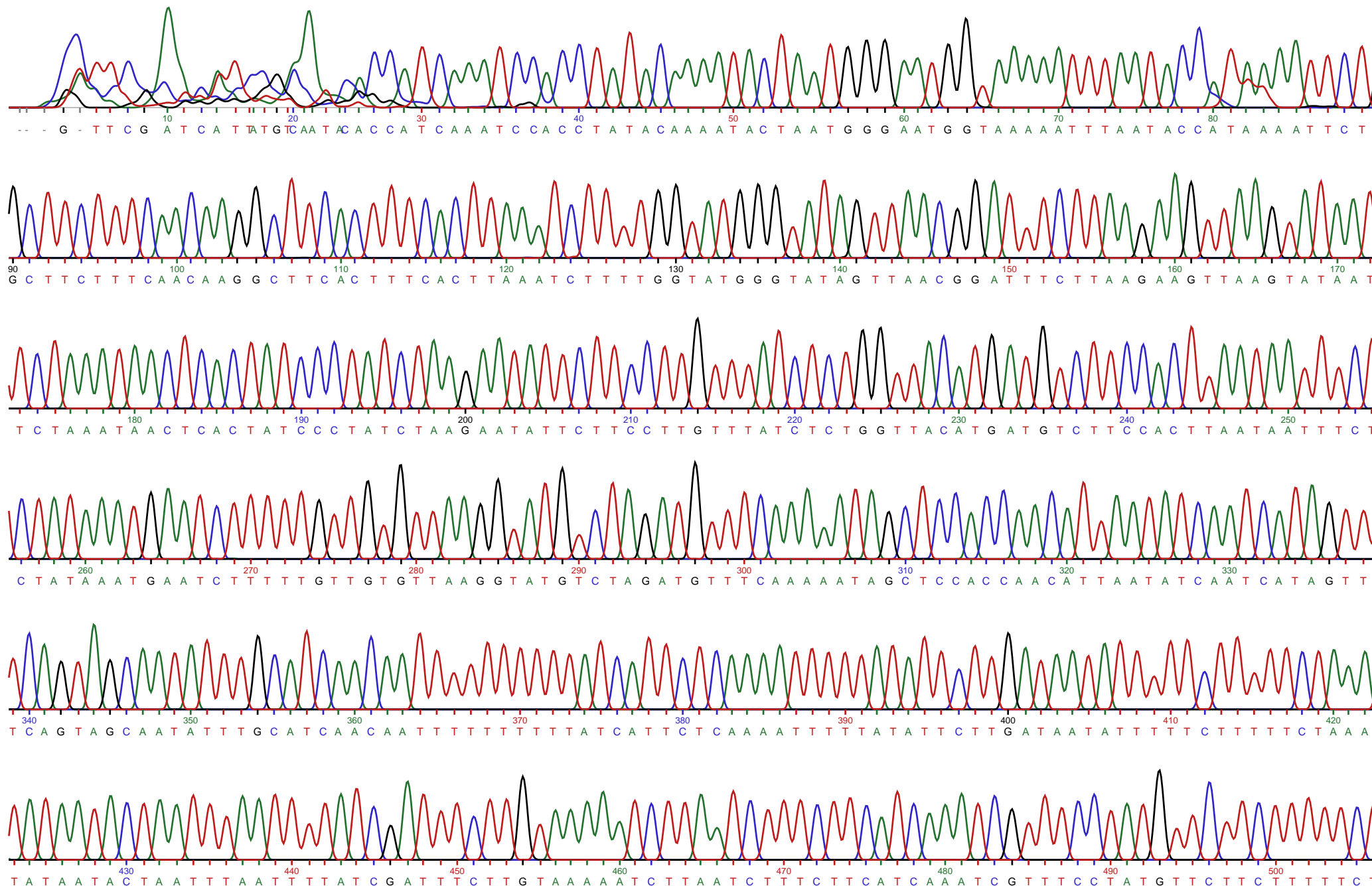

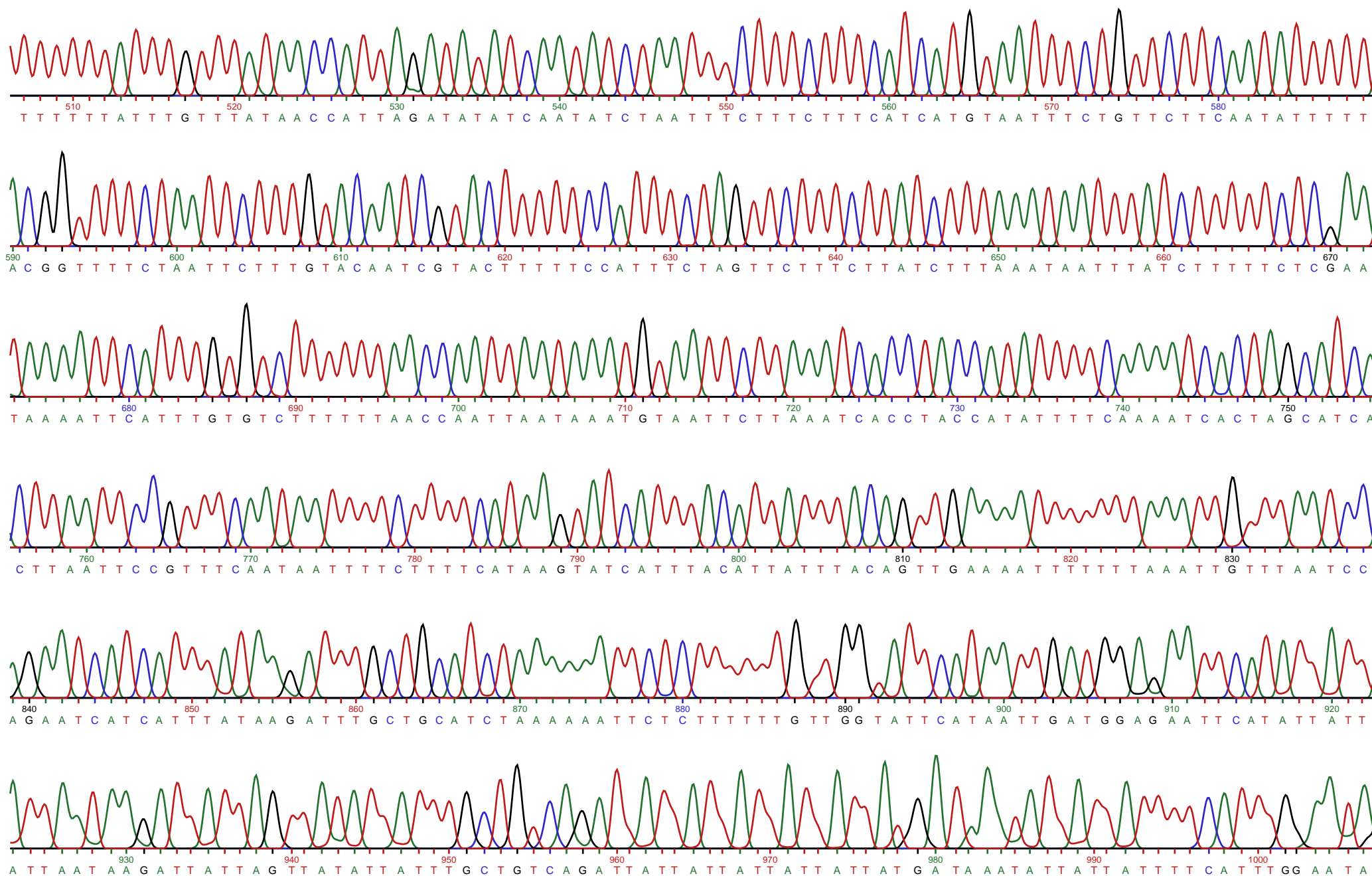

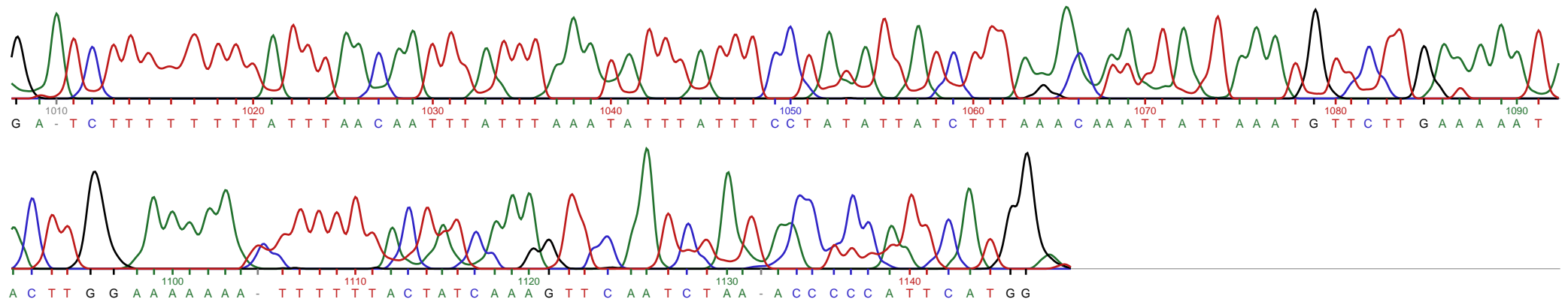

Supplement: Supporting information 2 — (ZIP) [file pone.0316479.s002.zip › 008KN2R_PREMIX_Plate_KELCH2_D04.pdf]

Samples: 12862  
Bases: 820  
Average spacing: 16

Page: 1 / 3  
8/17/2022

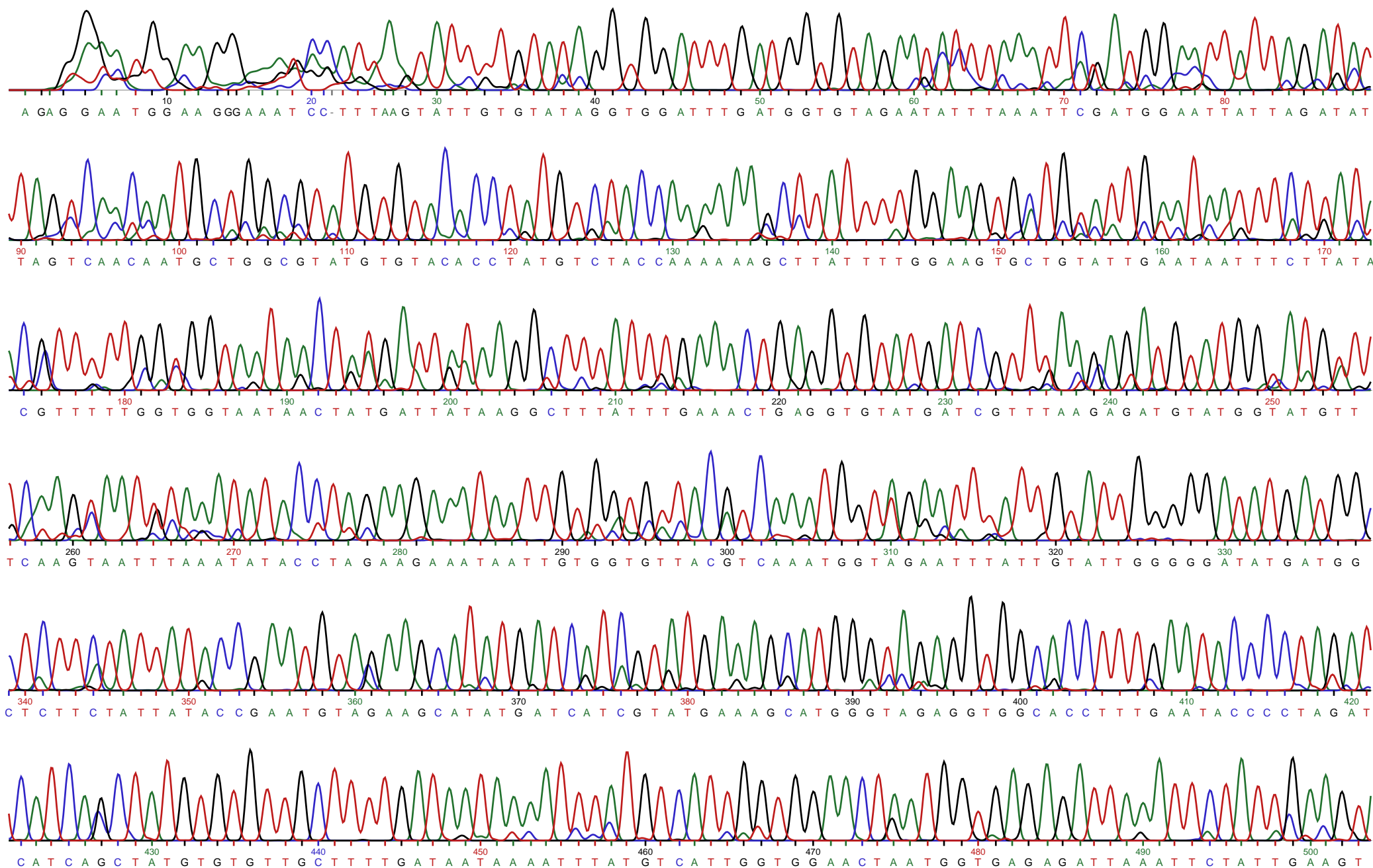

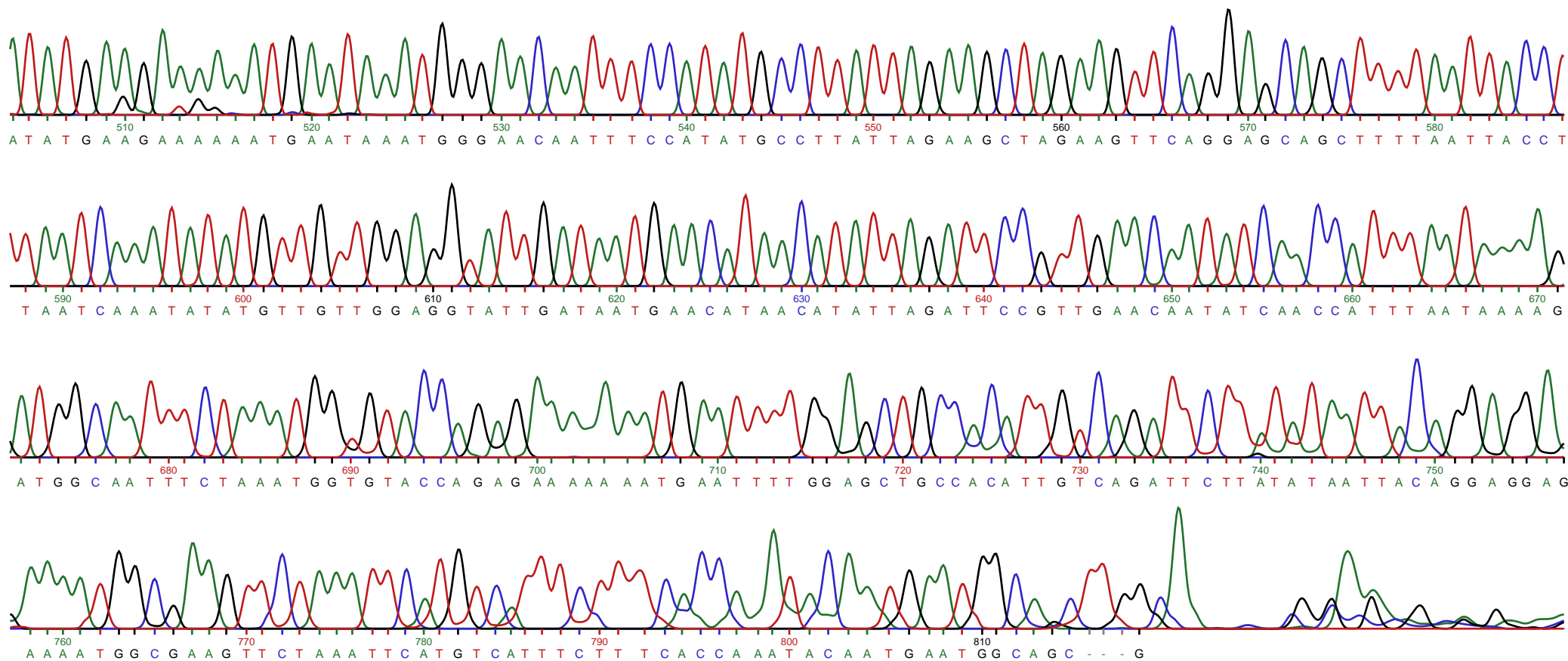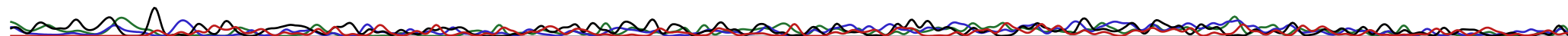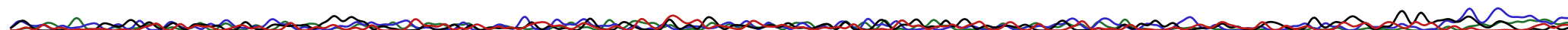

Samples: 12862  
Bases: 820  
Average spacing: 16

Page: 3 / 3  
8/17/2022

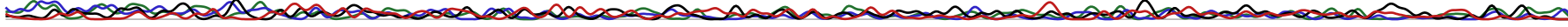

Supplement: Supporting information 2 — (ZIP) [file pone.0316479.s002.zip › 008KNIFW_PREMIX_Plate_CORKELCH_D12.pdf]

Samples: 14001  
Bases: 824  
Average spacing: 17

Page: 1 / 3  
8/17/2022

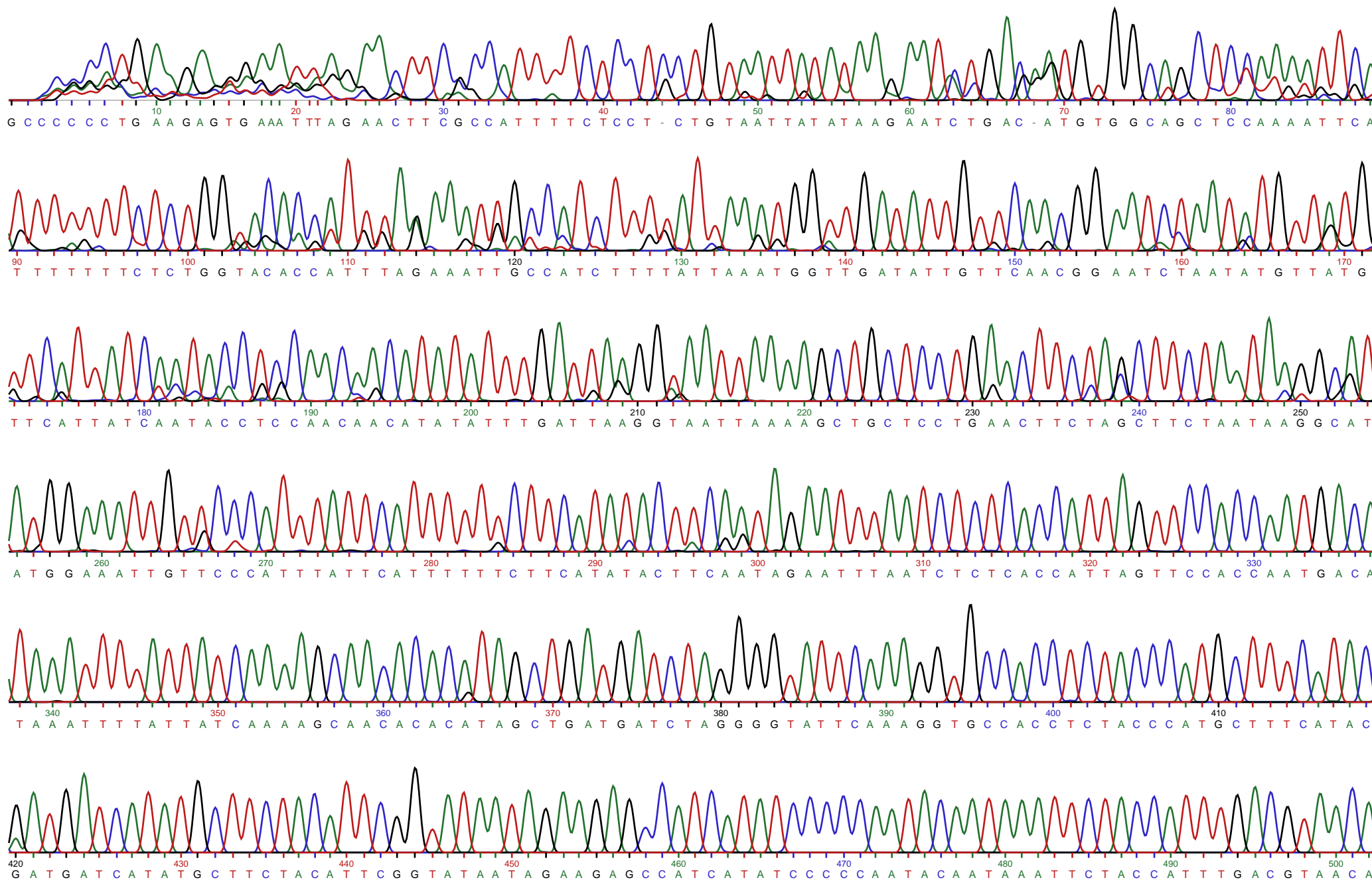

Samples: 14001  
Bases: 824  
Average spacing: 17

Page: 2 / 3  
8/17/2022

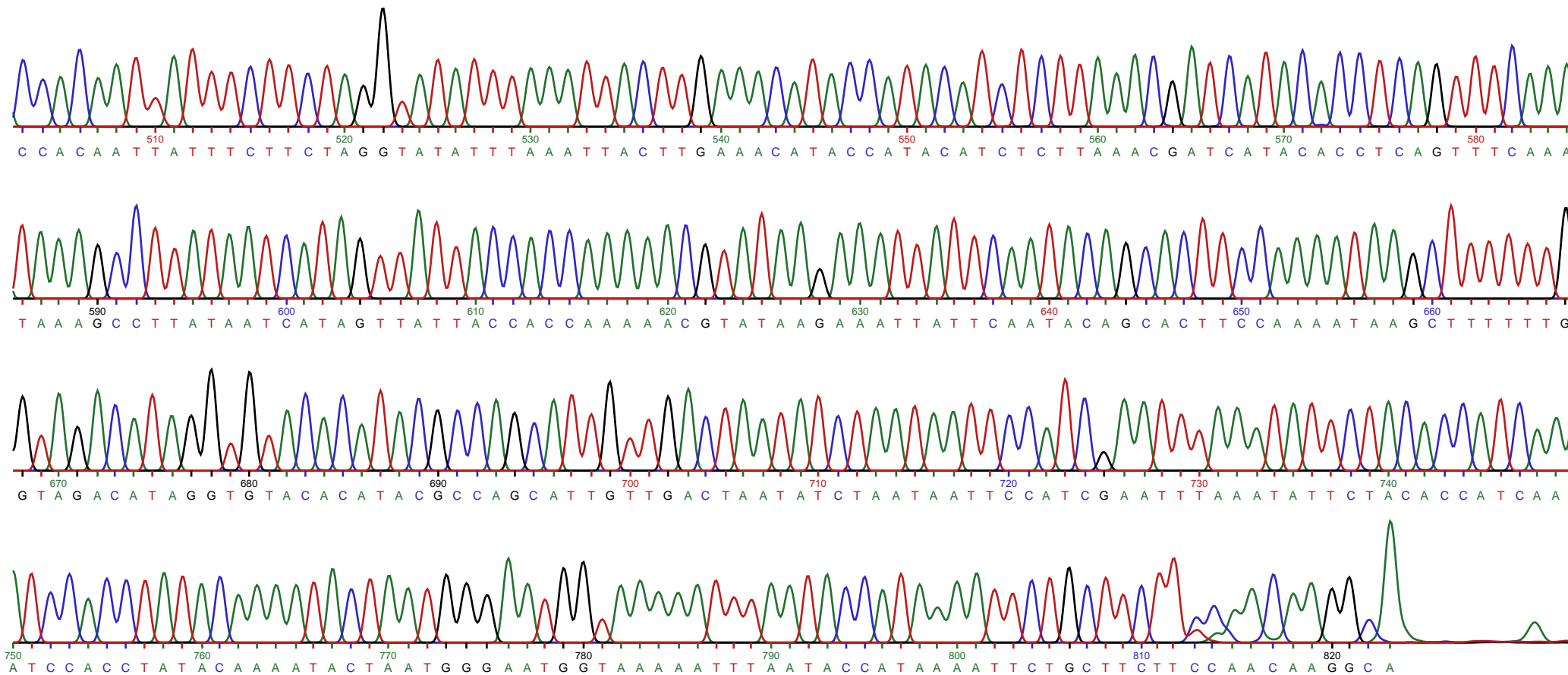

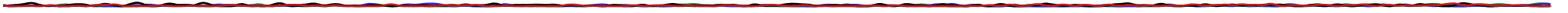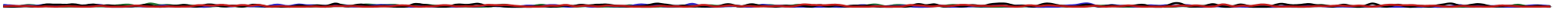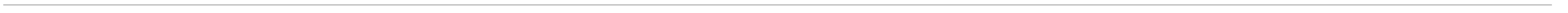

Supplement: Supporting information 2 — (ZIP) [file pone.0316479.s002.zip › 010KN1R_PREMIX_Plate_KELCH1_B03.pdf]

Page: 1 / 3  
8/17/2022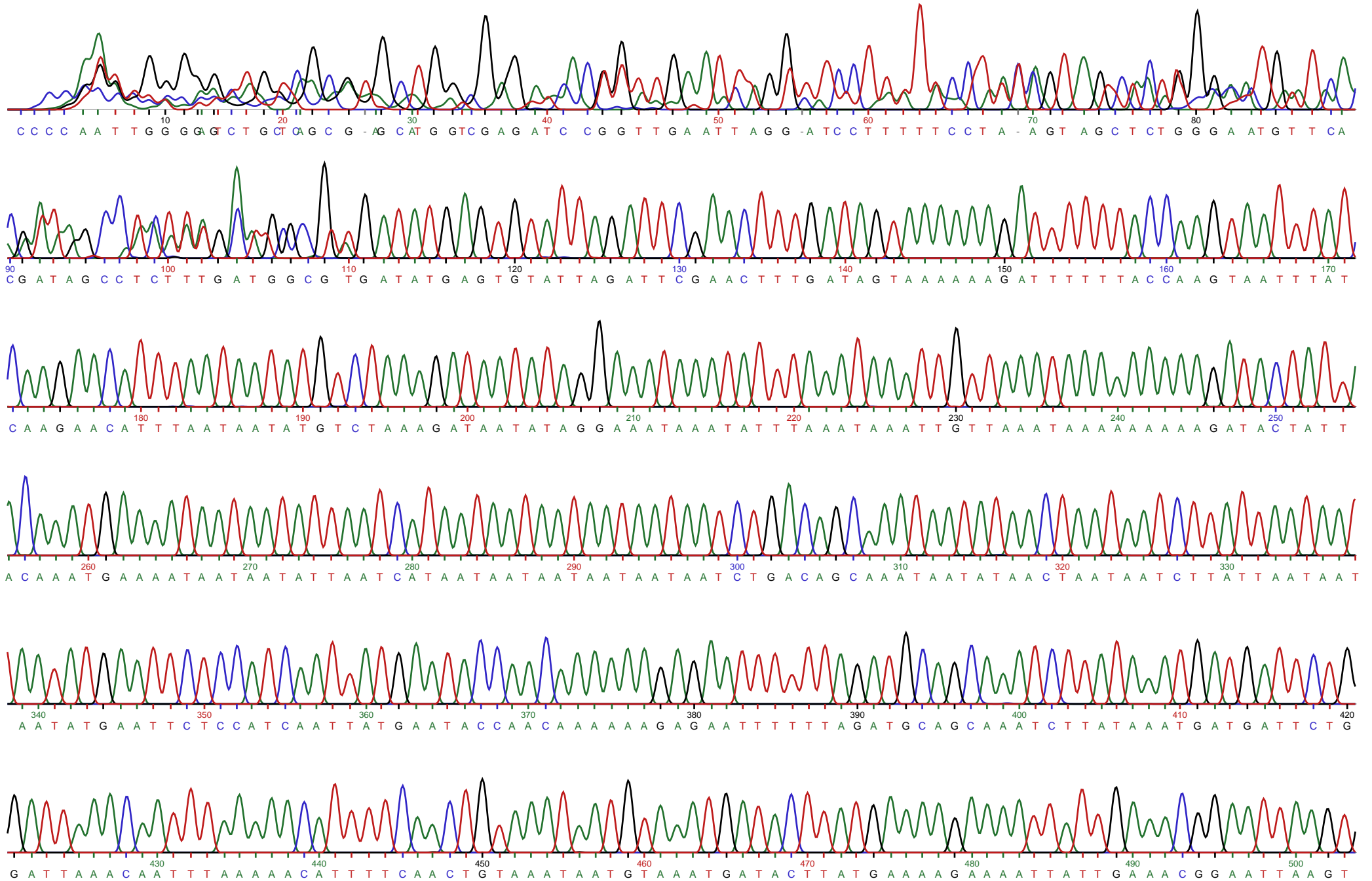

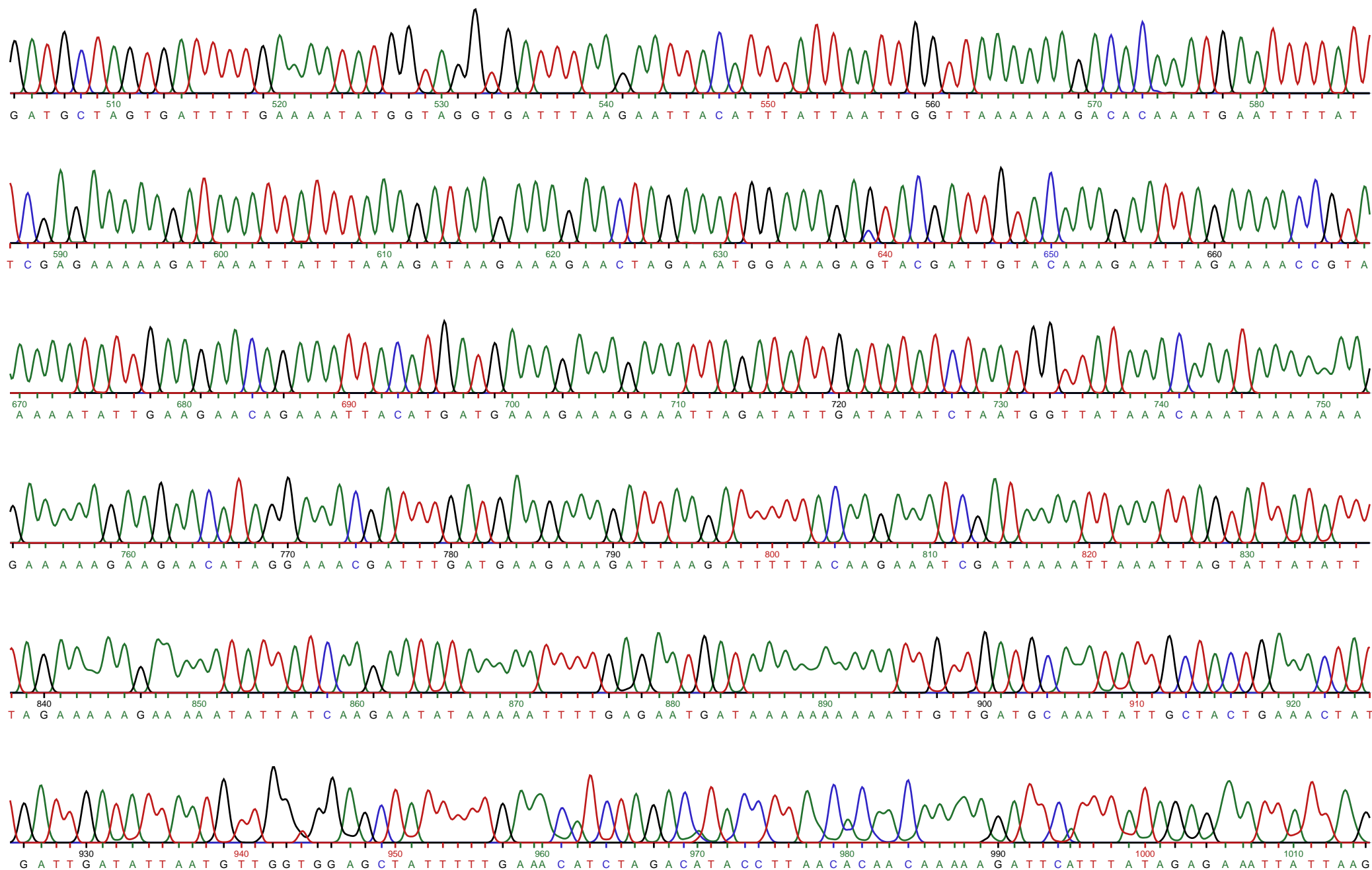

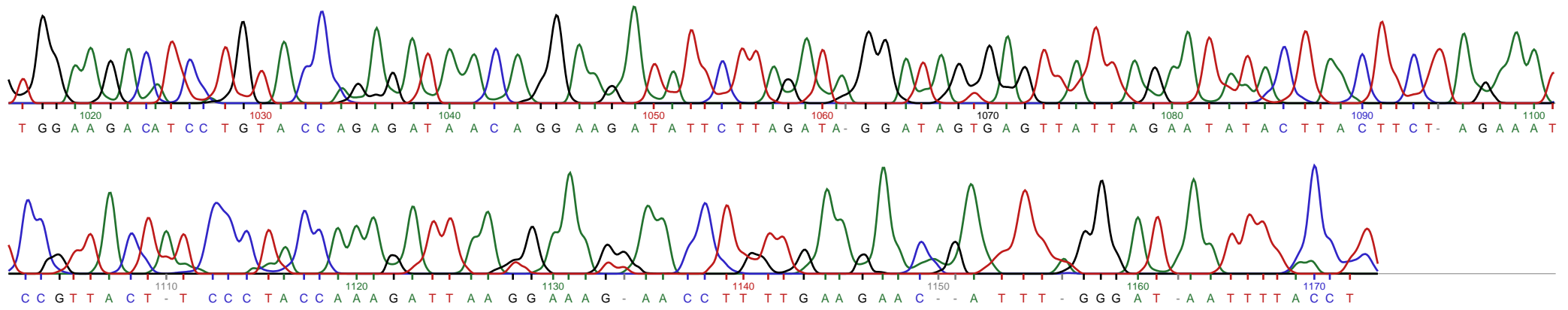

Supplement: Supporting information 2 — (ZIP) [file pone.0316479.s002.zip › 010KN2F_PREMIX_Plate_KELCH1_G03.pdf]

Page: 1 / 3  
8/17/2022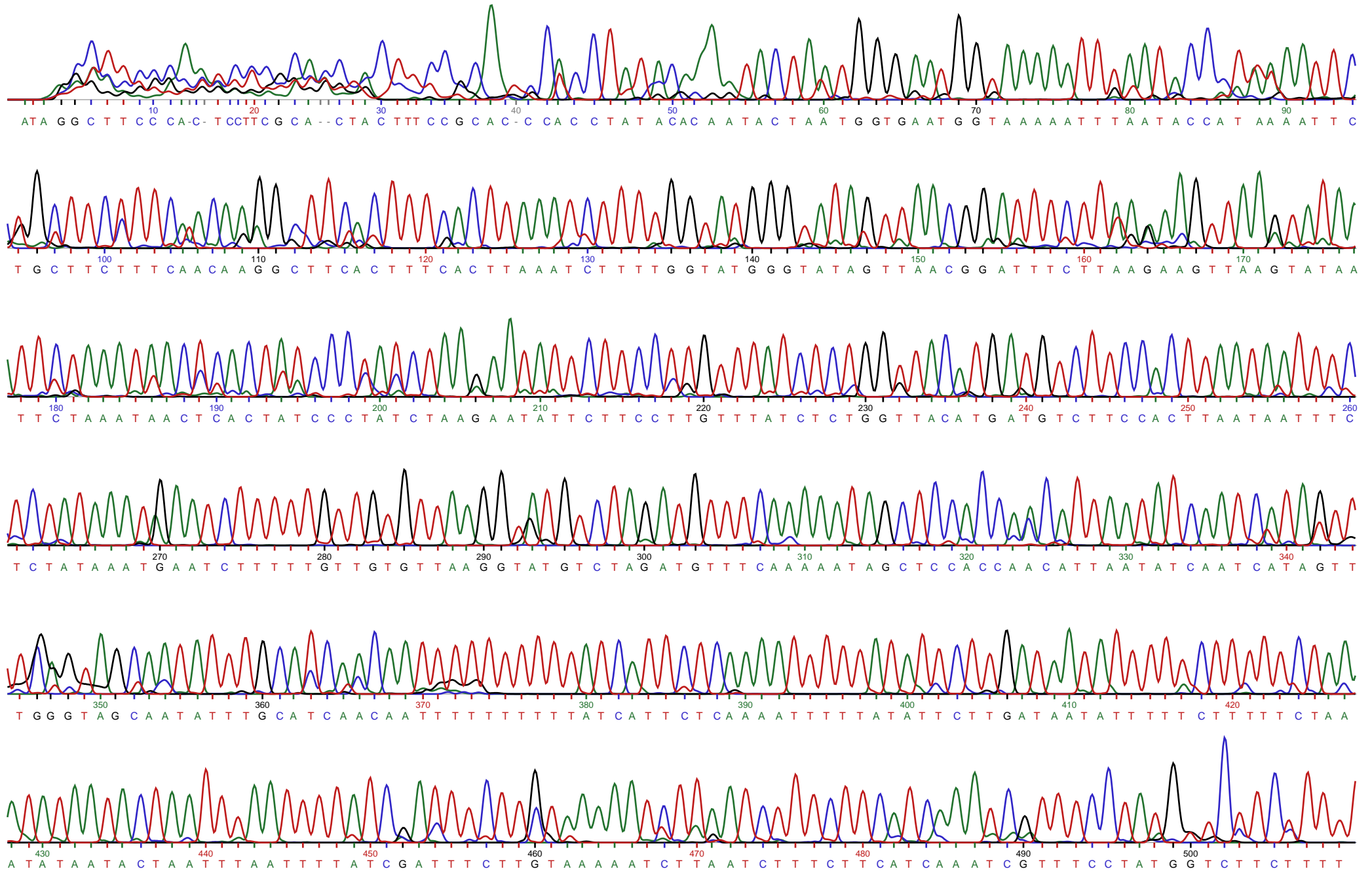

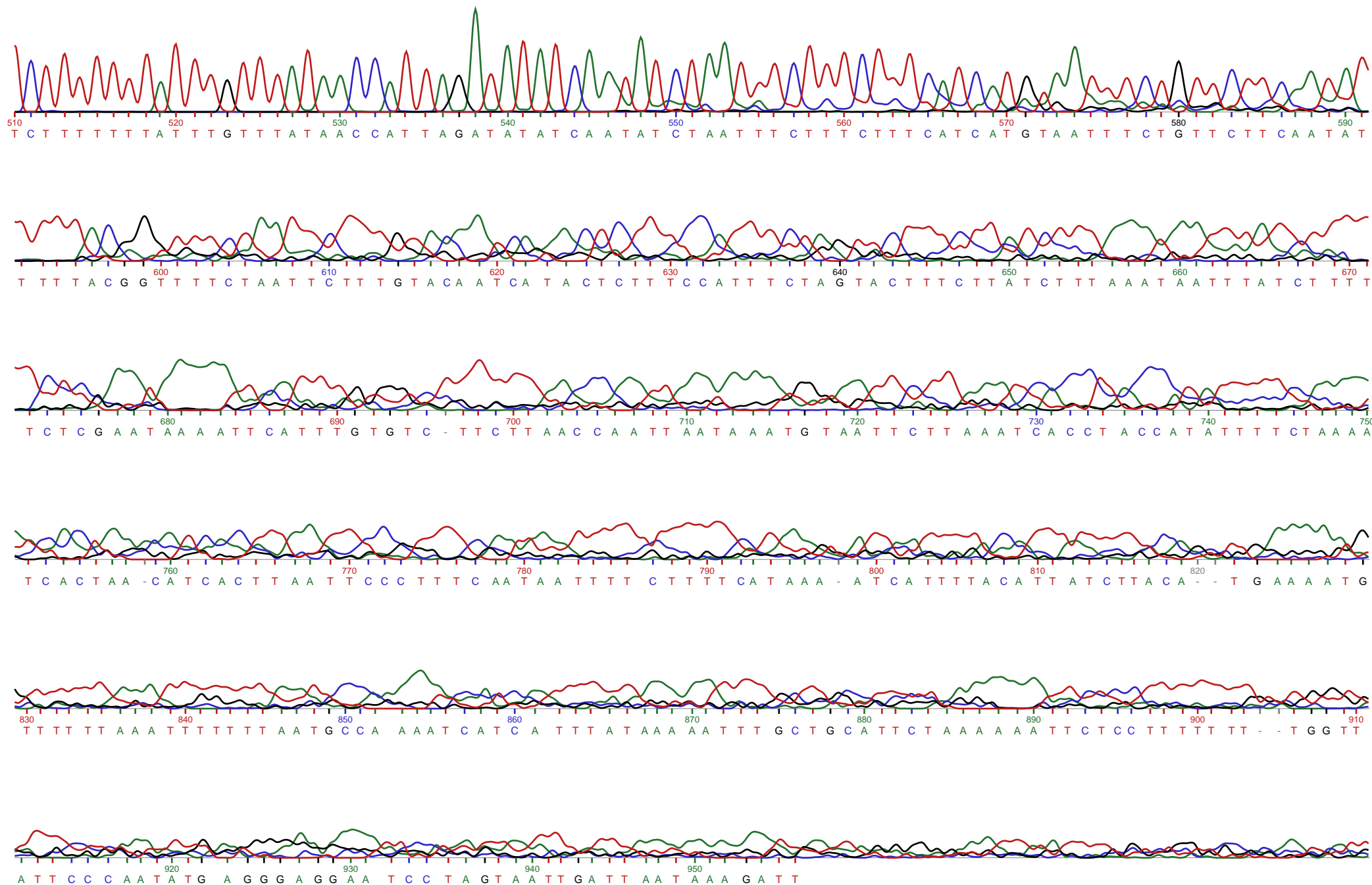

Samples: 14606  
Bases: 957  
Average spacing: 16

Page: 3 / 3  
8/17/2022

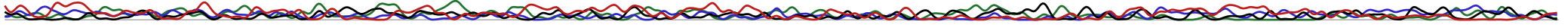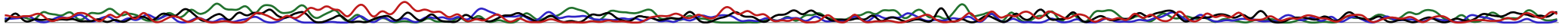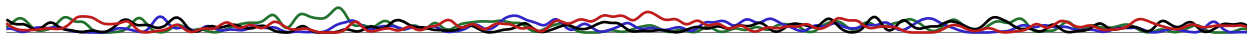

Supplement: Supporting information 2 — (ZIP) [file pone.0316479.s002.zip › 010KN2R_PREMIX_Plate_KELCH2_D05.pdf]

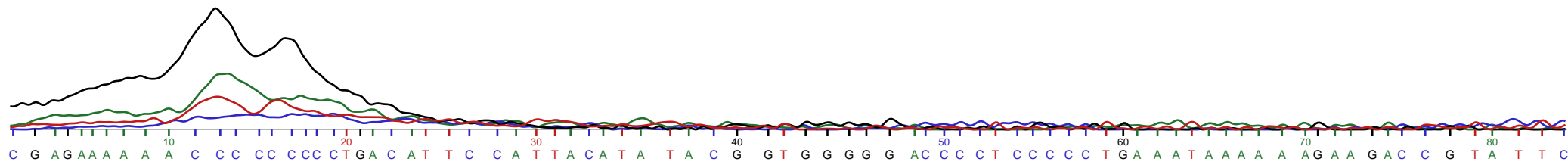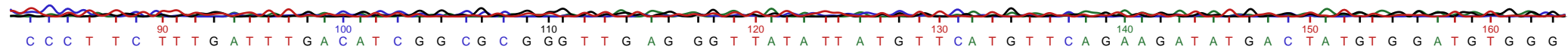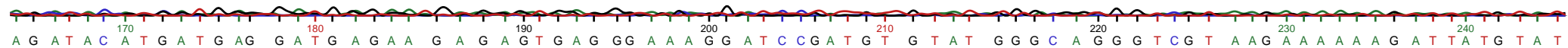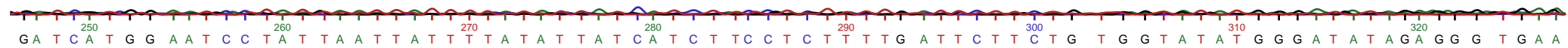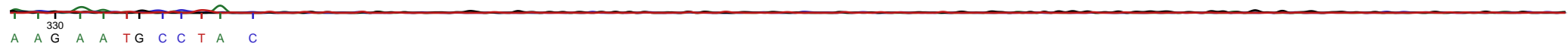

Samples: 14826  
Bases: 340  
Average spacing: 44

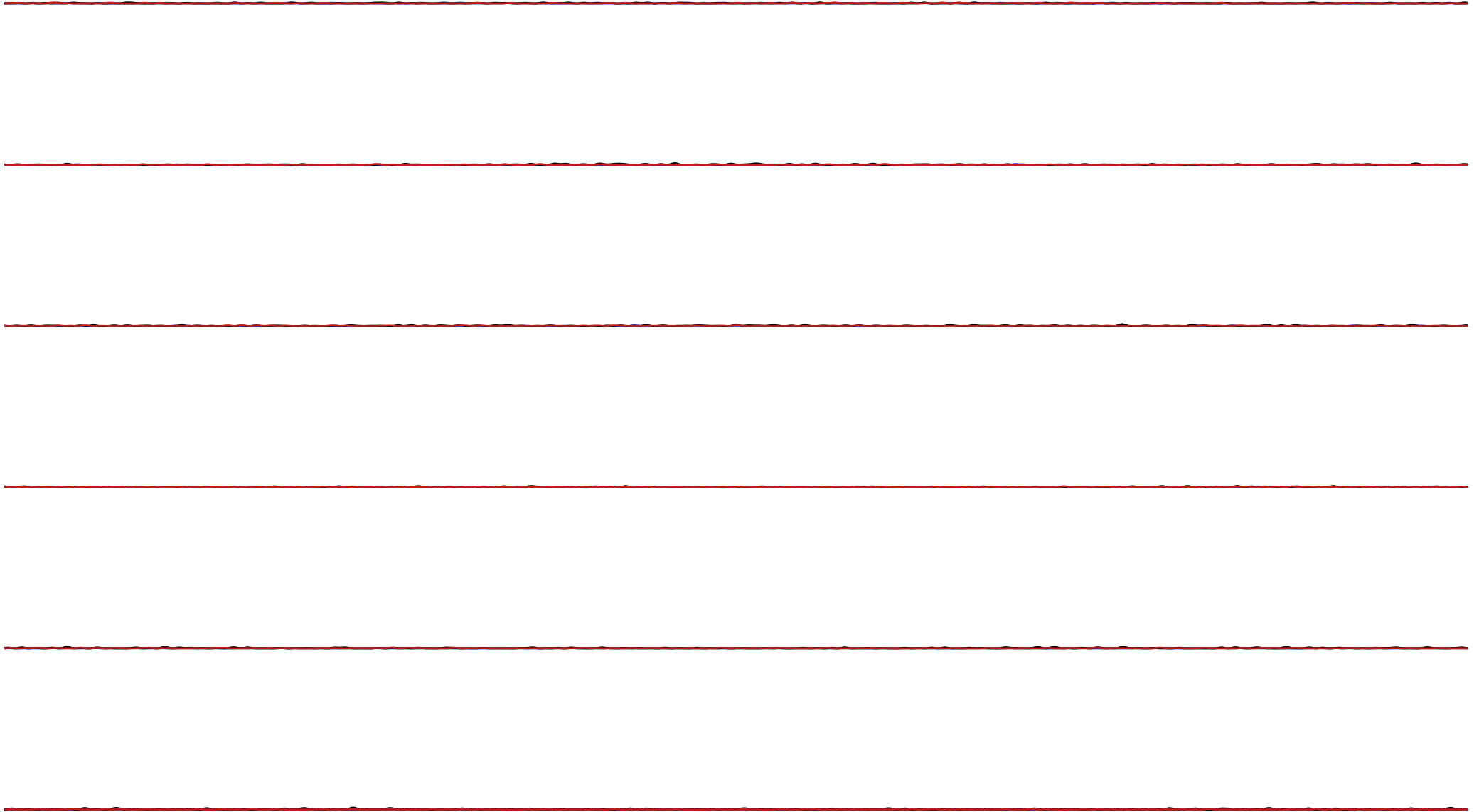

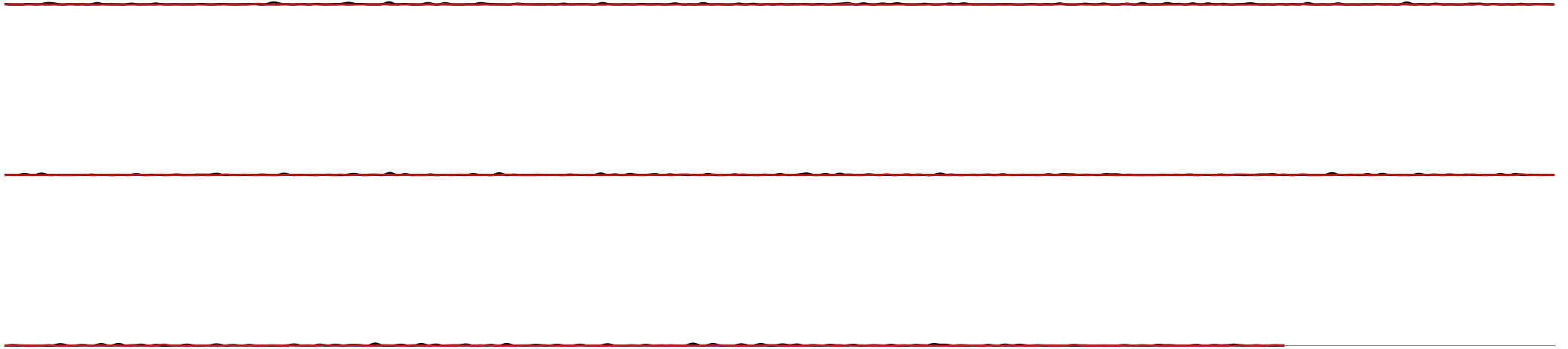

Supplement: Supporting information 2 — (ZIP) [file pone.0316479.s002.zip › 010KNIFW_PREMIX_Plate_CORKELCH_E01.pdf]

Samples: 14092  
Bases: 820  
Average spacing: 18

Page: 1 / 3  
8/17/2022

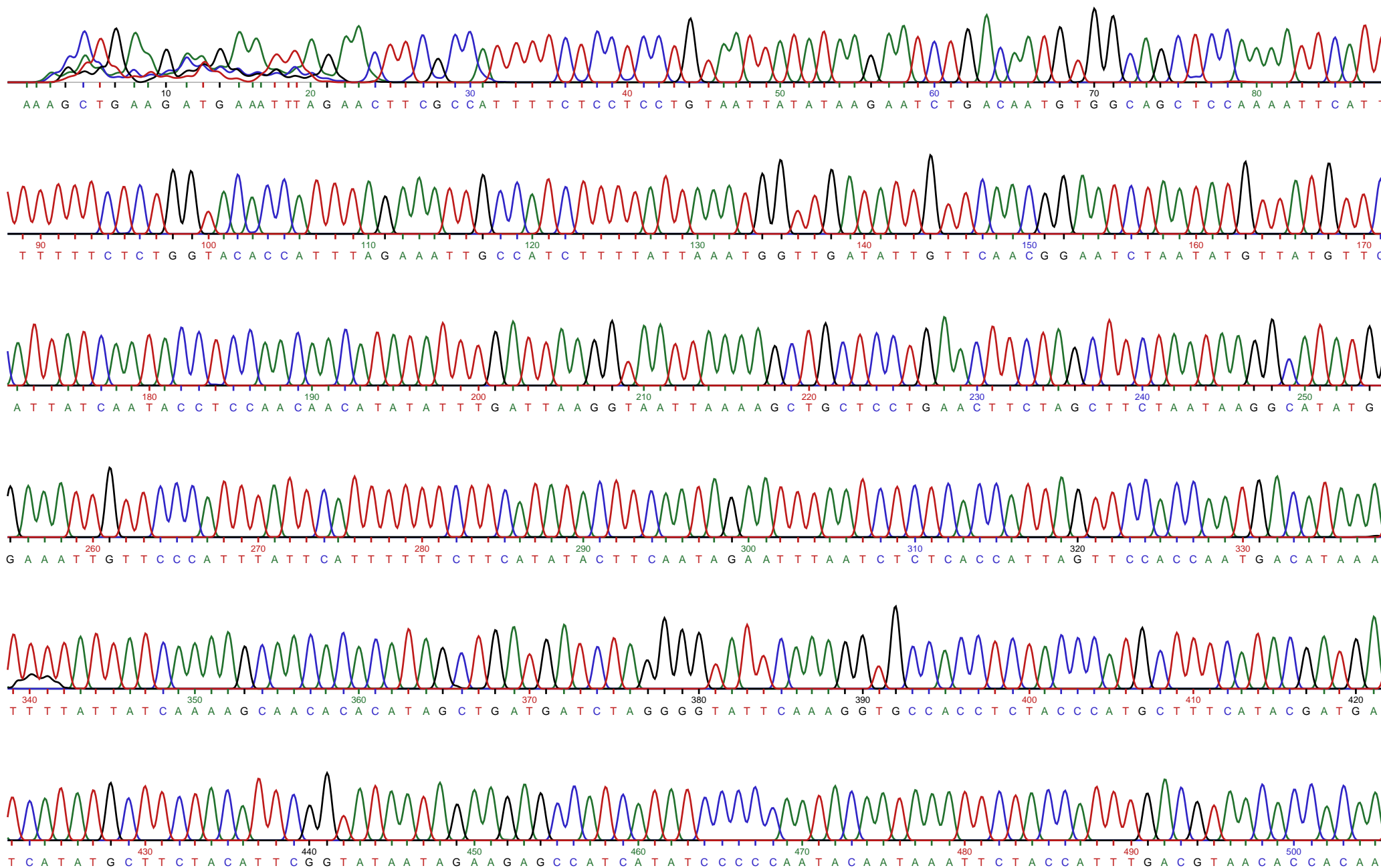

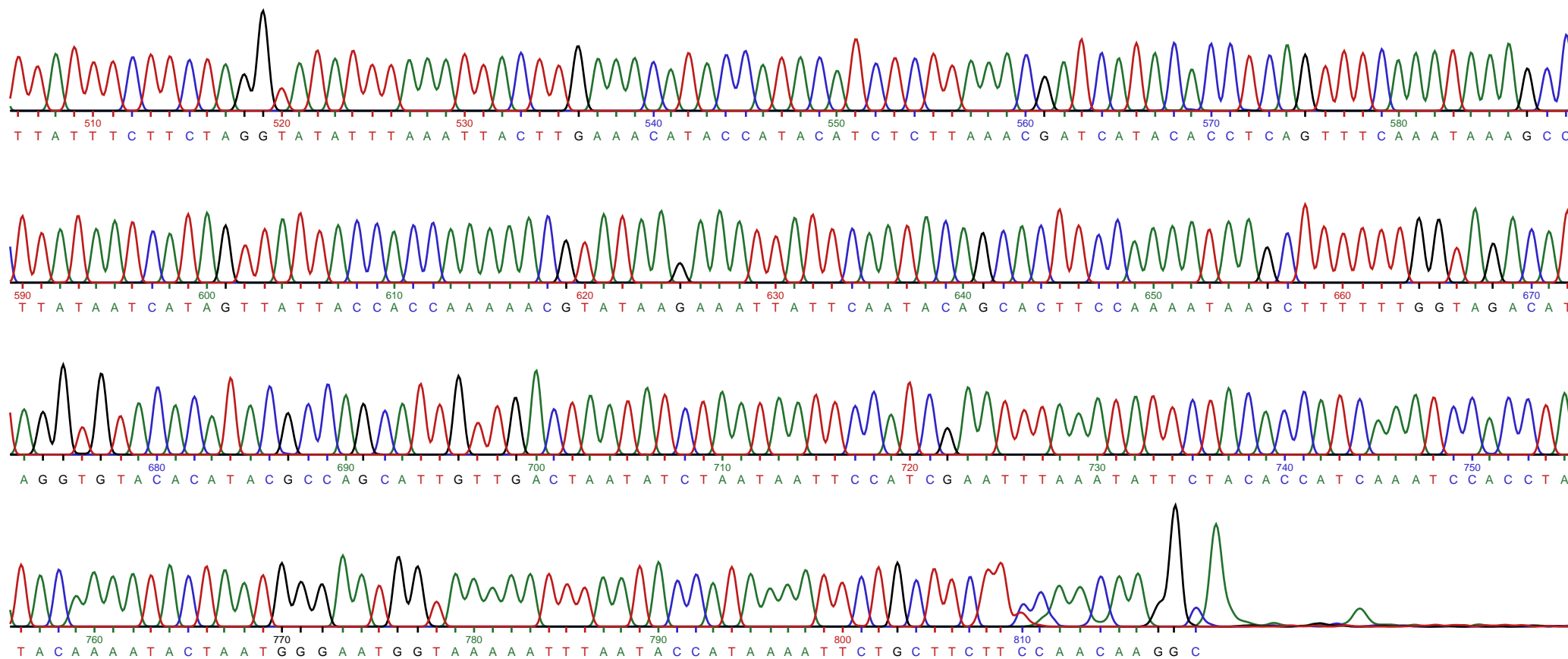

Samples: 14092  
Bases: 820  
Average spacing: 18

Page: 3 / 3  
8/17/2022

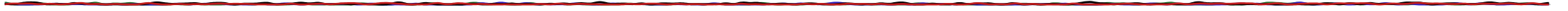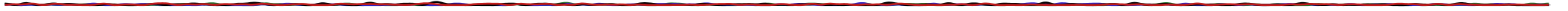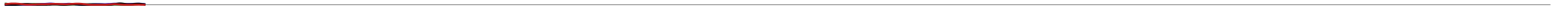

Supplement: Supporting information 2 — (ZIP) [file pone.0316479.s002.zip › 012KN1R_PREMIX_Plate_KELCH1_B04.pdf]

Page: 1 / 3  
8/17/2022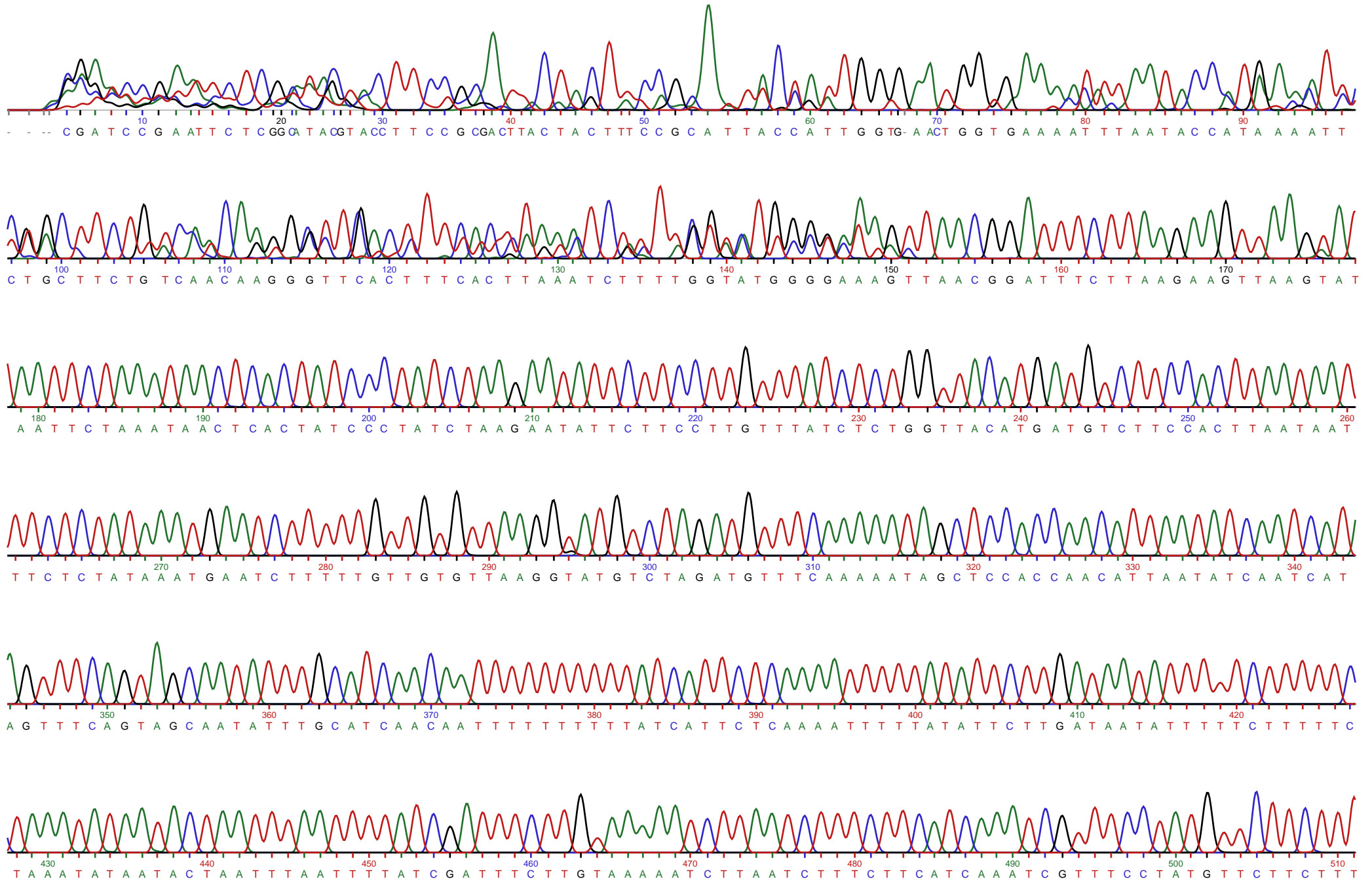

Page: 2 / 3  
8/17/2022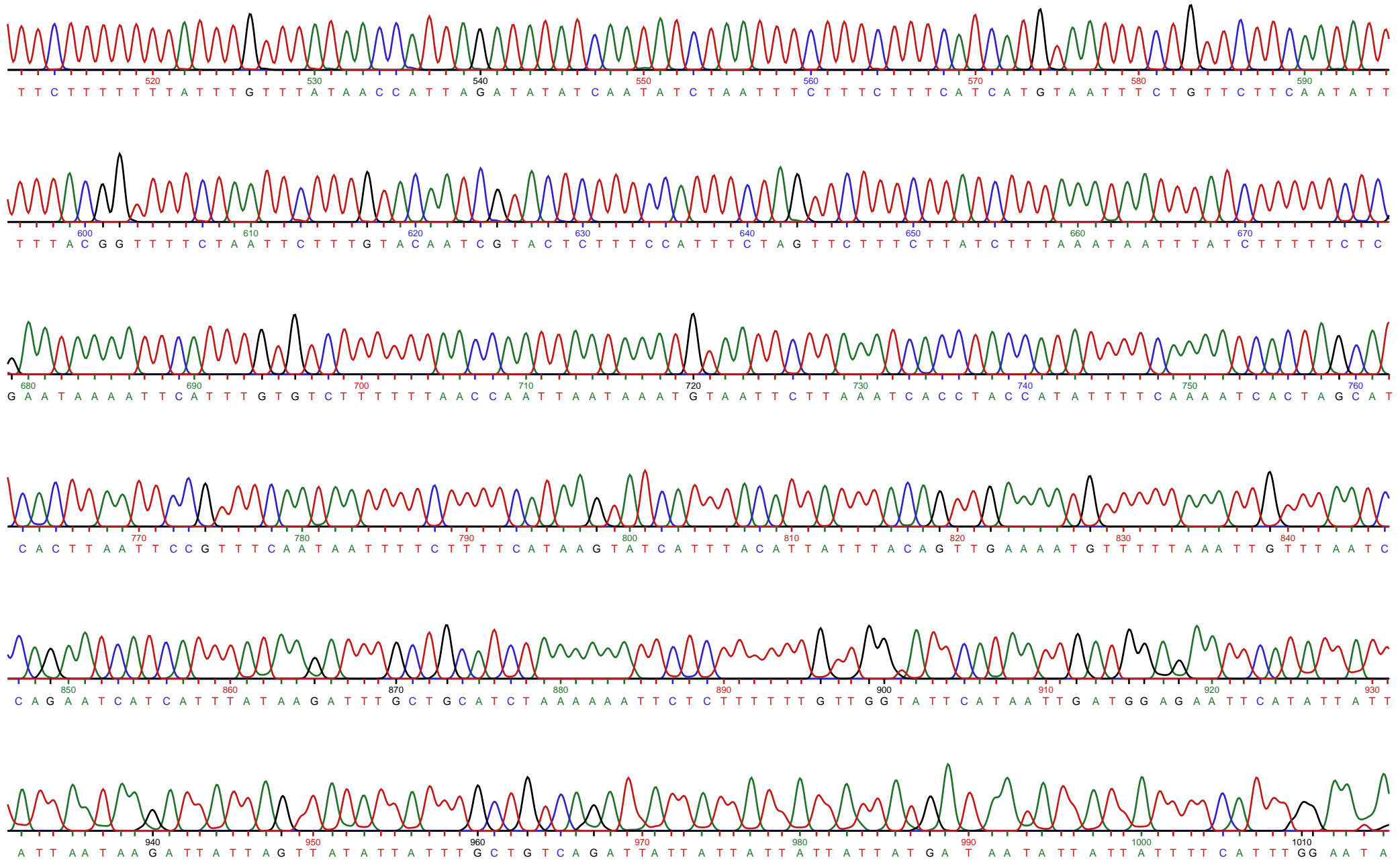

Samples: 13386  
Bases: 1130  
Average spacing: 12

Page: 3 / 3  
8/17/2022

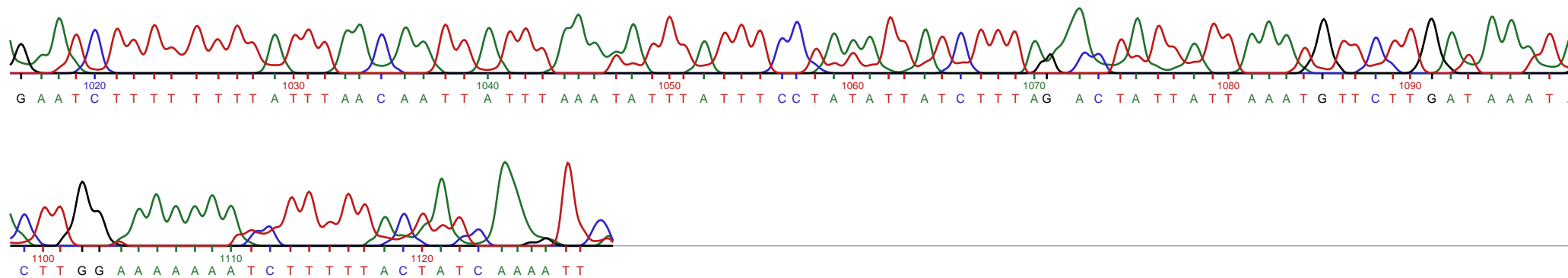

Supplement: Supporting information 2 — (ZIP) [file pone.0316479.s002.zip › 012KN2R_PREMIX_Plate_KELCH2_D06.pdf]

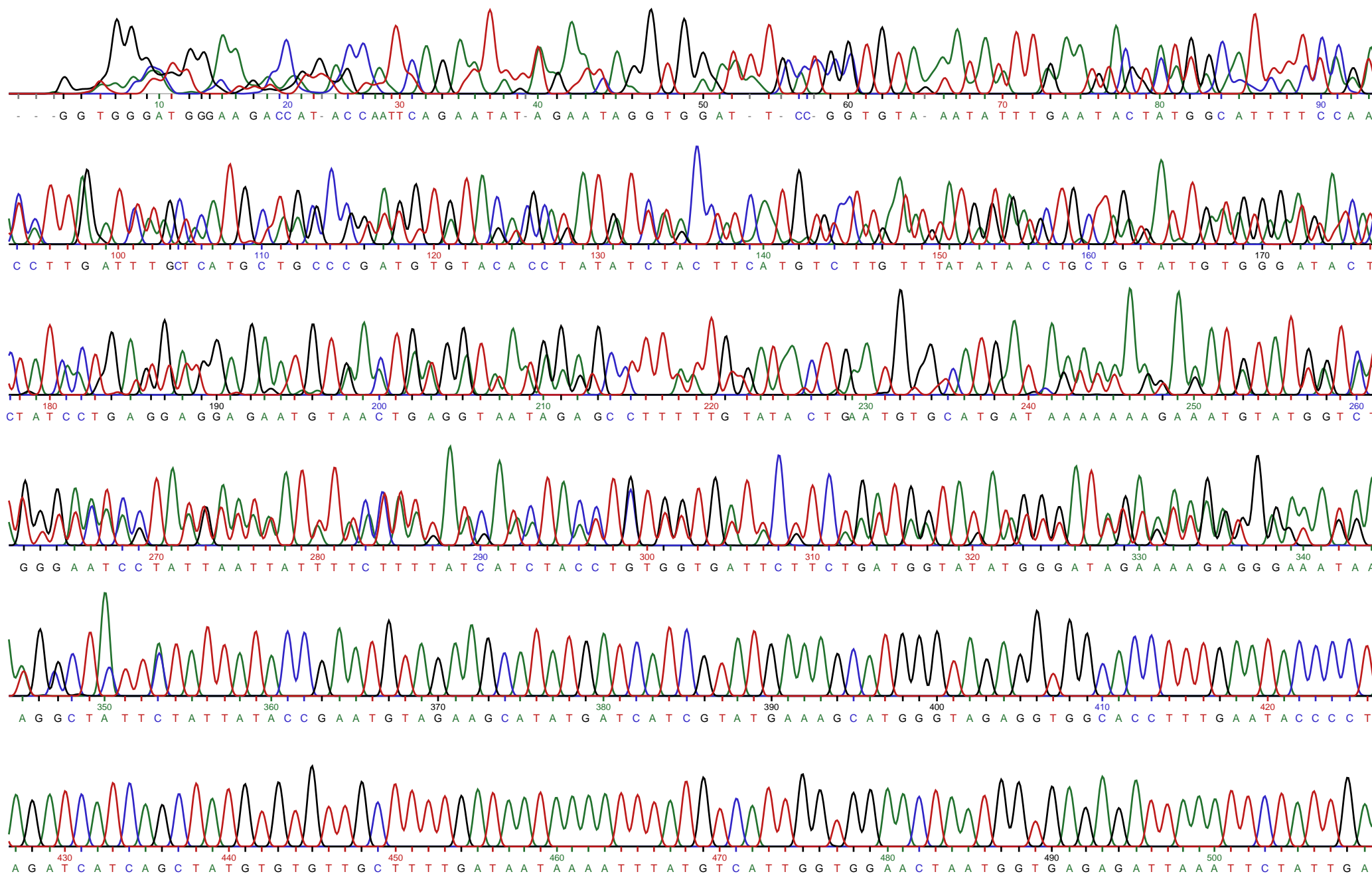

Samples: 12964  
Bases: 830  
Average spacing: 16

Page: 2 / 3  
8/17/2022

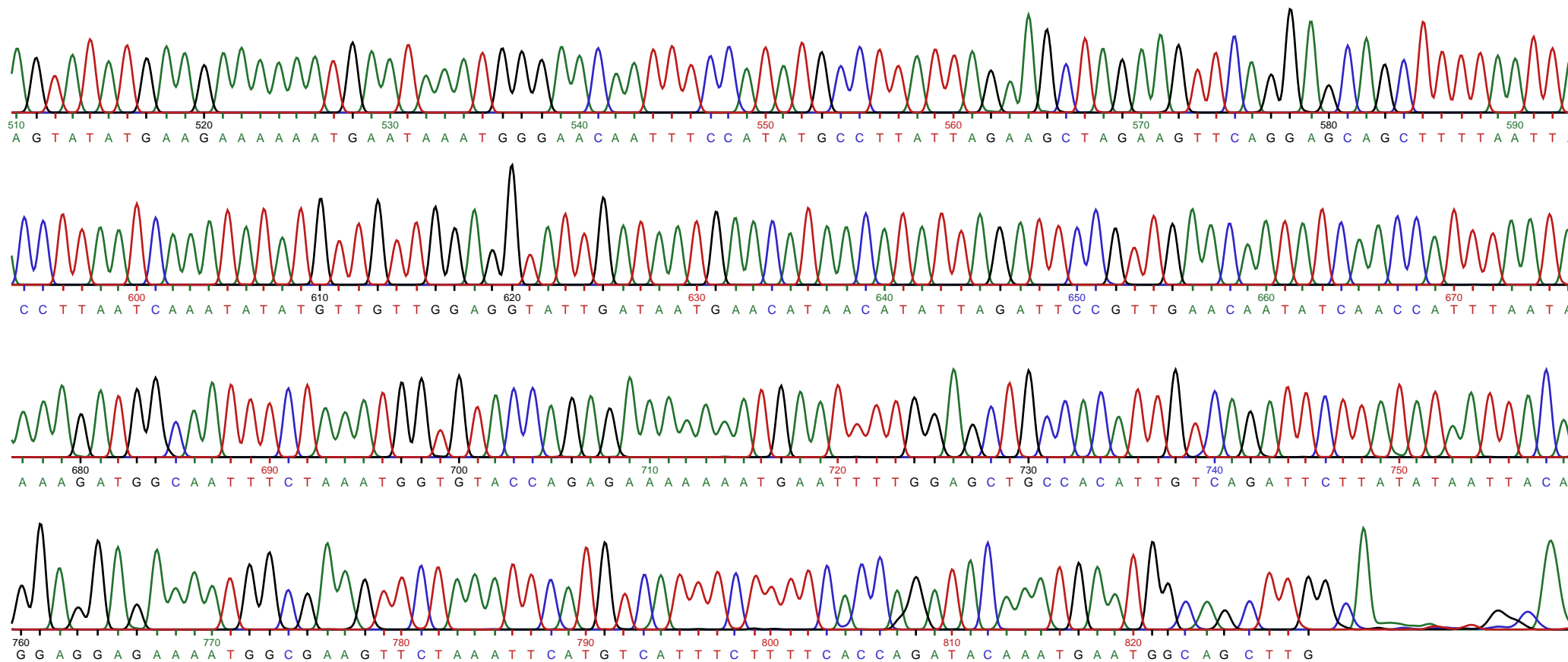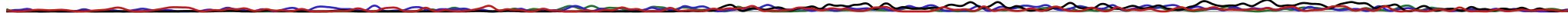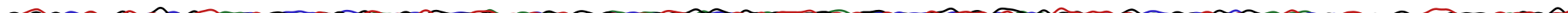

Samples: 12964  
Bases: 830  
Average spacing: 16

Page: 3 / 3  
8/17/2022

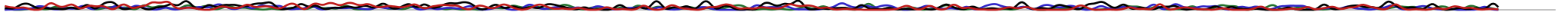

Supplement: Supporting information 2 — (ZIP) [file pone.0316479.s002.zip › 012KNIFW_PREMIX_Plate_CORKELCH_E02.pdf]

Samples: 14020  
Bases: 824  
Average spacing: 18

Page: 1 / 3  
8/17/2022

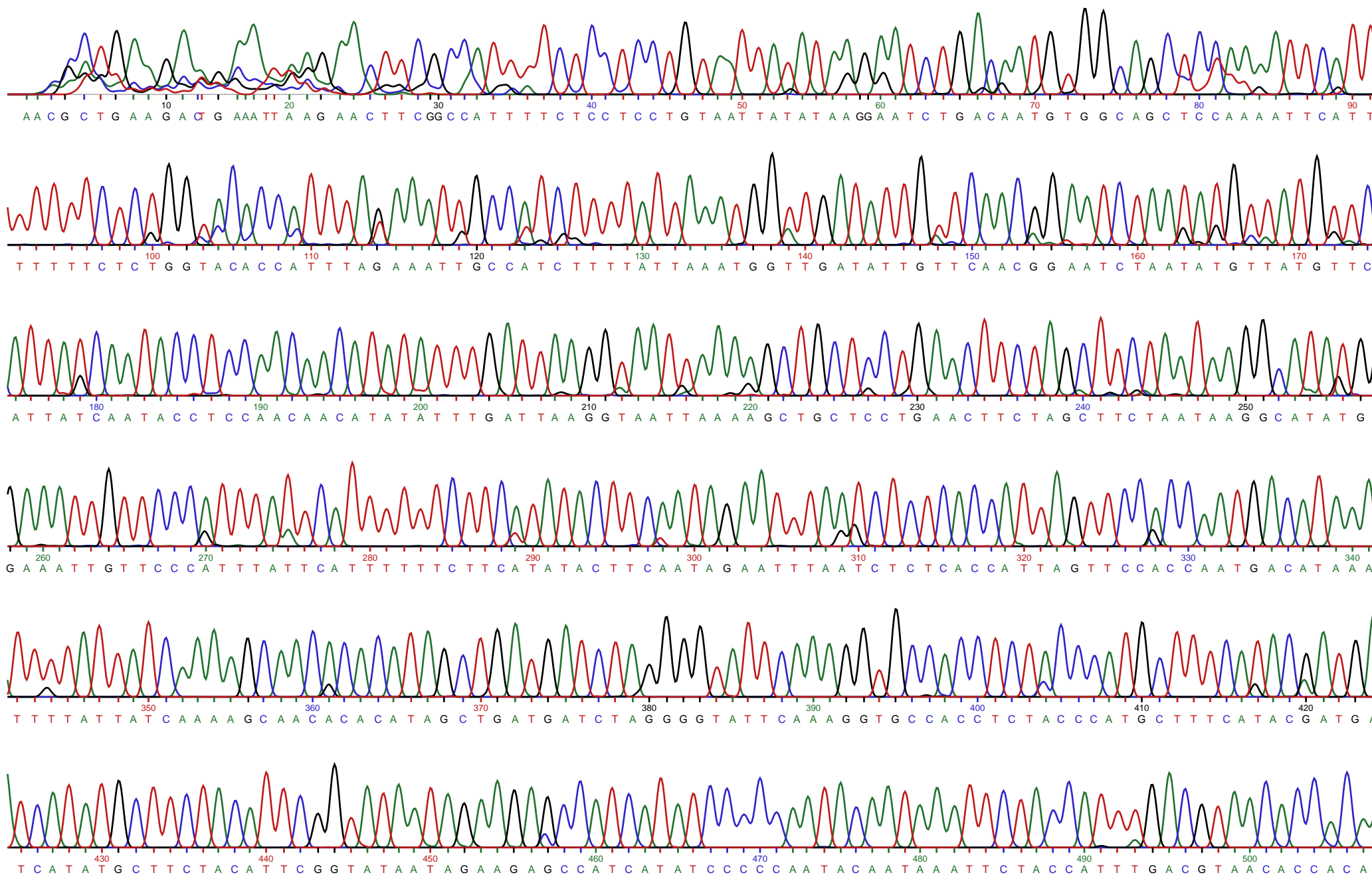

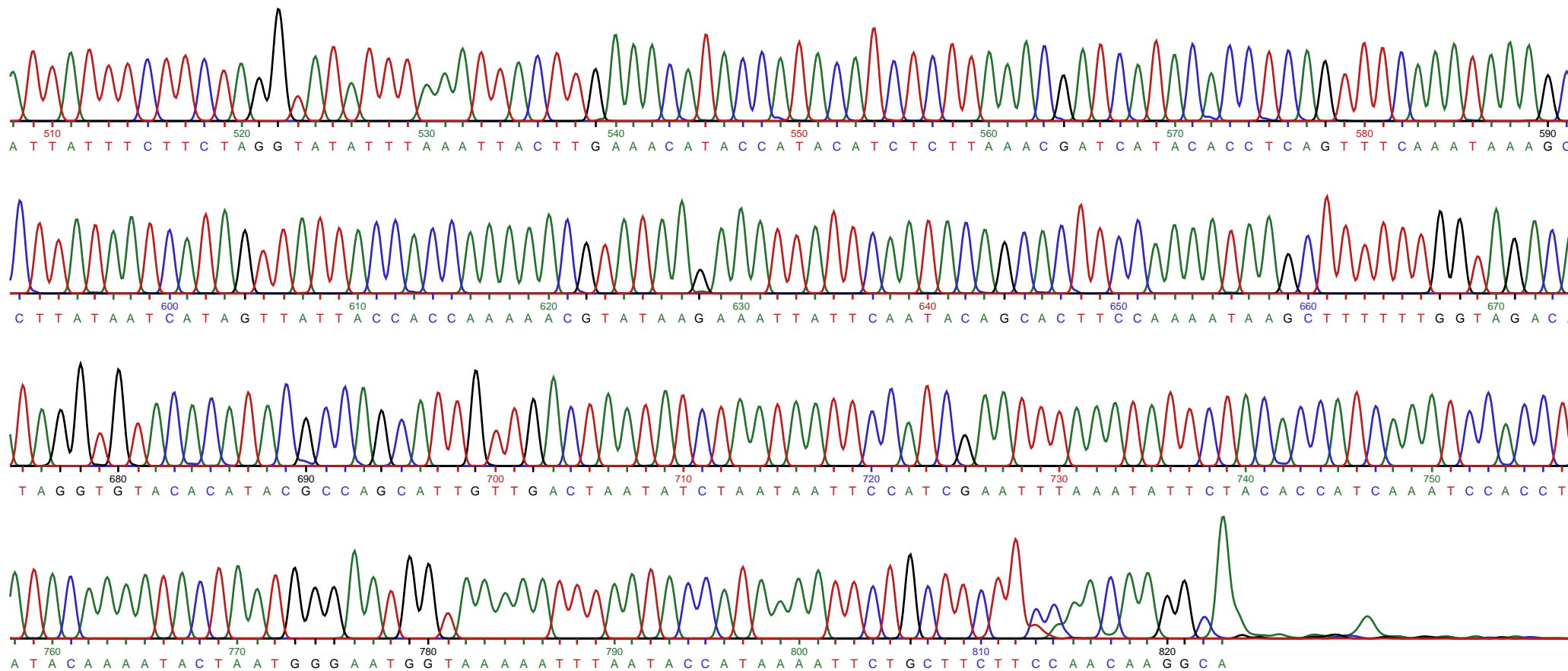

Samples: 14020  
Bases: 824  
Average spacing: 18

Page: 3 / 3  
8/17/2022

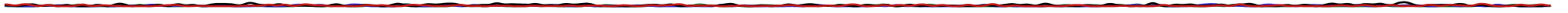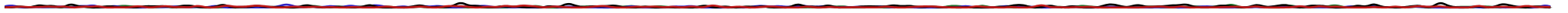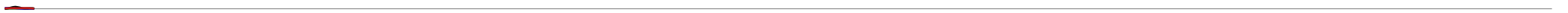

Supplement: Supporting information 2 — (ZIP) [file pone.0316479.s002.zip › 015KN1R_PREMIX_Plate_KELCH1_B05.pdf]

Page: 1 / 3  
8/17/2022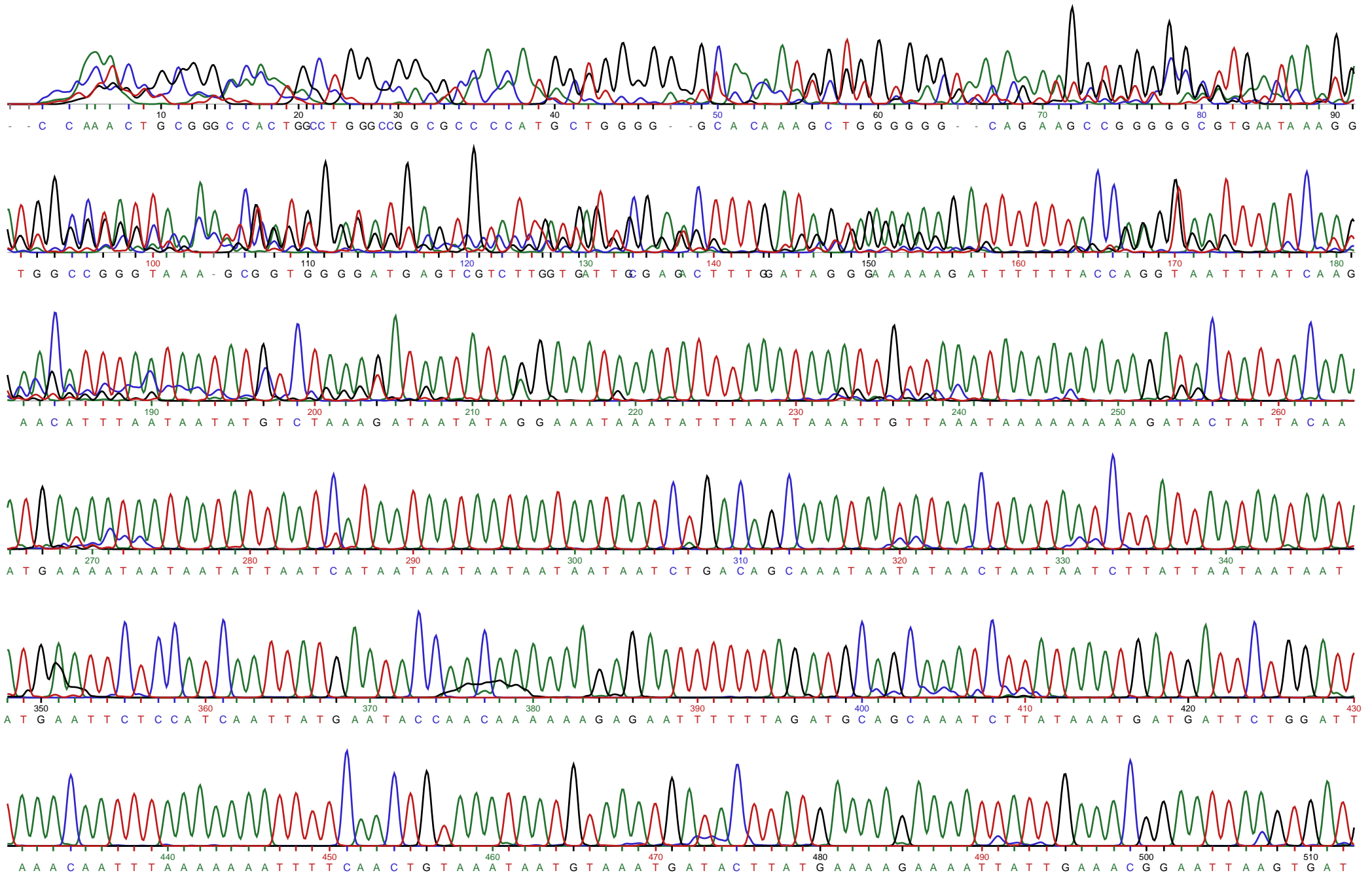

Samples: 13508  
Bases: 1106  
Average spacing: 13

Page: 2 / 3  
8/17/2022

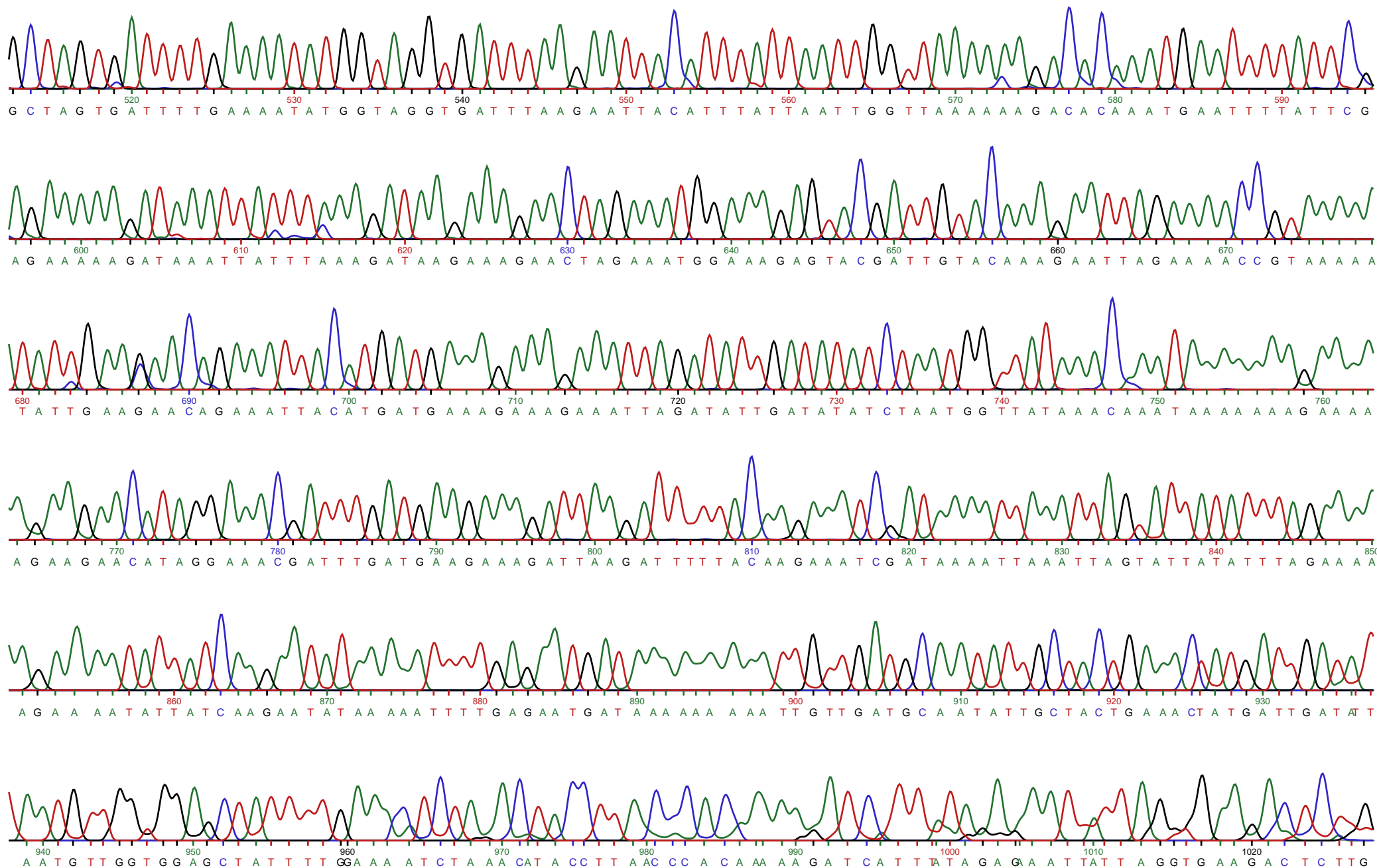

Samples: 13508  
Bases: 1106  
Average spacing: 13

Page: 3 / 3  
8/17/2022

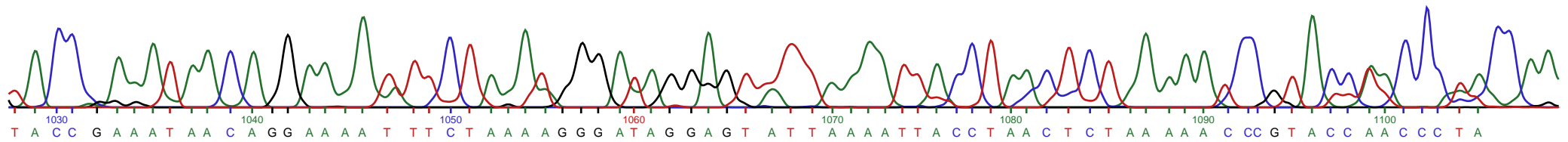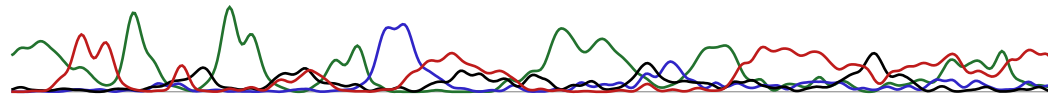

Supplement: Supporting information 2 — (ZIP) [file pone.0316479.s002.zip › 015KN2F_PREMIX_Plate_KELCH1_G05.pdf]

Page: 1 / 3  
8/17/2022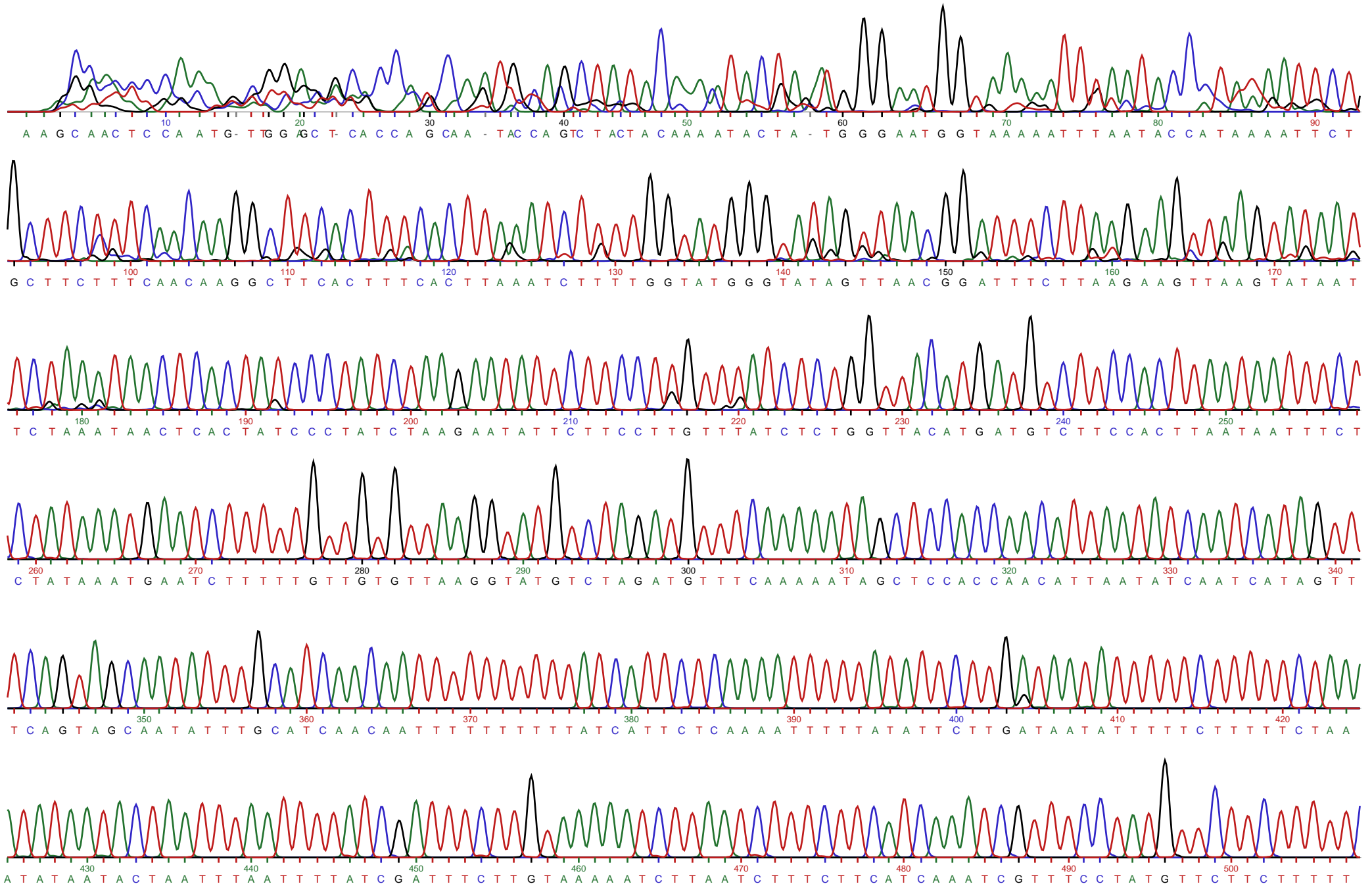

Page: 2 / 3  
8/17/2022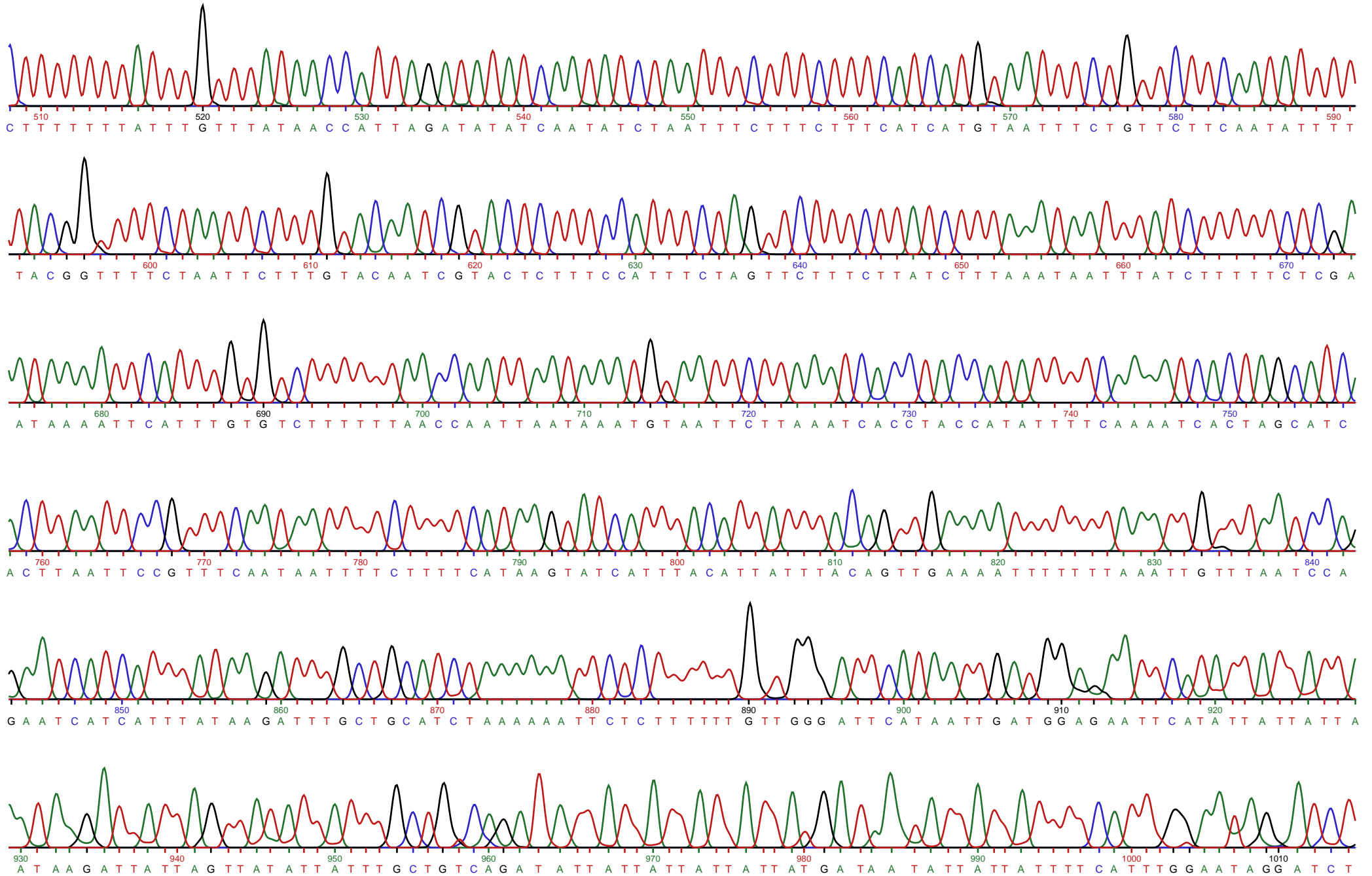

Samples: 13064  
Bases: 1101  
Average spacing: 12

Page: 3 / 3  
8/17/2022

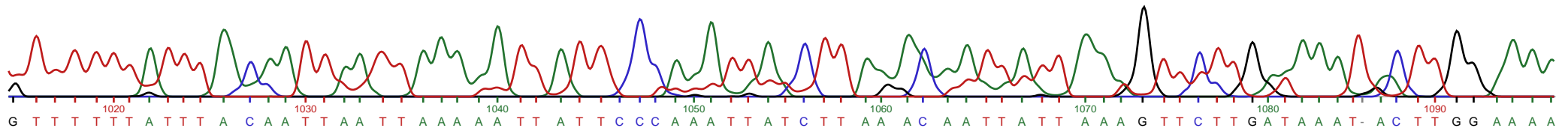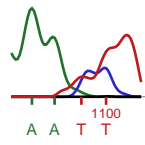

Supplement: Supporting information 2 — (ZIP) [file pone.0316479.s002.zip › 015KN2R_PREMIX_Plate_KELCH2_D07.pdf]

Samples: 13309  
Bases: 824  
Average spacing: 17

Page: 1 / 3  
8/17/2022

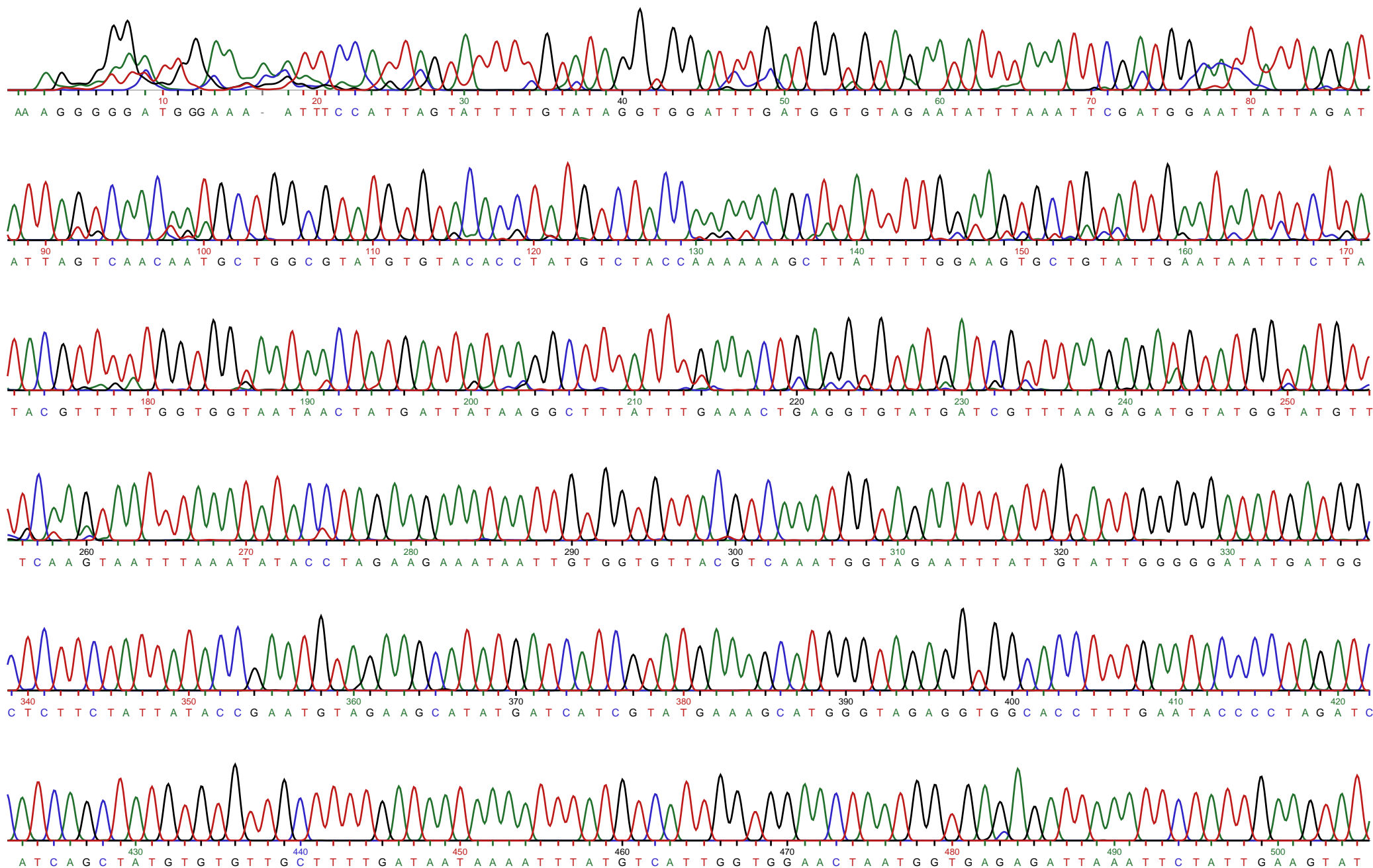

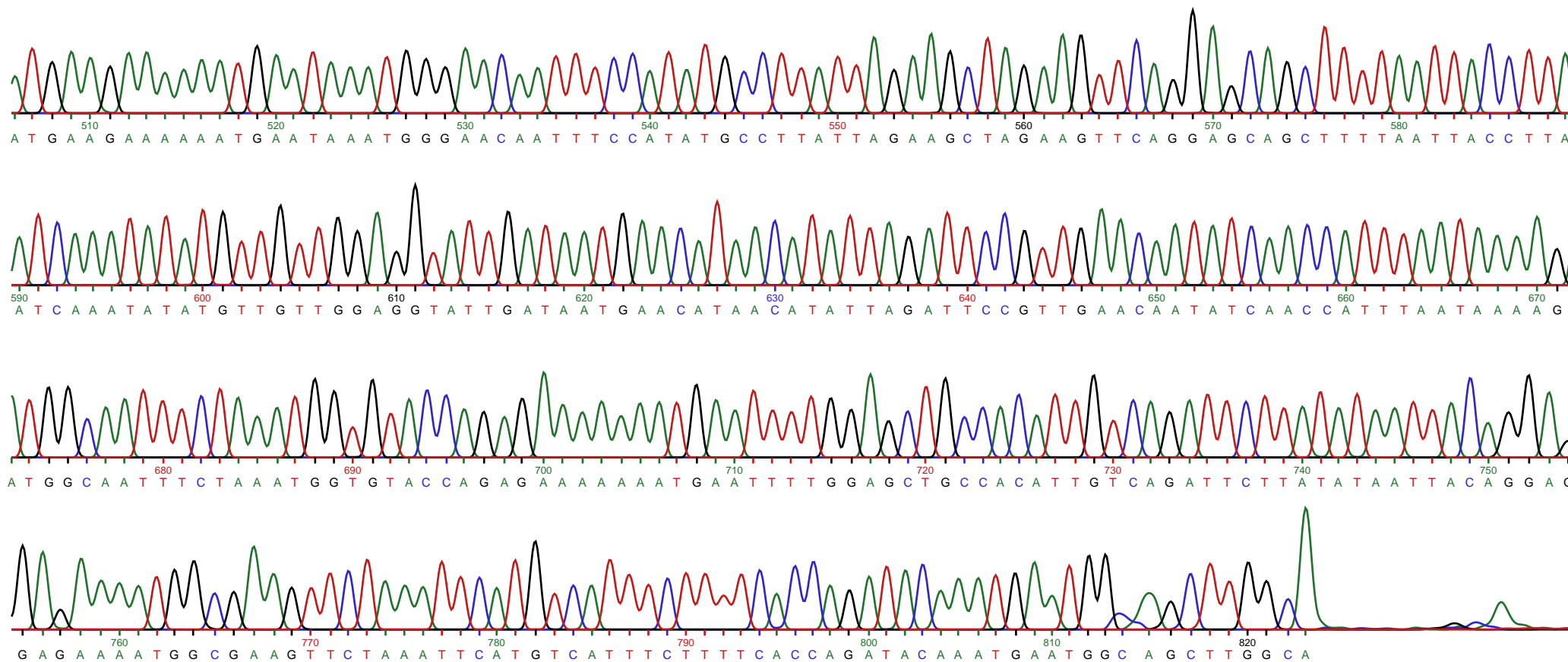

Samples: 13309  
Bases: 824  
Average spacing: 17

Page: 3 / 3  
8/17/2022

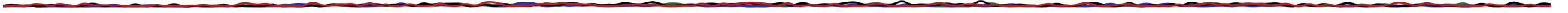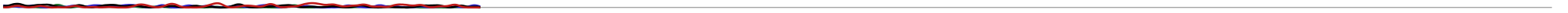

Supplement: Supporting information 2 — (ZIP) [file pone.0316479.s002.zip › 015KNIFW_PREMIX_Plate_CORKELCH_E03.pdf]

Samples: 14164  
Bases: 826  
Average spacing: 18

Page: 1 / 3  
8/17/2022

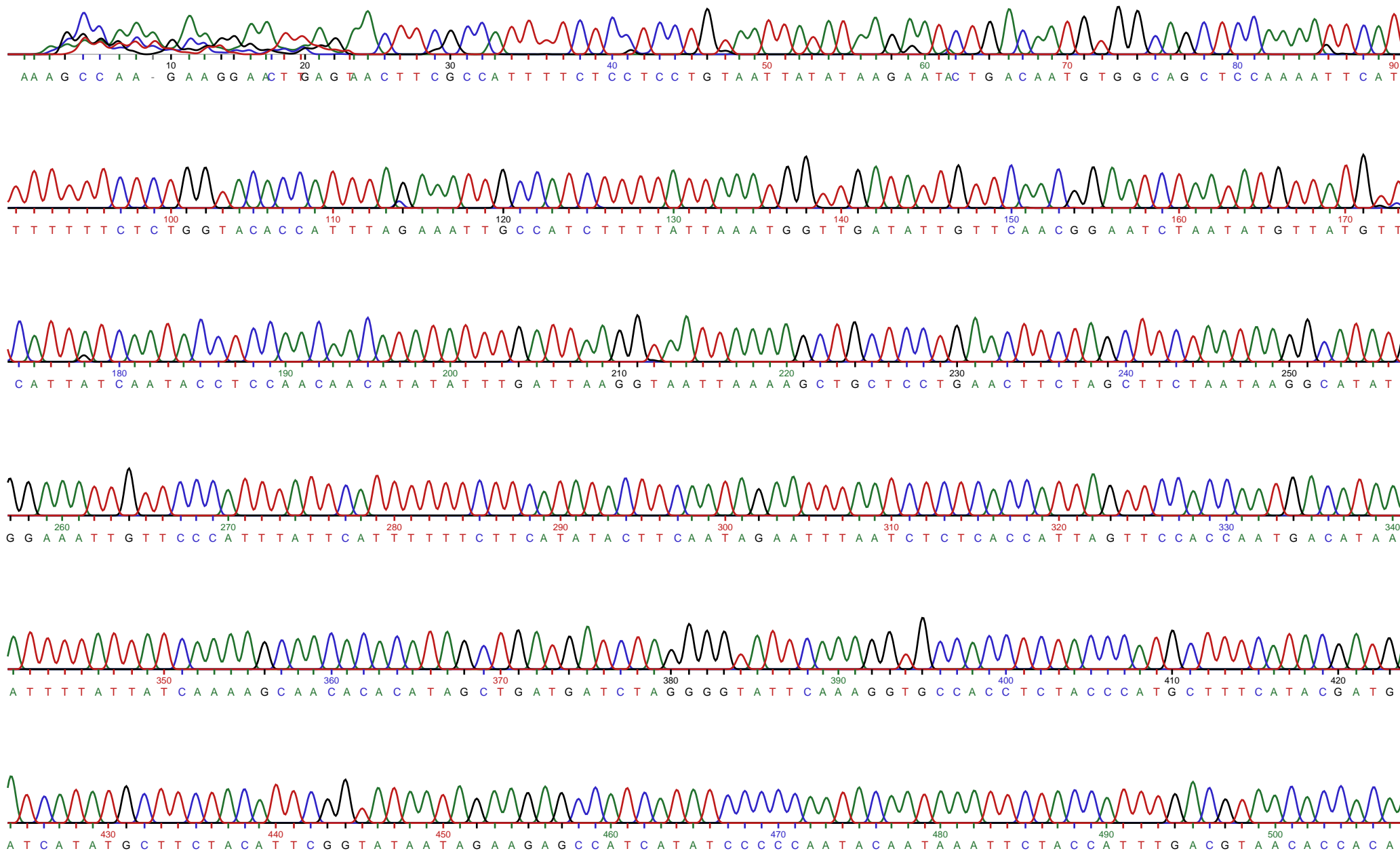

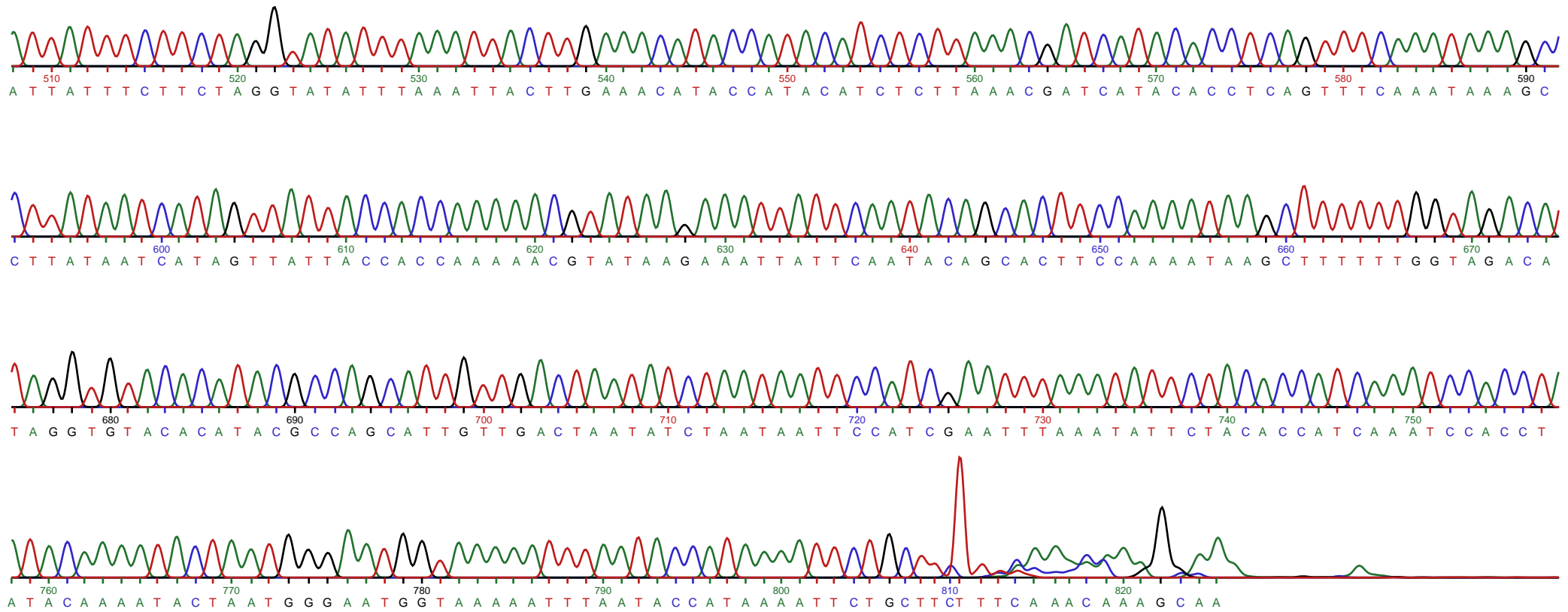

---

---

---

Supplement: Supporting information 2 — (ZIP) [file pone.0316479.s002.zip › 017KN1R_PREMIX_Plate_KELCH1_B06.pdf]

Samples: 13230  
Bases: 1041  
Average spacing: 13

Page: 1 / 3  
8/17/2022

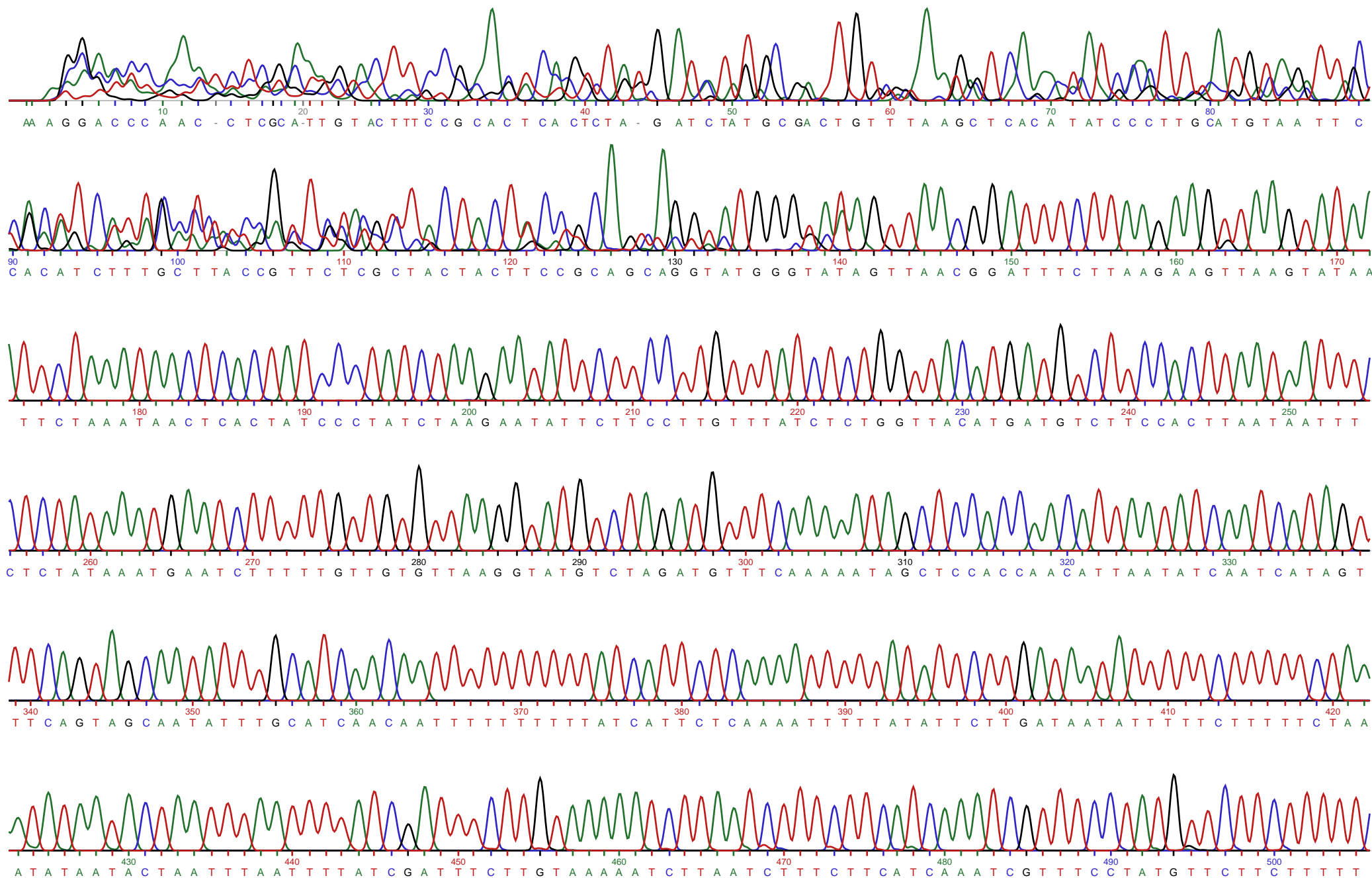

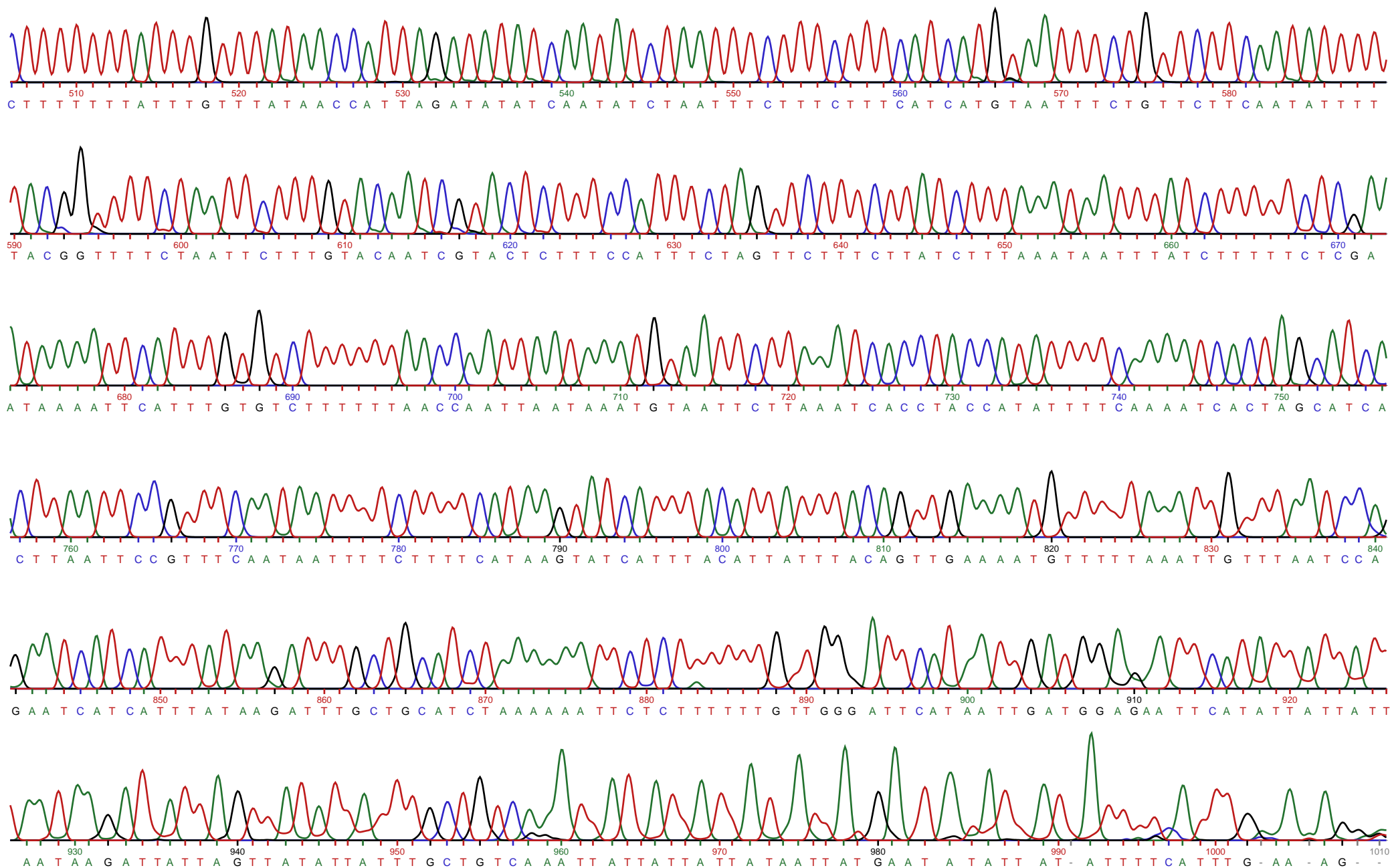

Samples: 13230  
Bases: 1041  
Average spacing: 13

Page: 3 / 3  
8/17/2022

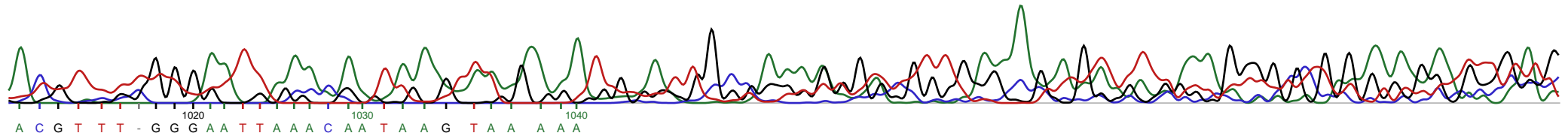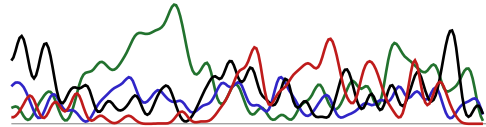

Supplement: Supporting information 2 — (ZIP) [file pone.0316479.s002.zip › 017KN2R_PREMIX_Plate_KELCH2_D08.pdf]

Samples: 14099  
Bases: 829  
Average spacing: 18

Page: 1 / 3  
8/17/2022

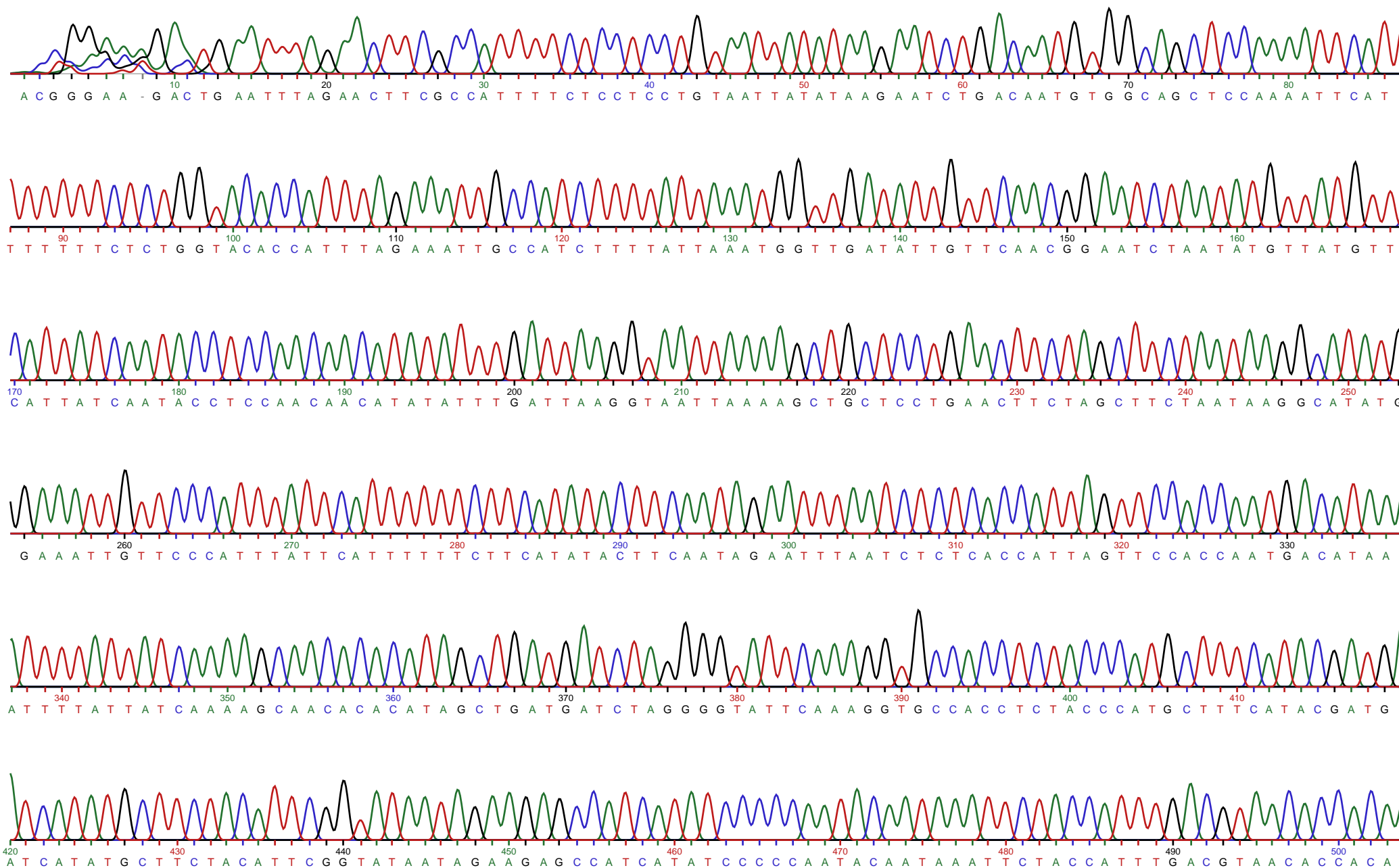

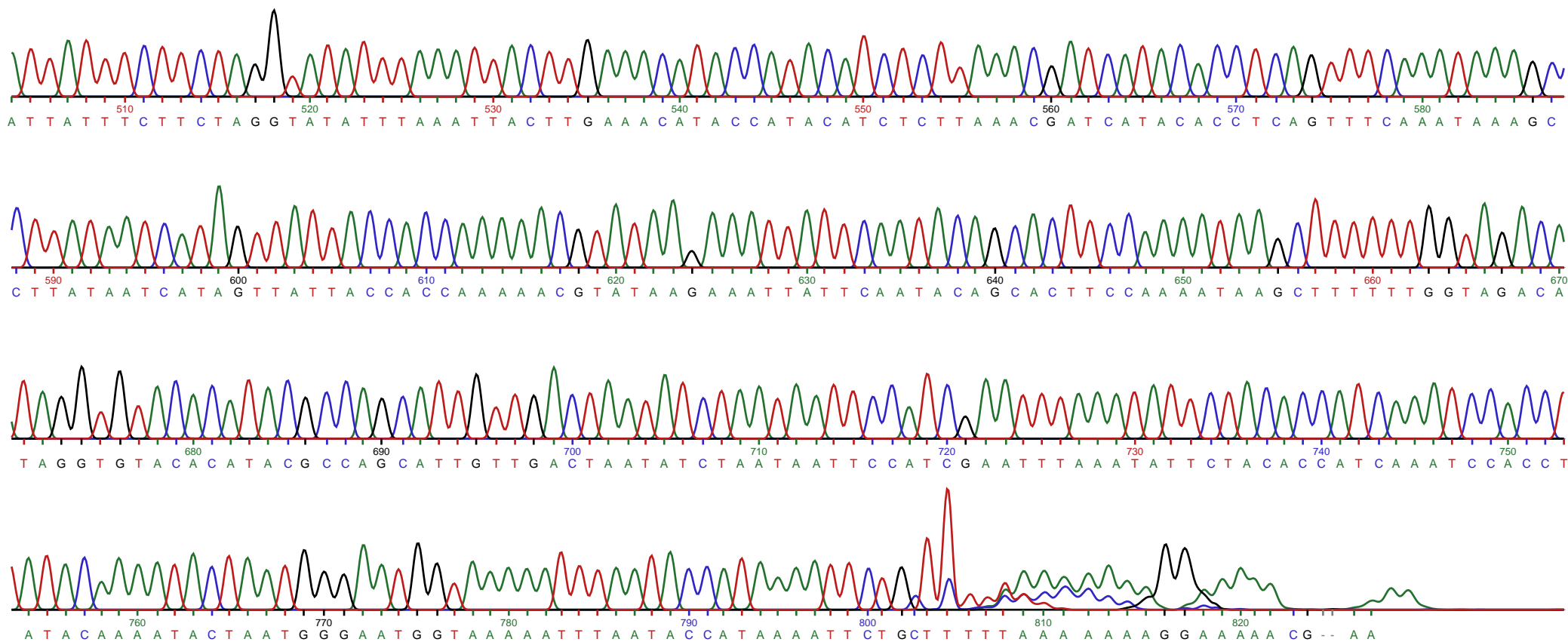

Supplement: Supporting information 2 — (ZIP) [file pone.0316479.s002.zip › 018KN1R_PREMIX_Plate_KELCH1_B07.pdf]

Page: 1 / 3  
8/17/2022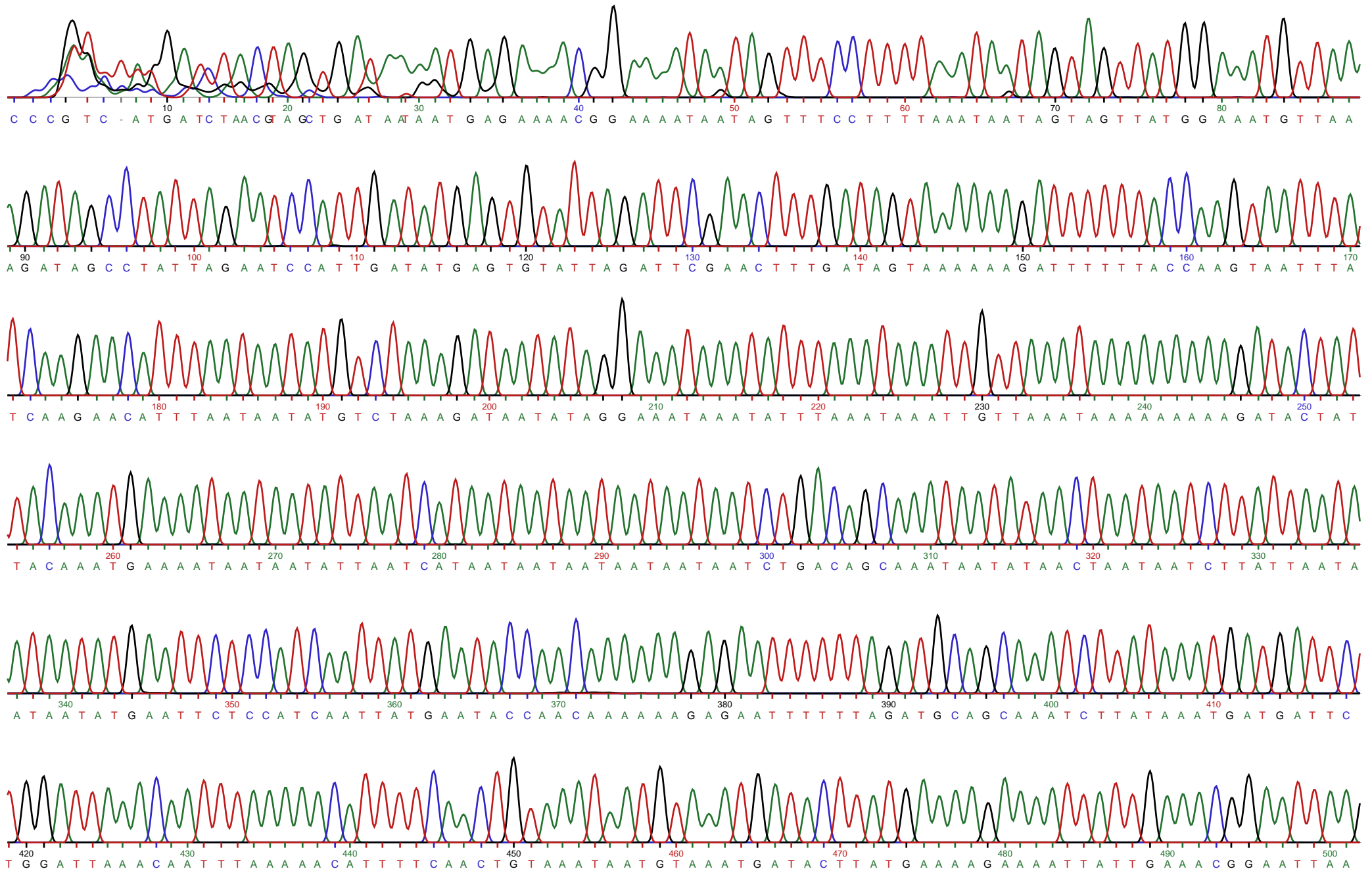

Samples: 13938  
Bases: 1176  
Average spacing: 12

Page: 2 / 3  
8/17/2022

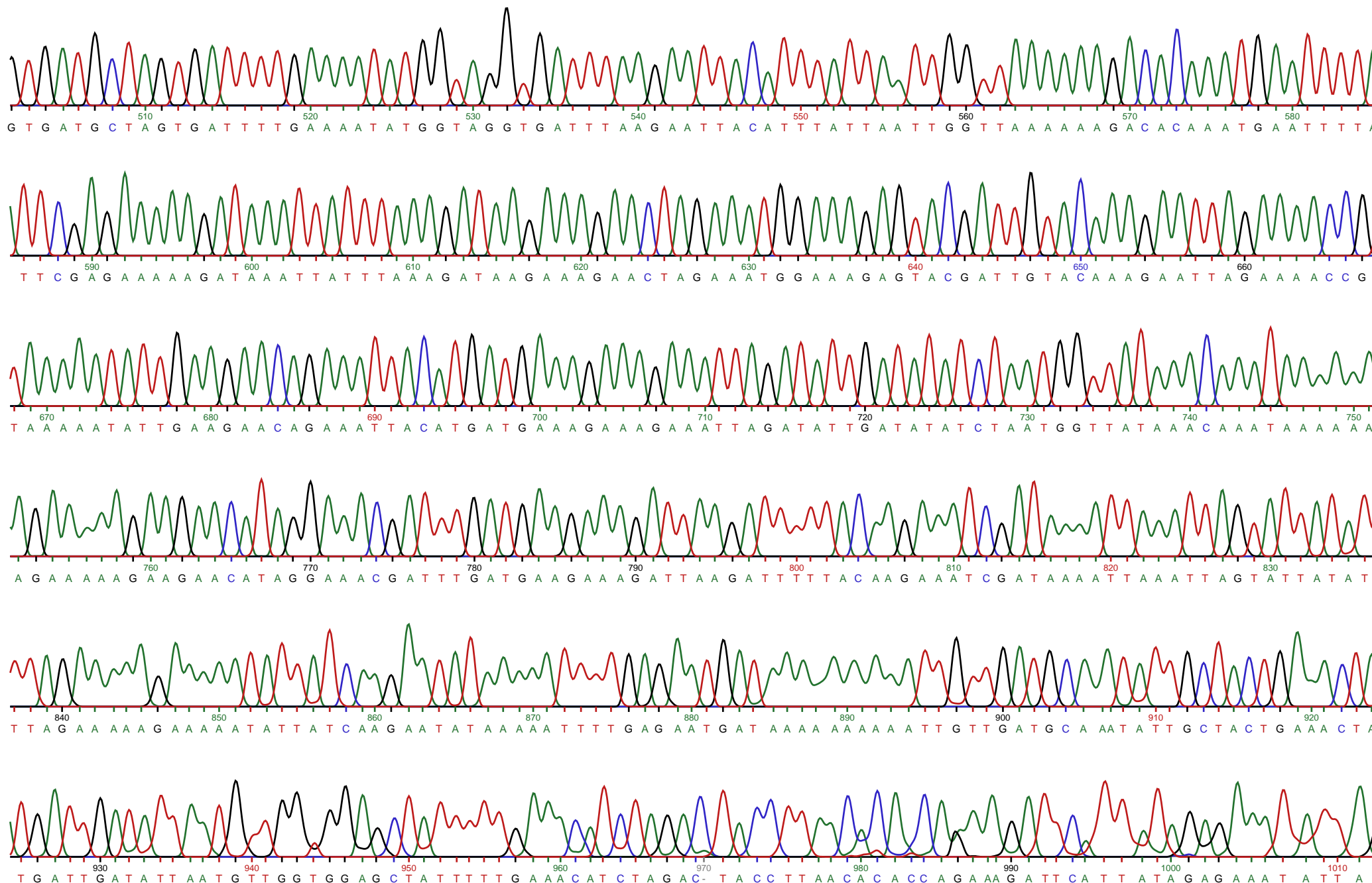

Samples: 13938  
Bases: 1176  
Average spacing: 12

Page: 3 / 3  
8/17/2022

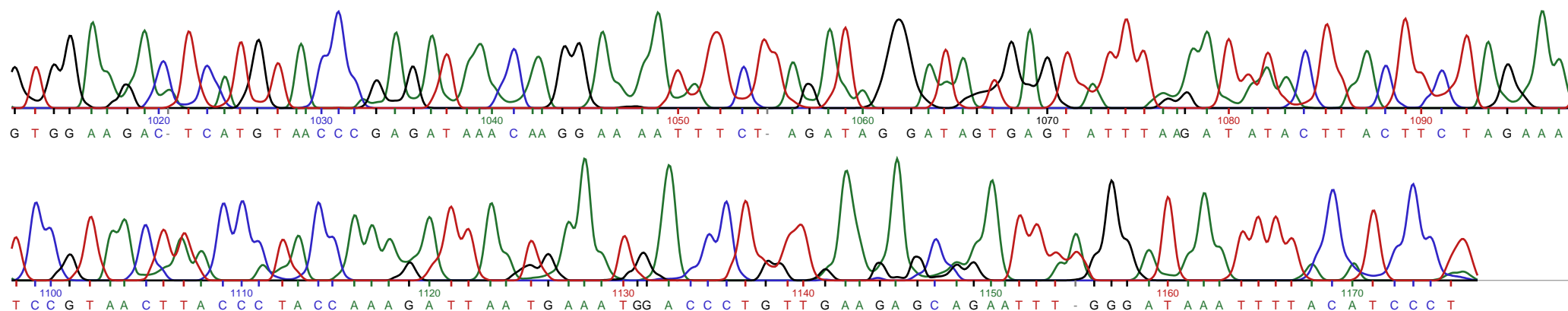

Supplement: Supporting information 2 — (ZIP) [file pone.0316479.s002.zip › 018KN2F_PREMIX_Plate_KELCH1_G07.pdf]

Samples: 14261  
Bases: 823  
Average spacing: 18

Page: 1 / 3  
8/17/2022

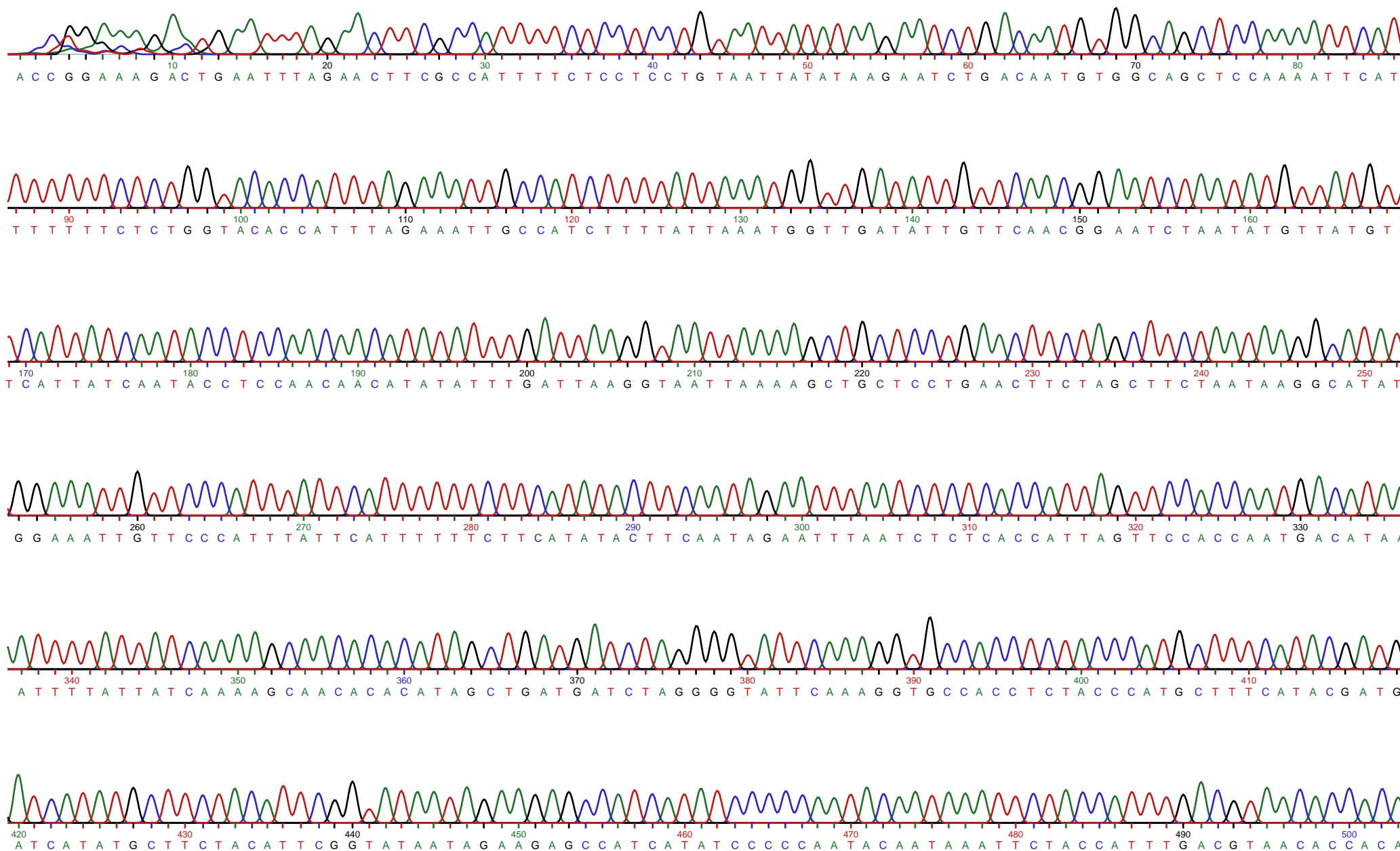

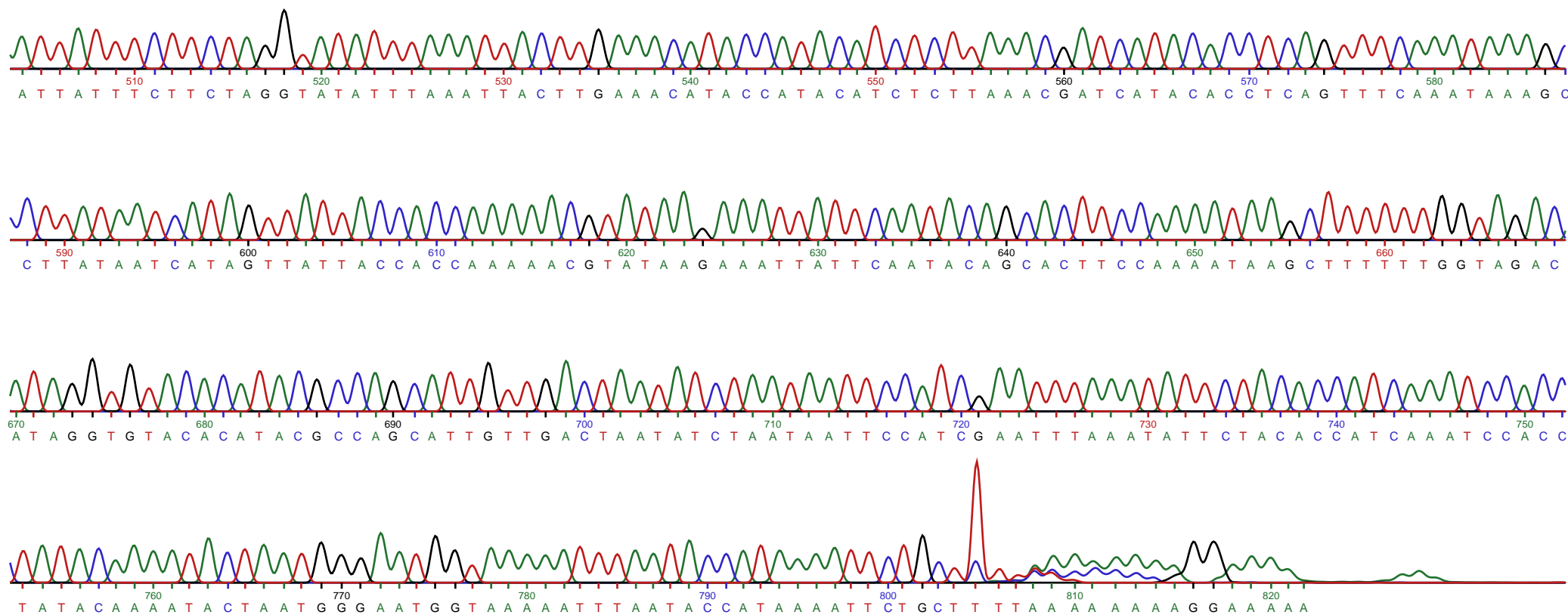

---

---

---

Supplement: Supporting information 2 — (ZIP) [file pone.0316479.s002.zip › 019KN1R_PREMIX_Plate_KELCH1_B08.pdf]

Samples: 12576  
Bases: 1059  
Average spacing: 12

Page: 1 / 3  
8/17/2022

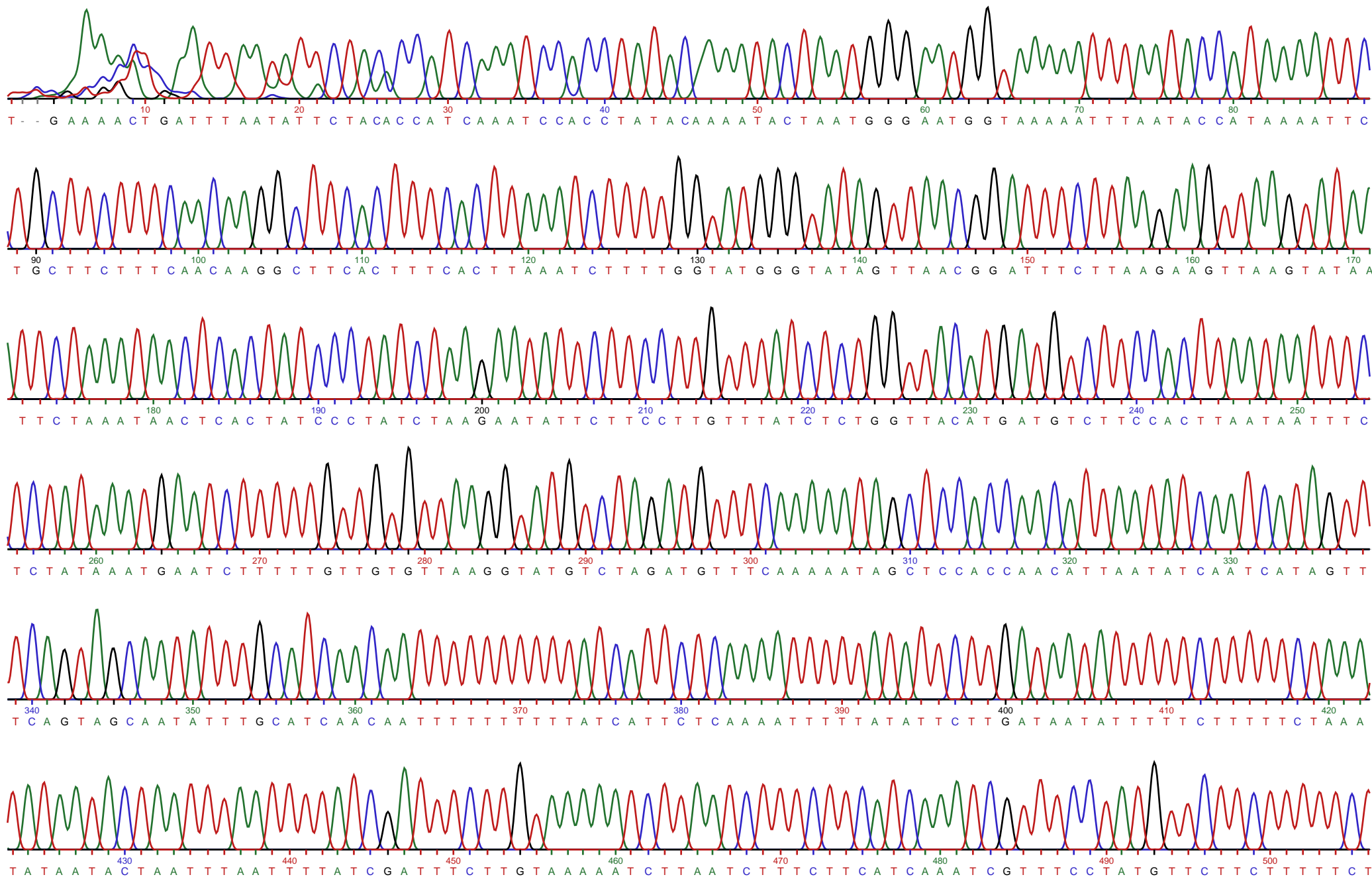

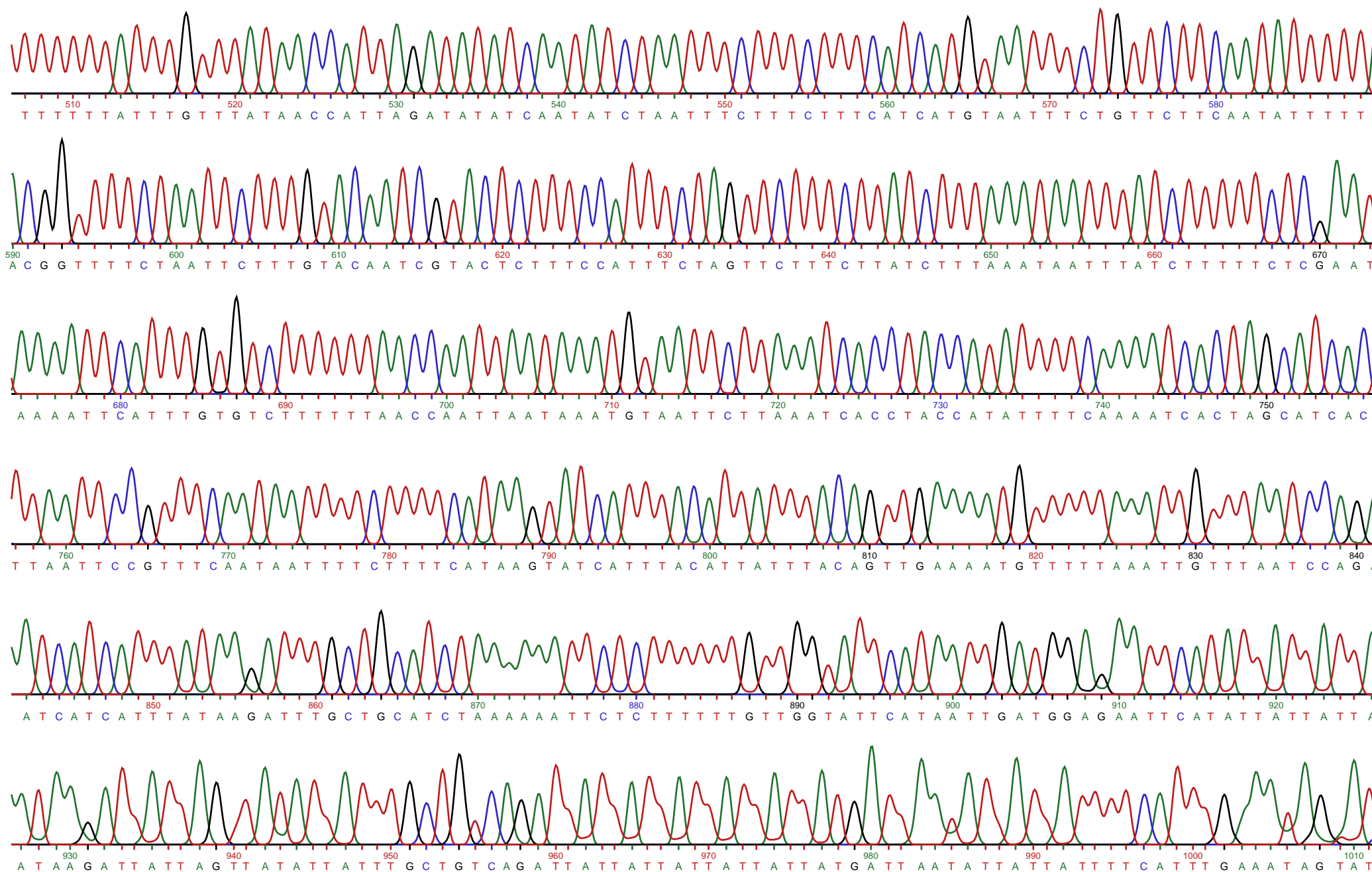

Samples: 12576  
Bases: 1059  
Average spacing: 12

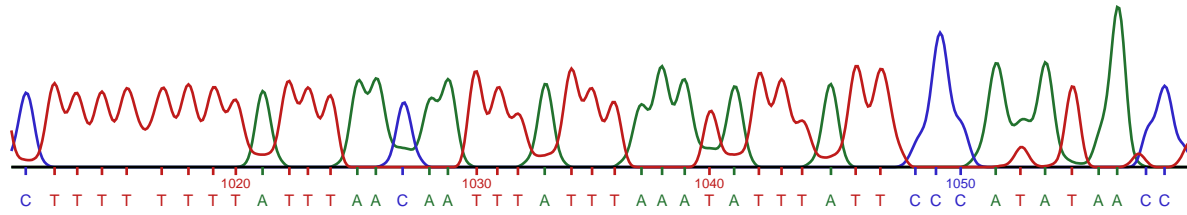

Supplement: Supporting information 2 — (ZIP) [file pone.0316479.s002.zip › 019KN2R_PREMIX_Plate_KELCH2_D10.pdf]

Samples: 13624  
Bases: 822  
Average spacing: 17

Page: 1 / 3  
8/17/2022

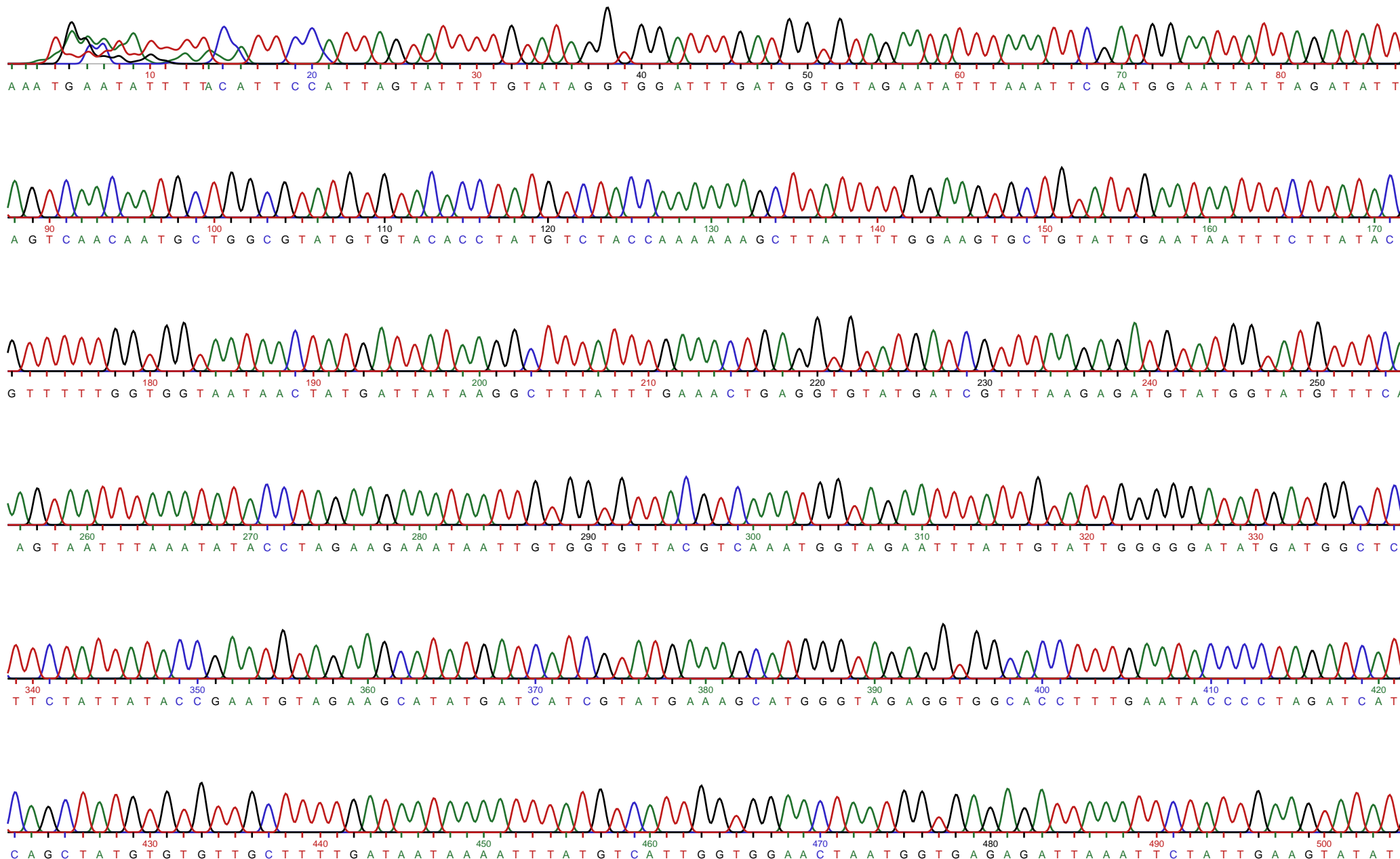

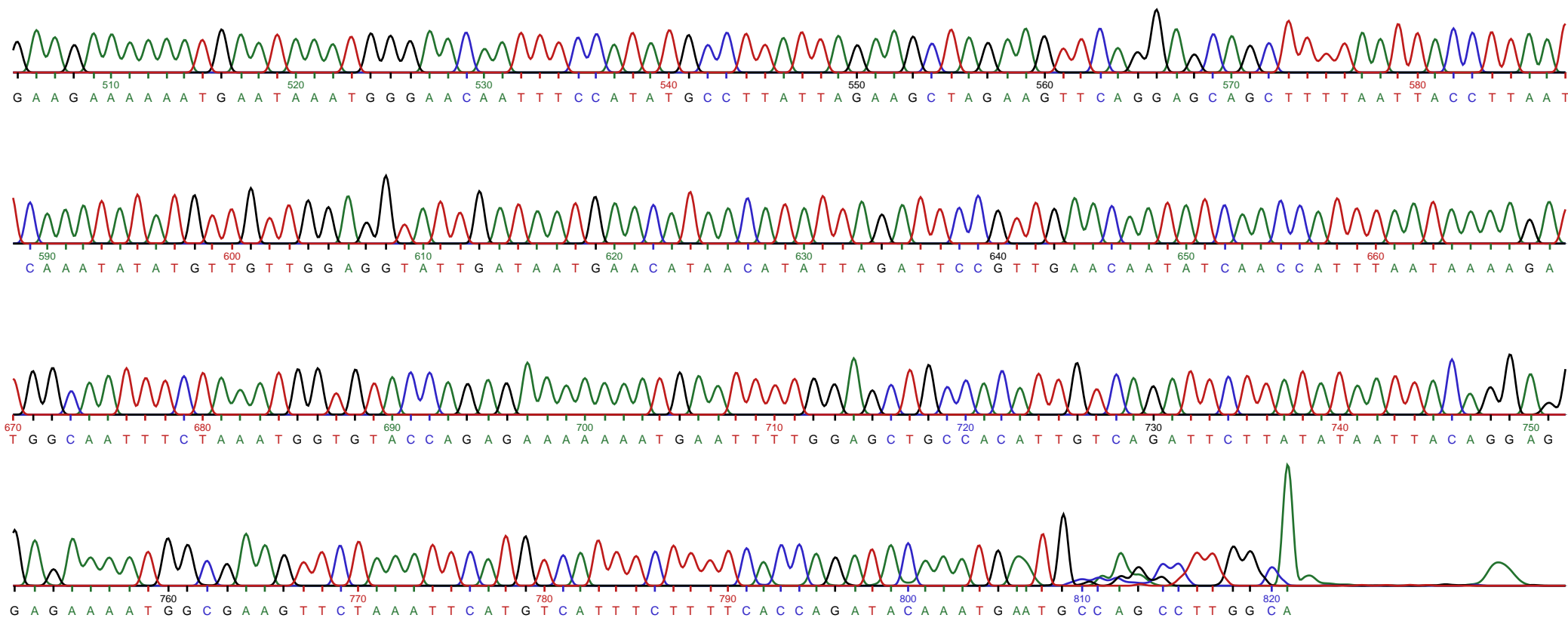

Supplement: Supporting information 2 — (ZIP) [file pone.0316479.s002.zip › 019KNIFW_PREMIX_Plate_CORKELCH_E06.pdf]

Samples: 13845  
Bases: 827  
Average spacing: 17

Page: 1 / 3  
8/17/2022

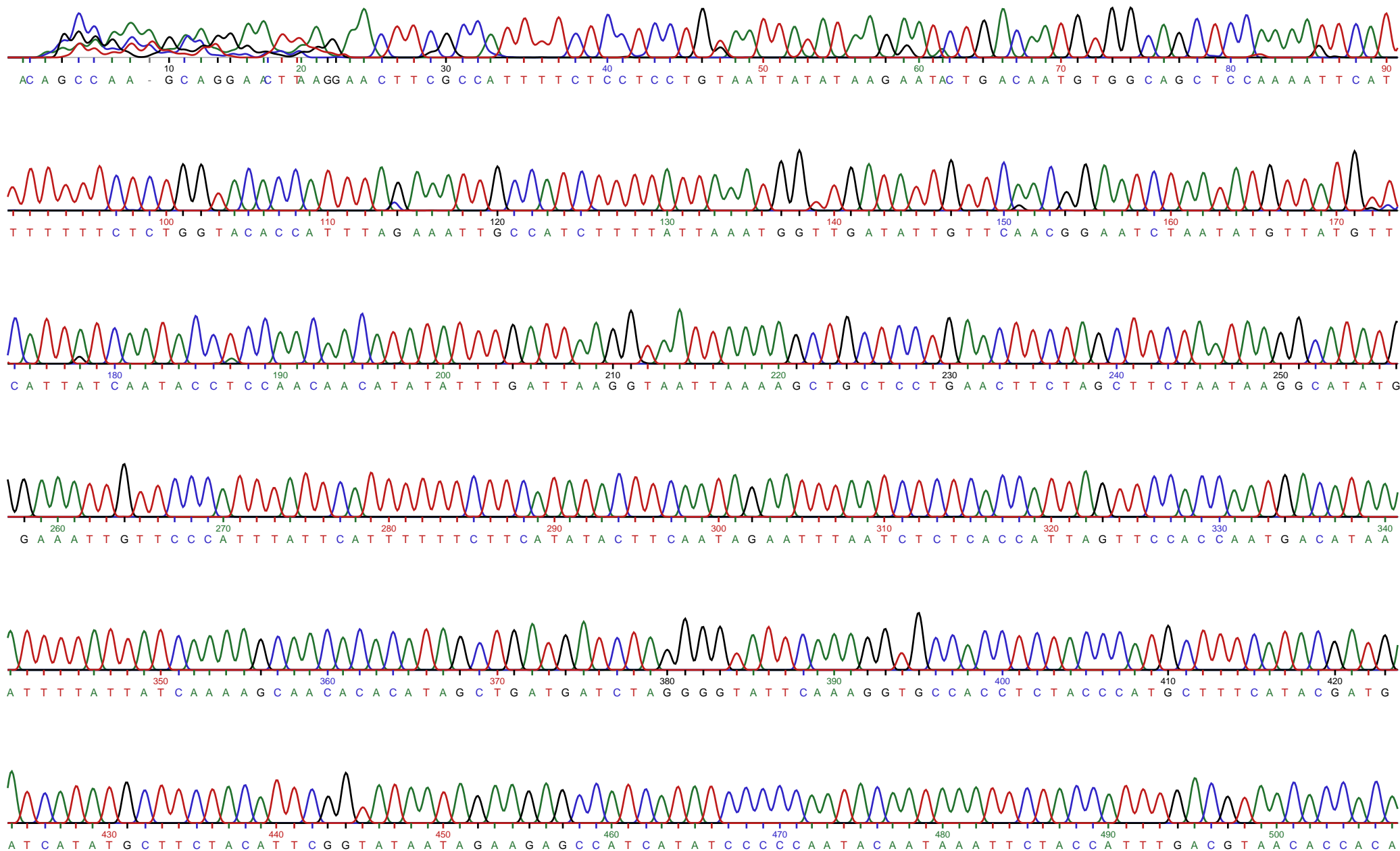

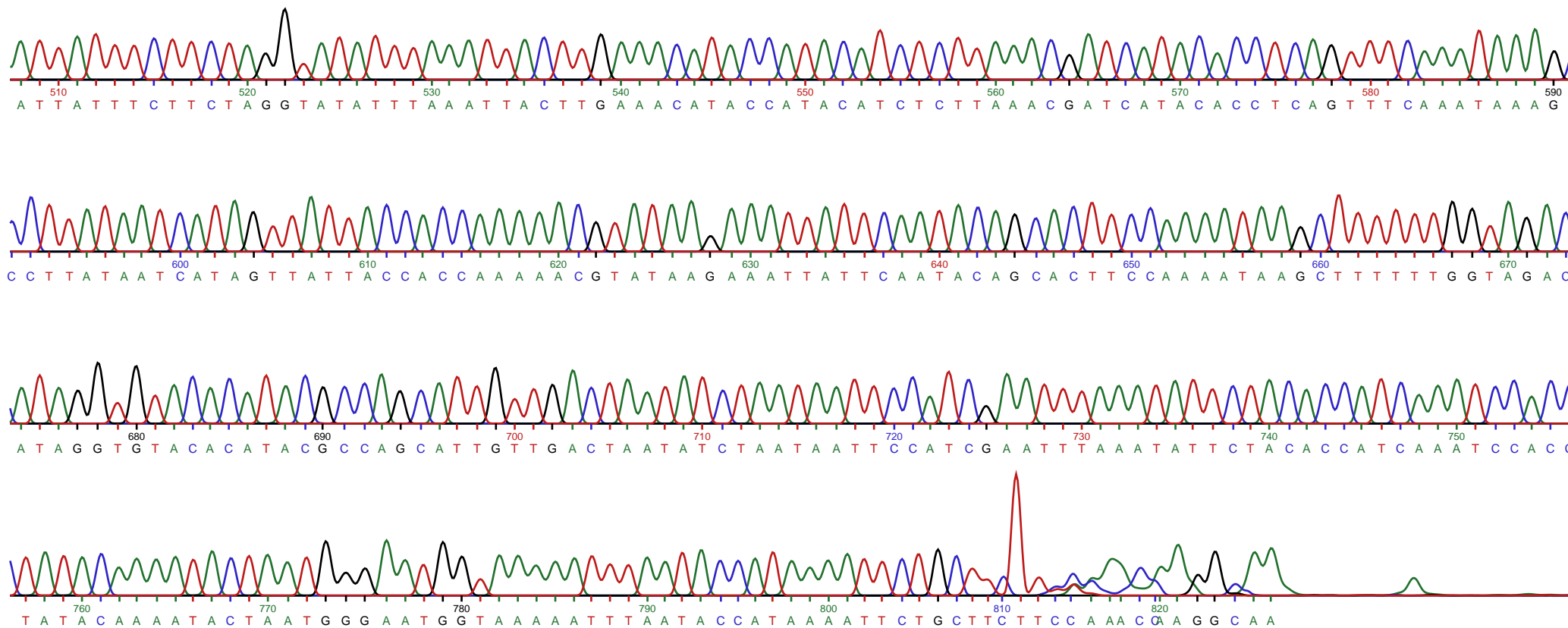

---

---

Supplement: Supporting information 2 — (ZIP) [file pone.0316479.s002.zip › 022KN1R_PREMIX_Plate_KELCH1_B09.pdf]

Samples: 11915  
Bases: 1014  
Average spacing: 12

Page: 1 / 2  
8/17/2022

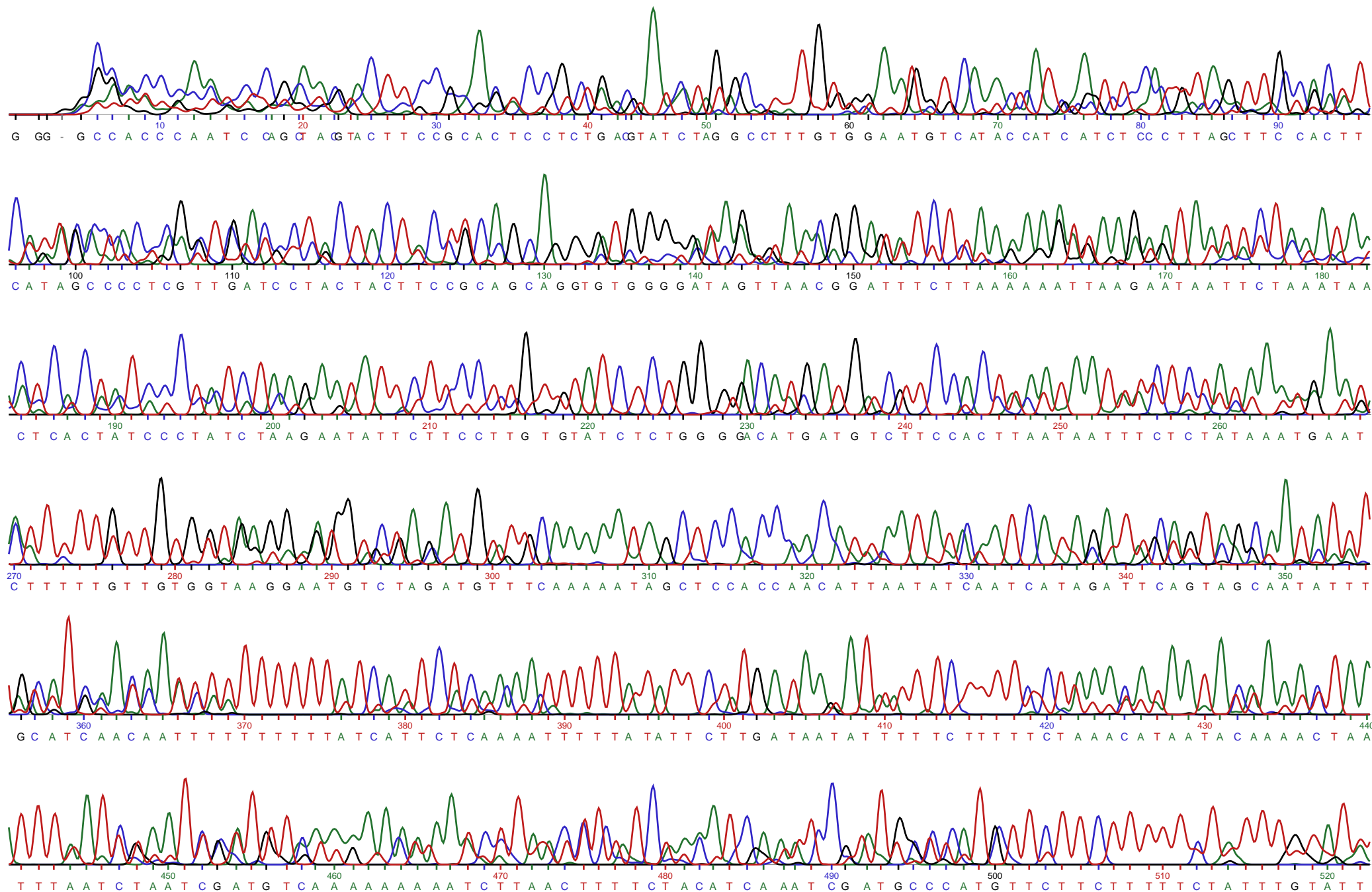

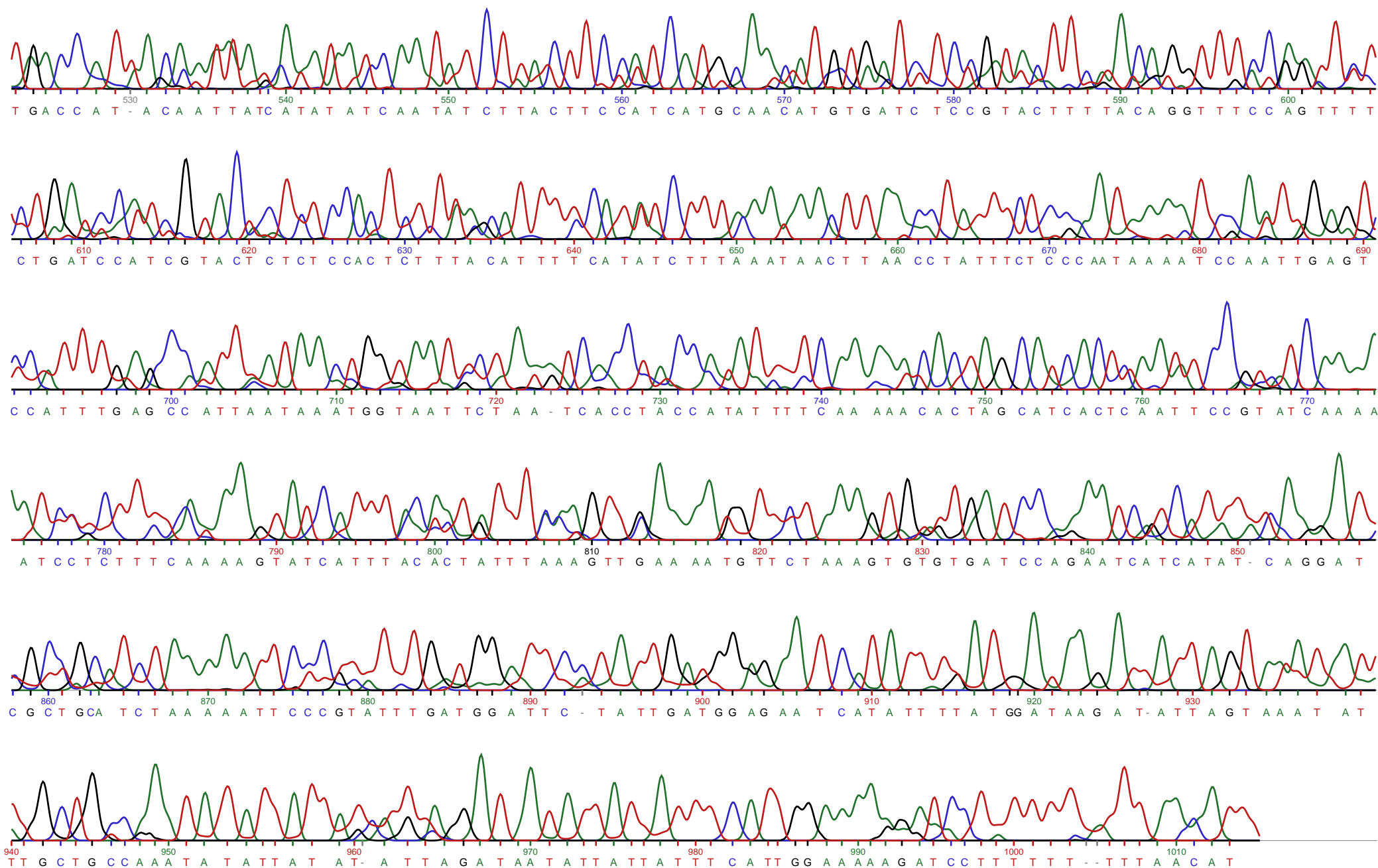

Supplement: Supporting information 2 — (ZIP) [file pone.0316479.s002.zip › 022KN2R_PREMIX_Plate_KELCH2_D11.pdf]

Page: 1 / 3  
8/17/2022

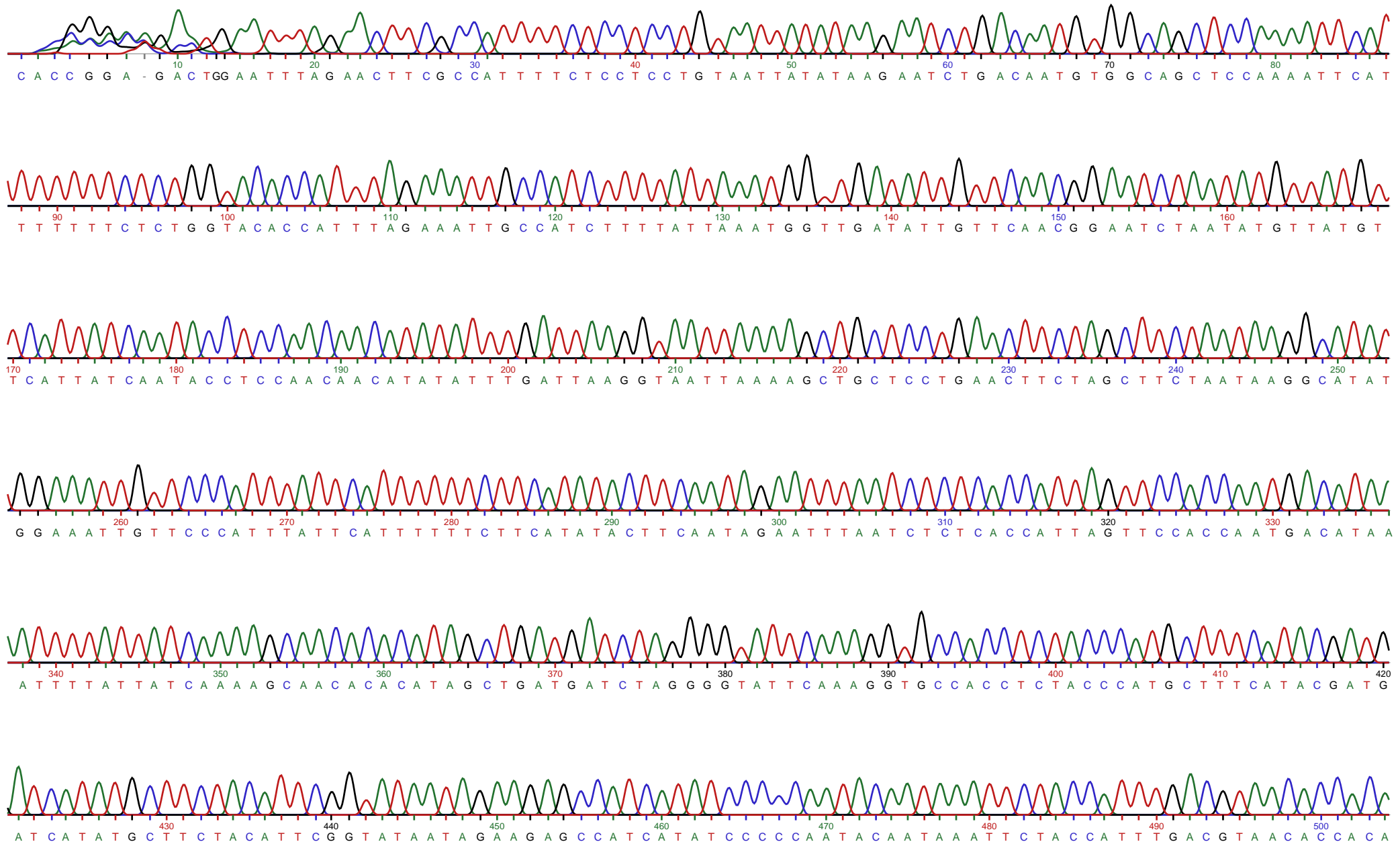

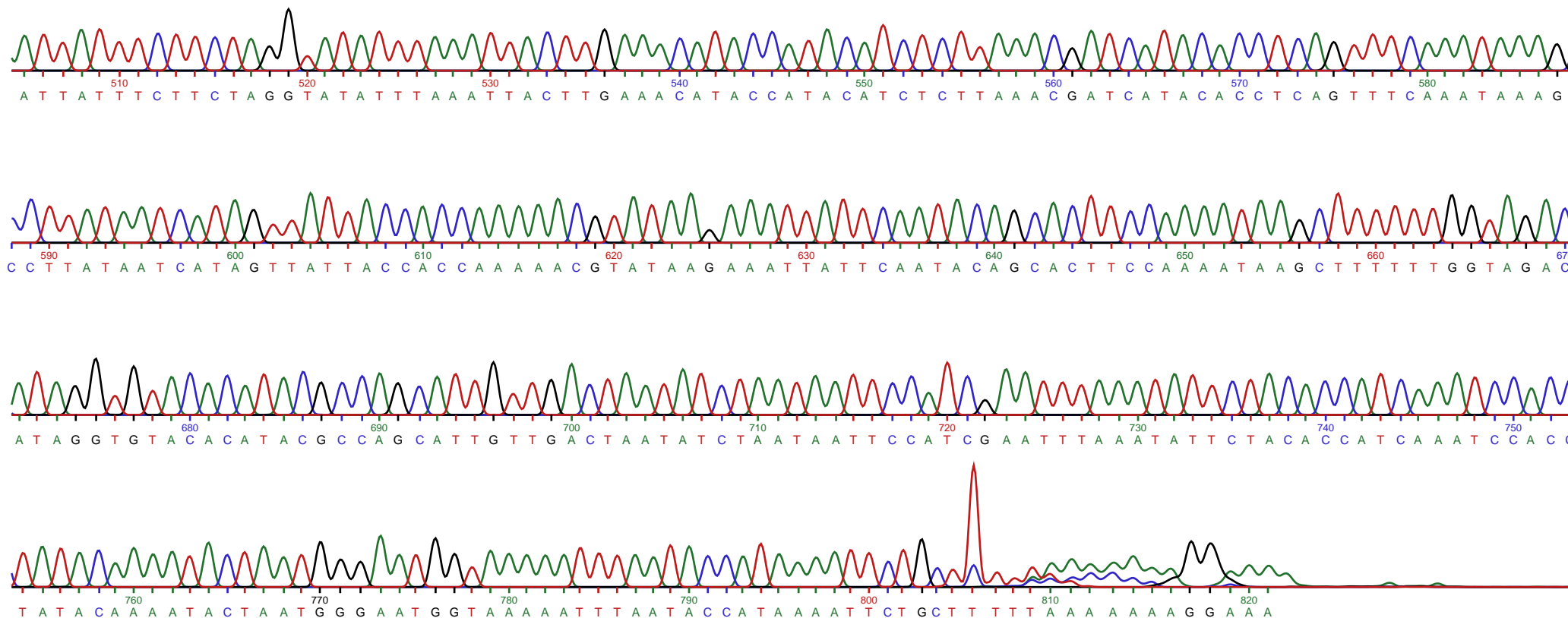

---

---

Supplement: Supporting information 2 — (ZIP) [file pone.0316479.s002.zip › 024KN1R_PREMIX_Plate_KELCH1_B10.pdf]

Page: 1 / 3  
8/17/2022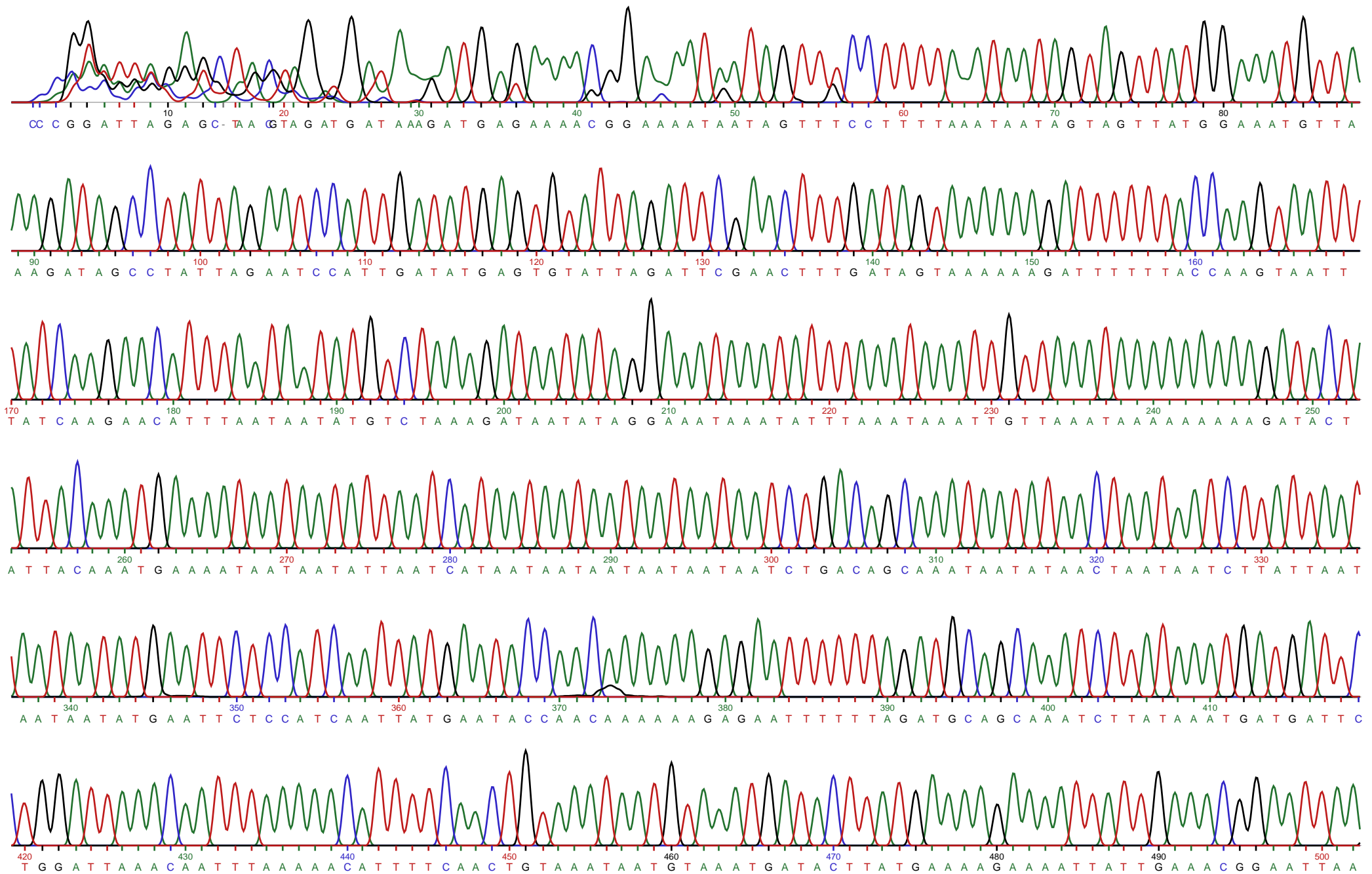

Samples: 13546  
Bases: 1142  
Average spacing: 12

Page: 2 / 3  
8/17/2022

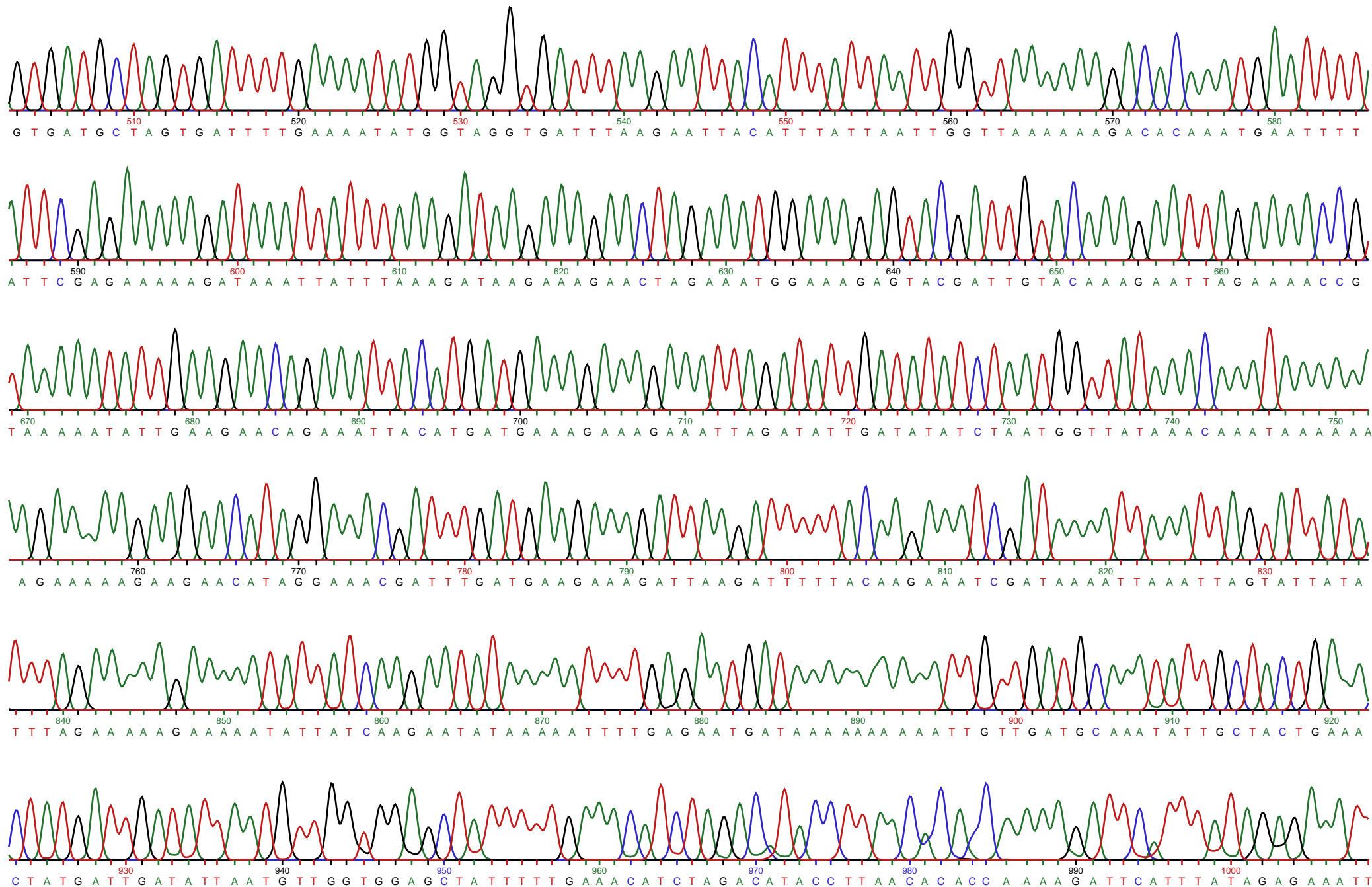

Samples: 13546  
Bases: 1142  
Average spacing: 12

Page: 3 / 3  
8/17/2022

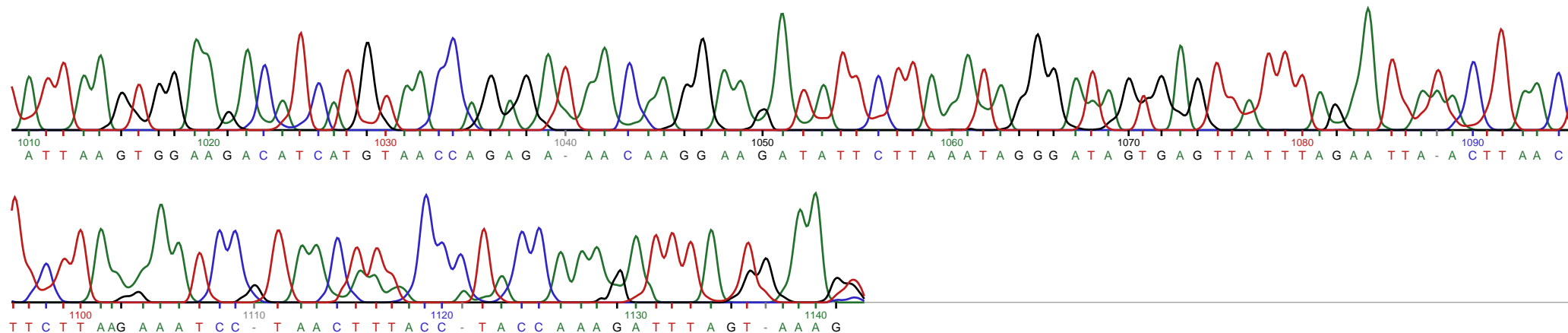

Supplement: Supporting information 2 — (ZIP) [file pone.0316479.s002.zip › 024KN2F_PREMIX_Plate_KELCH1_G10.pdf]

Samples: 12018  
Bases: 1010  
Average spacing: 12

Page: 1 / 3  
8/17/2022

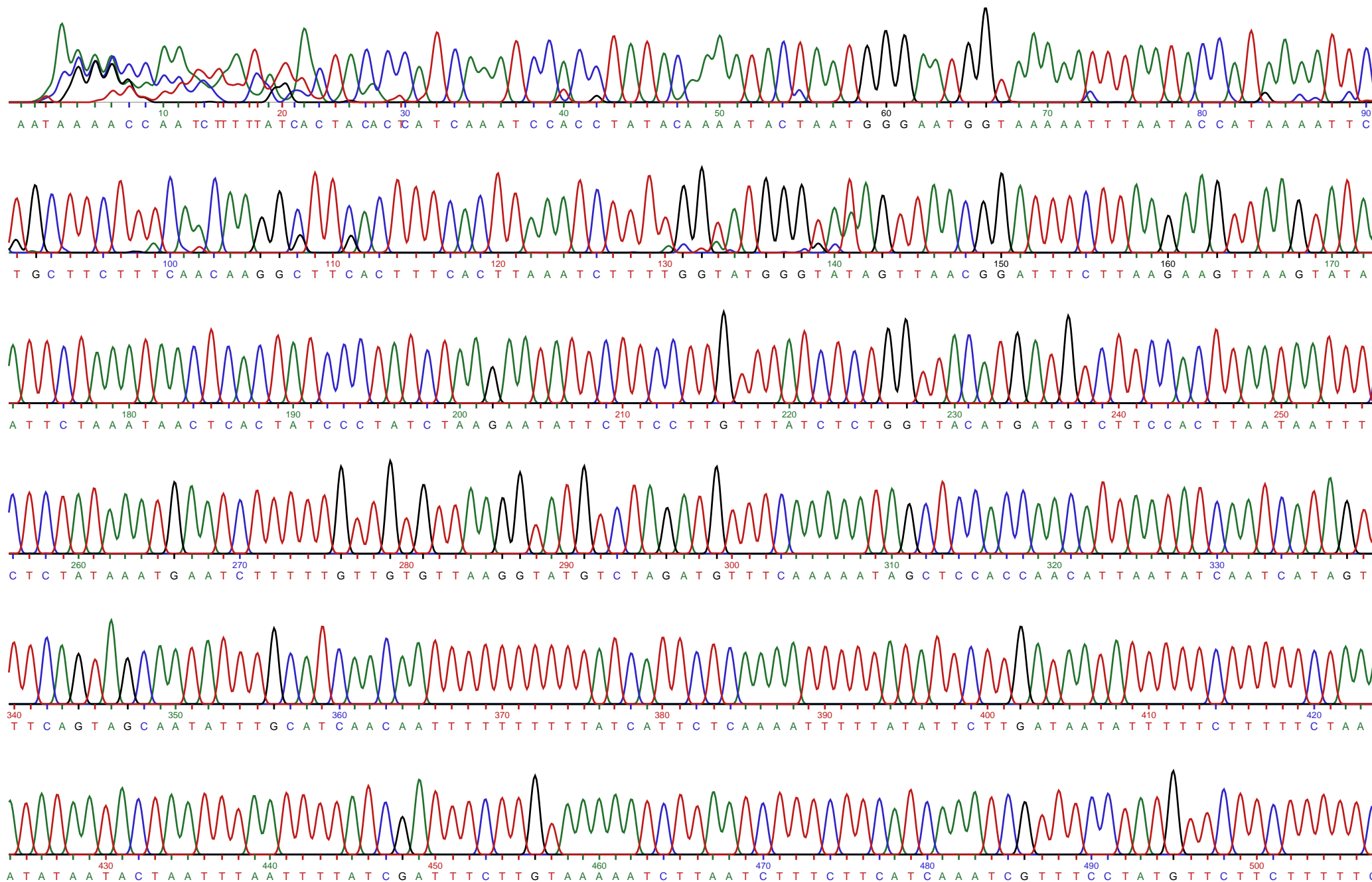

Page: 2 / 3  
8/17/2022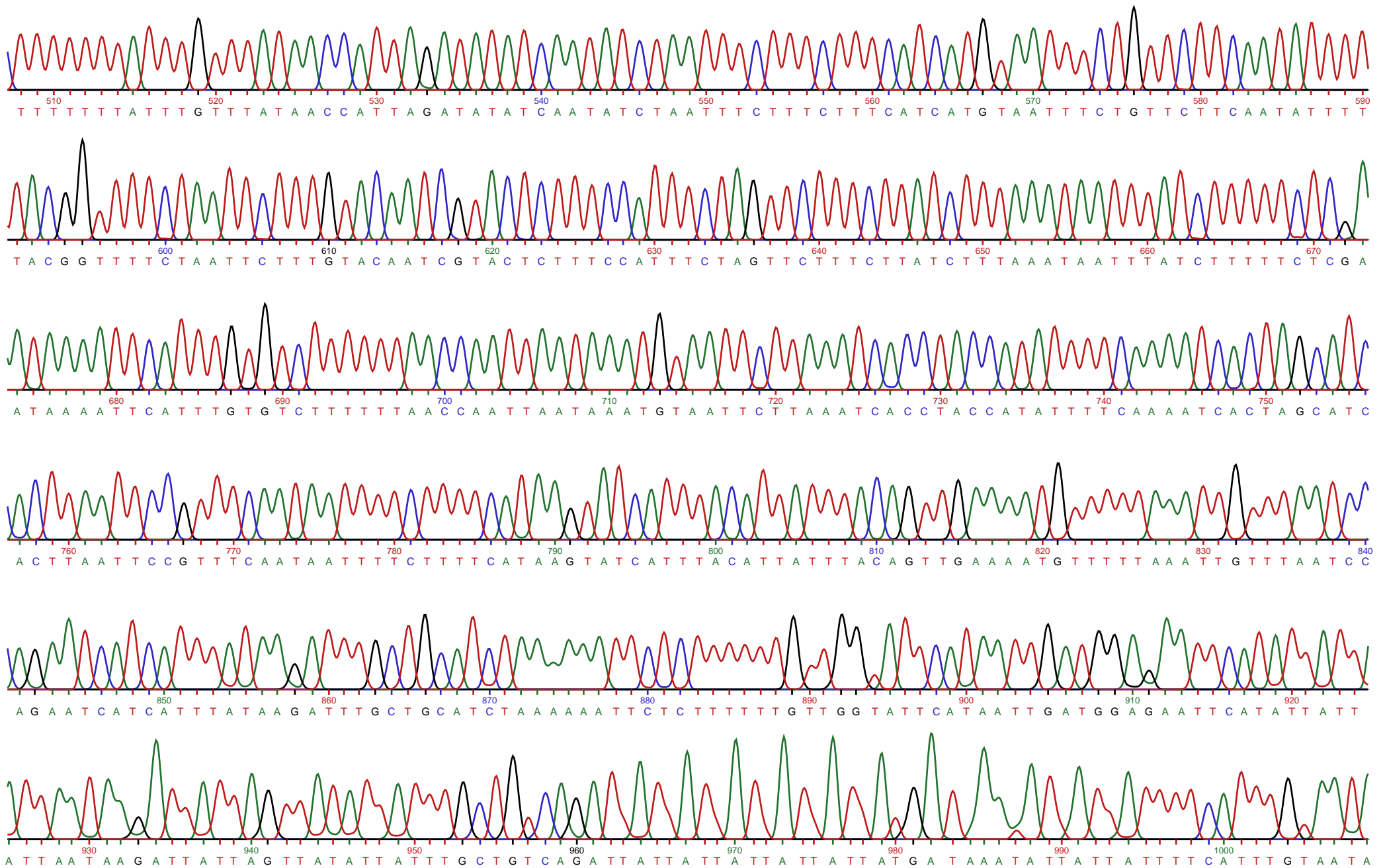

Samples: 12018  
Bases: 1010  
Average spacing: 12

Page: 3 / 3  
8/17/2022

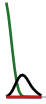

Supplement: Supporting information 2 — (ZIP) [file pone.0316479.s002.zip › 024KN2R_PREMIX_Plate_KELCH2_D12.pdf]

Samples: 13323  
Bases: 823  
Average spacing: 17

Page: 1 / 3  
8/17/2022

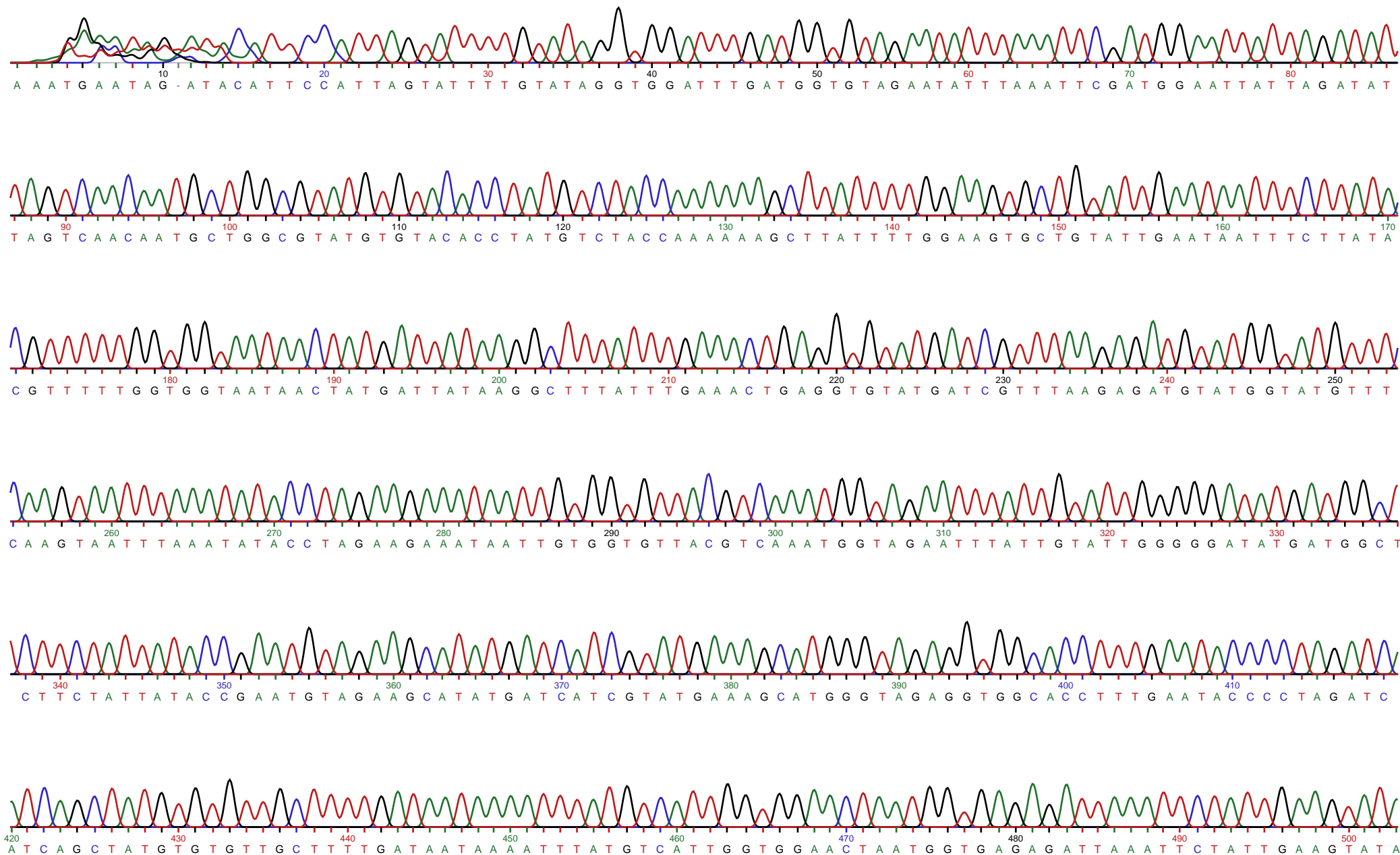

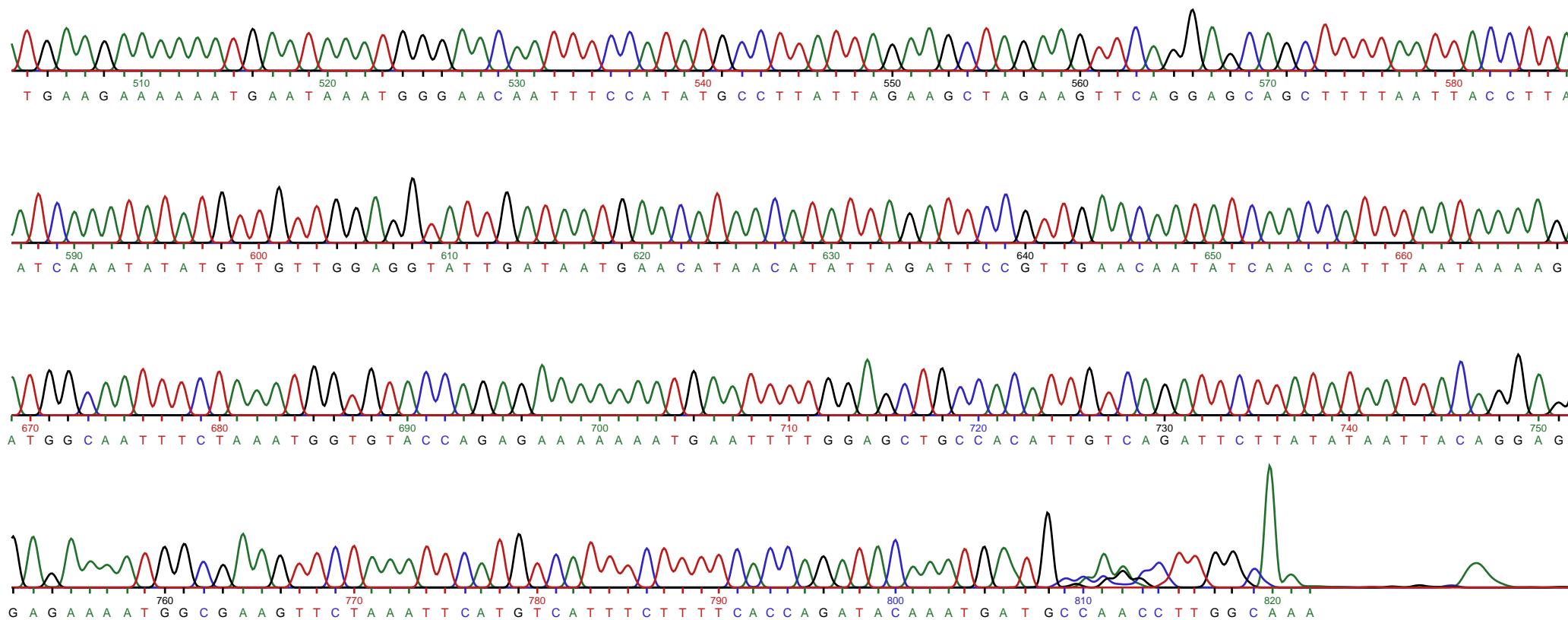

Supplement: Supporting information 2 — (ZIP) [file pone.0316479.s002.zip › 024KNIFW_PREMIX_Plate_CORKELCH_E08.pdf]

Samples: 13120  
Bases: 826  
Average spacing: 16

Page: 1 / 3  
8/17/2022

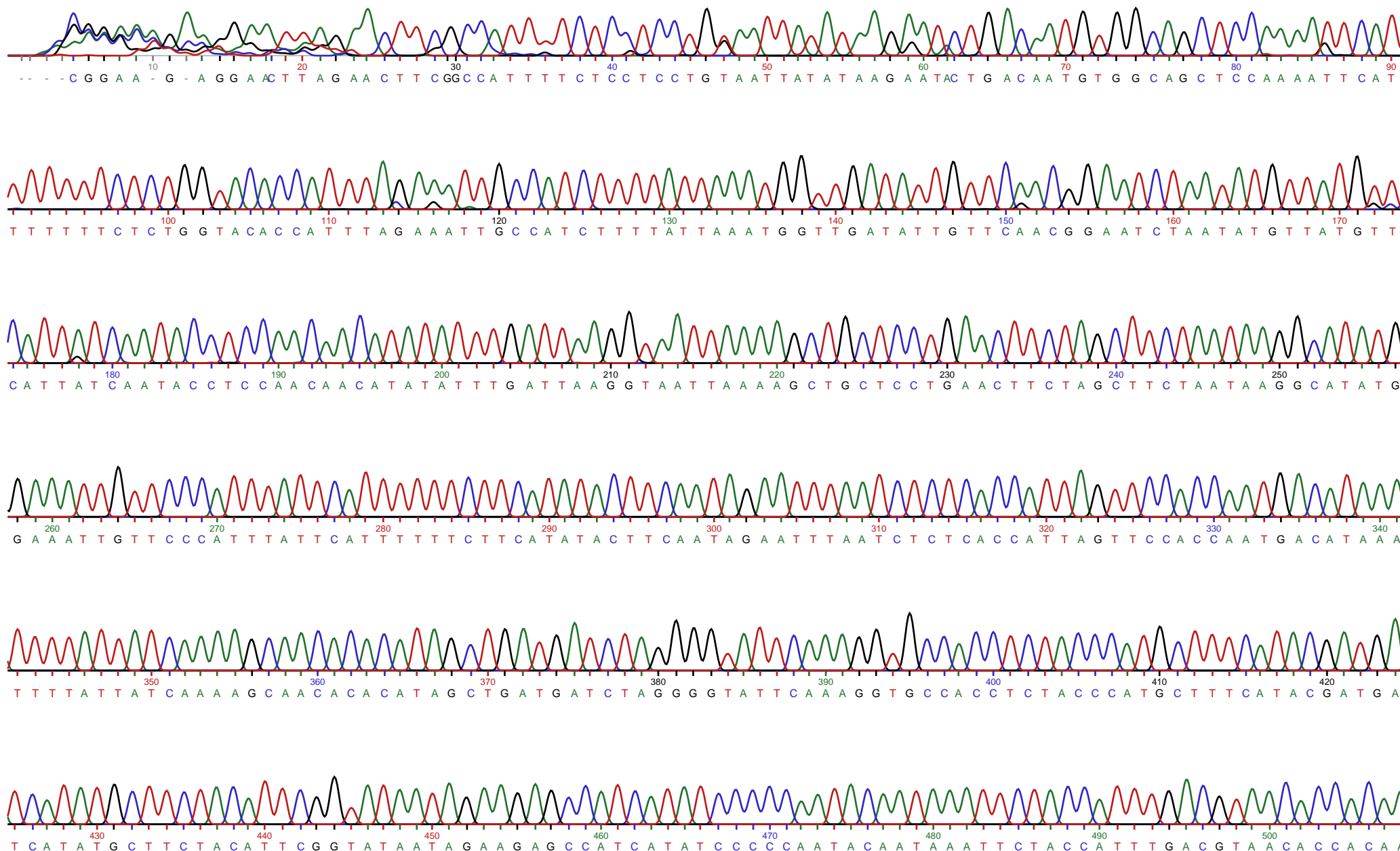

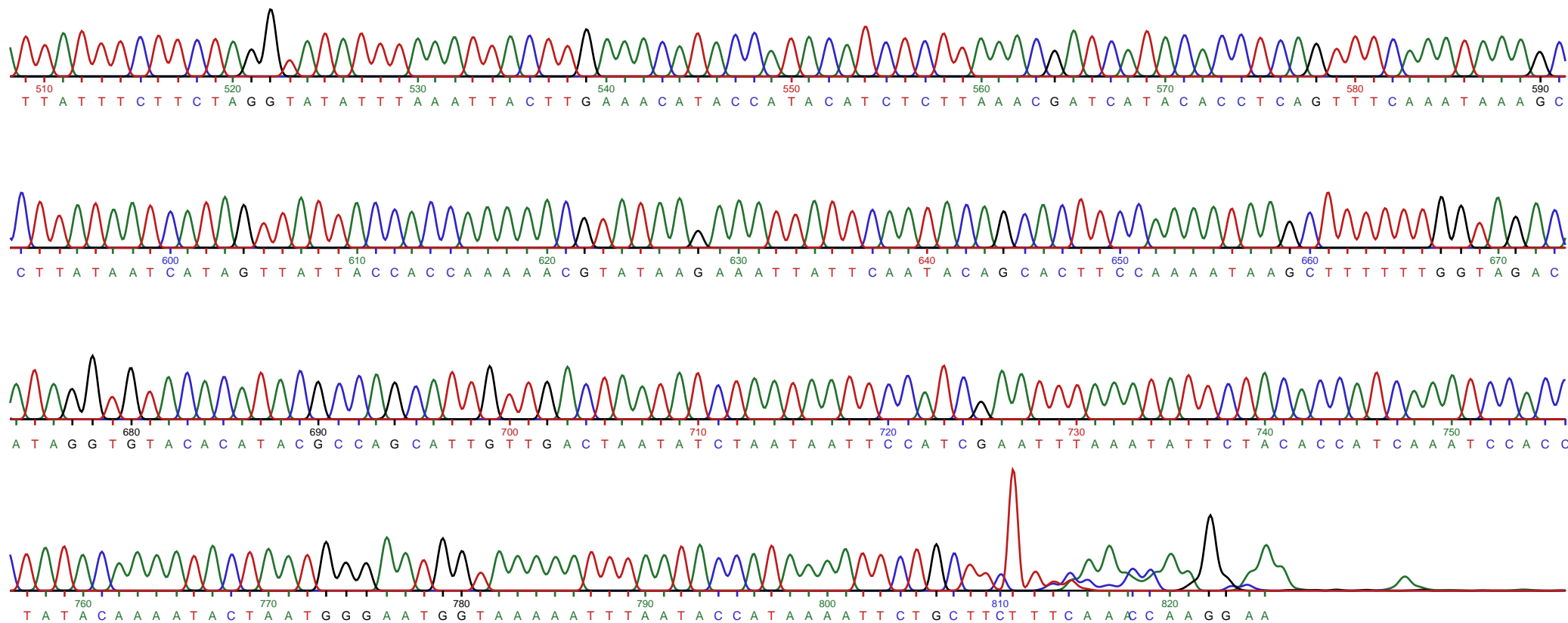

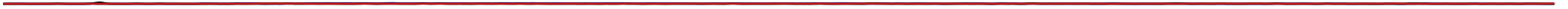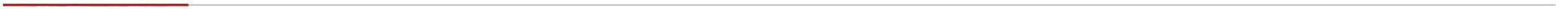

Supplement: Supporting information 2 — (ZIP) [file pone.0316479.s002.zip › 026KN1R_PREMIX_Plate_KELCH1_B11.pdf]

Page: 1 / 3  
8/17/2022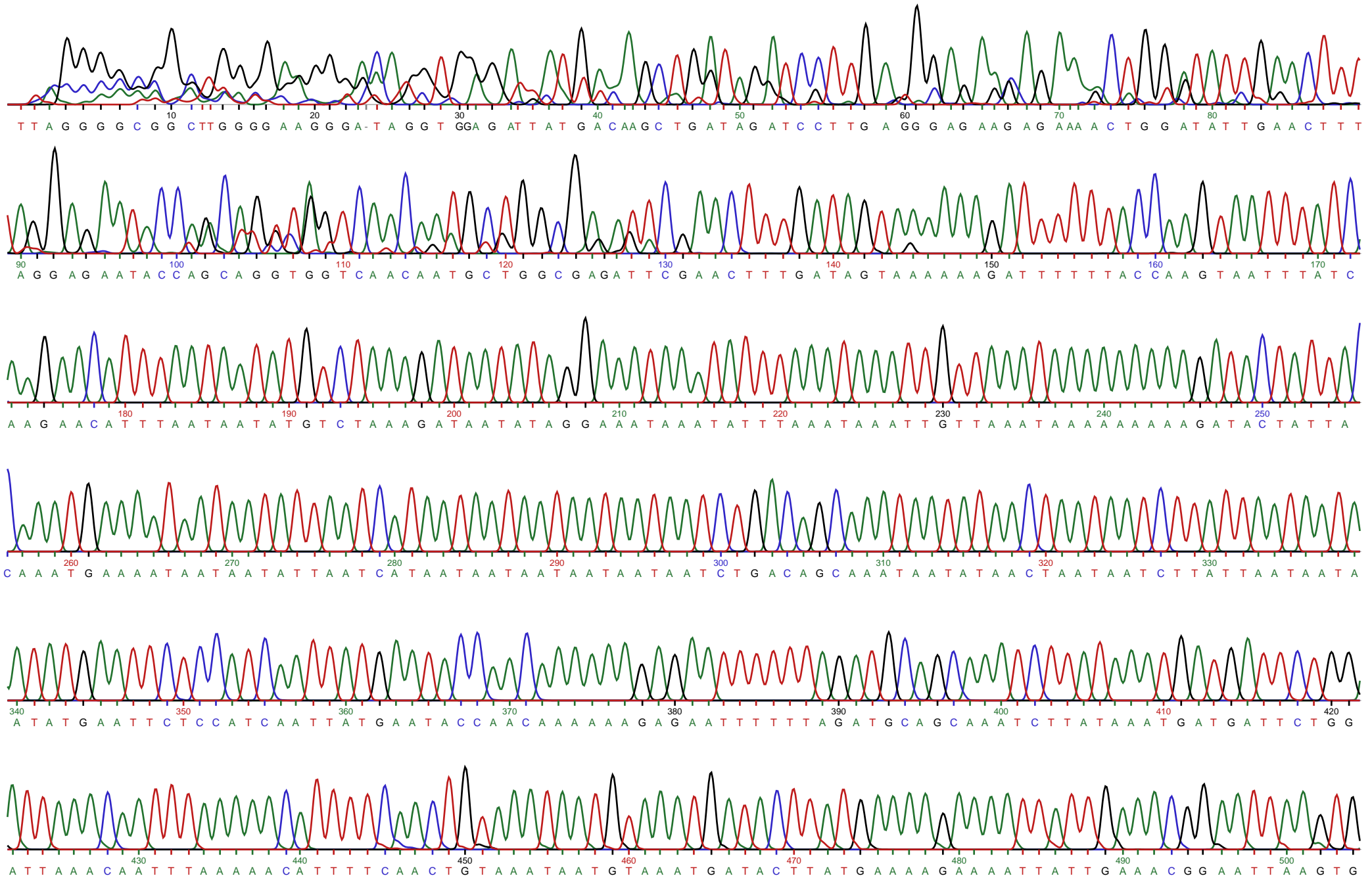

Samples: 13253  
Bases: 1076  
Average spacing: 13

Page: 2 / 3  
8/17/2022

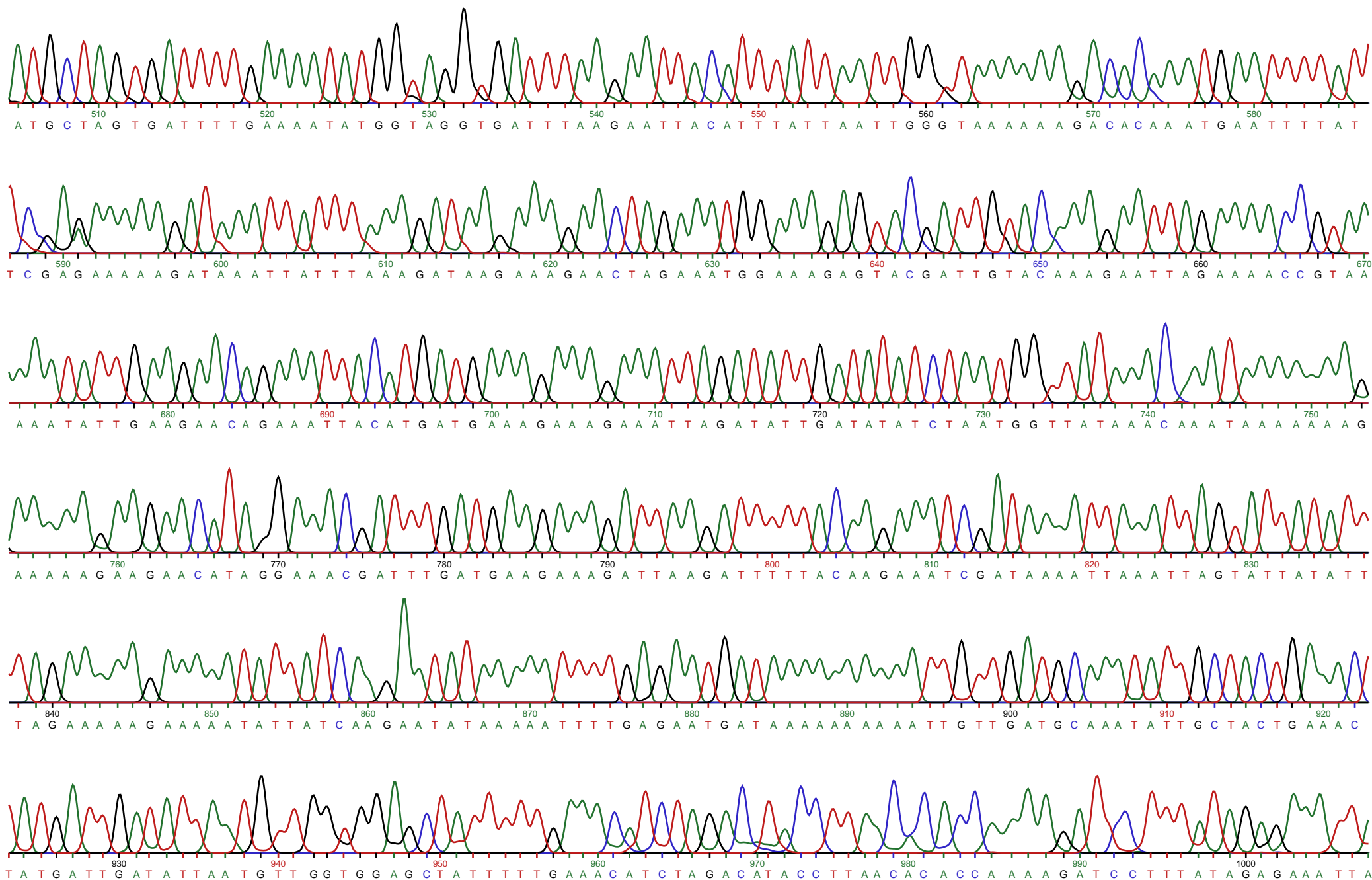

Samples: 13253  
Bases: 1076  
Average spacing: 13

Page: 3 / 3  
8/17/2022

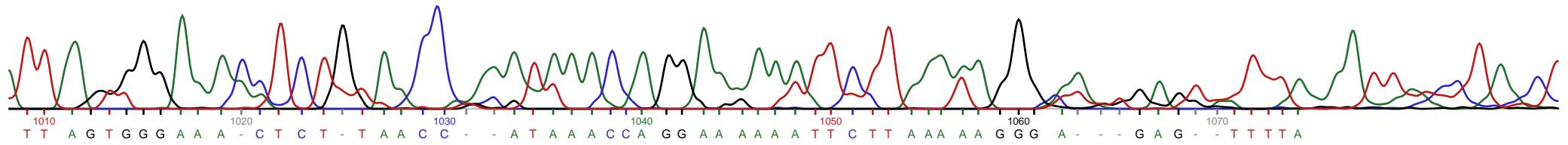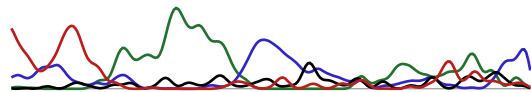

Supplement: Supporting information 2 — (ZIP) [file pone.0316479.s002.zip › 026KN2F_PREMIX_Plate_KELCH1_G11.pdf]

Samples: 13031  
Bases: 825  
Average spacing: 16

Page: 1 / 3  
8/17/2022

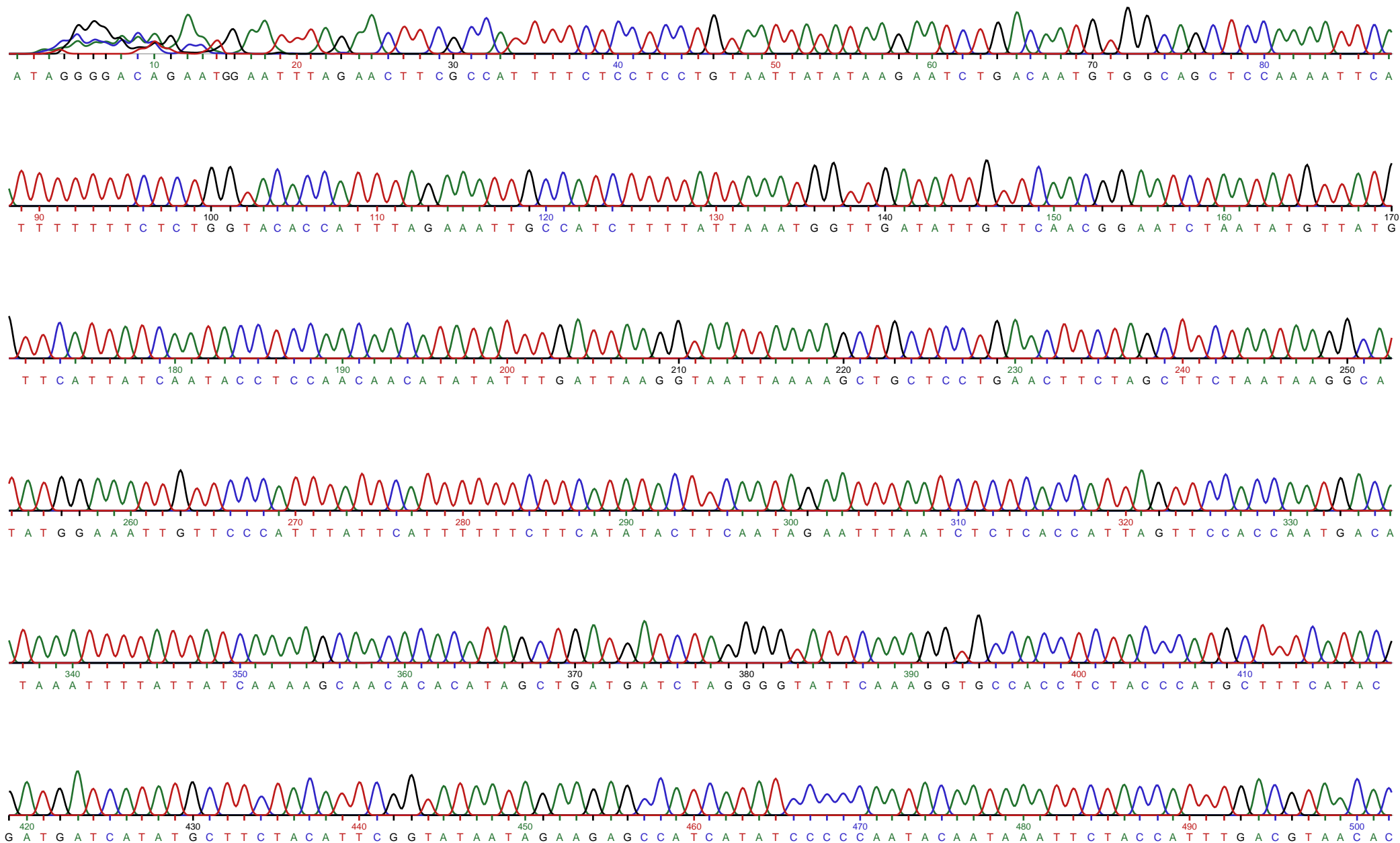

Samples: 13031  
Bases: 825  
Average spacing: 16

Page: 2 / 3  
8/17/2022

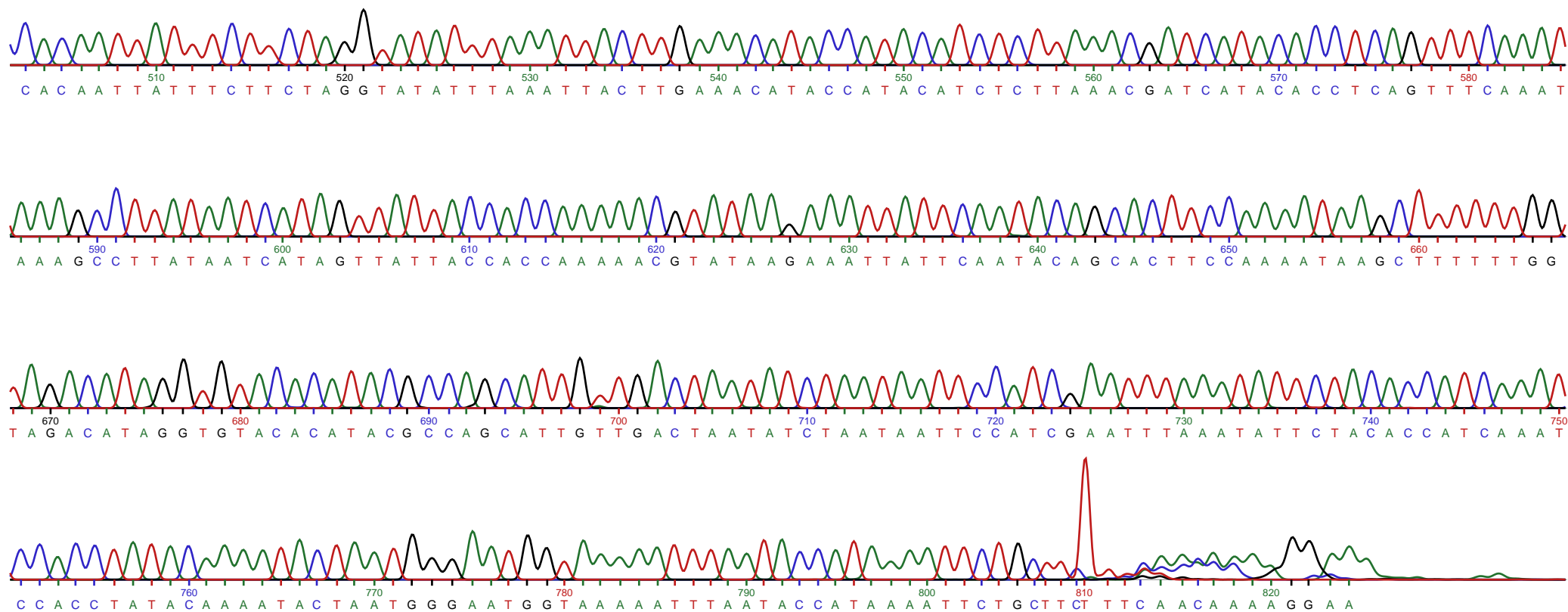

---

---

Supplement: Supporting information 2 — (ZIP) [file pone.0316479.s002.zip › 028KN1R_PREMIX_Plate_KELCH1_B12.pdf]

Samples: 13134  
Bases: 740  
Average spacing: 18

Page: 1 / 3  
8/17/2022

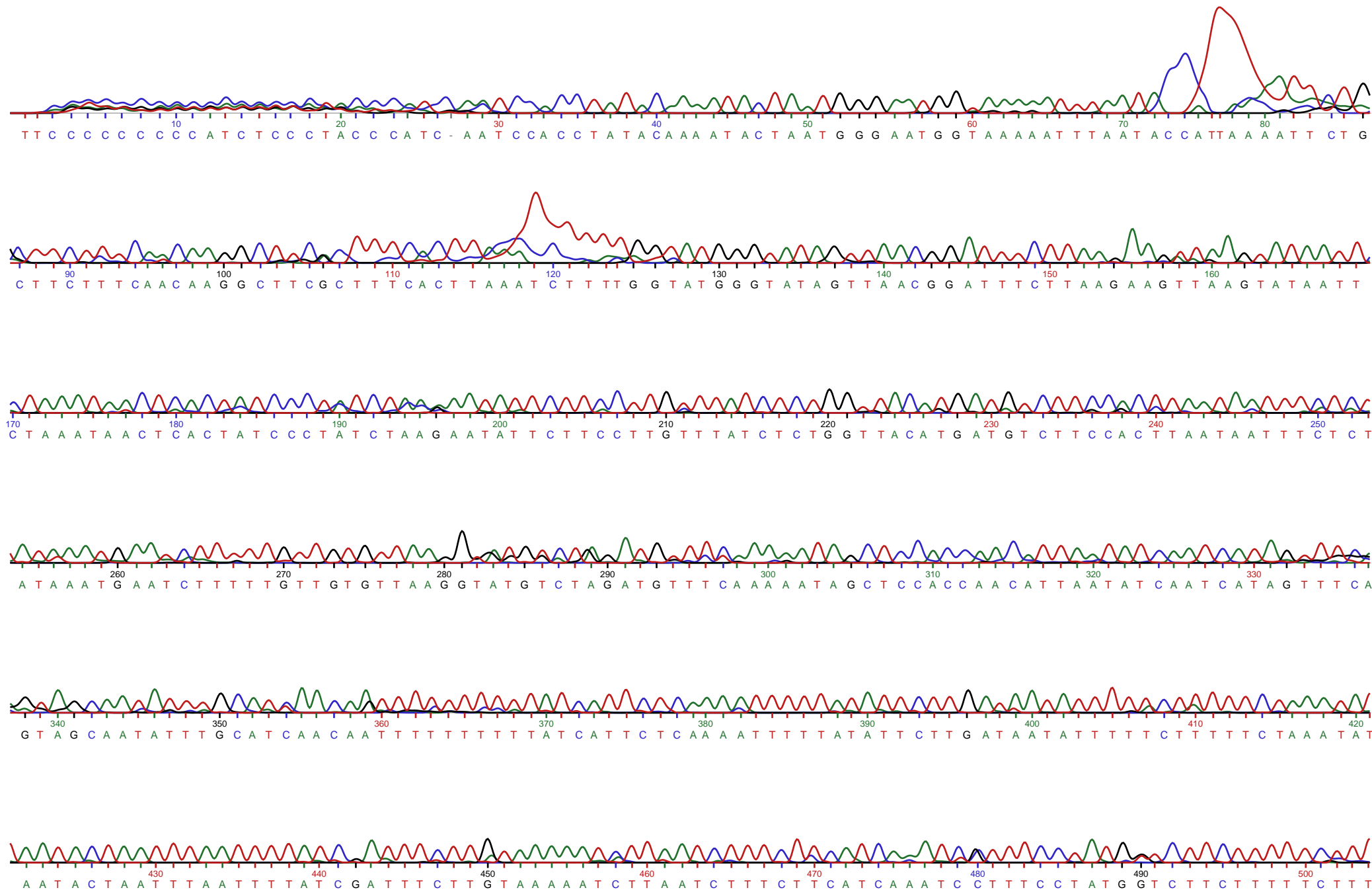

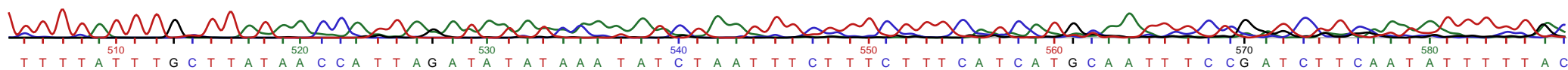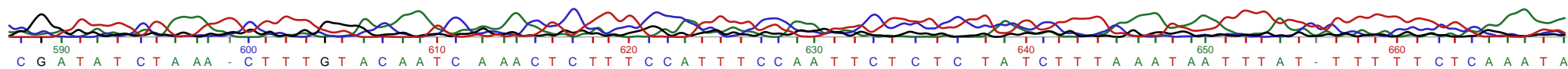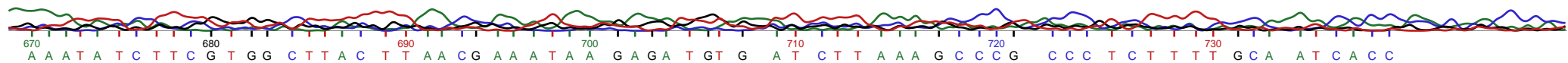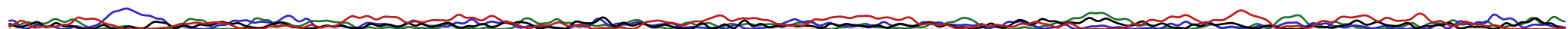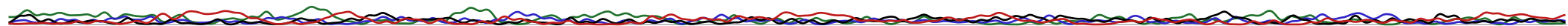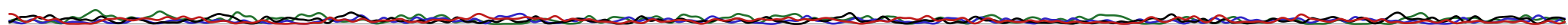

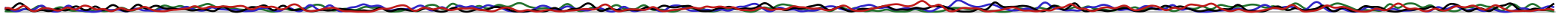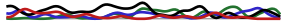

Supplement: Supporting information 2 — (ZIP) [file pone.0316479.s002.zip › 028KN2R_PREMIX_Plate_KELCH2_E02.pdf]

Samples: 12636  
Bases: 823  
Average spacing: 16

Page: 1 / 3  
8/17/2022

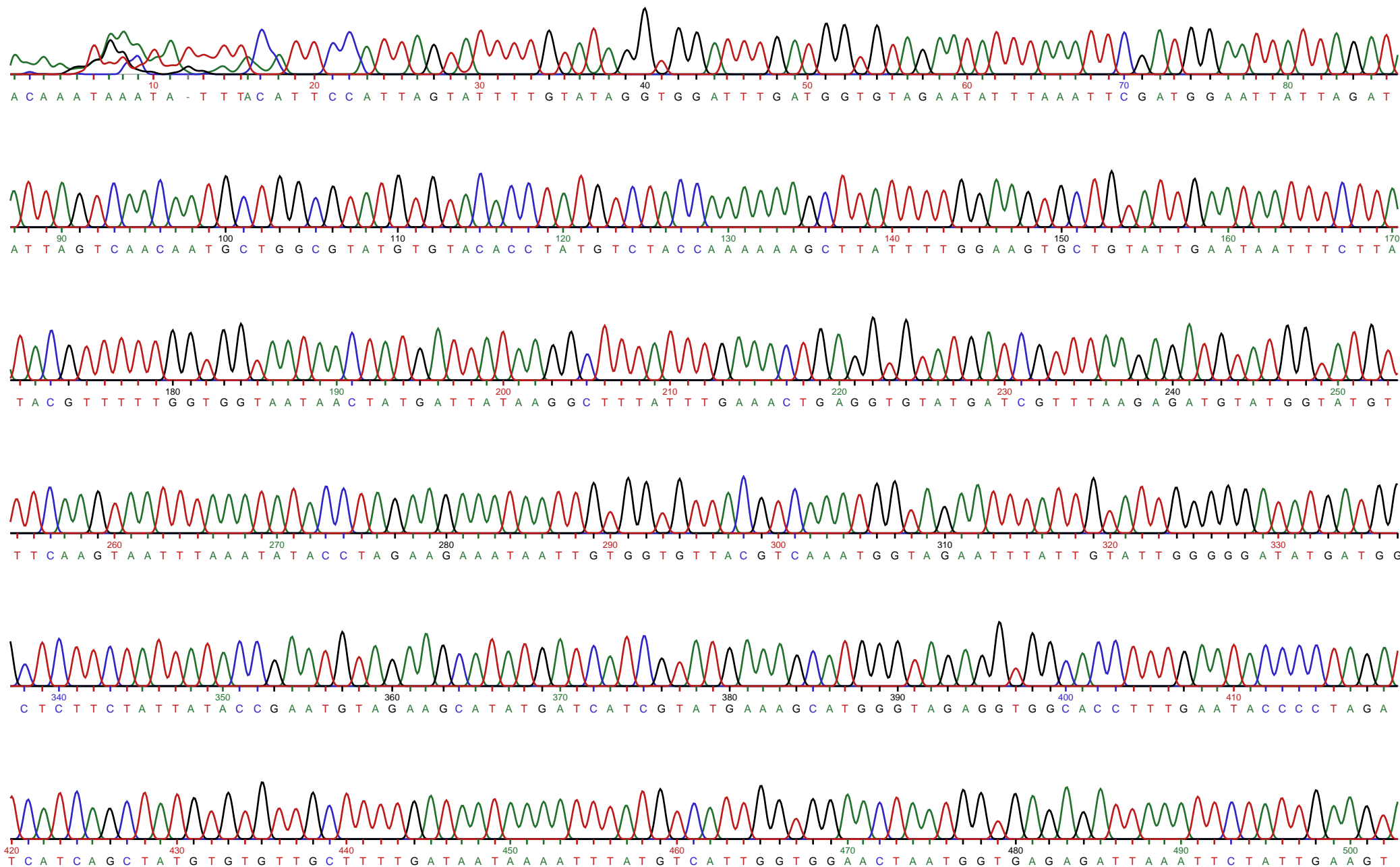

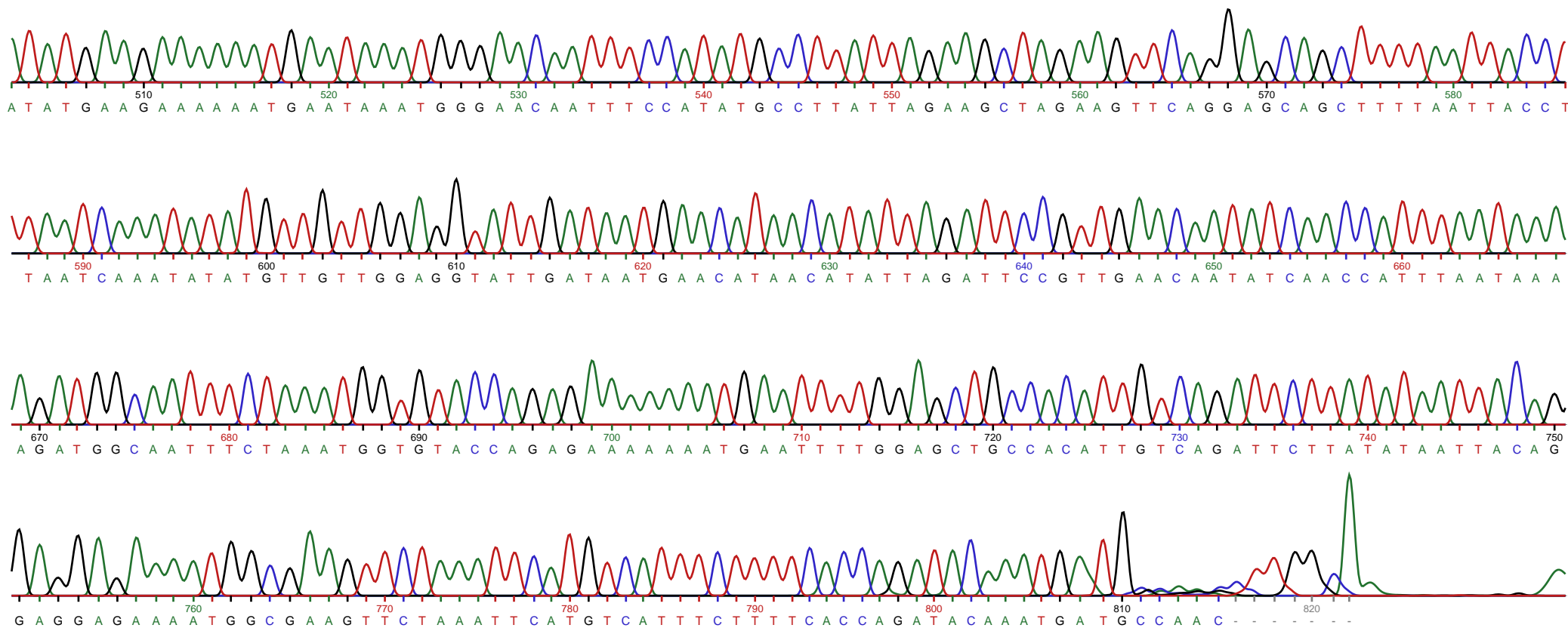

Samples: 12636  
Bases: 823  
Average spacing: 16

Page: 3 / 3  
8/17/2022

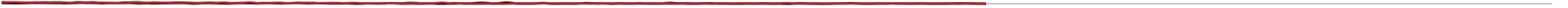

Supplement: Supporting information 2 — (ZIP) [file pone.0316479.s002.zip › 028KNIFW_PREMIX_Plate_CORKELCH_E10.pdf]

Samples: 13863  
Bases: 826  
Average spacing: 17

Page: 1 / 3  
8/17/2022

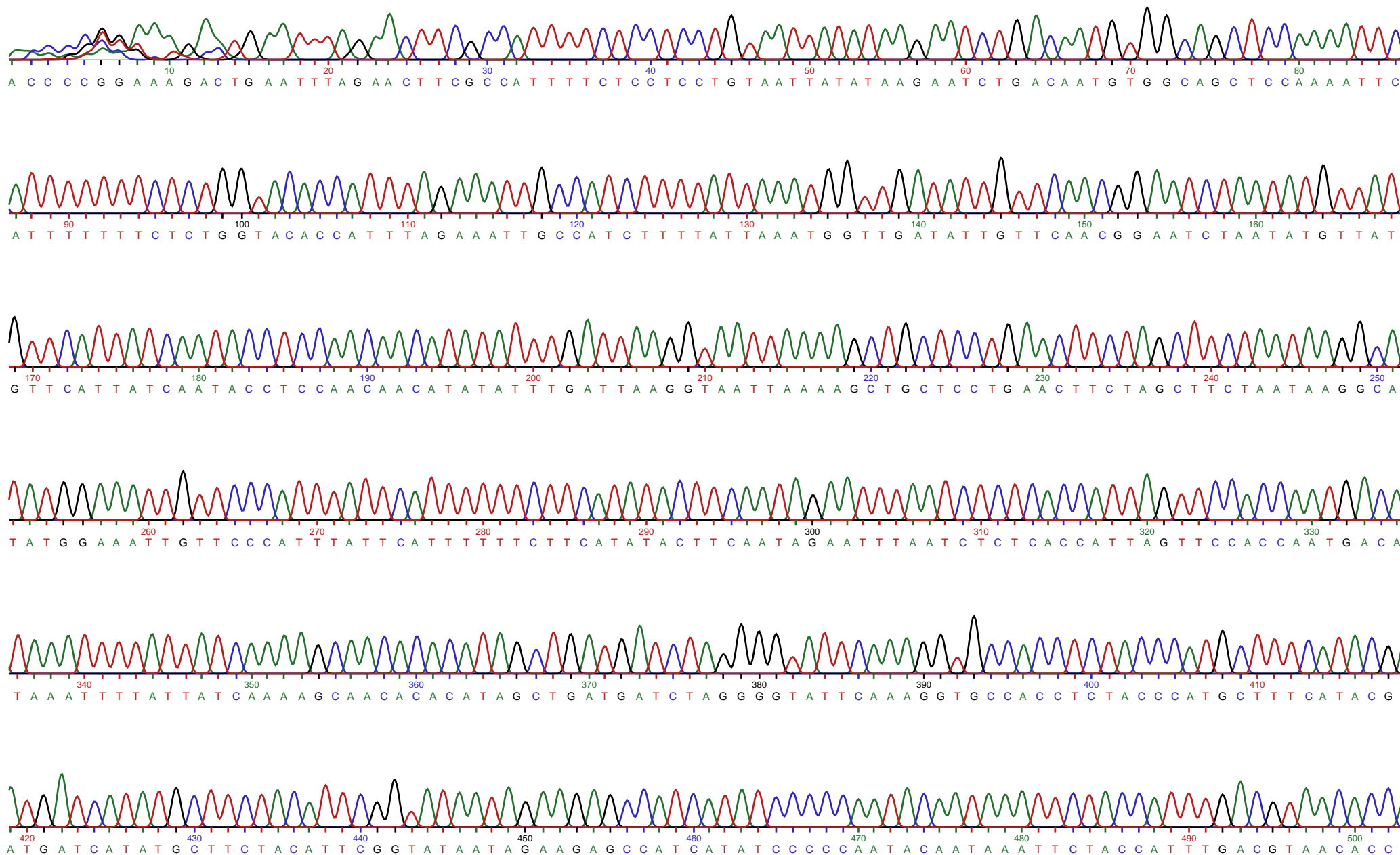

Samples: 13863  
Bases: 826  
Average spacing: 17

Page: 2 / 3  
8/17/2022

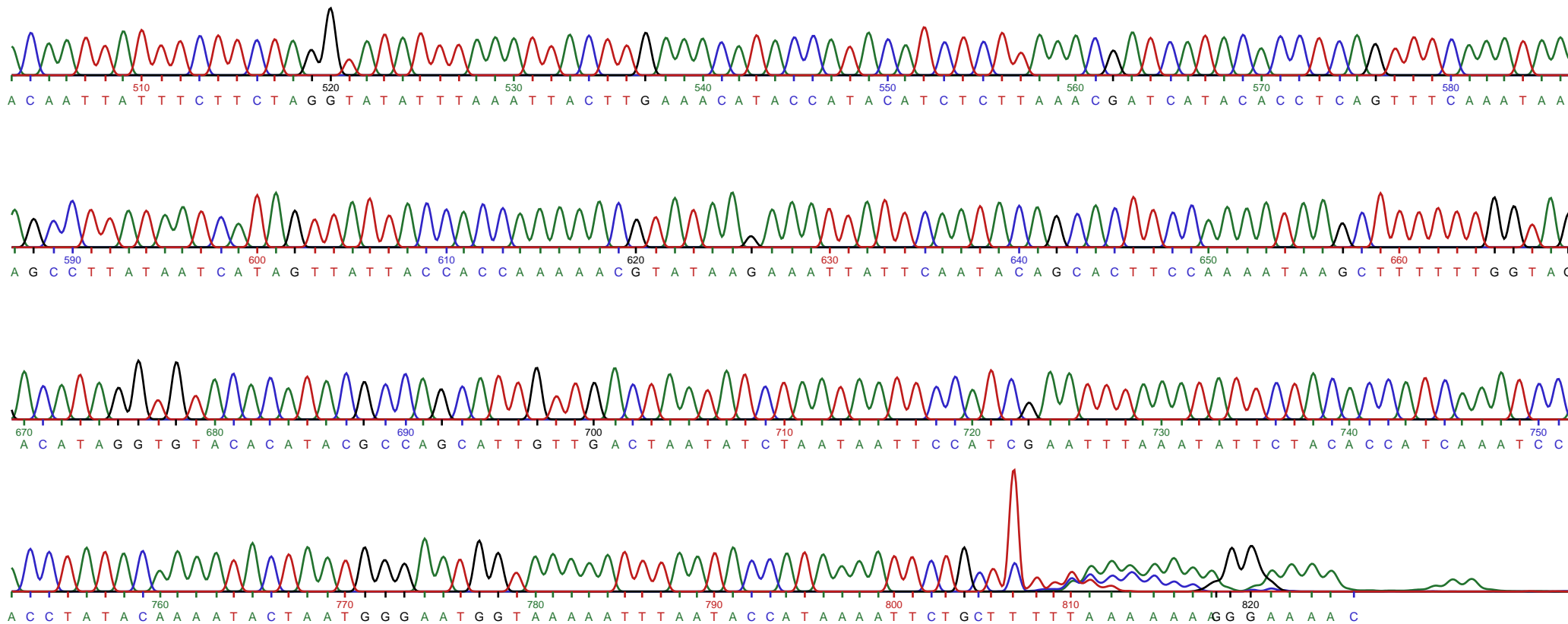

---

---

Supplement: Supporting information 2 — (ZIP) [file pone.0316479.s002.zip › 029KN1R_PREMIX_Plate_KELCH1_C01.pdf]

Page: 1 / 3  
8/17/2022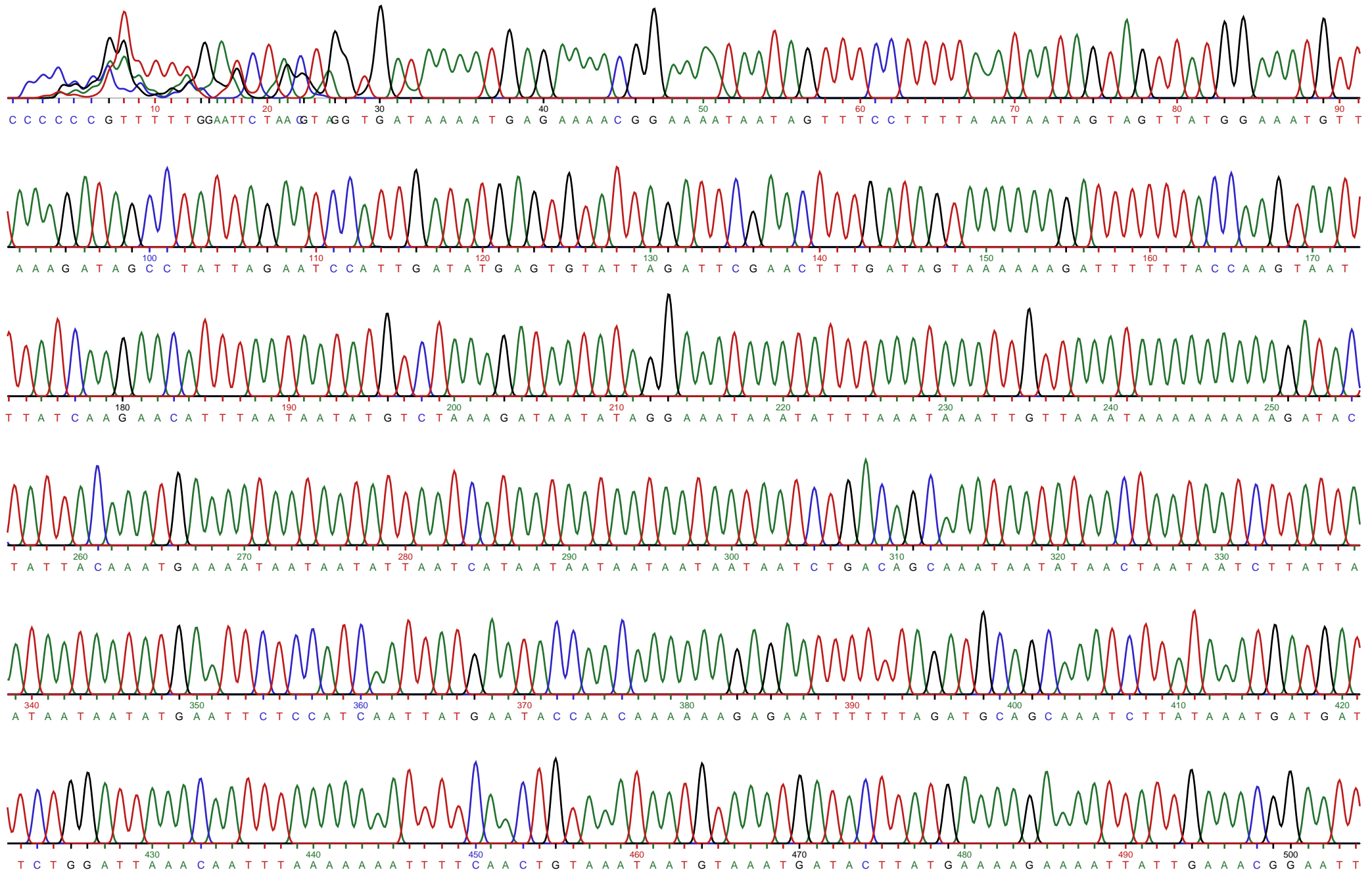

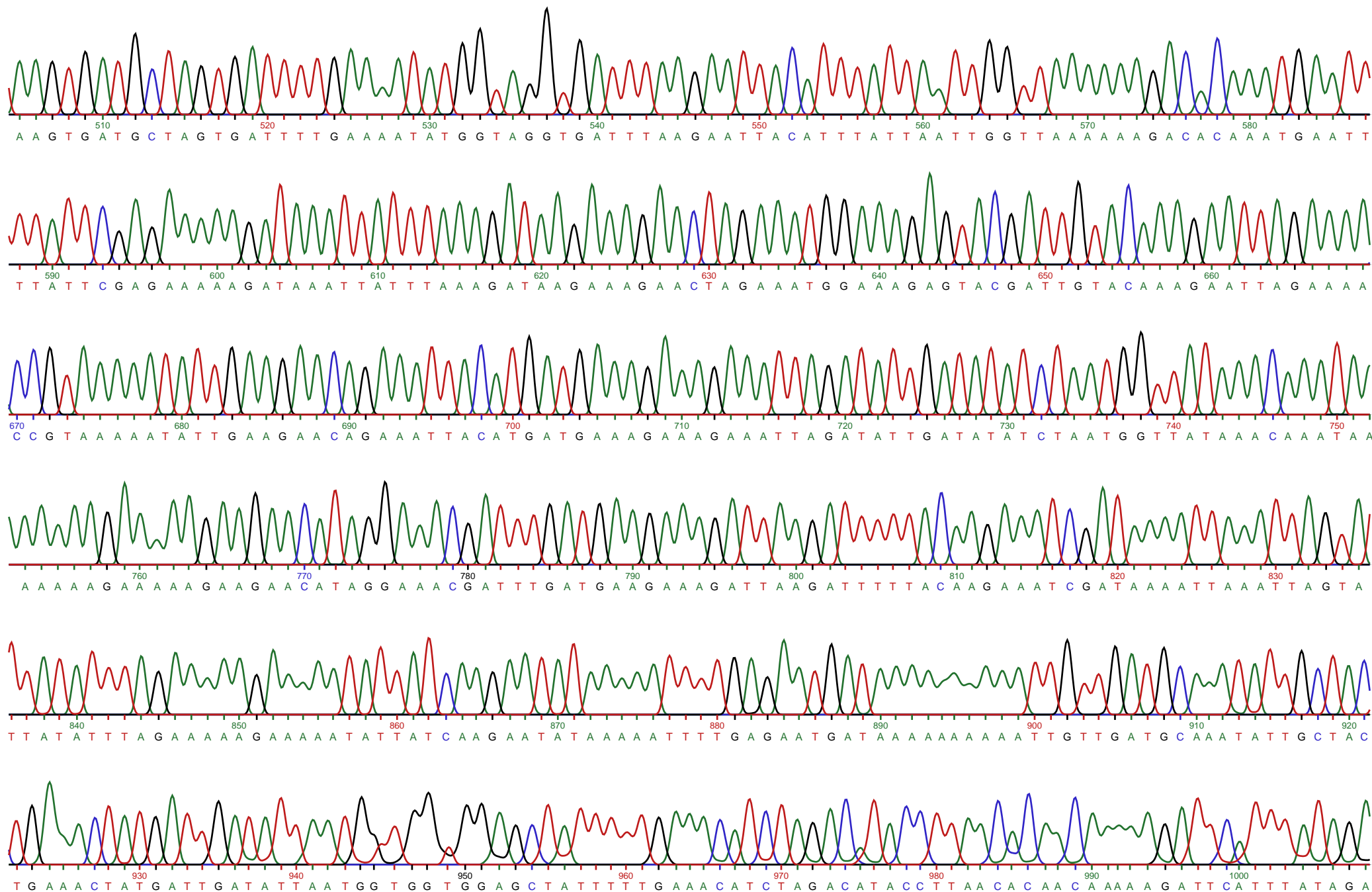

Samples: 13665  
Bases: 1151  
Average spacing: 12

Page: 3 / 3  
8/17/2022

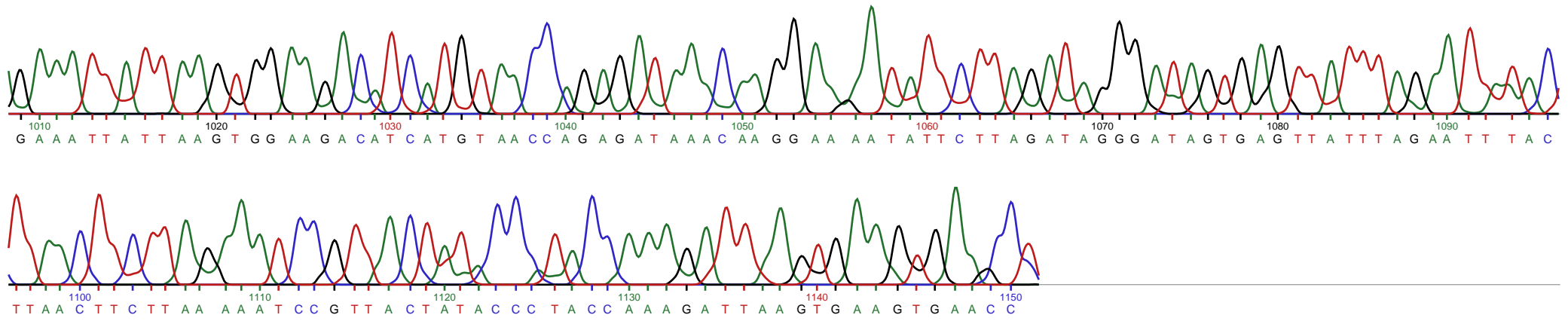

Supplement: Supporting information 2 — (ZIP) [file pone.0316479.s002.zip › 029KN2F_PREMIX_Plate_KELCH1_H01.pdf]

Samples: 13356  
Bases: 1132  
Average spacing: 12

Page: 1 / 3  
8/17/2022

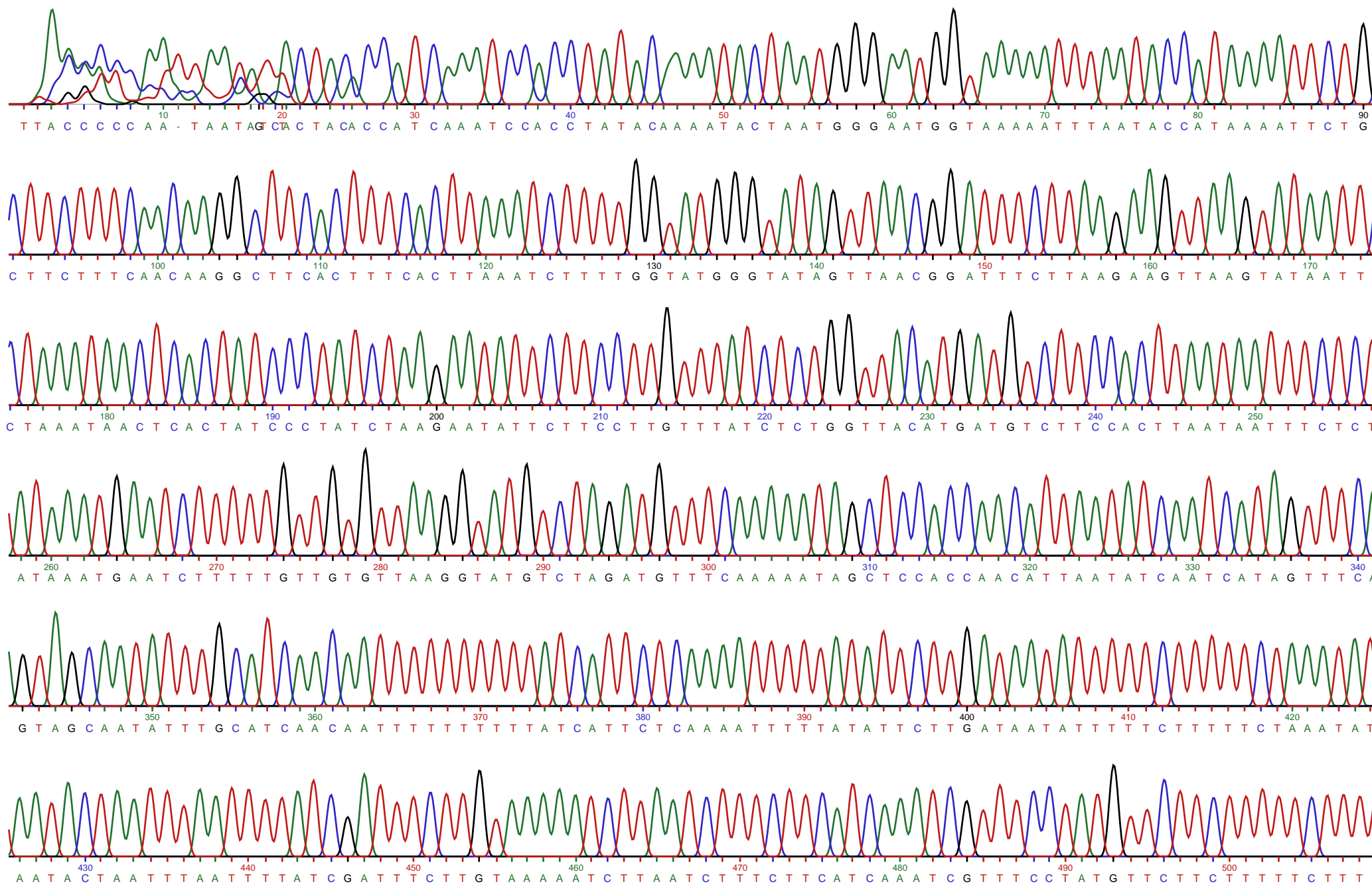

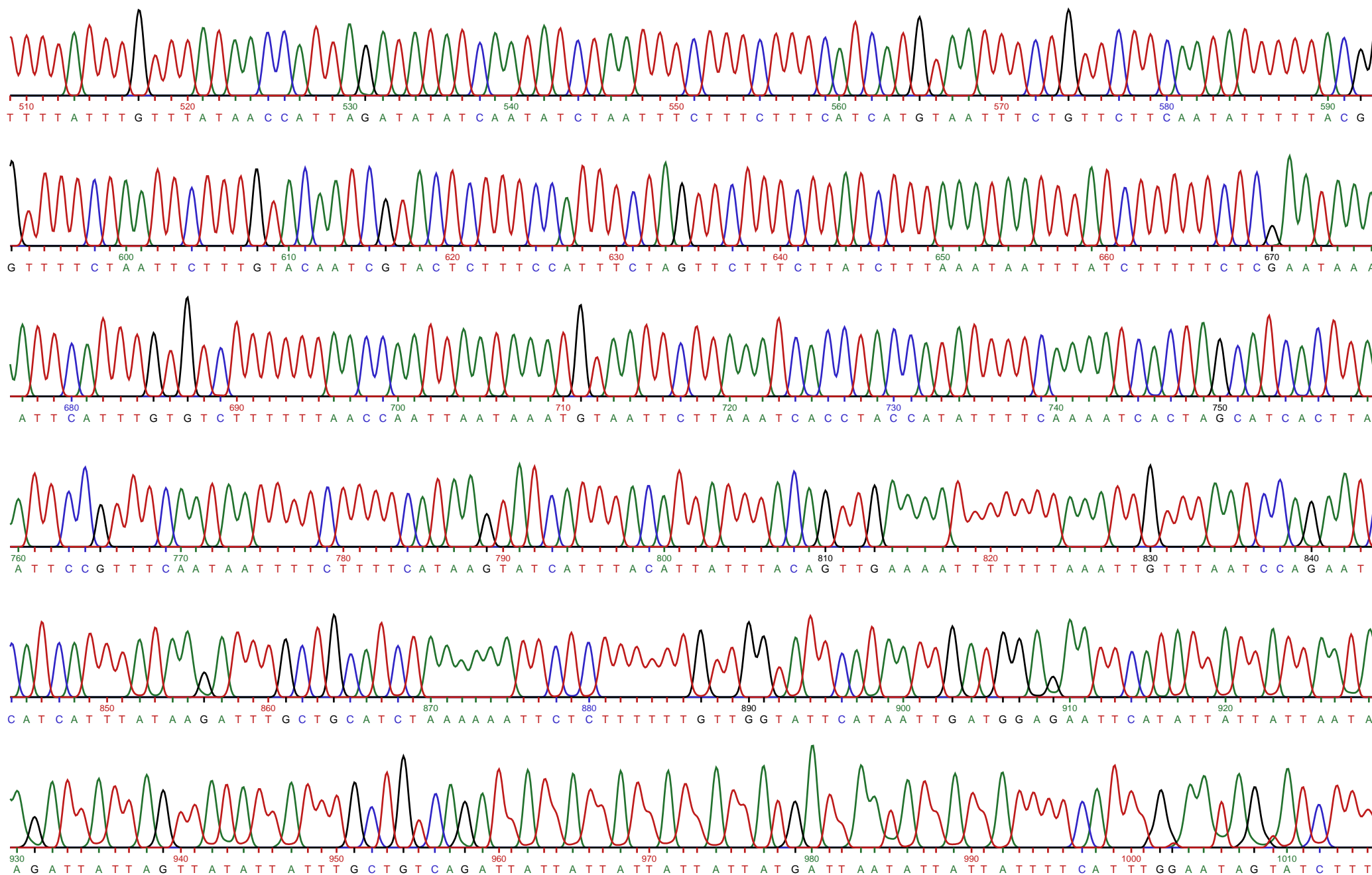

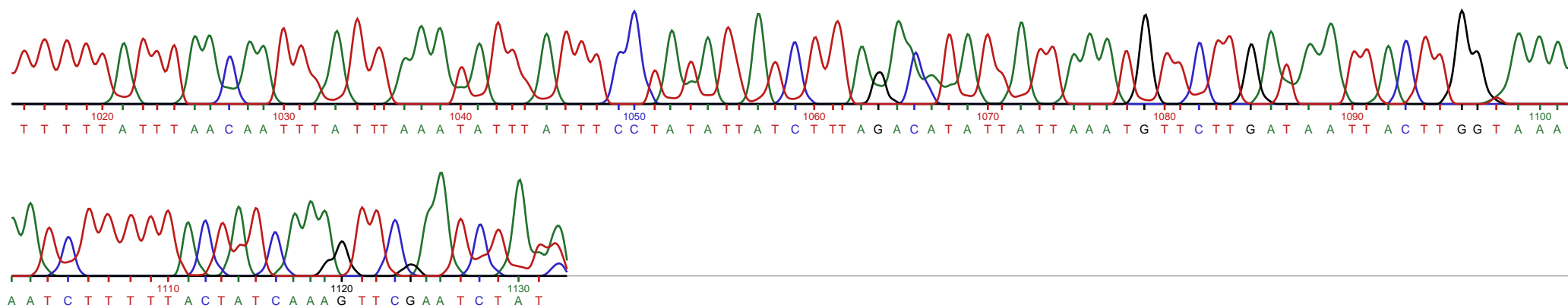

Supplement: Supporting information 2 — (ZIP) [file pone.0316479.s002.zip › 029KN2R_PREMIX_Plate_KELCH2_E03.pdf]
